# Peptidomic Characterization of < 3 kDa Peptides from Milk Fermented by Lactic Acid Bacteria: Sequence-Based Antimicrobial Prediction and *In Vitro* Immuno-Oncological Evaluation

**DOI:** 10.1007/s12602-026-10958-3

**Published:** 2026-03-23

**Authors:** Hale İnci Öztürk, Fadime Kiran, Melis Şardan Ekiz, Ömür Çelikbıçak, Sebnem Ozturkoglu-Budak

**Affiliations:** 1 Department of Food Engineering, Yıldız Technical University, Istanbul, Türkiye; 2 Pharmabiotic Technologies Research Laboratory, Department of Biology, Faculty of Science, Ankara University, Ankara, Türkiye; 3 Advanced Technologies Application and Research Center (HUNİTEK), Hacettepe University, Ankara, Türkiye; 4 Department of Chemistry, Hacettepe University, Beytepe, Ankara, Türkiye; 5 Department of Dairy Technology, Faculty of Agriculture, Ankara University, Ankara, Türkiye; 6 Faculty of Agriculture Department of Dairy Technology, Ankara University, Diskapi, Ankara, Türkiye

**Keywords:** Caseins, Serum proteins, Functional peptides, AMP, TNF-α, Anticancer

## Abstract

This study investigated the production of low-molecular-weight peptides (< 3 kDa) from milk proteins by endemic lactic acid bacteria strains and examined the effects of strain type, fermentation, and storage time on functional peptide formation to identify promising bioactive peptides and their producer strains for targeted functional dairy applications. To achieve this, < 3 kDa peptide fractions obtained from fermented milk with various LAB strains were characterized using LC-QTOF-MS. The highest peptides numbers were identified in samples fermented with *Lactobacillus (Lb.) helveticus*. β-casein–derived peptides predominated due to its high abundance in milk and flexible structure, which makes it more susceptible to proteolysis by LAB enzymes. This facilitates extensive peptide release during fermentation, and many of these fragments possess known antimicrobial activities, explaining their strong contribution to antibacterial potential. In vitro antibacterial activities were also assessed, and peptide sequences were scored for antibacterial potential using a Random Forest model. Random Forest analysis indicated that β-casein derived peptides were the main contributors to antibacterial potential. Serum albumin was hydrolyzed to a greater extent compared to other whey proteins. <3 kDa peptide fractions demonstrated inhibitory activity only against the Gram-negative bacteria *Salmonella* (*S.*) Typhimurium and *Escherichia* (*E.*) *coli*. The anticancer activities of the peptide fractions were evaluated against colorectal (HT-29), hepatocellular (Hep-G2), and breast (MCF-7) cancer cell lines, along with their immunomodulatory effects on RAW 264.7 macrophages. Anticancer activity was observed to be dose-dependent across all tested cell lines, with the most pronounced inhibitory effect generally detected at 50 µg/mL (*p* < 0.05). Peptide fractions derived from *L. paraplantarum* showed the strongest inhibitory effects against all tested cancer cell lines. Although no significant increase was observed in IL-12 and IL-6 levels, peptide fractions from *L. paraplantarum* and *Lb. helveticus* 11B2, enhanced TNF-α production in macrophages, indicating a controlled activation of macrophage-mediated innate immune responses that may contribute to antitumor immunity. This study demonstrates a clear strain-dependent divergence in peptide bioactivity, with *L. paraplantarum* NCCB 100,523 being associated with stronger anticancer and immunomodulatory effects, while *Lb. helveticus* 12B1 and *Lb. helveticus* 11B2 were linked to enhanced antibacterial activity, indicating their practical relevance for the targeted design of bioactive peptides and the development of functional foods with specific biological functions.

**Supplementary Information:**

The online version contains supplementary material available at 10.1007/s12602-026-10958-3.

## Introduction

In recent years, the increasing interest of consumers in natural and safe food has accelerated research on the identification of biologically active components in fermented foods. Bioactive peptides released by the hydrolysis of milk proteins by lactic acid bacteria (LAB) play a decisive role in the potential of fermented dairy products for human health [1]. Milk proteins such as caseins and whey proteins contain cryptic bioactive sequences in their structures, and their hydrolysis can release peptides with different functional properties [2]. The proteolytic activity of LAB enables the generation of small peptides from milk caseins and whey proteins, which may exert a range of biologically active effects [3]. Although it has been reported in the literature that these peptides exhibit various activities such as antimicrobial, antidiabetic, antihypertensive, antioxidant, immunomodulatory and anticancer, fractions in the < 3 kDa range have gained special importance recently due to their higher functional activity [4]. The high cellular membrane permeability and increased digestive resistance of low molecular weight peptides make this fraction attractive for both the food industry and pharmaceutical applications [5].

The proteolytic systems of various LAB species determine their peptide profile and hence their biological activity spectrum. Begunova, Savinova [6] reported that the number of peptides and bioactivity levels formed in milk fermented with *Lb. helveticus*,* Lacticaseibacillus rhamnosus* and *Limosilactobacillus reuteri* varied depending on the bacteria; the highest number and most functional peptides were obtained with *Lb. helveticus*. From an antimicrobial perspective, milk-derived peptides have been reported to exhibit broad-spectrum inhibitory activity against bacteria, fungi, and viruses, and they hold potential for use as natural preservatives in food systems [2]. Studies have shown that < 3 kDa peptides rich in cationic and hydrophobic residues cause pore formation by electrostatic interaction with bacterial membranes [7]. Furthermore, in silico antimicrobial peptide (AMP) prediction analyses of peptidomes derived from fermented milk have revealed a wide array of candidate peptide sequences with high predicted antibacterial potential [8].

Numerous peptides released from fermented milk proteins have been shown to exhibit not only antimicrobial activity but also immunomodulatory and even anti-cancer effects [9]. Certain peptides derived from fermented milk have been reported to exert direct effects on tumor cells as well as indirect anticancer effects mediated through the immune system [10]. Several studies have demonstrated that peptides, particularly those in the < 3 kDa fraction, can activate the innate immune response by stimulating the secretion of cytokines such as Tumor Necrosis Factor-alpha (TNF-α) and Interleukin-12 (IL-12) [11, 12]. In another study conducted by Matar, Valdez [13], the consumption of milk fermented with *Lb. helveticus* was shown to significantly increase the number of Immunoglobulin A (IgA)-secreting cells in the intestinal and respiratory mucosa of mice, while also slowing down and regressing the growth of subcutaneously implanted fibrosarcoma tumors. These biological effects have been associated with new peptides in milk resulting from the proteolytic activities of LAB.

Integrated use of peptidomics and bioinformatics has become essential for advancing the characterization of bioactive peptides from food sources. Peptidomics techniques based on high-resolution mass spectrometry, such as Liquid Chromatography–Quadrupole Time-of-Flight Mass Spectrometry (LC-QTOF-MS), simultaneously identify large numbers of peptide sequences in < 3 kDa peptide mixtures in food matrices [14]. These bioinformatic analyses predict which peptides may have antimicrobial activity based on their amino acid sequences and physicochemical characteristics [15]. In addition, cell-based biological tests also play a critical role in revealing the functional significance of candidate peptides identified by peptidomic screening [16]. Based on all these points, the present study employed selected autochthonous LAB strains, previously isolated from traditional cheeses and yogurts to ferment milk substrates, and subsequently characterized the resulting < 3 kDa peptide fractions through advanced peptidomic analysis using LC-QTOF-MS. The antimicrobial potential of the resulting peptide sequences was predicted bioinformatically with machine-learning algorithms. The < 3 kDa fractions were then assessed in vitro for anticancer activity against HT-29, HepG2, and MCF-7 cell lines and for immunomodulatory effects in RAW 264.7 macrophages.

## Materials and Methods

### Materials

In the present study, skimmed milk powder was supplied from the Dairy Technology Pilot Plant at Ankara University (Türkiye).

### LAB Strains and Culture Preparation for Fermentation

The LAB strains employed in this study were originally isolated from traditional yogurts [17] and raw milk cheeses [18]. A total of 13 LAB strains representing five genera were employed in this study, including *Lactobacillus* (*Lb. brevis* NCCB 100526, *Lb. helveticus* 9B5, 11B2, 12B1, *Lb. bulgaricus* 23), *Lactiplantibacillus* (*L. paraplantarum* NCCB 100523, *L. plantarum* NCCB 100524), *Loigolactobacillus (L. coryniformis* NCCB 100543), *Enterococcus* (*E. faecium* NCCB 100527, *E. faecalis* NCCB 100525), *Lactococcus* (*Lc. lactis* NCCB 100539), and *Streptococcus* (*S. thermophilus* K16, 1K4). Strain activation was performed using three different media, with De Man–Rogosa–Sharpe (MRS) broth employed for lactobacilli, M17 broth for lactococci and streptococci, and Kanamycin Aesculin (KA) broth for enterococci. For primary activation, lactobacilli were incubated in MRS broth at 37 °C for 72 h under anaerobic conditions, whereas enterococci and streptococci were incubated at 37 °C for 24–48 h under aerobic conditions, and lactococci at 30 °C for 24–48 h under aerobic conditions. Following incubation, the cultures were centrifuged at 6000 rpm for 10 min at 4 °C to obtain cell pellets. A second activation was carried out by inoculating 2% of the pellet into sterile (108 °C for 10 min) reconstituted skim milk (RSM) containing 11% total solids. The resulting pellets were washed twice with 0.85% sterile saline and resuspended in sterilized RSM with %11 total solids. The resuspended cultures were incubated again under the same conditions and subsequently used as cultures for fermentation.

### Fermentation of Reconstituted Skim Milk

Fermented milk was prepared by single-strain lactic acid fermentation. After completing second activation of cultures, each LAB strain was inoculated at 2% (v/v) into 3 L of freshly pasteurized RSM (11% total solids; 108 °C for 10 min). Incubation was conducted at 37 °C for lactobacilli, enterococci, and streptococci strains and at 30 °C for lactococci strain until the pH reached 4.60–4.70. Upon completion of fermentation, the samples were immediately cooled to 4 °C and stored under refrigeration for 28 days until further analyses. Pasteurized reconstituted milk without microbial inoculation was used as the control.

### Preparation of Water-Soluble Extracts from Fermented Milk

Water-soluble extracts (WSEs) of the fermented milk samples were prepared using a modified version of the method described by Sah, Vasiljevic [19]. For this purpose, the samples were centrifuged (Sartorius 3–18 K) at 7500 × g for 30 min at 4 °C to separate the casein-based macromolecules, and the resulting supernatant was collected. This supernatant was then filtered through a 0.45 μm membrane filter (Merck Millipore, PVDF). WSEs were obtained on days 1 and 28 of the storage period of fermented milk samples.

### Separation of < 3 kDa Peptides via Ultrafiltration

Low-molecular-weight (< 3 kDa) peptide fractions were obtained from the WSEs by ultrafiltration. For this purpose, centrifugation was performed at 14,600 × g for 30 min at 4 °C using centrifugal filter units with a 3 kDa molecular weight cut-off (Sartorius Vivaspin 20), and the resulting permeate was collected. The ultrafiltrated extracts were lyophilized overnight at − 58 °C under 0.5 mBar vacuum pressure and stored at − 20 °C until further analyses.

### Analysis of Low-Molecular-Weight Peptides by LC-QTOF/MS

A 66 mg of the lyophilized < 3 kDa peptide fractions was dissolved in 1 mL of distilled water and subsequently filtered through a 0.45 μm syringe membrane filter (ME Cellulose) prior to mass spectrometry analysis. Peptide identification was performed using LC-QTOF/MS analysis on an Electrospray Ionization–Quadrupole Time-of-Flight Mass Spectrometer (ESI-QTOF-MS) system coupled with a nano-liquid chromatography (nLC) unit. To ensure high mass accuracy, the instrument was calibrated according to the manufacturer’s instructions. Prepared samples were loaded onto a 2 cm × 100 μm i.d. trap column (Acclaim PepMap RSLC, Thermo Fisher). Peptide separation was performed using a high-pressure nano-LC system (Bruker NanoElute) coupled to a CaptiveSpray nano-ESI source and a C18 analytical column (25 cm length × 75 μm inner diameter, 1.9 μm particle size; PepSep). Each sample, containing 400 ng/mL of peptides dissolved in 2% (v/v) acetonitrile with 0.1% (v/v) trifluoroacetic acid, was injected in a volume of 1 µL and separated using an 80 min gradient. After loading with solvent A (0.1% formic acid), peptides were eluted at 0.4 µL min⁻¹ with solvent B (0.1% formic acid in acetonitrile) according to the following profile: 2–17% over 40 min, 17–25% over 20 min, 25–40% over 10 min, ramp to 80% in 5 min, and hold at 80% for 5 min. The column temperature was kept constant at 40 °C with a Peltier-controlled heater. Drying-gas temperature and flow were set to 180 °C and 4.0 L min⁻¹, respectively; capillary voltage was 4500 V and nebulizer pressure 0.4 bar. Spectra were acquired at a rate of 2 Hz, while MS/MS scan rates varied between 4 and 16 Hz depending on precursor ion intensity. Molecular precursor ions of peptide fragments were selectively fragmented in the collision-induced dissociation (CID) cell within the QTOF mass spectrometer using the Auto MS/MS mode. MS1 scans were performed over an m/z range of 350–2200 at a resolution of approximately 80,000, and Auto MS/MS scans were carried out over an m/z range of 150–2200 under the same resolution with active exclusion mode.

### Identification of Peptide Sequences

Raw data generated from LC-QTOF/MS analysis was processed using MaxQuant software (v2.1.4.0). The database search was conducted under nonspecific enzymatic digestion settings, with all other parameters retained as default. thionine oxidation and N-terminal acetylation were specified as variable modifications, while cysteine carbamidomethylation was defined as a fixed modification. Peptides exceeding a molecular mass of 3000 Da were excluded from the analysis. For confident peptide identification, the false discovery rate threshold was set at 1%, with precursor and fragment mass tolerances of 20 ppm and 10 ppm, respectively. Mass spectral data were searched against the UniProtKB database comprising validated sequences of major bovine milk proteins, including α_S1_-casein (P02662), α_S2_-casein (P02663), β-casein (P02666, Q9TVD0), κ-casein (P02668), serum albumin (P02769), α-lactalbumin (P00711), and β-lactoglobulin (P02754).

### Computational Modeling and MIC Assay of AMP Candidates

The antimicrobial potential of the identified peptide sequences was evaluated using the CAMP3-RF prediction model, a Random Forest-based classifier available through the CAMPR3 server [20]. Peptides predicted as antimicrobial by the CAMP3-RF model, which provides probability scores ranging from 0 to 1, were considered as potential AMP. In addition, antibacterial activity was also evaluated by in vitro determination of the minimum inhibitory concentration (MIC) of < 3 kDa WSE peptide fractions against selected pathogenic bacteria using a microdilution method modified from the protocol of Sevin, Basar [21]. The antimicrobial assay was conducted against selected reference pathogens, including Gram-negative strains such as *S*) Typhimurium (ATCC 13311) and *E. coli* (ATCC 25922) as well as the Gram-positive strain *Staphylococcus (St.) aureus* (ATCC 25923) and *Mycobacterium (M.) tuberculosis* (ATCC 7644). Serial dilutions of the samples (0.13–66.00 mg/mL) were prepared in BHI broth and dispensed into 96-well microplates, followed by inoculation with 4 log CFU/mL of each bacterial strain. Following 24 h of incubation at 37 °C, the MIC value was determined as the lowest concentration of the sample that completely inhibited visible growth of the tested pathogens.

### *In Vitro* Analysis of Anticancer Effects

Low-molecular-weight peptide fractions (< 3 kDa) were evaluated for their anticancer potential using human colorectal carcinoma (HT-29, ATCC HTB-38™), hepatocellular carcinoma (HepG2, ATCC HB-8065™), breast adenocarcinoma (MCF-7, ATCC HTB-22™) cell lines. Human keratinocyte cells (HaCaT, CLS Cell Lines Service, Cat. No. 300493) was included as a model of normal human epithelial cells to assess selective cytotoxic effects of low-molecular-weight peptide fractions (< 3 kDa) on non-cancerous cells. HT-29, MCF-7, and HaCaT cells were maintained in high-glucose DMEM, whereas HepG2 cells were cultured in low-glucose DMEM. Both media were supplemented with 10% fetal bovine serum and 1× penicillin–streptomycin. Cells were incubated at 37 °C in a humidified atmosphere containing 5% CO_2_.

Upon reaching approximately 80% confluence, cells were washed with 1× phosphate-buffered saline (PBS; Sartorius, Germany), detached using 0.25% trypsin–EDTA (Sartorius, Germany), and centrifuged at 200 × g for 5 min. After staining with trypan blue (Sartorius, Germany), viable cells were counted using a TC20 Automated Cell Counter (Bio-Rad, USA). For the viability assay, cells were seeded into 96-well plates at a density of 1 × 10⁵ cells per well and allowed to attach overnight before being treated for 48 h with peptide fractions at four protein-based concentrations (1, 5, 10, and 50 µg/mL). Peptide concentrations were normalized according to total protein content, with the control fraction representing the < 3 kDa permeate obtained from non-fermented milk. Untreated cells were used as negative control. Cell viability was assessed using the MTT assay (3-(4,5-dimethylthiazol-2-yl)−2,5-diphenyltetrazolium bromide). Briefly, 10 µL of MTT solution (5 mg/mL in PBS) was added to each well and incubated for 3 h at 37 °C. Formazan crystals formed by metabolically active cells were dissolved by adding 100 µl of dimethyl sulfoxide, and absorbance was read at 570 nm using a microplate reader.

### ELISA-Based Analysis of Immunomodulatory Effects

The murine monocyte/macrophage-like cell line RAW 264.7 (ATCC TIB-71™) was used for immunological assays. Lipopolysaccharide (LPS) from *E. coli* O111:B4 was employed as positive control to induce proinflammatory cytokine production in RAW 264.7 cells. Cells were cultured under the same conditions and media as described previously. First, the concentration of LPS that exhibited no cytotoxic effects on RAW 264.7 cells was determined using the MTT assay, as previously described. In addition, the cytotoxic effects of the low-molecular-weight peptide fractions (< 3 kDa) at various concentrations on macrophage cells were also evaluated. To assess the immunomodulatory effects, cells were seeded into 96-well plates at a density of 1 × 10⁵ cells per well and treated with peptide fractions at four protein-based concentrations (1, 5, 10, and 50 µg/mL) for 24 h. Following incubation, culture supernatants were collected, and levels of the cytokines TNF-α, IL-6, and IL-12 were quantified using commercially available ELISA kits according to the manufacturer’s instructions (ElabScience, USA). Cytokine concentrations were calculated based on standard curves.

### Statistical Analysis

All analyses were performed in triplicate. Data obtained from anticancer and immunomodulatory effect assays were analyzed using one-way analysis of variance (ANOVA) followed by Dunnett’s and Tukey’s multiple comparison tests in GraphPad Prism version 3.0 (GraphPad Software, San Diego, CA, USA). Results are presented as mean ± standard deviation (SD). Statistical significance was set at *p* < 0.05 and is indicated as follows: * (*p* < 0.05), ** (*p* < 0.01), *** (*p* < 0.001), **** (*p* < 0.0001), and ns for not significant (*p* > 0.05).

## Results and Discussion

### Peptide Profiling and Bioinformatic Characterization of < 3 kDa Fractions

Peptides with a molecular weight < 3 kDa were isolated from fermented milk samples throughout the storage period. In this study, the < 3 kDa fraction was specifically targeted, as milk protein–derived bioactive peptides are predominantly low-molecular-weight, short-chain sequences (2–20 amino acid residues) that are frequently associated with biological activities such as antimicrobial, antihypertensive, and antioxidant effects, largely due to their structural accessibility and capacity to interact efficiently with biological targets [2]. The peptides obtained for each fermented sample are provided in Supplementary Tables 1–13. No peptides were detected in the unfermented control sample. Studies in the literature have reported the presence of peptides in milk [22]. However, these peptides have also been reported to be of high molecular weight [23]. Therefore, the isolation of peptides in the < 3 kDa range in this study may have resulted in the absence of detectable peptides in the control milk sample.

**Fig. 1:**
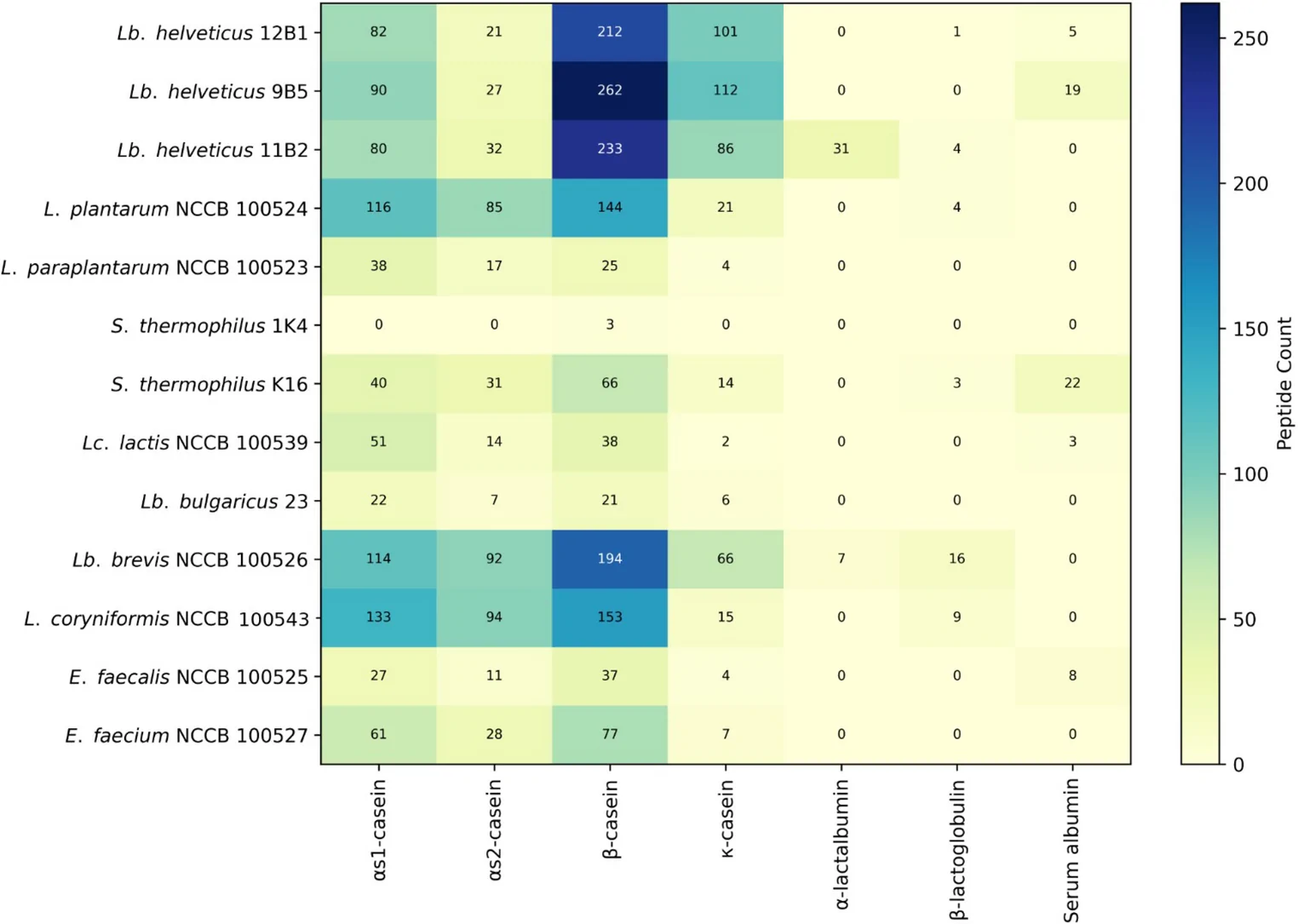
Heatmap showing the peptide counts and their distribution from different milk proteins by LAB isolates

The number of peptides generated from milk proteins varied markedly among LAB isolates, indicating pronounced species - and strain-dependent differences in proteolytic activity (Fig. 1). Overall, the highest peptide yields were observed in *Lb. helveticus* strains and *Lb. brevis*, whereas substantially lower peptide production was detected in certain strains such as *S. thermophilus* 1K4. Peptides originated mainly from α_s1_-, α_s2_-, β-, and κ-caseins, while whey protein-derived peptides were detected to a lesser extent, depending on the isolate. These findings have revealed that proteolytic activity on milk proteins varies from species to species and even from strain to strain. Previous studies have also reported that the ability of LAB to produce peptides from food proteins varies from strain to strain depending on the different proteinases they possess [24].

The peptide results obtained showed that the highest proteolytic activity was in *Lb. helveticus* strains (especially 9B5) and *Lb. brevis*, followed by *L. coryniformis* and *L. plantarum*. On the other hand, the lowest proteolytic activity was determined in *S. thermophilus* 1K4 strain, followed by *Lb. bulgaricus* 23 and *E. faecalis* NCCB 100,525. The proteolytic system of *Lb. helveticus* includes cell envelope-associated proteinases (CEP), peptide transport systems, and intracellular peptidases, which together enable efficient degradation of milk proteins.

In the present study, peptides generated by *Lb. helveticus* isolates were predominantly derived from β-casein, followed by κ-, α_s1_-, and α_s2_-caseins, a pattern consistent with the substrate specificity of *Lb. helveticus* CEPs by carboxypeptidase activity, along with contributions from endopeptidase, tripeptidase, and aminopeptidase activities. Previous studies have shown that CEPs from *Lb. helveticus* extensively hydrolyze β-CN and also hydrolyze Ƙ-, α_s1_-, and α_s2_-CN. Besides, the *Lb. helveticus* strains were found to be more effective on serum albumin among the whey proteins, making this the first study to report on a relationship between *Lb. helveticus* and serum albumin. These strains were found to act on the C-terminal, central, and N-terminal regions of serum albumin without apparent selectivity, and notably, to generate tripeptides from serum albumin, through tripeptidase activity.

**Fig. 2:**
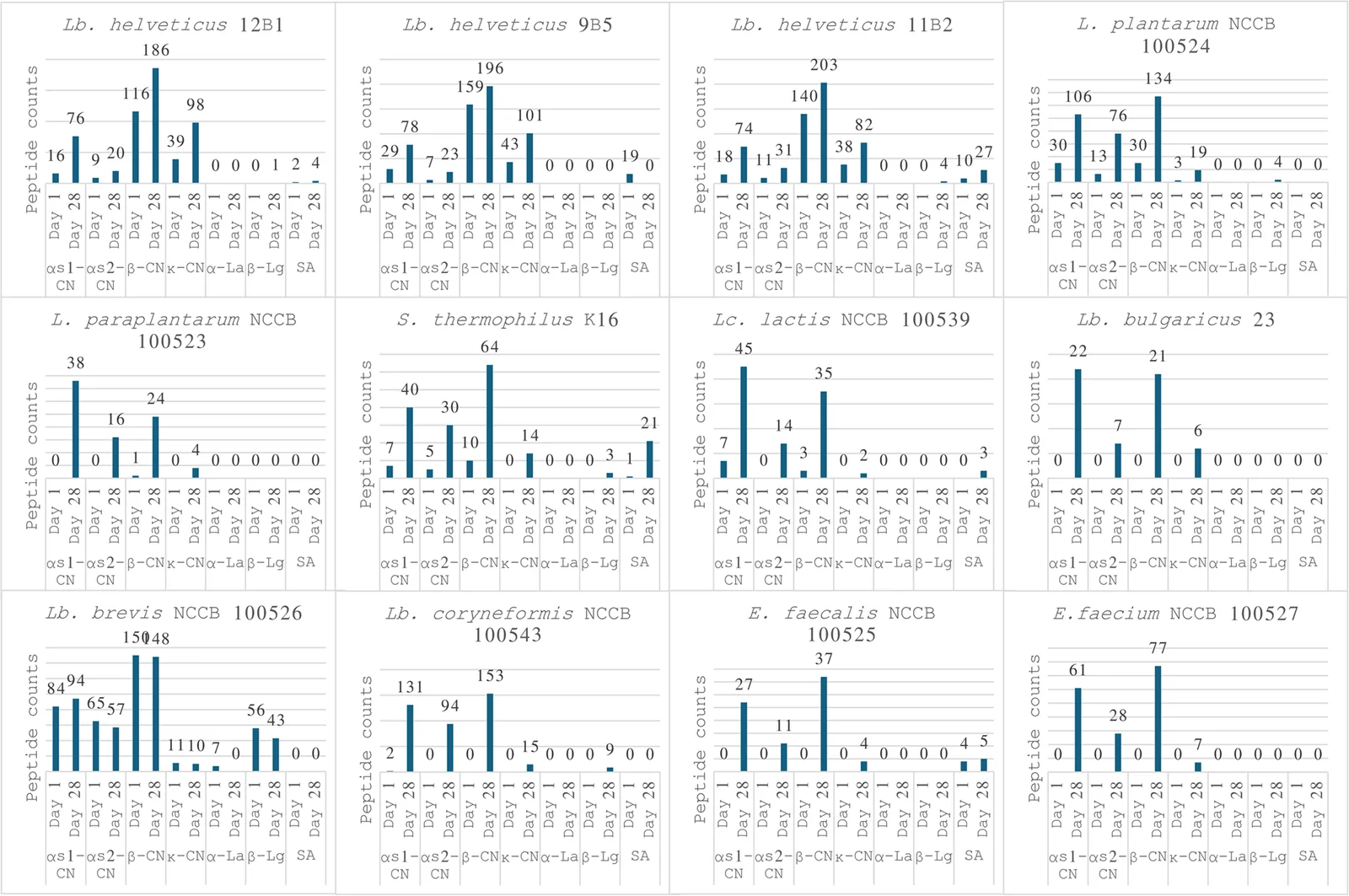
Dynamics of peptide generation from milk proteins by different LAB isolates during the 28 days of storage

The number of peptides (422–511) identified in fermented milk samples produced by *Lb. helveticus* strains in the present study was higher than those reported by Gao, Jiang [25], who identified 181–450 peptides from various *Lb. helveticus* strains isolated from dairy products. The variation in the number of peptides observed among different isolates highlights the importance of strain-specific differences and underscores the distinctive proteolytic characteristics of LAB isolates. The length of the peptides identified for *Lb. helveticus* isolates consisted of 3–29 amino acid sequences, and nonapeptides were predominant, followed by octapeptides and decapeptides. Changes in the number of < 3 kDa peptides according to different milk protein fractions in milk produced with different LAB isolates during 28 days of storage are depicted in Fig. 2. There was a significant increase in the number of peptides produced from all protein fractions by the proteolytic system of *Lb. helveticus* isolates with storage time. Previous studies have also reported increases in peptide content during the storage of fermented dairy products, attributing this to progressive proteolysis over time [26].

*L. plantarum* has been shown to play an important role in the degradation of milk proteins with high proteolytic activity [27]. The proteolytic system of *L. plantarum* consists of various enzymes such as CEPs (such as extracellular serine proteinase-PrtP), endopeptidases, carboxypeptidases, and aminopeptidases [28]. In this study, it was observed that especially carboxypeptidase activity, tripeptidase, CEP and endopeptidase activity were important in the peptides formed in milk produced with *L. plantarum* NCCB 100,524 (Supplementary Table 4). Although the majority of the peptides produced by *L. plantarum* NCCB 100,524 were β-CN derived, this was followed by α_s1_-CN, α_s2_-CN, and Ƙ-CN derived peptides, respectively. In addition, the proteolytic activity of *L. plantarum* was limited to β-lg among whey proteins, generating only four peptides. Chen, Huang [29] reported that CEPs from *L. plantarum* can hydrolyze β-lg. In another study, it was reported that *L. plantarum* AHQ-14 strain reduced milk protein allergenicity by degrading β-lg from milk proteins with a 10 h incubation [30]. Therefore, this hydrolysis ability can also play an important role in reducing food allergenicity. The length of the peptides identified from *L. plantarum* NCCB 100,524 varied between 3 and 21 amino acid sequences, with the most dominant peptides consisting of nona-, deca-, and undeca-peptides (Supplementary Table 4). However, tripeptides were detected more frequently compared to *Lb. helveticus* strains. By day 28 of storage, the number of identified peptides increased approximately fourfold (Fig. 2) compared to the beginning of the storage period, with progressive proteolysis.

There are limited studies in the literature on the proteolytic activity of *L. paraplantarum.* Strahinic, Kojic [31] determined that the prtP proteinase gene identified for *L. plantarum* was not present in *L. paraplantarum*, but *L. paraplantarum* isolates had proteolytic activity on β-CN. Peptides identified in milk fermented with *L. paraplantarum* NCCB 100,523 were mostly derived from α_s1_-CN, followed by β-CN, α_s2_-CN, and Ƙ-CN derived peptides, respectively (Supplementary Table 5). On the other hand, no whey protein-derived peptides were detected, suggesting that the proteolytic enzymes of the *L. paraplantarum* strain were primarily active on caseins. Interestingly, the dominant determination of tripeptides reflects the proteolysis pattern of this strain on milk proteins. Additionally, peptides were identified with *L. paraplantarum* NCCB 100,523 especially on the 28th day of storage, and very few peptides were identified at the beginning of storage (Fig. 2). Therefore, the storage or ripening period of fermented dairy products produced with *L. paraplantarum* may play a critical role in enhancing their functionality through peptide formation. Product applications can thus be developed in this manner.

In the literature, it has been reported that certain *S. thermophilus* strains, which are not typically known for their proteolytic properties, may exhibit such activity depending on their genetic characteristic. In this study, three peptides were identified for *S. thermophilus* 1K4 (Supplementary Table 6), while 176 peptides were identified for *S. thermophilus* K16 (Supplementary Table 7). It was concluded that *S. thermophilus* 1K4 is not proteolytic and cannot play a role in the production of bioactive peptides. Li, Peng [32] identified only 24 peptides in milk fermented with *S. thermophilus* strain. Therefore, for this species with low proteolytic activity, *S. thermophilus* K16 strain stands out in terms of bioactive peptide production from milk proteins. Furthermore, the peptides identified in milk fermented with this strain were β-CN derived and ranged from 3 to 14 amino acids in length. These peptides were detected only on day 28 of storage. *S. thermophilus* K16 appeared to act primarily on β-CN, and subsequently hydrolyzed α_s1_- and α_s2_-CN to generate peptides. This activity appears to result primarily from endopeptidase and tripeptidase activity, with minimal involvement of carboxypeptidase (Supplementary Table 7). Previous studies have reported that these enzymes can be found in some *S. thermophilus* strains. Furthermore, it has been reported that serine proteinases may also contribute to the proteolytic activity of *S. thermophilus*. Serine proteases have been shown to be highly effective in the hydrolysis of β- and α_S_-CN [33]. For *S. thermophilus* K16 strain, tripeptides were found to be predominant in the peptide composition, with identified sequences ranging from 3 to 21 amino acids. A substantial increase in peptide count was observed during the storage period (Fig. 2).

The CEPs (PrtP) of *Lc. lactis* has been reported to play a key role in its proteolytic system and to be effective in the degradation of milk proteins [34]. In this study, the highest level of proteolysis in fermented milk products produced with *Lc. lactis* NCCB 100,539 was observed on α_s1_-CN, followed by β-, α_s2_-, and κ-CN (Supplementary Table 8). Among the whey proteins, only serum albumin derived peptides were detected. The obtained peptide data revealed that the peptides generated by the *Lc. lactis* NCCB 100,539 were primarily the result of carboxypeptidase activity, along with contributions from tripeptidase and endopeptidase activity. Interestingly, a previous study reported that *Lc. lactis* strains exhibited either very low or undetectable carboxypeptidase activity under in vitro conditions [35]. However, in the present study, carboxypeptidase activity appeared to play a prominent role in peptide generation by the *Lc. lactis* NCCB 100,539. This suggests that the growth environment can influence enzymatic activity, and that carboxypeptidase activity of this species may be more pronounced in milk compared to liquid culture media. The identified peptides varied in length from 3 to 27 amino acids, with those consisting of 13–14 residues being the most predominant. Interestingly, the dominant peptides generated by *Lc. lactis* were longer than those produced by the other LAB cultures. The number of observed peptides was significantly influenced by the storage period, with 89 additional peptides identified on day 28 (Fig. 2).

The only CEP of *Lb. bulgaricus* has been identified as PrtB, and its peptide transport systems have been reported to consist of Opp and Dpp, along with aminopeptidases, endopeptidases, dipeptidases, and tripeptidases for the degradation of transported peptides [36]. The peptides generated by *Lb. bulgaricus* 23 revealed the involvement of aminopeptidase, carboxypeptidase, endopeptidase, and tripeptidase activities in the hydrolysis of milk proteins (Supplementary Table 9). In addition, the proteolytic system of *Lb. bulgaricus* 23 appeared to act predominantly on α_s2_- and β-CN, from which most peptides were derived. Although a smaller number of peptides originated from α_s1_- and κ-CN, no peptides derived from whey proteins were detected. Li, Habermann [37] reported that β-casein was highly susceptible to the proteinase enzymes encoded by the *prtB* gene of *Lb. bulgaricus*. Additionally, Laloi, Atlan [38] demonstrated that PrtB from *Lb. bulgaricus* preferentially hydrolyzed α_s1_- and β-CN. A greater number of peptides were identified for *L. bulgaricus* 23 in a milk-based environment compared to earlier studies [37]. However, its peptide production potential was still found to be lower than that of *Lb. helveticus* strains and some other isolates. In the < 3 kDa fraction of fermented milk samples produced with *Lb. bulgaricus* 23, peptides ranged from 3 to 21 amino acids in length, with tripeptides being predominant (Supplementary Table 9). Storage time appeared to be a critical factor for *L. bulgaricus* 23, as the detected peptides were primarily observed on day 28 of storage (Fig. 2).

In the present study, a high number of peptides were generated from milk proteins by *Lb. brevis* NCCB 100,526, which was attributed to its strong proteolytic activity (Supplementary Table 10). Carboxypeptidase activity was found to play a major role in the generation of these peptides, with additional contributions from endopeptidase and tripeptidase activities. Similarly, Bounouala, Roudj [34] also reported that *Lb. brevis* exhibited prominent carboxypeptidase activity against milk proteins. In this study, *Lb. brevis* NCCB 100,526 appeared to exhibit higher proteolytic activity toward β-CN, as the majority of identified peptides were derived from β-CN, followed by peptides originating from α_s1_-, α_s2_-, and κ-CN, respectively. Moreover, peptides derived from β-lg and α-la were also detected among the whey proteins. Notably, α-la derived peptides were observed only in *Lb. brevis* NCCB 100,526 in this study, highlighting the distinctiveness of its proteolytic system. The identified peptides ranged from 3 to 24 amino acids in length, with octapeptides and nonapeptides being predominant (Supplementary Table 10). The impact of storage time on peptide dynamics was found to be important (Fig. 2), as the total number of peptides remained nearly constant, but different peptides were formed during this period.

Among LAB species, *L. coryniformis* has been reported to exhibit high aminopeptidase activity under in vitro conditions [39]. However, there is limited information in the literature regarding its carboxypeptidase activity of *L. coryniformis*. In the current study, *L. coryniformis* NCCB 100,543 demonstrated good potential for generating peptides from milk proteins. According to the peptide data, the peptides were predominantly produced through carboxypeptidase and endopeptidase activity (Supplementary Table 11). β- and α_s1_-CN were found to be particularly susceptible to the proteolytic enzymes of *L. coryniformis* NCCB 100,543, with a high number of peptides derived from these precursor proteins. Additionally, the number of α_s2_-CN derived peptides was also found to be high. In contrast, only a small number of peptides originated from κ-CN and β-lg. This study is the first to comprehensively investigate the proteolytic activity of *L. coryniformis* on milk proteins. In fermented milk produced with *L. coryniformis* NCCB 100,543, peptides ranging from 3 to 21 amino acids were identified, with nonapeptides and decapeptides being the most dominant. While only two peptides were detected at the beginning of storage, a total of 402 peptides were observed by day 28 (Fig. 2). These findings suggest that the storage period following fermentation contributes significantly to the advanced proteolytic activity of the *L. coryniformis* strain.

In the present study, analysis of the peptide sequences obtained from *E. faecalis* NCCB 100,525 (Supplementary Table 12) indicated that carboxypeptidase and endopeptidase activities played a role in the generation of peptides from precursor proteins. These enzymes appeared to act particularly on β- and α_s_-CN, while smaller numbers of peptides derived from κ-CN and serum albumin were also detected. In line with these findings, Tulini, Bíscola [40] also demonstrated that *E. faecalis* FT132 exhibits high proteolytic activity against β- and α_s_-CN. On the other hand, Worsztynowicz, Białas [41] reported that coccolysin and glutamyl endopeptidase enzymes in *E. faecalis* were responsible for the hydrolysis of α-la, β-lg, lactoferrin, and serum albumin, resulting in peptide formation. In milk fermented with *E. faecalis* NCCB 100,525, peptides ranging from 3 to 21 amino acids in length were identified, with those comprising 12 residues being the most predominant (Supplementary Table 12). Additionally, nearly all of the detected peptides were formed on day 28, indicating that storage time plays a significant role in protein hydrolysis by this *E. faecalis* NCCB 100,525 (Fig. 2).

The peptides obtained from *E. faecium* NCCB 100,527 revealed that they were generated primarily through carboxypeptidase and endopeptidase activity (Supplementary Table 13). β- and α_s1_-CN appeared to be more susceptible to the proteolytic enzymes of *E. faecium* NCCB 100,527, with the highest number of peptides derived from β-CN. These were followed by peptides derived from α_s1_-, α_s2_-, and κ-CN, respectively. Veljovic, Fira [42] previously reported that *E. faecium* isolates exhibited intensive hydrolysis of β- and α_s1_-CN. Likewise, Kordesedehi, Taheri-Kafrani [43] found that *E. faecium* isolated from camel milk demonstrated high proteolytic activity against α_s1_-CN. In this study, peptides identified in milk fermented with *E. faecium* NCCB 100,527 ranged from 3 to 26 amino acids in length, with sequences containing 11–13 residues being predominant. Notably, all peptide sequences identified in this study were observed only on day 28 of storage (Fig. 2). Therefore, a storage period of 28 days is suggested to ensure optimal development of peptides.

Overall, among the LAB isolates, storage time was a common factor influencing peptide formation, with a general increase in peptide abundance and progressive proteolysis observed during storage. Despite this, marked strain-dependent differences were evident in the extent of proteolysis, peptide length distribution, and precursor protein susceptibility. Strains with high proteolytic capacity, particularly *Lb. helveticus* and *Lb. brevis*, exhibited the generation of both short- and longer-chain peptides throughout storage. In contrast, isolates such as *L. plantarum* and *L. coryniformis* showed moderate proteolytic behavior, with peptide profiles dominated by short to intermediate-length sequences. *L. paraplantarum*, *Lb. bulgaricus* 23, and *S. thermophilus* K16, produced peptides primarily at later storage stages and were characterized mainly by short-chain peptides, while *S. thermophilus* 1K4 exhibited negligible proteolytic activity. These findings demonstrate that although storage time contributes to a general increase in peptide formation, the qualitative characteristics of peptides are predominantly affected by strain-specific enzymatic properties.

### Prediction and *In Vitro* Validation of Antimicrobial Activity of Peptide Fractions.

Antibacterial properties of < 3 kDa peptide fractions obtained from milk samples fermented with LAB isolates were evaluated in silico and in vitro by the CAMP3 random forest algorithm and MIC test, respectively. In this study, the random forest algorithm was used for AMP prediction, as it has been reported to yield higher accuracy among the CAMP3 models [1]. It was particularly interesting that AMPs came from β-CN derived peptides. Additionally, the highest AMP score was also determined in β-CN derived peptides. LHLPPLLLQ, VLGPVRGPFPIIV and LGPVRGPFPIIV were identified as β-CN derived peptides with high AMP scores (> 0.800). These peptides are cationic and have a hydrophobicity between 65 and 75%. The positive charge of the peptides has been reported to be important in their antibacterial activity by interacting with the negative cell membrane of bacteria [2]. Therefore, the presence of basic amino acids such as lysine and arginine in the peptide sequences is thought to contribute to the higher AMP scores of these β-CN derived peptides. Moreover, it has been reported that the presence of hydrophobic regions within AMPs is important for their interaction with the hydrophobic phospholipid bilayer of bacterial membranes and for their ability to form pores [3]. Other AMP candidates were determined to be mostly peptides derived from α_s1_- and α_s2_-CN. Similarly, despite using different milk types in previous studies, milk AMPs were determined to consist mostly of peptides derived from β-CN [4, 5]. These findings suggest that the hydrolysis of β-CN plays a crucial role in the generation of AMPs, thereby underscoring the importance of lactic cultures exhibiting proteolytic activity toward β-CN. From whey proteins, AMPs were predominantly identified as derivatives of serum albumin, along with some peptides derived from β-lg and α-la. However, AMP scores of whey protein precursor peptides and κ-CN derived peptides were found to be lower.

While the highest number of AMPs was determined in *Lb. helveticus* 11B2 and *Lb. brevis* NCCB 100,526 isolates, the lowest number of AMPs was detected in *Lc. lactis* NCCB 100,539, *Lb. bulgaricus* 23, and *E. faecalis* NCCB 100,525. In addition, no AMPs were detected in *S. thermophilus* 1K4. On the other hand, the highest scoring AMPs were observed in isolates *Lb. helveticus* 12B1, 9B5, 11B2, *L. plantarum* NCCB 100,523, *S. thermophilus* K16, *Lc. lactis* NCCB 100,539, *Lb. brevis* NCCB 100,526, *L. coryniformis* NCCB 100,543, *E. faecalis* NCCB 100,525, and *E. faecium* NCCB 100,527. The β-CN-derived peptide DVENLHLPLPL was shown to downregulate the expression of specific virulence genes in *Salmonella* Typhimurium DT104 in a dose-dependent manner [6]. This peptide was identified in cultures of *Lb. helveticus* 12B1, 9B5, 11B2, *L. plantarum* NCCB 100,524, *S. thermophilus* K16, *Lc. lactis* NCCB 100,539, and *L. coryniformis* NCCB 100,543. Furthermore, the Ƙ-CN-derived peptide MMK was found to exhibit antibacterial activity against *E. coli* PTCC 1399 and *St. aureus* PTCC 1431 [7]. This peptide was observed only in *E. faecium* NCCB 100,527. Future studies are recommended to further investigate the antibacterial activity of high-scoring AMP candidates by isolating them through preparative HPLC or synthesizing them for testing against a range of Gram-positive and Gram-negative bacteria.

The in vitro antibacterial effects of the < 3 kDa WSE fractions were evaluated using the MIC test on days 1 and 28 of storage. The results (Table 2) showed that the samples exhibited antibacterial activity against Gram-negative bacteria such as *S.* Typhimurium and *E. coli*, whereas no inhibition was observed against *St. aureus* and *M. tuberculosis*. It has been reported that peptide hydrophobicity plays a role in the inhibition of Gram-negative bacteria [44]. Therefore, the hydrophobic amino acid sequences identified in the WSEs (Table 1.) may be responsible for the inhibition of these Gram-negative pathogens. Antibacterial peptides are known to bind to the surface of lipopolysaccharides in Gram-negative bacteria [45]. It has been reported that hydrophobic properties are important for the antibacterial component to adhere to this surface due to the hydrophobic structure of the lipid bilayers that form the bacterial membrane [8]. Considering Table 1., it has been suggested that fractions containing peptides derived from β-CN, with high hydrophobicity and elevated AMP scores, exhibited inhibition against Gram-negative bacteria Table 2.

**Table 1:**
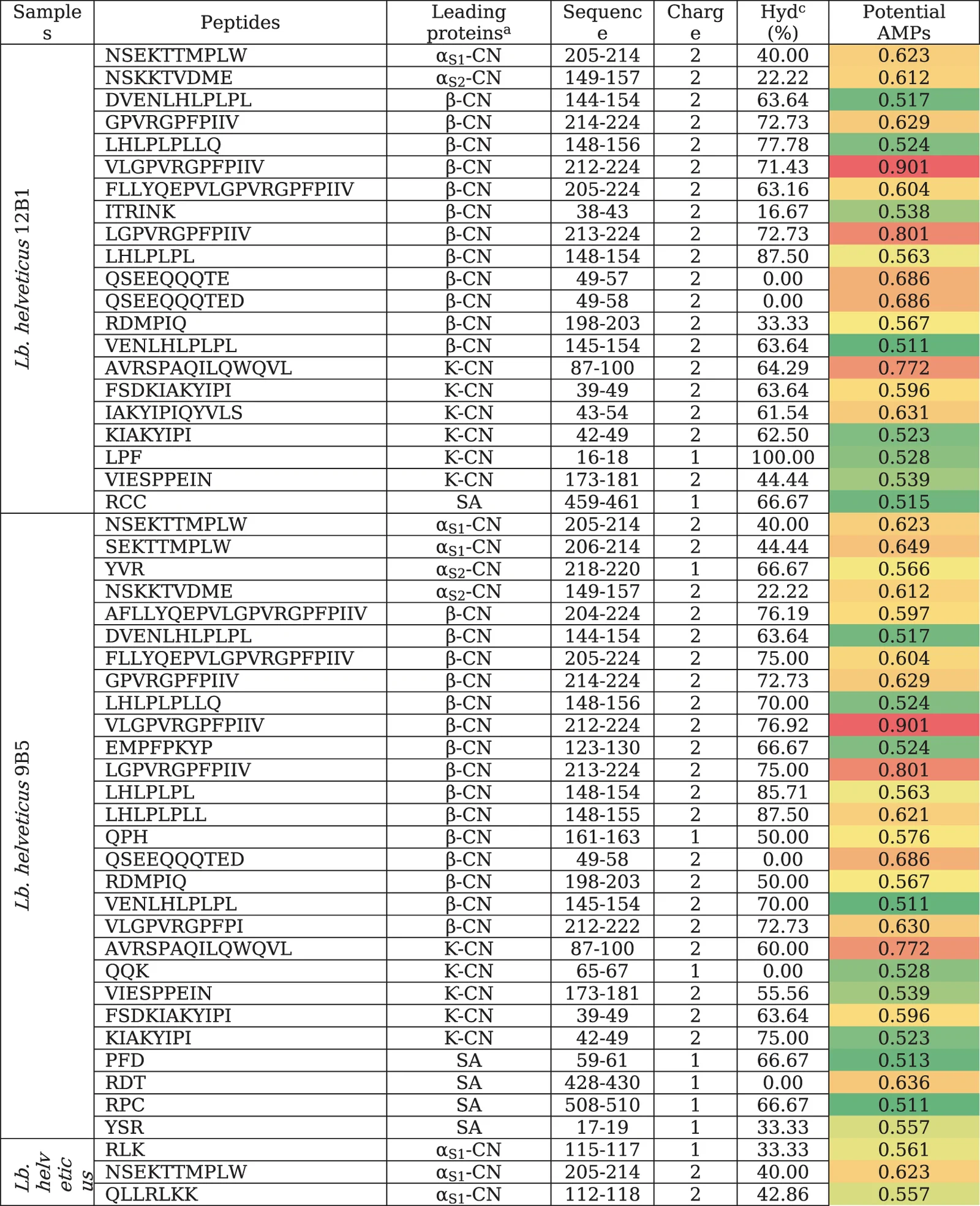

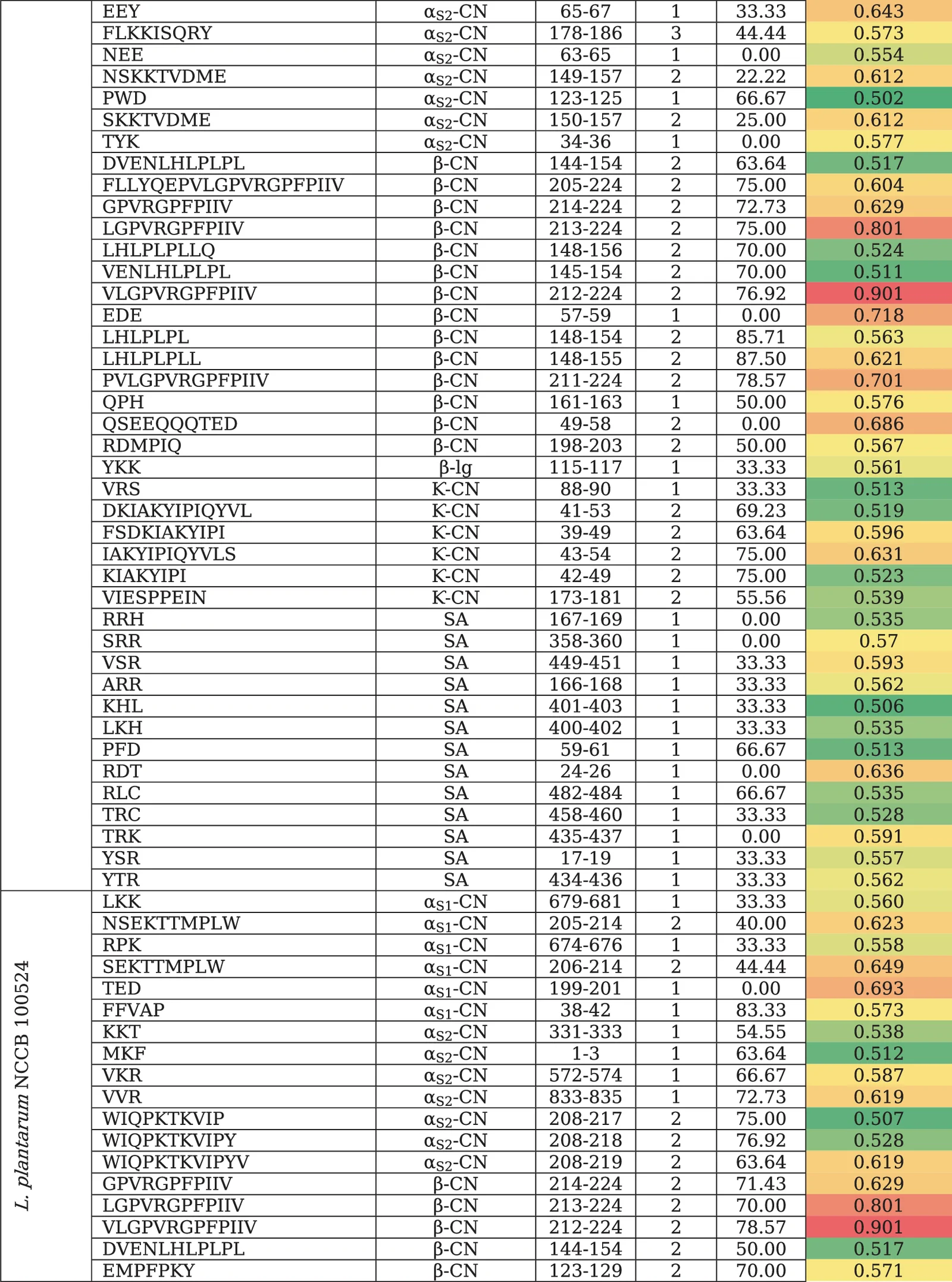

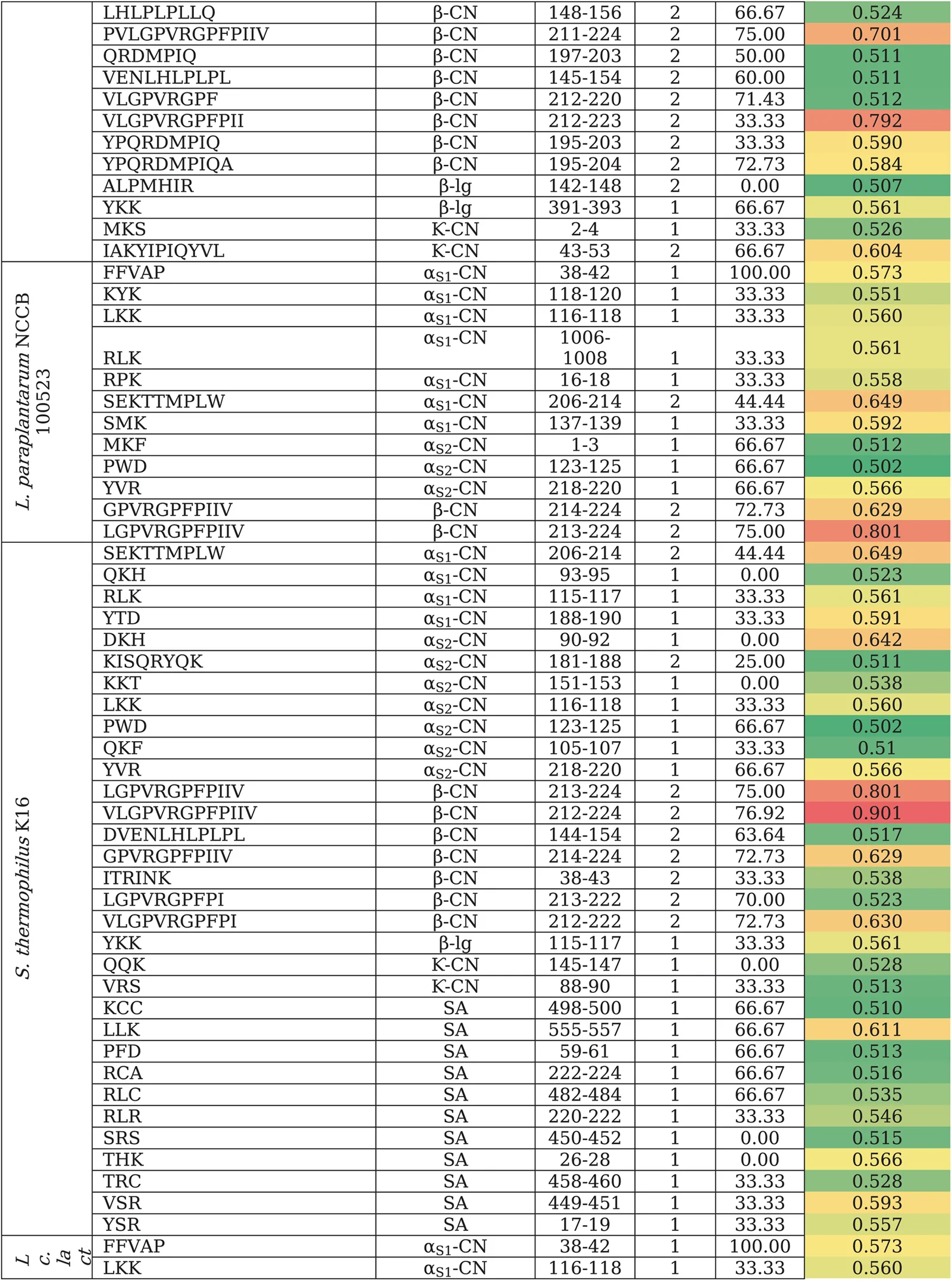

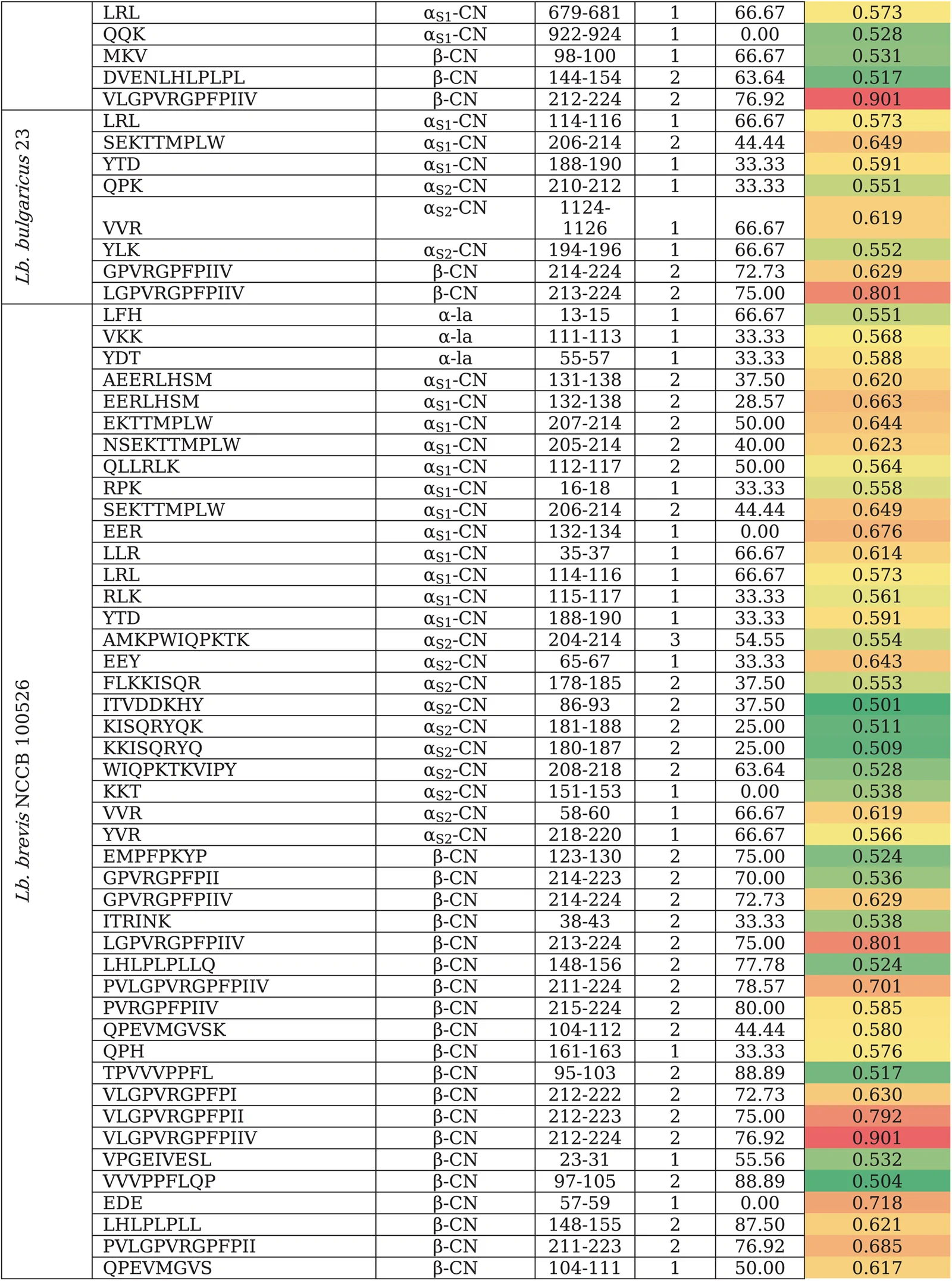

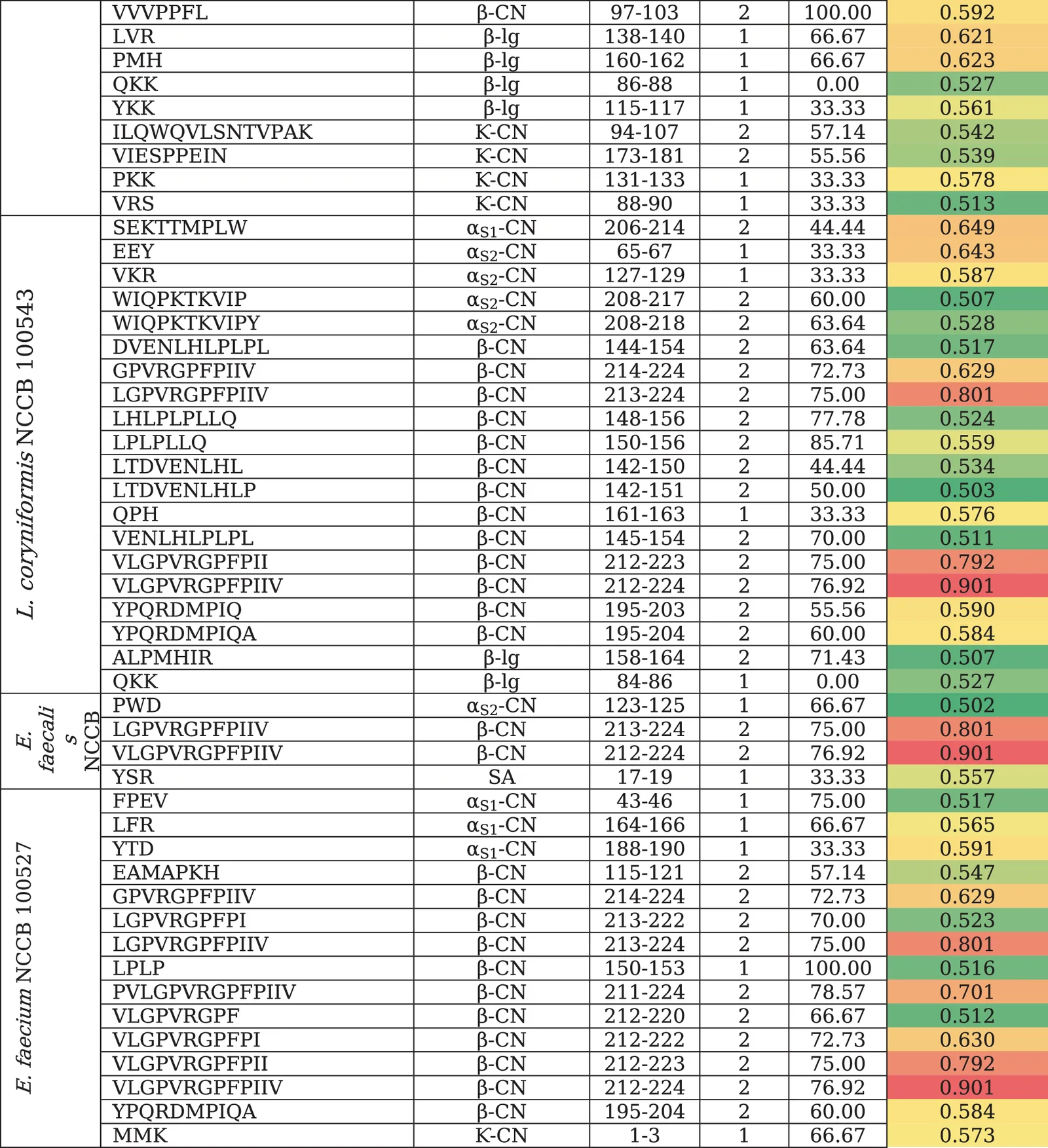
Prediction of antimicrobial peptides in <3 kDa fractions from milk samples fermented with different LAB isolates using the CAMP3 random forest model

^a^*CN* casein, *SA* serum albumin, *lg* lactoglobulin, *la* lactalbumin.

^b^*RT* retention time.

^c^Hydrophobicity.

**Table 2:** In vitro determination of the antibacterial activity of < 3 kDa WSE peptide fractions by MIC assay during storage

LAB sample	Days	MIC value (mg/mL)			
		*S.* Typhimurium	*E. coli*	*S. aureus*	*M. tuberculosis*
*Lb. helveticus* 12B1	1	33.00	66.00	ND	ND
	28	33.00	66.00	ND	ND
*Lb. helveticus* 9B5	1	41.25	ND	ND	ND
	28	24.75	66.00	ND	ND
*Lb. helveticus* 11B2	1	33.00	66.00	ND	ND
	28	33.00	66.00	ND	ND
*L. plantarum* NCCB 100,524	1	33.00	ND	ND	ND
	28	24.75	ND	ND	ND
*L. paraplantarum* NCCB 100,523	1	49.50	ND	ND	ND
	28	49.50	ND	ND	ND
*S. thermophilus* 1K4	1	66.00	ND	ND	ND
	28	41.25	ND	ND	ND
*S. thermophilus* K16	1	66.00	ND	ND	ND
	28	33.00	ND	ND	ND
*Lc. lactis* NCCB 100,539	1	66.00	ND	ND	ND
	28	66.00	ND	ND	ND
*Lb. bulgaricus* 23	1	24.75	ND	ND	ND
	28	33.00	ND	ND	ND
*Lb. brevis* NCCB 100,526	1	49.50	ND	ND	ND
	28	49.50	66.00	ND	ND
*L. coryniformis* NCCB 100,543	1	66.00	ND	ND	ND
	28	49.50	66.00	ND	ND
*E. faecalis* NCCB 100,525	1	49.50	ND	ND	ND
	28	16.50	ND	ND	ND
*E.faecium* NCCB 100,527	1	49.50	ND	ND	ND
	28	16.50	ND	ND	ND

*ND: not detected

The strongest antibacterial activity against *S.* Typhimurium ATCC 13,311 was observed in the samples of *Lb. helveticus* 9B5, *L. plantarum* NCCB 100,524, *E. faecalis* NCCB 100,525, and *E. faecium* NCCB 100,527. Evaluation of the MIC values indicated that antibacterial activity increased over the storage period, likely due to proteolysis. This enhancement can be attributed to the formation of newly generated antibacterial peptides (Supplementary Tables 1–13). Sah, Vasiljevic [19] also reported that the antibacterial activity of yogurt increased by the end of the 28 days due to the accumulation of bioactive peptides. The antibacterial activity against *E. coli* ATCC 25,922 was observed at the same level (66.00 mg/mL) in the samples of *Lb. helveticus* 12B1, *Lb. helveticus* 11B2, *Lb. brevis* NCCB 100,526, and *L. coryniformis* NCCB 100,543. This uniform activity may be attributed to the presence of common β-CN derived peptides of GPVRGPFPIIV, LGPVRGPFPIIV, LHLPLPLLQ, and VLGPVRGPFPIIV in these samples, each exhibiting hydrophobicity levels above 70%, which indicates their potential contribution to the observed antibacterial effect.

### Anticancerogenic Properties of < 3 kDa Peptides Derived from Fermented Milk

#### Selective Cytotoxicity of < 3 kDa Peptide Fractions on Cancer and Normal Cells

**Fig. 3:**
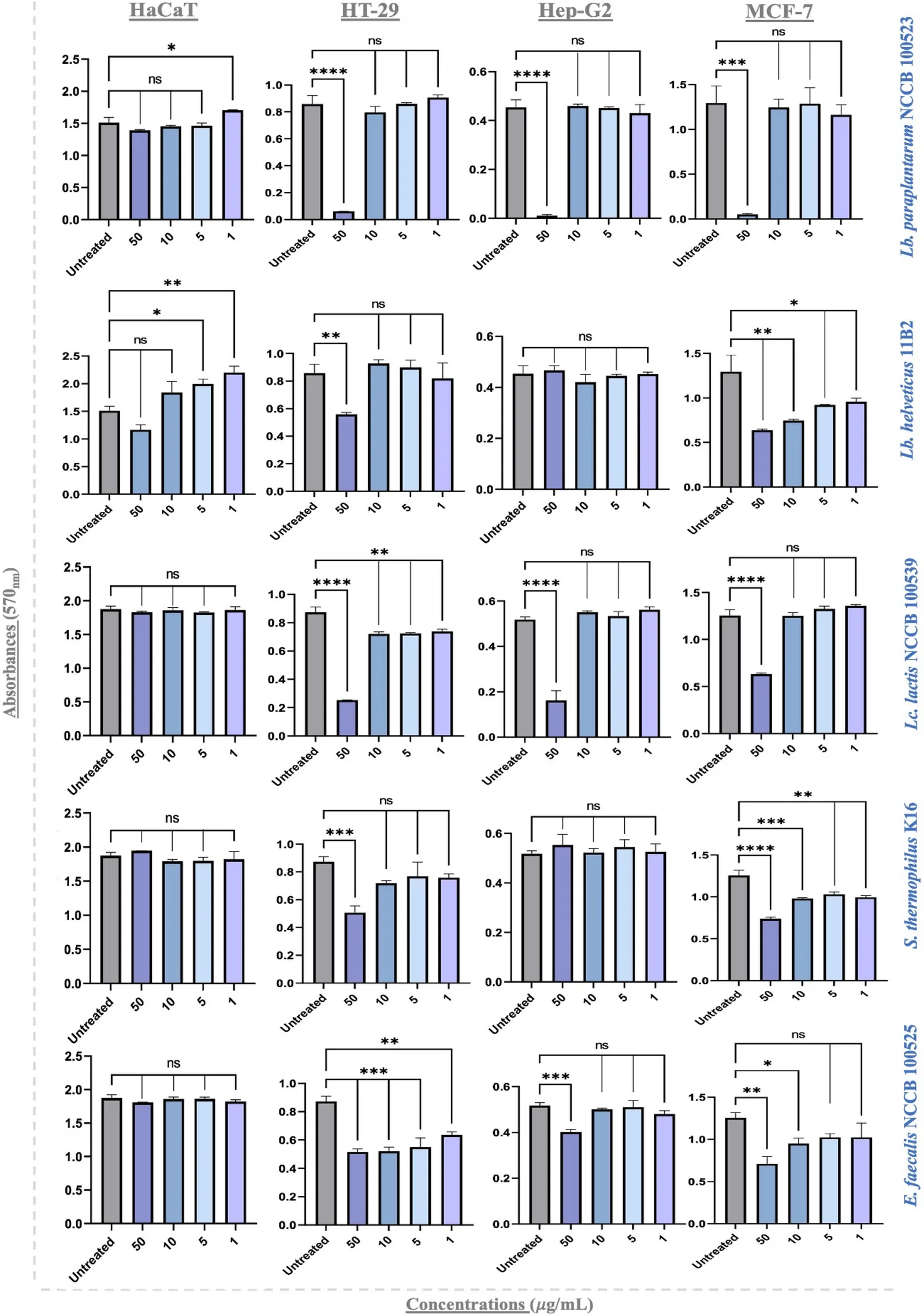
Cytotoxic effects of < 3 kDa peptide fractions obtained on day 1 from milk fermented with different LAB strains on cancer cells. Data are expressed as mean ± SD (*n* = 3). Statistical significance is indicated as follows: * *p* < 0.05, ** *p* < 0.01, *** *p* < 0.001, **** *p* < 0.0001, n.s: non-significant (*p* > 0.05)

Unlike conventional chemotherapy, which often causes toxicity in normal cells and promotes drug resistance, peptides derived from LAB strains are increasingly recognized as promising natural-origin therapeutic agents [46]. In the present study, the cytotoxic effects of < 3 kDa peptide fractions obtained from milk fermented with different LAB isolates were evaluated across multiple cancer cell lines. HaCaT cells were used as a non-cancerous control model to assess selective cytotoxicity [47]. Based on the experimental results, five strains (*L. paraplantarum* NCCB 100523, *Lb. helveticus* 12B2, *Lc. lactis* NCCB 100539, *S. thermophilus* K16, and *E. faecium* NCCB 100525) were identified as promising candidates. The peptide fractions obtained from these strains during the initial storage period significantly reduced cancer cell viability (*p* < 0.05), while no cytotoxic effects were observed in HaCaT cells at any of the tested concentrations (Fig. 3). However, peptide fractions obtained at later storage days exhibited pronounced cytotoxic effects on HaCaT cells, with more than 50% reduction in cell viability observed at all tested concentrations compared to untreated controls (*p* < 0.05, data not shown). Since, a general decline in HaCaT cell viability was observed during storage, peptide fractions obtained on day 1, which did not exhibit cytotoxic effects on HaCaT cells were selected for subsequent analyses. This decline is likely associated with continued proteolytic activity during storage, leading to further peptide degradation and the accumulation of smaller peptides and free amino acids, which may contribute to cytotoxic effects in normal cells [48]. Similar effects have been reported previously, including a reduction in HaCaT cell viability following fermentation with *L. helveticus* NS8, an effect attributed to the presence of low-molecular-weight peptides [49]. Moreover, the inhibitory activities of peptides derived from lactoferrin [50], casein, and β-lactoglobulin [45, 51] further support the notion that cytotoxic effects on normal cells are strain-specific [52].

Interestingly, low concentrations (1 and 5 µg/mL) of < 3 kDa peptide fractions from milk fermented with *L. paraplantarum* NCCB 100,523 and *Lb. helveticus* 12B2 induced a modest but statistically significant increase in HaCaT cell viability compared to untreated controls (*p* < 0.05) (Fig. 3). Similar effects have been reported for the P8 protein isolated from *Lacticaseibacillus rhamnosus*, which exhibits anticancer activity while simultaneously promoting normal cell viability [53]. However, although these findings suggest a potential proliferative response under the tested conditions, further studies are required to elucidate its biological relevance and underlying mechanisms.

#### Antiproliferative Effects on HT-29 Colon Cancer Cells

In HT-29 cells, < 3 kDa peptide fractions obtained on day 1 from milk fermented with *L. paraplantarum* NCCB 100,523 (*p* < 0.0001), *Lb. helveticus* 12B2 (*p* < 0.01), and *S. thermophilus* K16 (*p* < 0.001) showed significant cytotoxic effects at 50 µg/mL, compared to the control. Additionally, peptide fractions derived from *Lc. lactis* NCCB 100,539 and *E. faecium* NCCB 100,525 significantly reduced cell viability across all tested concentrations (*p* < 0.05). Overall, HT-29 cell inhibition increased with peptide concentration, indicating a dose-dependent antiproliferative response. The strongest inhibition (96.2%) was observed with the 50 µg/mL peptide fraction from *L. paraplantarum* NCCB 100,523 (Fig. 3). These findings are in agreement with previous reports demonstrating strong HT-29 growth inhibition at higher concentrations of < 3 kDa peptide fractions derived from dairy products [46].

#### Cell-Specific Cytotoxic Responses of HepG2 Liver Cancer Cells

Peptide fractions derived from *L. paraplantarum* NCCB 100,523 (*p* < 0.0001), *Lc. lactis* NCCB 100,539 (*p* < 0.0001) and *E. faecalis* NCCB 100,525 (*p* < 0.001) strains demonstrated statistically significant cytotoxic effects at 50 µg/mL, with the highest inhibition rate (98.6%) observed for *L. paraplantarum* NCCB 100,523 (Fig. 3). However, < 3 kDa peptide fractions obtained from milk fermented with *Lb. helveticus* 12B2 and *S. thermophilus* K16 did not exhibit significant cytotoxic effects (*p*> 0.05) against HepG2 cells (Fig. 3). This cell-specific response may be attributed to differences in membrane lipid composition [54], rapid hepatic peptide metabolism [55], or the absence of molecular targets required for peptide–cell interactions [56]. Notably, to the best of our knowledge, this study provides the first experimental evidence demonstrating the antiproliferative activity of < 3 kDa peptide fractions derived from LAB-fermented milk against the HepG2 hepatocellular carcinoma cell line.

#### Cytotoxic Activity on MCF-7 Breast Cancer Cells

In the case of MCF-7 cells, all tested concentrations of < 3 kDa peptide fractions obtained on day 1 from milk fermented with *Lb. helveticus* 12B2 and *S. thermophilus* K16 induced dose-dependent cytotoxic effects (*p* < 0.05) in MCF-7 cells (Fig. 3). These results align with previous studies reporting dose-dependent antiproliferative activity of fermentation-derived peptides against breast cancer cells [49, 52, 53]. Furthermore, significant cytotoxic activity was observed at 50 µg/mL for peptide fractions derived from *L. paraplantarum* NCCB 100,523 (*p* < 0.001) and *Lc. lactis* NCCB 100,539 (*p* < 0.0001), while peptide fractions from *E. faecium* NCCB 100,525 exhibited selective anticancer effects at 10 µg/mL (*p* < 0.05) and 50 µg/mL (*p* < 0.01). Consistent with other cell lines, the highest inhibition rate (97.1%) was recorded for *L. paraplantarum* NCCB 100,523 at 50 µg/mL (Fig. 3). From a mechanistic point of view, the presence of the β-casein-derived peptide YPFP in milk fermented with *L. paraplantarum* NCCB 100,523 (Supplementary Table 5), previously shown to suppress breast cancer cell growth via opioid and somatostatin receptor-mediated mechanisms [51], further supports the observed antiproliferative effects.

#### Implications for Functional Dairy-Based Anticancer Agents

From both biological and safety perspective, the identification of < 3 kDa peptide fractions that selectively reduced cancer cell viability while preserving, and in some cases enhancing, HaCaT cell viability highlights their potential as safe bioactive compounds for functional or therapeutic applications. Consistent with this favorable safety profile, the present findings demonstrate that < 3 kDa peptide fractions derived from LAB-fermented milk exhibit pronounced anticancer activities that are both strain- and cell line–specific, and are strongly influenced by peptide composition and storage time.

Across the tested cancer cell lines, cytotoxic effects were generally minimal at lower concentrations (1 and 5 µg/mL) and progressively incresed at higher concentrations (10 and 50 µg/mL), with the most pronounced inhibitory effects consistently observed at 50 µg/mL. However, the magnitude of response varied depending on the bacterial strain and cancer cell type. This variability is likely attributable to intrinsic cell line–specific characteristics, including differences in membrane lipid composition, surface charge, metabolic activity, and receptor expression profiles, which may collectively influence peptide–cell interactions, uptake efficiency, and downstream intracellular signaling pathways [46, 48]. This mechanism provides a general explanation for the selective cytotoxic effects observed throughout this study.

Accordingly, the strain-dependent nature of the observed cytotoxic effects underscores that the anticancer activity of LAB-derived low-molecular-weight peptides is governed by a complex interplay between peptide features and the biological properties of the target cancer cells. In this context, *L. paraplantarum* NCCB 100,523 emerged as the most promising strain, consistently exhibiting the highest and most selective cytotoxic effects against HT-29, HepG2, and MCF-7 cancer cell lines, while preserving the viability of non-cancerous HaCaT cells, particularly when peptide fractions were obtained on the first day of storage. From a molecular perspective, the anticancer activities observed in this study may be partially associated with various peptides identified in the < 3 kDa fractions. Several detected peptides originated from milk proteins such as β-casein and β-lactoglobulin and contained amino acid residues including tyrosine, phenylalanine, proline, and hydrophobic residues that have previously been linked to antiproliferative and membrane-active properties. Notably, β-casein–derived peptides such as YPFP and YQEPVLGPVRGPFPIIV, present in the peptide profiles of strains exhibiting strong anticancer activity, have been reported to exert antiproliferative effects in various cancer cell models [19]. Although direct structure–activity relationships cannot be conclusively established within the scope of the present study, the presence of these bioactive sequence motifs provides biological feasibility for the observed strain-dependent cytotoxic effects. Collectively, these findings emphasize the critical importance of strain selection in generating bioactive low-molecular-weight peptides with targeted and selective anticancer potential.

### Immunomodulatory Effects of < 3 kDa Peptide Fractions in Macrophage Cells

**Fig. 4:**
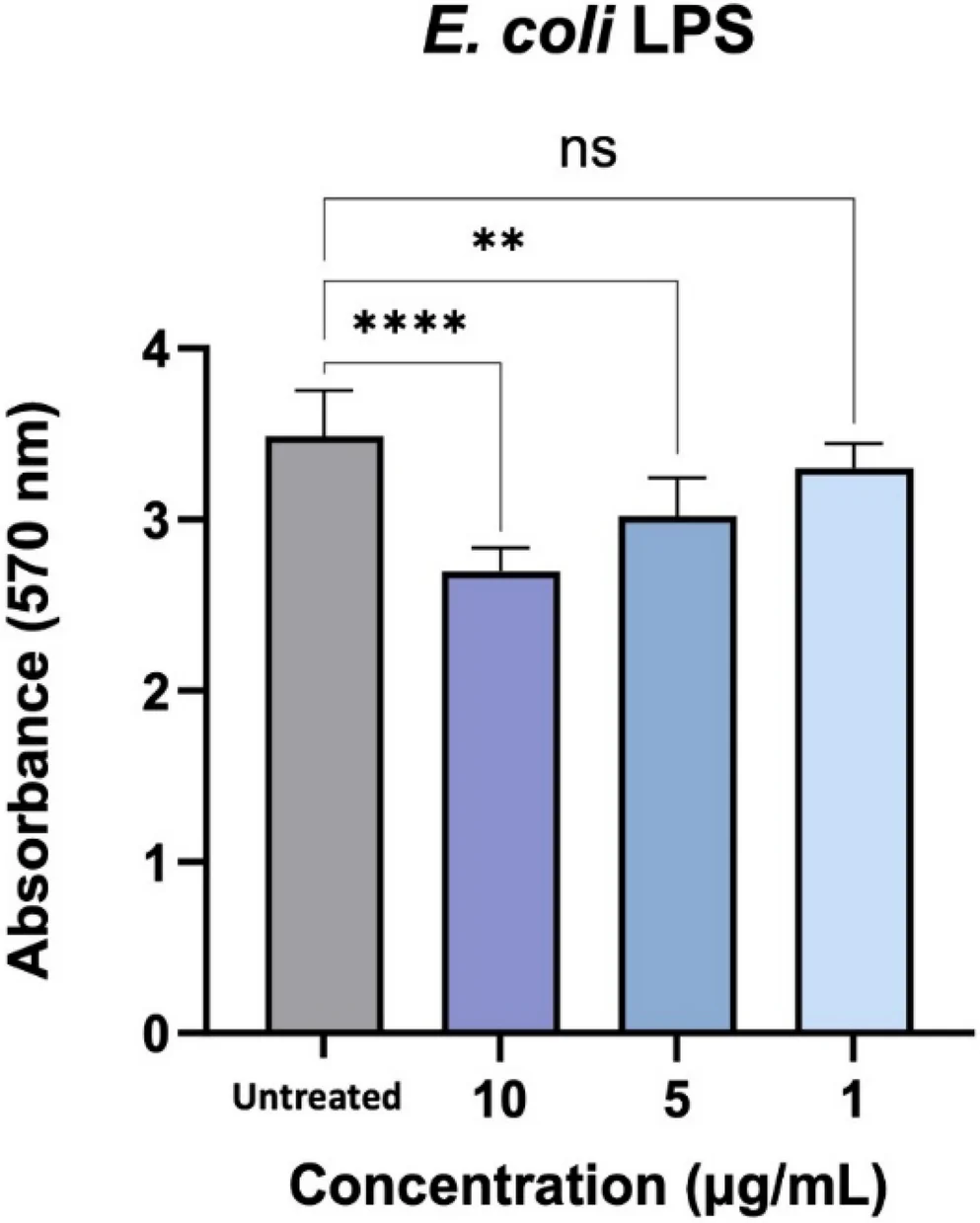
Determination of the highest non-cytotoxic concentration of LPS using the MTT assay. Data are expressed as mean ± SD (*n* = 3). Statistical significance is indicated as follows: ** *p* < 0.01, *** *p* < 0.001, n.s: non-significant (*p* > 0.05)

**Fig. 5:**
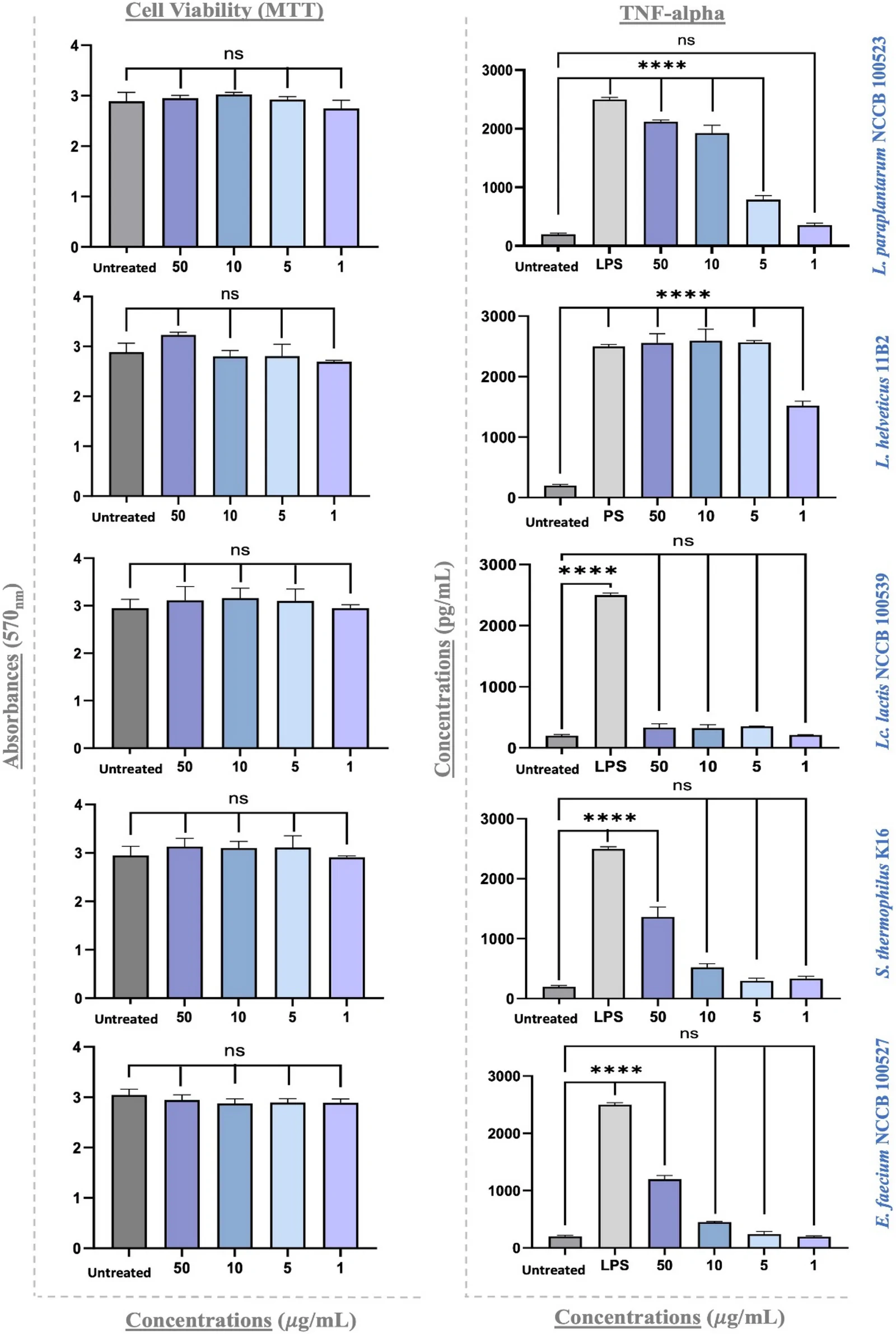
Cytotoxic effects of < 3 kDa peptide fractions obtained on day 1 from milk fermented with various LAB strains on RAW 264.7 macrophages, along with TNF-α levels in RAW 264.7 cells treated with LPS and different concentrations of these peptide fractions, are presented. Data are expressed as mean ± SD (*n* = 3). Statistical significance is indicated as follows: **** *p* < 0.0001; n.s.: not significant (*p* > 0.05)

Macrophages play a crucial role in the early detection of pathogens during infection, their elimination through phagocytosis, and the clearance of dead cells, tumors, foreign substances, and waste from the body [57]. Upon activation, macrophages secrete cytokines such as TNF-α, IL-6, and IL-12, which promote inflammation and stimulate adaptive immune responses [58]. Therefore, TNF-α, IL-6, and IL-12 levels are considered important indicators of immunomodulatory activity. In this study, the levels of IL-12, TNF-α, and IL-6 cytokines were determined after 24 h of incubation at 37 °C with varying concentrations (1–50 µg protein/mL) of peptide fractions. Their effect was further compared using LPS-induced macrophages. To determine a non-cytotoxic concentration of LPS for RAW 264.7, an MTT assay was performed. The results showed that the lowest concentration of LPS (1 µg/mL) did not significantly affect the viability of RAW 264.7 cells and therefore selected for subsequent experiments (Fig. 4). Similarly, all tested concentrations of the < 3 kDa peptide fractions showed no cytotoxic effects on macrophage cell viability and thus included in the study (Fig. 5). Based on the data obtained from our study, signals were detected in the standard ELISA samples for IL-2 and IL-6 production by macrophages. However, although LPS enhanced IL-2 and IL-6 production, it was found that peptides from all LAB strains did not induce any signal for IL-12 and IL-6. On the other hand, literature reports indicate that the intact cell wall structure of LAB strains plays a significant role in their ability to induce IL-6 and IL-12, and that *Lactobacillus* strains with a rigid cell wall resistant to intracellular digestion effectively stimulate macrophages to induce cytokines [59]. Accordingly, it was concluded that peptides derived from LAB strains did not stimulate IL-12 and IL-6 synthesis in macrophages.

TNF-α, as a key regulator of the inflammatory response, is involved in various activities such as the induction of apoptosis and initiation of the inflammatory response [60]. The TNF-α levels produced by macrophage cells following stimulation with varying concentrations (5–50 µg protein/mL) of peptide fractions derived from selected LAB strains are presented in Fig. 5. According to the data obtained, all concentrations of the < 3 kDa peptide fractions derived on day 1 from milk fermented with *Lb. helveticus* 11B2 significantly induced TNF-α production in macrophages compared to the control (*p* < 0.0001), similar to the effect observed with LPS. Likewise, all concentrations of the peptide fractions from *L. paraplantarum* NCCB 100,523 except at 1 µg/mL, significantly increased TNF-α levels compared to the control (*p* < 0.0001). In contrast, none of the tested concentrations of < 3 kDa peptide fractions from *Lc. lactis* NCCB 100,539 led to a statistically significant elevation in TNF-α production. However, the 50 µg/mL dose of the < 3 kDa peptide fractions obtained from *S. thermophilus* K16 and *E. faecalis* NCCB 100,525 on day 1 significantly enhanced TNF-α levels in macrophages compared to the control (*p* < 0.0001). Notably, since the immune stimulation induced by these peptides did not exceed the cytokine levels elicited by LPS, it suggests that these peptides likely do not trigger an excessive inflammatory response but instead exhibit immunostimulatory effects capable of modulating immune activity. The ability of LAB strains to generate immunomodulatory peptides from bovine β-CN has been reported in earlier studies [61]. In particular, *Lb. helveticus* strains have been considered prominent in this regard. Peptides formed in milk fermented with *Lb. helveticus* R389 have been reported to influence cytokine levels (IL-2, IL-6, TNF-α) in the lamina propria located beneath the intestinal mucosa [62]. Furthermore, < 1.0 kDa peptide fractions derived from fermented milk produced *with Lb. delbrueckii* subsp. *bulgaricus* LB340 were found to exhibit strong immunomodulatory activity by enhancing lymphocyte proliferation [63]. Similarly, yogurt fermented with *Lb. brevis* B7 has been shown to increase the gene expression of TNF-α [64]. Consequently, these findings indicate that peptides generated in milk fermented with LAB isolates play a crucial role in immune defense. The fermentation of milk with LAB can enhance immune responses and contribute functional benefits.

On day 1 of storage, peptide sequences derived from β-CN such as HQPHQPLPPTVMFPPQ [65], KVLPVPQ [65], LYQEPVLGPVRGPFPIIV [66], YQEPVLGPVRGPFPIIV [67] were detected in fermented milk produced with *Lb. helveticus* 11B2 (Supplementary Table 3), all of which have been previously reported to possess various immunomodulatory activities. HQPHQPLPPTVMFPPQ and KVLPVPQ have been shown to stimulate the production of regulatory cytokine IL-10 by monocytes [65]. LYQEPVLGPVRGPFPIIV has been reported to provide significant proliferative responses in both lymph node and spleen cells [66], while YQEPVLGPVRGPFPIIV has been found to enhance macrophage phagocytic activity by inducing TNF-α expression [67]. Interestingly, the peptide YQEPVLGPVRGPFPIIV was also identified in *L. paraplantarum* NCCB 100,523, while LYQEPVLGPVRGPFPIIV and YQEPVLGPVRGPFPIIV were also detected in *S. thermophilus* K16. Hence, these peptides, commonly found in all three LAB isolates, may play a role in mitogenic activity against TNF-α cytokine.

## Conclusion

This work presented a comprehensive peptidomic profiling of < 3 kDa peptide fractions from milk fermented with different LAB strains, with a focus on their biofunctional potential via integrated in silico and in vitro studies. Among the 13 strains studied, *Lb. helveticus* 12B1, 9B5, 11B2, *L. plantarum* NCCB 100,524, *Lb. brevis* NCCB 100,526, and *L. coryniformis* NCCB 100,543 showed higher proteolytic activity. The peptides were predominantly derived from β-CN, followed by those from α_s1_-CN, α_s2_-CN, and Ƙ-CN, respectively. Random Forest predicted AMP models identified β-CN derived peptides, particularly VLGPVRGPFPIIV and LGPVRGPFPIIV, as the major peptides contributing to antibacterial activity. In the in vitro antibacterial activity assay, inhibition was observed only against the Gram-negative bacteria *E. coli* and *S.* Typhimurium. The highest inhibitory effect against S. Typhimurium was recorded for *E. faecalis* NCCB 100,525 and *E. faecium* NCCB 100,527, with a MIC value of 16.50 mg/mL, and for *Lb. helveticus* 9B5, *L. plantarum* NCCB 100,524, and *Lb. bulgaricus* 23, with a MIC value of 24.75 mg/mL. For *E. coli*, inhibition was observed only at a MIC value of 66.00 mg/mL in *Lb. helveticus* 12B1, *Lb. helveticus* 9B5, *Lb. helveticus* 11B2, *Lb. brevis* NCCB 100,526, and *L. coryniformis* NCCB 100,543. Remarkably, anticancer assays established dose-dependent HT-29, HepG2, and MCF-7 cell line inhibition with the effects most pronounced at 50 µg/mL. In addition, < 3 kDa peptide fractions of *L. paraplantarum* NCCB 100,523 and *Lb. helveticus* 11B2 were capable of strongly inducing TNF-α production in RAW 264.7 macrophages. Storage time was found to play a significant role in influencing peptide content and bioactivity. Therefore, it is advisable that fermented milk products produced with these cultures be consumed no earlier than the second week of storage to ensure optimal functional properties. This study provides the first strain-level integrated evaluation linking < 3 kDa milk-derived peptide profiles with in silico antimicrobial prediction and in vitro antibacterial, anticancer, and immunomodulatory activities. The results demonstrate the potential of selected LAB strains as targeted peptide producers for the development of innovative dairy products, next-generation functional foods, and peptide-based bioactive ingredients. Although this study reveals strain-dependent differences in peptide bioactivity, it is limited to in vitro experiments and qualitative peptidomic analysis. Future studies should focus on peptide purification, quantitative structure–activity evaluation, and in vivo validation, as well as testing across different dairy matrices and processing conditions, to support the development of safe and effective peptide-based functional foods.

## Supplementary Information

Below is the link to the electronic supplementary material.

Supplementary File 1 (DOCX 833 KB)

## Data Availability

The data generated in this study are available from the corresponding author upon request.
