# Supplementary material for: Peptidomic Characterization of < 3 kDa Peptides from Milk Fermented by Lactic Acid Bacteria: Sequence-Based Antimicrobial Prediction and In Vitro Immuno-Oncological Evaluation
Source: Probiotics Antimicrob Proteins. 2026 Mar 23;18(6):7778–800. doi: 10.1007/s12602-026-10958-3 (PMC13518473; doi:10.1007/s12602-026-10958-3)
Supplement: Supplementary file 1 [file 12602_2026_10958_MOESM1_ESM.docx]

**Supplementary Table 1.** Milk peptides identified throughout the 28 days of storage period using the *Lb. helveticus* 12B1*.*

|  | **Sequence** | **Length** | **Leading proteins** | **Charge** |  | **m/z** | **Mass** | **Retention time** | **Sequences** | **Day 1** | **Day 28** | **Incidence** |
| --- | --- | --- | --- | --- | --- | --- | --- | --- | --- | --- | --- | --- |
| **1** | APSFSDIPNPIGSENSEKTTMP | 22 | Alpha S1 casein | 2 |  | 1160.04 | 2318.07 | 44.60 | 191-212 | + |  | 1 |
| **2** | APSFSDIPNPIGSENSEKTTMPLW | 24 | Alpha S1 casein | 2 |  | 1309.63 | 2617.24 | 48.79 | 191-214 | + | + | 2 |
| **3** | DAPSFSDIPNPIGSENSEKTTMPLW | 25 | Alpha S1 casein | 2 |  | 1367.14 | 2732.26 | 50.15 | 190-214 | + |  | 1 |
| **4** | DIPNPIGSENSEKTTMPLW | 19 | Alpha S1 casein | 2 |  | 1065.01 | 2128.01 | 48.05 | 196-214 | + | + | 2 |
| **5** | EVLNENLLR | 9 | Alpha S1 casein | 2 |  | 550.31 | 1098.60 | 41.98 | 29-37 | + | + | 2 |
| **6** | EVLNENLLRF | 10 | Alpha S1 casein | 2 |  | 623.84 | 1245.67 | 46.45 | 29-38 | + | + | 2 |
| **7** | FSDIPNPIGSENSEKTTMPLW | 21 | Alpha S1 casein | 2 |  | 1182.06 | 2362.12 | 47.84 | 194-214 | + | + | 2 |
| **8** | FYP | 3 | Alpha S1 casein | 1 |  | 426.20 | 425.20 | 36.18 | 160-162 | + |  | 1 |
| **9** | IPNPIGSENSEKTTMPLW | 18 | Alpha S1 casein | 2 |  | 1007.50 | 2012.99 | 46.15 | 197-214 | + | + | 2 |
| **10** | NENLLRF | 7 | Alpha S1 casein | 2 |  | 453.25 | 904.48 | 43.61 | 32-38 | + | + | 2 |
| **11** | QAMEDIKQMEAE | 12 | Alpha S1 casein | 2 |  | 711.82 | 1421.62 | 37.64 | 67-78 | + | + | 2 |
| **12** | QKEPMIGVNQEL | 12 | Alpha S1 casein | 2 |  | 693.36 | 1384.70 | 41.69 | 146-157 | + | + | 2 |
| **13** | SDIPNPIGSENSEKTTMPLW | 20 | Alpha S1 casein | 2 |  | 1108.53 | 2215.05 | 46.74 | 195-214 | + |  | 1 |
| **14** | SFSDIPNPIGSENSEKTTMPLW | 22 | Alpha S1 casein | 2 |  | 1225.58 | 2449.15 | 48.38 | 193-214 | + | + | 2 |
| **15** | SKDIGSESTEDQAMEDIK | 18 | Alpha S1 casein | 2 |  | 991.95 | 1981.88 | 34.16 | 56-73 | + |  | 1 |
| **16** | SMKEGIHAQ | 9 | Alpha S1 casein | 2 |  | 500.75 | 999.48 | 17.67 | 137-145 | + | + | 2 |
| **17** | APSFSDIPNPIGSENSEK | 18 | Alpha S1 casein | 2 |  | 944.95 | 1887.89 | 43.64 | 191-208 |  |  | 1 |
| **18** | AQQKEPMIGVNQEL | 14 | Alpha S1 casein | 2 |  | 792.91 | 1583.80 | 41.73 | 144-157 |  | + | 1 |
| **19** | DIKQMEAE | 8 | Alpha S1 casein | 2 |  | 482.23 | 962.44 | 29.34 | 71-78 |  | + | 1 |
| **20** | DIPNPIGSE | 9 | Alpha S1 casein | 1 |  | 941.46 | 940.45 | 43.80 | 196-204 |  | + | 1 |
| **21** | DIPNPIGSENSEK | 13 | Alpha S1 casein | 2 |  | 700.34 | 1398.66 | 38.52 | 196-208 |  | + | 1 |
| **22** | DQAMEDIKQM | 10 | Alpha S1 casein | 2 |  | 604.77 | 1207.52 | 38.92 | 66-75 |  | + | 1 |
| **23** | DQAMEDIKQME | 11 | Alpha S1 casein | 2 |  | 669.29 | 1336.56 | 40.74 | 66-76 |  | + | 1 |
| **24** | DQAMEDIKQMEAE | 13 | Alpha S1 casein | 2 |  | 769.33 | 1536.64 | 42.20 | 66-78 |  | + | 1 |
| **25** | DVPSERYL | 8 | Alpha S1 casein | 2 |  | 489.75 | 977.48 | 38.98 | 100-107 |  | + | 1 |
| **26** | DVPSERYLG | 9 | Alpha S1 casein | 2 |  | 518.26 | 1034.50 | 36.24 | 100-108 |  | + | 1 |
| **27** | DVPSERYLGYLE | 12 | Alpha S1 casein | 2 |  | 720.85 | 1439.69 | 44.97 | 100-111 |  | + | 1 |
| **28** | DVPSERYLGYLEQL | 14 | Alpha S1 casein | 2 |  | 841.43 | 1680.84 | 49.05 | 100-113 |  | + | 1 |
| **29** | EDIKQME | 7 | Alpha S1 casein | 2 |  | 446.71 | 891.40 | 26.12 | 70-76 |  | + | 1 |
| **30** | EKVNELSKDIGSE | 13 | Alpha S1 casein | 2 |  | 724.37 | 1446.72 | 30.88 | 50-62 |  | + | 1 |
| **31** | ENLLRF | 6 | Alpha S1 casein | 2 |  | 396.22 | 790.43 | 43.86 | 33-38 |  | + | 1 |
| **32** | ENLLRFFVAPFPE | 13 | Alpha S1 casein | 2 |  | 789.92 | 1577.82 | 52.53 | 33-45 |  | + | 1 |
| **33** | EQLLRLK | 7 | Alpha S1 casein | 2 |  | 450.29 | 898.56 | 33.10 | 111-117 |  | + | 1 |
| **34** | ERYLGYLEQL | 10 | Alpha S1 casein | 2 |  | 642.34 | 1282.66 | 45.36 | 104-113 |  | + | 1 |
| **35** | EVLNENLLRFF | 11 | Alpha S1 casein | 2 |  | 697.38 | 1392.74 | 50.70 | 29-39 |  | + | 1 |
| **36** | FVAPFPEVFGK | 11 | Alpha S1 casein | 2 |  | 619.33 | 1236.65 | 47.48 | 39-49 |  | + | 1 |
| **37** | GKEKVNEL | 8 | Alpha S1 casein | 2 |  | 458.76 | 915.50 | 18.58 | 48-55 |  | + | 1 |
| **38** | GKEKVNELS | 9 | Alpha S1 casein | 2 |  | 502.27 | 1002.53 | 17.16 | 48-56 |  | + | 1 |
| **39** | GLPQEVLNEN | 10 | Alpha S1 casein | 2 |  | 556.78 | 1111.55 | 43.00 | 25-34 |  | + | 1 |
| **40** | GLPQEVLNENLL | 12 | Alpha S1 casein | 2 |  | 669.87 | 1337.72 | 49.27 | 25-36 |  | + | 1 |
| **41** | GLPQEVLNENLLRF | 14 | Alpha S1 casein | 2 |  | 821.45 | 1640.89 | 49.21 | 25-38 |  | + | 1 |
| **42** | GSENSEKTTMPLW | 13 | Alpha S1 casein | 2 |  | 740.34 | 1478.67 | 43.92 | 202-214 |  | + | 1 |
| **43** | GTQYTDAPSFS | 11 | Alpha S1 casein | 2 |  | 587.26 | 1172.50 | 40.62 | 185-195 |  | + | 1 |
| **44** | GYLEQLLRLK | 10 | Alpha S1 casein | 2 |  | 616.87 | 1231.73 | 45.69 | 108-117 |  | + | 1 |
| **45** | GYLEQLLRLKK | 11 | Alpha S1 casein | 3 |  | 454.28 | 1359.82 | 43.51 | 108-118 |  | + | 1 |
| **46** | HIQKEDVPS | 9 | Alpha S1 casein | 2 |  | 526.77 | 1051.53 | 18.80 | 95-103 |  | + | 1 |
| **47** | HIQKEDVPSER | 11 | Alpha S1 casein | 3 |  | 446.57 | 1336.67 | 18.00 | 95-105 |  | + | 1 |
| **48** | IKQMEAE | 7 | Alpha S1 casein | 2 |  | 424.71 | 847.41 | 20.13 | 72-78 |  | + | 1 |
| **49** | IVPNSAEERLH | 11 | Alpha S1 casein | 2 |  | 632.84 | 1263.66 | 26.67 | 126-136 |  | + | 1 |
| **50** | KEDVPSER | 8 | Alpha S1 casein | 2 |  | 480.24 | 958.47 | 15.93 | 98-105 |  | + | 1 |
| **51** | KHQGLPQE | 8 | Alpha S1 casein | 2 |  | 468.75 | 935.48 | 17.70 | 22-29 |  | + | 1 |
| **52** | KHQGLPQEVL | 10 | Alpha S1 casein | 2 |  | 574.82 | 1147.63 | 33.95 | 22-31 |  | + | 1 |
| **53** | KYKVPQLE | 8 | Alpha S1 casein | 2 |  | 502.79 | 1003.57 | 31.27 | 118-125 |  | + | 1 |
| **54** | LLRFFVAPFPE | 11 | Alpha S1 casein | 2 |  | 668.38 | 1334.74 | 49.84 | 35-45 |  | + | 1 |
| **55** | NENLLR | 6 | Alpha S1 casein | 2 |  | 379.71 | 757.41 | 27.53 | 32-37 |  | + | 1 |
| **56** | NENLLRFF | 8 | Alpha S1 casein | 2 |  | 526.78 | 1051.55 | 48.02 | 32-39 |  | + | 1 |
| **57** | NENLLRFFVAPFPE | 14 | Alpha S1 casein | 2 |  | 846.94 | 1691.87 | 52.22 | 32-45 |  | + | 1 |
| **58** | NPIGSENSEKTTMPLW | 16 | Alpha S1 casein | 2 |  | 902.43 | 1802.85 | 44.71 | 199-214 |  | + | 1 |
| **59** | NSEKTTMPLW | 10 | Alpha S1 casein | 2 |  | 603.79 | 1205.58 | 43.80 | 205-214 |  | + | 1 |
| **60** | QAMEDIKQM | 9 | Alpha S1 casein | 2 |  | 547.25 | 1092.49 | 33.70 | 67-75 |  | + | 1 |
| **61** | QAMEDIKQME | 10 | Alpha S1 casein | 2 |  | 611.78 | 1221.54 | 35.08 | 67-76 |  | + | 1 |
| **62** | QKEPMIGV | 8 | Alpha S1 casein | 2 |  | 451.24 | 900.47 | 36.18 | 146-153 |  | + | 1 |
| **63** | QKEPMIGVN | 9 | Alpha S1 casein | 2 |  | 508.27 | 1014.52 | 32.10 | 146-154 |  | + | 1 |
| **64** | QKEPMIGVNQ | 10 | Alpha S1 casein | 2 |  | 572.29 | 1142.58 | 31.76 | 146-155 |  | + | 1 |
| **65** | QKEPMIGVNQE | 11 | Alpha S1 casein | 2 |  | 636.82 | 1271.62 | 32.44 | 146-156 |  | + | 1 |
| **66** | QKEPMIGVNQELAY | 14 | Alpha S1 casein | 2 |  | 810.41 | 1618.80 | 42.79 | 146-159 |  | + | 1 |
| **67** | RFFVAPFPE | 9 | Alpha S1 casein | 2 |  | 555.29 | 1108.57 | 45.97 | 37-45 |  | + | 1 |
| **68** | RLH | 3 | Alpha S1 casein | 1 |  | 425.26 | 424.25 | 28.18 | 134-136 |  | + | 1 |
| **69** | SENSEKTTMPLW | 12 | Alpha S1 casein | 2 |  | 711.83 | 1421.65 | 43.98 | 203-214 |  | + | 1 |
| **70** | SFSDIPNPIGSENSEK | 16 | Alpha S1 casein | 2 |  | 860.90 | 1719.80 | 42.66 | 193-208 |  | + | 1 |
| **71** | SKDIGSESTED | 11 | Alpha S1 casein | 2 |  | 584.25 | 1166.49 | 18.38 | 56-66 |  | + | 1 |
| **72** | SVEQKHIQKE | 10 | Alpha S1 casein | 2 |  | 613.33 | 1224.65 | 13.49 | 90-99 |  | + | 1 |
| **73** | TDAPSFSDIPNPIGSENSEKTTMPLW | 26 | Alpha S1 casein | 2 |  | 1417.66 | 2833.31 | 49.22 | 189-214 |  | + | 1 |
| **74** | VAPFPEVF | 8 | Alpha S1 casein | 1 |  | 905.48 | 904.47 | 50.42 | 40-47 |  | + | 1 |
| **75** | VAPFPEVFGK | 10 | Alpha S1 casein | 2 |  | 545.80 | 1089.59 | 44.92 | 40-49 |  | + | 1 |
| **76** | VFGKEKVN | 8 | Alpha S1 casein | 2 |  | 460.76 | 919.51 | 19.12 | 46-53 |  | + | 1 |
| **77** | VFGKEKVNEL | 10 | Alpha S1 casein | 2 |  | 581.83 | 1161.64 | 31.31 | 46-55 |  | + | 1 |
| **78** | VFGKEKVNELS | 11 | Alpha S1 casein | 2 |  | 625.34 | 1248.67 | 29.37 | 46-56 |  | + | 1 |
| **79** | VLNENLLRF | 9 | Alpha S1 casein | 2 |  | 559.32 | 1116.63 | 45.51 | 30-38 |  | + | 1 |
| **80** | VLNENLLRFF | 10 | Alpha S1 casein | 2 |  | 632.86 | 1263.70 | 49.75 | 30-39 |  | + | 1 |
| **81** | VPQLEIVPN | 9 | Alpha S1 casein | 2 |  | 504.79 | 1007.57 | 45.24 | 121-129 |  | + | 1 |
| **82** | YKVPQLE | 7 | Alpha S1 casein | 2 |  | 438.74 | 875.48 | 37.73 | 119-125 |  | + | 1 |
| **83** | LNREQL | 6 | Alpha S2 casein | 2 |  | 386.72 | 771.42 | 25.07 | 138-143 | + | + | 2 |
| **84** | LYQGPIVLNPWDQVK | 15 | Alpha S2 casein | 2 |  | 885.48 | 1768.95 | 46.92 | 114-128 | + | + | 2 |
| **85** | LYQGPIVLNPWDQVKR | 16 | Alpha S2 casein | 2 |  | 963.53 | 1925.05 | 44.60 | 114-129 | + | + | 2 |
| **86** | LYQGPIVLNPWDQVKRN | 17 | Alpha S2 casein | 3 |  | 680.71 | 2039.10 | 44.54 | 114-130 | + | + | 2 |
| **87** | QGPIVLNPWDQVKRN | 15 | Alpha S2 casein | 3 |  | 588.66 | 1762.95 | 43.11 | 116-130 | + |  | 1 |
| **88** | QYLYQGPIVLNPWDQVKRN | 19 | Alpha S2 casein | 3 |  | 777.75 | 2330.22 | 45.59 | 112-130 | + | + | 2 |
| **89** | YLYQGPIVLNPWDQVKRN | 18 | Alpha S2 casein | 3 |  | 735.06 | 2202.16 | 45.45 | 113-130 | + | + | 2 |
| **90** | YQGPIVLNPWDQVK | 14 | Alpha S2 casein | 2 |  | 828.94 | 1655.87 | 45.93 | 115-128 | + | + | 2 |
| **91** | YQGPIVLNPWDQVKRN | 16 | Alpha S2 casein | 2 |  | 964.01 | 1926.01 | 43.82 | 115-130 | + | + | 2 |
| **92** | AINPSKEN | 8 | Alpha S2 casein | 2 |  | 436.73 | 871.44 | 16.46 | 42-49 |  | + | 1 |
| **93** | EEEKNRLN | 8 | Alpha S2 casein | 2 |  | 516.26 | 1030.50 | 13.82 | 170-177 |  | + | 1 |
| **94** | EVVRNANEE | 9 | Alpha S2 casein | 2 |  | 530.26 | 1058.50 | 17.44 | 57-65 |  | + | 1 |
| **95** | IISQETYKQ | 9 | Alpha S2 casein | 2 |  | 555.30 | 1108.58 | 25.43 | 29-37 |  | + | 1 |
| **96** | IISQETYKQEK | 11 | Alpha S2 casein | 2 |  | 683.86 | 1365.71 | 21.90 | 29-39 |  | + | 1 |
| **97** | NSKKTVDME | 9 | Alpha S2 casein | 2 |  | 526.26 | 1050.50 | 15.61 | 149-157 |  | + | 1 |
| **98** | STEVFTKK | 8 | Alpha S2 casein | 2 |  | 470.26 | 938.51 | 18.69 | 158-165 |  | + | 1 |
| **99** | STEVFTKKTK | 10 | Alpha S2 casein | 2 |  | 584.83 | 1167.65 | 15.80 | 158-167 |  | + | 1 |
| **100** | TEEEKNRLN | 9 | Alpha S2 casein | 2 |  | 566.78 | 1131.55 | 14.60 | 169-177 |  | + | 1 |
| **101** | TKKTKLTEE | 9 | Alpha S2 casein | 2 |  | 539.31 | 1076.61 | 13.09 | 163-171 |  | + | 1 |
| **102** | TKVIPYVRYL | 10 | Alpha S2 casein | 2 |  | 626.38 | 1250.74 | 42.54 | 213-222 |  | + | 1 |
| **103** | VFTKKTKLTEE | 11 | Alpha S2 casein | 2 |  | 662.38 | 1322.74 | 20.76 | 161-171 |  | + | 1 |
| **104** | AQTQSLVYPFPGPIPN | 16 | Beta casein | 2 |  | 864.95 | 1727.89 | 48.99 | 68-83 | + | + | 2 |
| **105** | AVPYPQRDMPIQA | 13 | Beta casein | 2 |  | 743.38 | 1484.74 | 41.42 | 192-204 | + | + | 2 |
| **106** | DELQDKIHPF | 10 | Beta casein | 2 |  | 621.31 | 1240.61 | 41.87 | 58-67 | + | + | 2 |
| **107** | DELQDKIHPFAQTQ | 14 | Beta casein | 2 |  | 835.41 | 1668.81 | 41.14 | 58-71 | + | + | 2 |
| **108** | DELQDKIHPFAQTQS | 15 | Beta casein | 2 |  | 878.93 | 1755.84 | 40.98 | 58-72 | + |  | 1 |
| **109** | DELQDKIHPFAQTQSL | 16 | Beta casein | 2 |  | 935.47 | 1868.93 | 43.02 | 58-73 | + |  | 1 |
| **110** | DKIHPF | 6 | Beta casein | 2 |  | 378.71 | 755.40 | 29.16 | 62-67 | + | + | 2 |
| **111** | DKIHPFAQT | 9 | Beta casein | 2 |  | 528.78 | 1055.54 | 29.24 | 62-70 | + | + | 2 |
| **112** | DKIHPFAQTQ | 10 | Beta casein | 2 |  | 592.81 | 1183.60 | 28.43 | 62-71 | + | + | 2 |
| **113** | DKIHPFAQTQSL | 12 | Beta casein | 2 |  | 692.86 | 1383.71 | 38.09 | 62-73 | + | + | 2 |
| **114** | DVENLHLPLPL | 11 | Beta casein | 2 |  | 630.35 | 1258.69 | 50.73 | 144-154 | + | + | 2 |
| **115** | DVENLHLPLPLL | 12 | Beta casein | 2 |  | 686.90 | 1371.78 | 53.21 | 144-155 | + | + | 2 |
| **116** | DVENLHLPLPLLQ | 13 | Beta casein | 2 |  | 750.92 | 1499.83 | 51.98 | 144-156 | + | + | 2 |
| **117** | DVENLHLPLPLLQSW | 15 | Beta casein | 2 |  | 887.48 | 1772.95 | 54.78 | 144-158 | + | + | 2 |
| **118** | EDELQDKIHPFAQTQ | 15 | Beta casein | 2 |  | 899.93 | 1797.85 | 41.03 | 57-71 | + | + | 2 |
| **119** | EDELQDKIHPFAQTQSL | 17 | Beta casein | 2 |  | 999.99 | 1997.97 | 42.85 | 57-73 | + |  | 1 |
| **120** | EEQQQTEDELQDKIHPF | 17 | Beta casein | 3 |  | 705.33 | 2112.96 | 42.02 | 51-67 | + |  | 1 |
| **121** | ELQDKIHPF | 9 | Beta casein | 2 |  | 563.80 | 1125.58 | 37.19 | 59-67 | + | + | 2 |
| **122** | ELQDKIHPFAQT | 12 | Beta casein | 2 |  | 713.87 | 1425.73 | 37.53 | 59-70 | + |  | 1 |
| **123** | ELQDKIHPFAQTQ | 13 | Beta casein | 2 |  | 777.90 | 1553.78 | 36.80 | 59-71 | + | + | 2 |
| **124** | ELQDKIHPFAQTQSL | 15 | Beta casein | 2 |  | 877.96 | 1753.90 | 41.69 | 59-73 | + | + | 2 |
| **125** | EMPFPKYPVEPF | 12 | Beta casein | 2 |  | 748.86 | 1495.71 | 45.24 | 123-134 | + | + | 2 |
| **126** | EMPFPKYPVEPFTES | 15 | Beta casein | 2 |  | 899.42 | 1796.83 | 46.17 | 123-137 | + | + | 2 |
| **127** | EPVLGPVRGPFPIIV | 15 | Beta casein | 2 |  | 795.47 | 1588.93 | 50.14 | 210-224 | + |  | 1 |
| **128** | EQQQTEDELQ | 10 | Beta casein | 2 |  | 624.27 | 1246.53 | 27.62 | 52-61 | + | + | 2 |
| **129** | EQQQTEDELQDK | 12 | Beta casein | 2 |  | 745.83 | 1489.65 | 23.63 | 52-63 | + | + | 2 |
| **130** | EQQQTEDELQDKIHPF | 16 | Beta casein | 2 |  | 992.97 | 1983.92 | 40.81 | 52-67 | + | + | 2 |
| **131** | EQQQTEDELQDKIHPFAQT | 19 | Beta casein | 3 |  | 762.36 | 2284.06 | 41.53 | 52-70 | + |  | 1 |
| **132** | EQQQTEDELQDKIHPFAQTQ | 20 | Beta casein | 3 |  | 805.05 | 2412.12 | 40.98 | 52-71 | + | + | 2 |
| **133** | EQQQTEDELQDKIHPFAQTQS | 21 | Beta casein | 2 |  | 1250.58 | 2499.15 | 41.20 | 52-72 | + |  | 1 |
| **134** | EQQQTEDELQDKIHPFAQTQSL | 22 | Beta casein | 3 |  | 871.75 | 2612.24 | 42.13 | 52-73 | + | + | 2 |
| **135** | FPGPIPN | 7 | Beta casein | 1 |  | 741.39 | 740.39 | 43.76 | 77-83 | + |  | 1 |
| **136** | FQSEEQQQTEDELQDK | 16 | Beta casein | 2 |  | 991.43 | 1980.85 | 32.28 | 48-63 | + |  | 1 |
| **137** | GPVRGPFPIIV | 11 | Beta casein | 2 |  | 576.35 | 1150.69 | 47.03 | 214-224 | + | + | 2 |
| **138** | GVSKVKEA | 8 | Beta casein | 2 |  | 409.24 | 816.47 | 13.49 | 109-116 | + | + | 2 |
| **139** | GVSKVKEAMAPK | 12 | Beta casein | 3 |  | 415.57 | 1243.70 | 19.45 | 109-120 | + | + | 2 |
| **140** | HKEMPFPKYPVEPF | 14 | Beta casein | 3 |  | 582.63 | 1744.86 | 42.62 | 121-134 | + | + | 2 |
| **141** | HKEMPFPKYPVEPFTES | 17 | Beta casein | 3 |  | 688.34 | 2061.99 | 42.40 | 121-137 | + |  | 1 |
| **142** | KAVPYPQ | 7 | Beta casein | 2 |  | 401.73 | 801.44 | 25.36 | 191-197 | + | + | 2 |
| **143** | KAVPYPQR | 8 | Beta casein | 2 |  | 479.78 | 957.54 | 20.42 | 191-198 | + | + | 2 |
| **144** | KAVPYPQRDMPIQA | 14 | Beta casein | 2 |  | 807.43 | 1612.84 | 36.18 | 191-204 | + | + | 2 |
| **145** | KVLPVPQ | 7 | Beta casein | 2 |  | 390.75 | 779.49 | 33.06 | 184-190 | + |  | 1 |
| **146** | KVLPVPQK | 8 | Beta casein | 2 |  | 454.80 | 907.59 | 24.37 | 184-191 | + | + | 2 |
| **147** | KVLPVPQKAVPYPQ | 14 | Beta casein | 2 |  | 782.47 | 1562.92 | 37.53 | 184-197 | + | + | 2 |
| **148** | LHLPLPLLQ | 9 | Beta casein | 2 |  | 522.33 | 1042.65 | 49.37 | 148-156 | + | + | 2 |
| **149** | LLYQEPVLGPVRGPFP | 16 | Beta casein | 2 |  | 891.50 | 1780.99 | 47.43 | 206-221 | + | + | 2 |
| **150** | LLYQEPVLGPVRGPFPII | 18 | Beta casein | 2 |  | 1004.59 | 2007.16 | 51.19 | 206-223 | + | + | 2 |
| **151** | LLYQEPVLGPVRGPFPIIV | 19 | Beta casein | 2 |  | 1054.12 | 2106.22 | 51.81 | 206-224 | + | + | 2 |
| **152** | LQSWMHQPHQPLPPT | 15 | Beta casein | 2 |  | 898.95 | 1795.88 | 39.28 | 155-169 | + | + | 2 |
| **153** | LSQSKVLPVPQ | 11 | Beta casein | 2 |  | 598.36 | 1194.70 | 38.46 | 180-190 | + | + | 2 |
| **154** | LYQEPVLGPVRGPFPIIV | 18 | Beta casein | 2 |  | 997.58 | 1993.14 | 50.78 | 207-224 | + | + | 2 |
| **155** | MAPKHKEMPFPKYPVEPF | 18 | Beta casein | 3 |  | 725.04 | 2172.09 | 41.87 | 117-134 | + | + | 2 |
| **156** | MHQPHQPLPPT | 11 | Beta casein | 2 |  | 641.82 | 1281.63 | 26.31 | 159-169 | + | + | 2 |
| **157** | MHQPHQPLPPTVMFPPQ | 17 | Beta casein | 3 |  | 661.33 | 1980.97 | 42.56 | 159-175 | + |  | 1 |
| **158** | MPFPKYPVEPF | 11 | Beta casein | 2 |  | 676.34 | 1350.67 | 46.33 | 124-134 | + | + | 2 |
| **159** | NIPPLTQTPVVVPPFLQPEVM | 21 | Beta casein | 2 |  | 1166.63 | 2331.25 | 53.74 | 88-108 | + | + | 2 |
| **160** | NIPPLTQTPVVVPPFLQPEVMGV | 23 | Beta casein | 2 |  | 1236.68 | 2471.35 | 55.06 | 88-110 | + | + | 2 |
| **161** | NIPPLTQTPVVVPPFLQPEVMGVSKV | 26 | Beta casein | 2 |  | 1393.78 | 2785.55 | 52.28 | 88-113 | + |  | 1 |
| **162** | NIPPLTQTPVVVPPFLQPEVMGVSKVKEA | 29 | Beta casein | 3 |  | 1038.91 | 3113.72 | 49.45 | 88-116 | + |  | 1 |
| **163** | NLHLPLPL | 8 | Beta casein | 2 |  | 458.78 | 915.55 | 47.97 | 147-154 | + | + | 2 |
| **164** | NLHLPLPLLQ | 10 | Beta casein | 2 |  | 579.36 | 1156.70 | 49.68 | 147-156 | + | + | 2 |
| **165** | QDKIHPFAQTQ | 11 | Beta casein | 2 |  | 656.84 | 1311.66 | 27.90 | 61-71 | + |  | 1 |
| **166** | QDKIHPFAQTQSL | 13 | Beta casein | 2 |  | 756.89 | 1511.77 | 37.08 | 61-73 | + | + | 2 |
| **167** | QEPVLGPVRGPFPIIV | 16 | Beta casein | 2 |  | 859.50 | 1716.99 | 49.78 | 209-224 | + | + | 2 |
| **168** | QQQTEDELQDKIHPF | 15 | Beta casein | 3 |  | 619.30 | 1854.87 | 41.53 | 53-67 | + | + | 2 |
| **169** | QQTEDELQDKIHPF | 14 | Beta casein | 3 |  | 576.61 | 1726.82 | 41.58 | 54-67 | + | + | 2 |
| **170** | QSEEQQQTEDELQDK | 15 | Beta casein | 2 |  | 917.90 | 1833.79 | 27.62 | 49-63 | + | + | 2 |
| **171** | QSEEQQQTEDELQDKIHPF | 19 | Beta casein | 3 |  | 777.02 | 2328.05 | 42.23 | 49-67 | + | + | 2 |
| **172** | QSEEQQQTEDELQDKIHPFAQTQ | 23 | Beta casein | 3 |  | 919.76 | 2756.25 | 41.75 | 49-71 | + |  | 1 |
| **173** | QSEEQQQTEDELQDKIHPFAQTQSL | 25 | Beta casein | 3 |  | 986.46 | 2956.37 | 43.11 | 49-73 | + |  | 1 |
| **174** | QSKVLPVPQ | 9 | Beta casein | 2 |  | 498.30 | 994.58 | 34.61 | 182-190 | + | + | 2 |
| **175** | QSKVLPVPQKAVPYPQ | 16 | Beta casein | 3 |  | 593.68 | 1778.01 | 38.58 | 182-197 | + | + | 2 |
| **176** | QSWMHQPHQPLPPT | 14 | Beta casein | 3 |  | 561.94 | 1682.80 | 36.44 | 156-169 | + | + | 2 |
| **177** | QTEDELQDKIHPF | 13 | Beta casein | 2 |  | 800.39 | 1598.76 | 41.53 | 55-67 | + | + | 2 |
| **178** | QTEDELQDKIHPFAQTQ | 17 | Beta casein | 2 |  | 1014.49 | 2026.96 | 40.88 | 55-71 | + |  | 1 |
| **179** | QTEDELQDKIHPFAQTQSL | 19 | Beta casein | 2 |  | 1114.55 | 2227.08 | 42.62 | 55-73 | + |  | 1 |
| **180** | RDMPIQAF | 8 | Beta casein | 2 |  | 489.25 | 976.48 | 42.56 | 198-205 | + | + | 2 |
| **181** | RDMPIQAFL | 9 | Beta casein | 2 |  | 545.79 | 1089.56 | 46.69 | 198-206 | + | + | 2 |
| **182** | RDMPIQAFLL | 10 | Beta casein | 2 |  | 602.33 | 1202.65 | 49.25 | 198-207 | + | + | 2 |
| **183** | RELEEL | 6 | Beta casein | 2 |  | 394.71 | 787.41 | 32.86 | 16-21 | + | + | 2 |
| **184** | SEEQQQTEDELQDK | 14 | Beta casein | 2 |  | 853.87 | 1705.73 | 26.65 | 50-63 | + | + | 2 |
| **185** | SEEQQQTEDELQDKIHPF | 18 | Beta casein | 3 |  | 734.34 | 2199.99 | 42.18 | 50-67 | + |  | 1 |
| **186** | SKVLPVPQKAVPYPQ | 15 | Beta casein | 3 |  | 550.99 | 1649.95 | 38.38 | 183-197 | + | + | 2 |
| **187** | SLSQSKVLPVPQ | 12 | Beta casein | 2 |  | 641.87 | 1281.73 | 40.41 | 179-190 | + | + | 2 |
| **188** | SLSQSKVLPVPQKAVPYPQ | 19 | Beta casein | 3 |  | 689.39 | 2065.16 | 41.20 | 179-197 | + | + | 2 |
| **189** | SLVYPFPGPIPN | 12 | Beta casein | 2 |  | 650.85 | 1299.69 | 49.78 | 72-83 | + | + | 2 |
| **190** | SLVYPFPGPIPNSLPQ | 16 | Beta casein | 2 |  | 863.46 | 1724.91 | 50.90 | 72-87 | + |  | 1 |
| **191** | SQSKVLPVPQ | 10 | Beta casein | 2 |  | 541.81 | 1081.61 | 34.85 | 181-190 | + | + | 2 |
| **192** | SQSKVLPVPQKAVPYPQ | 17 | Beta casein | 2 |  | 933.53 | 1865.04 | 38.79 | 181-197 | + | + | 2 |
| **193** | SVLSLSQSKVLPVPQ | 15 | Beta casein | 2 |  | 791.46 | 1580.91 | 44.48 | 176-190 | + | + | 2 |
| **194** | SVLSLSQSKVLPVPQK | 16 | Beta casein | 2 |  | 855.51 | 1709.01 | 42.13 | 176-191 | + |  | 1 |
| **195** | SVLSLSQSKVLPVPQKAVPYPQ | 22 | Beta casein | 3 |  | 789.12 | 2364.34 | 43.54 | 176-197 | + | + | 2 |
| **196** | SWMHQPHQPLPPT | 13 | Beta casein | 2 |  | 778.38 | 1554.74 | 35.54 | 157-169 | + | + | 2 |
| **197** | SWMHQPHQPLPPTVMFPPQ | 19 | Beta casein | 3 |  | 752.37 | 2254.08 | 43.99 | 157-175 | + | + | 2 |
| **198** | SWMHQPHQPLPPTVMFPPQSVL | 22 | Beta casein | 3 |  | 852.10 | 2553.27 | 46.53 | 157-178 | + | + | 2 |
| **199** | TDVENLHLPLPL | 12 | Beta casein | 2 |  | 680.88 | 1359.74 | 49.52 | 143-154 | + | + | 2 |
| **200** | TDVENLHLPLPLLQ | 14 | Beta casein | 2 |  | 801.45 | 1600.88 | 50.76 | 143-156 | + | + | 2 |
| **201** | TEDELQDKIHPF | 12 | Beta casein | 2 |  | 736.36 | 1470.70 | 40.66 | 56-67 | + | + | 2 |
| **202** | TEDELQDKIHPFAQTQ | 16 | Beta casein | 2 |  | 950.46 | 1898.90 | 40.88 | 56-71 | + | + | 2 |
| **203** | TEDELQDKIHPFAQTQSL | 18 | Beta casein | 2 |  | 1050.52 | 2099.02 | 42.68 | 56-73 | + | + | 2 |
| **204** | TESQSLTLT | 9 | Beta casein | 2 |  | 490.25 | 978.49 | 39.71 | 135-143 | + | + | 2 |
| **205** | TQTPVVVPPFLQPE | 14 | Beta casein | 2 |  | 776.42 | 1550.83 | 50.57 | 93-106 | + | + | 2 |
| **206** | TQTPVVVPPFLQPEVM | 16 | Beta casein | 2 |  | 891.48 | 1780.94 | 53.06 | 93-108 | + | + | 2 |
| **207** | VENLHLPLPLLQ | 12 | Beta casein | 2 |  | 693.41 | 1384.81 | 49.79 | 145-156 | + | + | 2 |
| **208** | VLGPVRGPFP | 10 | Beta casein | 2 |  | 519.81 | 1037.60 | 43.34 | 212-221 | + |  | 1 |
| **209** | VLGPVRGPFPIIV | 13 | Beta casein | 2 |  | 682.43 | 1362.84 | 48.99 | 212-224 | + | + | 2 |
| **210** | VLPVPQKAVPYPQ | 13 | Beta casein | 2 |  | 718.42 | 1434.82 | 41.75 | 185-197 | + | + | 2 |
| **211** | VMFPPQSVLSLSQSK | 15 | Beta casein | 2 |  | 824.44 | 1646.87 | 45.79 | 170-184 | + |  | 1 |
| **212** | VMGVSKVKEA | 10 | Beta casein | 2 |  | 524.30 | 1046.58 | 23.45 | 107-116 | + | + | 2 |
| **213** | VMGVSKVKEAMAPK | 14 | Beta casein | 3 |  | 492.28 | 1473.80 | 29.89 | 107-120 | + | + | 2 |
| **214** | VYPFPGPIPN | 10 | Beta casein | 2 |  | 550.79 | 1099.57 | 47.47 | 74-83 | + | + | 2 |
| **215** | VYPFPGPIPNSLPQ | 14 | Beta casein | 2 |  | 763.41 | 1524.80 | 48.99 | 74-87 | + | + | 2 |
| **216** | YPFPGPIPNSLPQ | 13 | Beta casein | 2 |  | 713.87 | 1425.73 | 48.03 | 75-87 | + |  | 1 |
| **217** | YQEPVLGPVRGPFP | 14 | Beta casein | 2 |  | 778.42 | 1554.82 | 45.12 | 208-221 | + | + | 2 |
| **218** | YQEPVLGPVRGPFPI | 15 | Beta casein | 2 |  | 834.96 | 1667.90 | 47.59 | 208-222 | + | + | 2 |
| **219** | YQEPVLGPVRGPFPIIV | 17 | Beta casein | 2 |  | 941.04 | 1880.06 | 50.27 | 208-224 | + | + | 2 |
| **220** | APKHKEMPFPKYPVEPF | 17 | Beta casein | 3 |  | 681.36 | 2041.05 | 41.65 | 118-134 |  | + | 1 |
| **221** | AQTQSL | 6 | Beta casein | 1 |  | 647.34 | 646.33 | 24.21 | 68-73 |  | + | 1 |
| **222** | AVPYPQRDMPIQ | 12 | Beta casein | 2 |  | 707.86 | 1413.71 | 40.11 | 192-203 |  | + | 1 |
| **223** | AVPYPQRDMPIQAF | 14 | Beta casein | 2 |  | 816.91 | 1631.81 | 44.48 | 192-205 |  | + | 1 |
| **224** | DELQDKIHP | 9 | Beta casein | 2 |  | 547.78 | 1093.54 | 32.57 | 58-66 |  | + | 1 |
| **225** | DKIHPFAQTQS | 11 | Beta casein | 2 |  | 636.32 | 1270.63 | 28.12 | 62-72 |  | + | 1 |
| **226** | DMPIQAFLL | 9 | Beta casein | 2 |  | 524.28 | 1046.55 | 55.27 | 199-207 |  | + | 1 |
| **227** | DVENLHLP | 8 | Beta casein | 2 |  | 468.74 | 935.47 | 43.68 | 144-151 |  | + | 1 |
| **228** | DVENLHLPLP | 10 | Beta casein | 2 |  | 573.81 | 1145.61 | 47.54 | 144-153 |  | + | 1 |
| **229** | DVENLHLPLPLLQS | 14 | Beta casein | 2 |  | 794.44 | 1586.87 | 51.55 | 144-157 |  | + | 1 |
| **230** | EDELQDKIHPF | 11 | Beta casein | 2 |  | 685.83 | 1369.65 | 40.51 | 57-67 |  | + | 1 |
| **231** | ELQDKIHP | 8 | Beta casein | 2 |  | 490.26 | 978.51 | 27.44 | 59-66 |  | + | 1 |
| **232** | ENLHLPLPLLQ | 11 | Beta casein | 2 |  | 643.88 | 1285.74 | 49.61 | 146-156 |  | + | 1 |
| **233** | FLLYQEPVLGPVRGPFPIIV | 20 | Beta casein | 2 |  | 1127.65 | 2253.29 | 53.65 | 205-224 |  | + | 1 |
| **234** | FPKYPVEPF | 9 | Beta casein | 2 |  | 562.29 | 1122.57 | 44.53 | 126-134 |  | + | 1 |
| **235** | GVSKVKEAM | 9 | Beta casein | 2 |  | 474.76 | 947.51 | 19.61 | 109-117 |  | + | 1 |
| **236** | GVSKVKEAMAP | 11 | Beta casein | 2 |  | 558.81 | 1115.60 | 24.17 | 109-119 |  | + | 1 |
| **237** | HLPLPLLQ | 8 | Beta casein | 2 |  | 465.79 | 929.57 | 46.52 | 149-156 |  | + | 1 |
| **238** | HPF | 3 | Beta casein | 1 |  | 400.20 | 399.19 | 21.06 | 65-67 |  | + | 1 |
| **239** | IHPFAQTQ | 8 | Beta casein | 2 |  | 471.25 | 940.48 | 29.54 | 64-71 |  | + | 1 |
| **240** | ITRINK | 6 | Beta casein | 2 |  | 372.74 | 743.47 | 14.26 | 38-43 |  | + | 1 |
| **241** | KAVPYPQRD | 9 | Beta casein | 2 |  | 537.29 | 1072.57 | 20.78 | 191-199 |  | + | 1 |
| **242** | KAVPYPQRDMPIQ | 13 | Beta casein | 2 |  | 779.91 | 1557.80 | 27.90 | 191-203 |  | + | 1 |
| **243** | KAVPYPQRDMPIQAF | 15 | Beta casein | 2 |  | 880.96 | 1759.91 | 42.27 | 191-205 |  | + | 1 |
| **244** | KAVPYPQRDMPIQAFL | 16 | Beta casein | 3 |  | 625.34 | 1872.99 | 45.02 | 191-206 |  | + | 1 |
| **245** | KIEKFQSE | 8 | Beta casein | 2 |  | 504.77 | 1007.53 | 19.55 | 44-51 |  | + | 1 |
| **246** | KIHPFAQTQ | 9 | Beta casein | 2 |  | 535.29 | 1068.57 | 23.58 | 63-71 |  | + | 1 |
| **247** | KKIEKFQ | 7 | Beta casein | 2 |  | 460.78 | 919.55 | 14.54 | 43-49 |  | + | 1 |
| **248** | KVKEAMAPK | 9 | Beta casein | 2 |  | 501.29 | 1000.57 | 14.01 | 112-120 |  | + | 1 |
| **249** | LGPVRGPFPIIV | 12 | Beta casein | 2 |  | 632.89 | 1263.77 | 48.48 | 213-224 |  | + | 1 |
| **250** | LHLPLPL | 7 | Beta casein | 2 |  | 401.76 | 801.51 | 47.63 | 148-154 |  | + | 1 |
| **251** | LHLPLPLLQS | 10 | Beta casein | 2 |  | 565.85 | 1129.69 | 49.05 | 148-157 |  | + | 1 |
| **252** | LLYQEPVLGPVRGP | 14 | Beta casein | 2 |  | 769.44 | 1536.87 | 44.37 | 206-219 |  | + | 1 |
| **253** | LLYQEPVLGPVRGPFPI | 17 | Beta casein | 2 |  | 948.04 | 1894.07 | 49.50 | 206-222 |  | + | 1 |
| **254** | LNVPGEIVE | 9 | Beta casein | 1 |  | 969.53 | 968.52 | 44.15 | 21-29 |  | + | 1 |
| **255** | LQDKIHPF | 8 | Beta casein | 2 |  | 499.28 | 996.54 | 33.51 | 60-67 |  | + | 1 |
| **256** | LQSWMHQPHQPLPPTVMFPPQ | 21 | Beta casein | 3 |  | 832.75 | 2495.22 | 44.50 | 155-175 |  | + | 1 |
| **257** | LSLSQSKVLPVPQ | 13 | Beta casein | 2 |  | 698.41 | 1394.81 | 43.06 | 178-190 |  | + | 1 |
| **258** | LTDVENLHLPLPL | 13 | Beta casein | 2 |  | 737.42 | 1472.82 | 50.09 | 142-154 |  | + | 1 |
| **259** | LTQTPVVVPPFLQPEVM | 17 | Beta casein | 2 |  | 948.02 | 1894.03 | 53.71 | 92-108 |  | + | 1 |
| **260** | LVYPFPGPIPN | 11 | Beta casein | 2 |  | 607.33 | 1212.65 | 49.21 | 73-83 |  | + | 1 |
| **261** | LYQEPVLGPVRGPFP | 15 | Beta casein | 2 |  | 834.96 | 1667.90 | 45.81 | 207-221 |  | + | 1 |
| **262** | LYQEPVLGPVRGPFPII | 17 | Beta casein | 2 |  | 948.04 | 1894.07 | 49.99 | 207-223 |  | + | 1 |
| **263** | NIPPLTQTPV | 10 | Beta casein | 2 |  | 540.31 | 1078.60 | 44.53 | 88-97 |  | + | 1 |
| **264** | NIPPLTQTPVVVPPFLQP | 18 | Beta casein | 2 |  | 979.06 | 1956.11 | 53.88 | 88-105 |  | + | 1 |
| **265** | NIPPLTQTPVVVPPFLQPE | 19 | Beta casein | 2 |  | 1043.58 | 2085.15 | 53.38 | 88-106 |  | + | 1 |
| **266** | NIPPLTQTPVVVPPFLQPEVMGVSK | 25 | Beta casein | 2 |  | 1344.25 | 2686.48 | 51.55 | 88-112 |  | + | 1 |
| **267** | NIPPLTQTPVVVPPFLQPEVMGVSKVK | 27 | Beta casein | 3 |  | 972.22 | 2913.64 | 49.44 | 88-114 |  | + | 1 |
| **268** | NLHLPLP | 7 | Beta casein | 2 |  | 402.24 | 802.47 | 44.80 | 147-153 |  | + | 1 |
| **269** | NLHLPLPLL | 9 | Beta casein | 2 |  | 515.33 | 1028.64 | 50.47 | 147-155 |  | + | 1 |
| **270** | NLHLPLPLLQS | 11 | Beta casein | 2 |  | 622.87 | 1243.73 | 49.33 | 147-157 |  | + | 1 |
| **271** | NLHLPLPLLQSW | 12 | Beta casein | 2 |  | 715.91 | 1429.81 | 52.58 | 147-158 |  | + | 1 |
| **272** | NVPGEIVE | 8 | Beta casein | 2 |  | 428.72 | 855.43 | 39.27 | 22-29 |  | + | 1 |
| **273** | PFPKYPVEPF | 10 | Beta casein | 2 |  | 610.82 | 1219.63 | 44.93 | 125-134 |  | + | 1 |
| **274** | PLPPT | 5 | Beta casein | 1 |  | 524.31 | 523.30 | 30.52 | 165-169 |  | + | 1 |
| **275** | QDKIHPF | 7 | Beta casein | 2 |  | 442.73 | 883.46 | 28.46 | 61-67 |  | + | 1 |
| **276** | QEPVLGPVRGPFPII | 15 | Beta casein | 2 |  | 809.97 | 1617.92 | 48.82 | 209-223 |  | + | 1 |
| **277** | QQQTEDELQ | 9 | Beta casein | 2 |  | 559.75 | 1117.49 | 26.54 | 53-61 |  | + | 1 |
| **278** | QQQTEDELQDK | 11 | Beta casein | 2 |  | 681.31 | 1360.61 | 22.38 | 53-63 |  | + | 1 |
| **279** | QQTEDELQ | 8 | Beta casein | 2 |  | 495.72 | 989.43 | 26.27 | 54-61 |  | + | 1 |
| **280** | QQTEDELQDK | 10 | Beta casein | 2 |  | 617.28 | 1232.55 | 22.04 | 54-63 |  | + | 1 |
| **281** | QSEEQQQTE | 9 | Beta casein | 2 |  | 553.73 | 1105.45 | 15.40 | 49-57 |  | + | 1 |
| **282** | QSEEQQQTED | 10 | Beta casein | 2 |  | 611.25 | 1220.48 | 16.05 | 49-58 |  | + | 1 |
| **283** | QSLVYPFPGPIPN | 13 | Beta casein | 2 |  | 714.88 | 1427.74 | 49.39 | 71-83 |  | + | 1 |
| **284** | QTEDELQDK | 9 | Beta casein | 2 |  | 553.25 | 1104.49 | 21.00 | 55-63 |  | + | 1 |
| **285** | RDMPIQ | 6 | Beta casein | 2 |  | 380.19 | 758.37 | 25.79 | 198-203 |  | + | 1 |
| **286** | RDMPIQA | 7 | Beta casein | 2 |  | 415.71 | 829.41 | 30.23 | 198-204 |  | + | 1 |
| **287** | RDMPIQAFLLY | 11 | Beta casein | 2 |  | 683.86 | 1365.71 | 49.84 | 198-208 |  | + | 1 |
| **288** | RELEELNVPG | 10 | Beta casein | 2 |  | 578.30 | 1154.59 | 40.00 | 16-25 |  | + | 1 |
| **289** | SITRIN | 6 | Beta casein | 2 |  | 352.21 | 702.40 | 22.64 | 37-42 |  | + | 1 |
| **290** | SITRINK | 7 | Beta casein | 2 |  | 416.26 | 830.50 | 16.74 | 37-43 |  | + | 1 |
| **291** | SLPQNIPPL | 9 | Beta casein | 2 |  | 489.78 | 977.55 | 46.31 | 84-92 |  | + | 1 |
| **292** | SLPQNIPPLTQTPVVVPPFLQPE | 23 | Beta casein | 2 |  | 1256.20 | 2510.38 | 53.65 | 84-106 |  | + | 1 |
| **293** | SLPQNIPPLTQTPVVVPPFLQPEVM | 25 | Beta casein | 2 |  | 1371.25 | 2740.49 | 54.90 | 84-108 |  | + | 1 |
| **294** | SLSQSKVLPVPQK | 13 | Beta casein | 2 |  | 705.92 | 1409.82 | 32.49 | 179-191 |  | + | 1 |
| **295** | SLTLTDVEN | 9 | Beta casein | 1 |  | 991.49 | 990.49 | 42.94 | 139-147 |  | + | 1 |
| **296** | SWMHQPHQPLPP | 12 | Beta casein | 2 |  | 727.85 | 1453.69 | 35.40 | 157-168 |  | + | 1 |
| **297** | TEDELQDK | 8 | Beta casein | 2 |  | 489.22 | 976.43 | 20.11 | 56-63 |  | + | 1 |
| **298** | TESQSLTLTDVE | 12 | Beta casein | 2 |  | 661.82 | 1321.62 | 43.62 | 135-146 |  | + | 1 |
| **299** | TLTDVENLHLPLPL | 14 | Beta casein | 2 |  | 787.94 | 1573.87 | 50.98 | 141-154 |  | + | 1 |
| **300** | TQTPVVVPPFLQPEVMGVSKVKEA | 24 | Beta casein | 3 |  | 860.81 | 2579.40 | 47.13 | 93-116 |  | + | 1 |
| **301** | TVMFPPQSVL | 10 | Beta casein | 2 |  | 559.80 | 1117.58 | 48.82 | 169-178 |  | + | 1 |
| **302** | VENLHLPLPL | 10 | Beta casein | 2 |  | 572.84 | 1143.67 | 48.31 | 145-154 |  | + | 1 |
| **303** | VKEAMAPK | 8 | Beta casein | 2 |  | 437.25 | 872.48 | 15.73 | 113-120 |  | + | 1 |
| **304** | VLSLSQSKVLPVPQ | 14 | Beta casein | 2 |  | 747.95 | 1493.88 | 43.80 | 177-190 |  | + | 1 |
| **305** | VMFPPQSVL | 9 | Beta casein | 2 |  | 509.28 | 1016.54 | 48.37 | 170-178 |  | + | 1 |
| **306** | VMFPPQSVLSLS | 12 | Beta casein | 2 |  | 652.85 | 1303.68 | 50.31 | 170-181 |  | + | 1 |
| **307** | VMFPPQSVLSLSQSKVLPVPQ | 21 | Beta casein | 2 |  | 1141.13 | 2280.26 | 48.82 | 170-190 |  | + | 1 |
| **308** | VMGVSKVK | 8 | Beta casein | 2 |  | 424.26 | 846.50 | 20.21 | 107-114 |  | + | 1 |
| **309** | VMGVSKVKEAM | 11 | Beta casein | 2 |  | 589.82 | 1177.62 | 30.97 | 107-117 |  | + | 1 |
| **310** | VPYPQRDMPIQA | 12 | Beta casein | 2 |  | 707.86 | 1413.71 | 40.74 | 193-204 |  | + | 1 |
| **311** | VVPPFLQP | 8 | Beta casein | 2 |  | 448.77 | 895.52 | 48.09 | 98-105 |  | + | 1 |
| **312** | VVPPFLQPE | 9 | Beta casein | 2 |  | 513.29 | 1024.56 | 47.52 | 98-106 |  | + | 1 |
| **313** | WMHQPHQPLPPT | 12 | Beta casein | 2 |  | 734.86 | 1467.71 | 33.06 | 158-169 |  | + | 1 |
| **314** | YQEPVLGPVR | 10 | Beta casein | 2 |  | 579.32 | 1156.62 | 39.09 | 208-217 |  | + | 1 |
| **315** | YQEPVLGPVRGPFPII | 16 | Beta casein | 2 |  | 891.50 | 1780.99 | 49.35 | 208-223 |  | + | 1 |
| **316** | DKALKALPM | 9 | Beta lactoglobulin | 2 |  | 493.79 | 985.56 | 37.13 | 137-145 |  | + | 1 |
| **317** | AIPPKKNQ | 8 | Kappa casein | 2 |  | 448.27 | 894.53 | 12.95 | 128-135 | + | + | 2 |
| **318** | AIPPKKNQD | 9 | Kappa casein | 2 |  | 505.79 | 1009.56 | 13.16 | 128-136 | + | + | 2 |
| **319** | ALINNQFLPYPYYAKPA | 17 | Kappa casein | 2 |  | 992.02 | 1982.03 | 46.44 | 70-86 | + | + | 2 |
| **320** | AVRSPAQIL | 9 | Kappa casein | 2 |  | 477.79 | 953.57 | 40.35 | 87-95 | + | + | 2 |
| **321** | AVRSPAQILQWQVL | 14 | Kappa casein | 2 |  | 804.96 | 1607.91 | 48.48 | 87-100 | + | + | 2 |
| **322** | DKIAKYIPI | 9 | Kappa casein | 2 |  | 530.82 | 1059.63 | 42.60 | 41-49 | + | + | 2 |
| **323** | DKIAKYIPIQY | 11 | Kappa casein | 2 |  | 676.38 | 1350.75 | 42.96 | 41-51 | + | + | 2 |
| **324** | DKTEIPTIN | 9 | Kappa casein | 2 |  | 515.77 | 1029.53 | 38.32 | 136-144 | + | + | 2 |
| **325** | DKTEIPTINTIA | 12 | Kappa casein | 2 |  | 658.36 | 1314.70 | 43.93 | 136-147 | + | + | 2 |
| **326** | EKDERFFSDKIA | 12 | Kappa casein | 2 |  | 742.87 | 1483.73 | 34.85 | 33-44 | + | + | 2 |
| **327** | FLPYPYYAKPA | 11 | Kappa casein | 2 |  | 665.35 | 1328.68 | 43.43 | 76-86 | + | + | 2 |
| **328** | FMAIPPKKN | 9 | Kappa casein | 2 |  | 523.30 | 1044.58 | 28.99 | 126-134 | + | + | 2 |
| **329** | FSDKIAKYIPI | 11 | Kappa casein | 2 |  | 647.87 | 1293.73 | 43.88 | 39-49 | + | + | 2 |
| **330** | INNQFLPYPY | 10 | Kappa casein | 2 |  | 634.82 | 1267.62 | 48.07 | 72-81 | + | + | 2 |
| **331** | INNQFLPYPYYAKPA | 15 | Kappa casein | 2 |  | 899.96 | 1797.91 | 44.54 | 72-86 | + | + | 2 |
| **332** | KIAKYIPIQY | 10 | Kappa casein | 2 |  | 618.87 | 1235.73 | 40.98 | 42-51 | + | + | 2 |
| **333** | KTEIPTIN | 8 | Kappa casein | 2 |  | 458.26 | 914.51 | 35.23 | 137-144 | + | + | 2 |
| **334** | KYIPIQY | 7 | Kappa casein | 2 |  | 462.76 | 923.51 | 40.36 | 45-51 | + | + | 2 |
| **335** | KYIPIQYVL | 9 | Kappa casein | 2 |  | 568.84 | 1135.66 | 45.59 | 45-53 | + | + | 2 |
| **336** | LINNQFLPYPYYAKPA | 16 | Kappa casein | 2 |  | 956.50 | 1910.99 | 45.54 | 71-86 | + | + | 2 |
| **337** | NYYQQKPVAL | 10 | Kappa casein | 2 |  | 612.32 | 1222.63 | 37.41 | 62-71 | + | + | 2 |
| **338** | QDKTEIPTIN | 10 | Kappa casein | 2 |  | 579.80 | 1157.59 | 36.79 | 135-144 | + | + | 2 |
| **339** | QFLPYPYYAKPA | 12 | Kappa casein | 2 |  | 729.38 | 1456.74 | 44.10 | 75-86 | + | + | 2 |
| **340** | QYVLSRYPSYGLN | 13 | Kappa casein | 2 |  | 780.40 | 1558.78 | 42.62 | 50-62 | + | + | 2 |
| **341** | SPPEINTVQVTSTAV | 15 | Kappa casein | 2 |  | 771.90 | 1541.79 | 43.71 | 176-190 | + | + | 2 |
| **342** | TIASGEPTSTPTTE | 14 | Kappa casein | 2 |  | 696.33 | 1390.65 | 31.59 | 145-158 | + | + | 2 |
| **343** | TIASGEPTSTPTTEAVE | 17 | Kappa casein | 2 |  | 845.90 | 1689.79 | 37.31 | 145-161 | + | + | 2 |
| **344** | TVQVTSTAV | 9 | Kappa casein | 1 |  | 905.49 | 904.49 | 35.51 | 182-190 | + |  | 1 |
| **345** | VIESPPEINTVQVT | 14 | Kappa casein | 2 |  | 763.41 | 1524.80 | 43.99 | 173-186 | + | + | 2 |
| **346** | VIESPPEINTVQVTSTAV | 18 | Kappa casein | 2 |  | 942.50 | 1882.99 | 45.18 | 173-190 | + | + | 2 |
| **347** | VLSRYPSYGL | 10 | Kappa casein | 2 |  | 577.81 | 1153.61 | 41.69 | 52-61 | + | + | 2 |
| **348** | YQQKPVALINN | 11 | Kappa casein | 2 |  | 644.36 | 1286.70 | 35.12 | 64-74 | + | + | 2 |
| **349** | YYQQKPV | 7 | Kappa casein | 2 |  | 463.24 | 924.47 | 22.82 | 63-69 | + | + | 2 |
| **350** | YYQQKPVA | 8 | Kappa casein | 2 |  | 498.76 | 995.51 | 23.07 | 63-70 | + | + | 2 |
| **351** | YYQQKPVAL | 9 | Kappa casein | 2 |  | 555.30 | 1108.59 | 35.16 | 63-71 | + | + | 2 |
| **352** | YYQQKPVALIN | 11 | Kappa casein | 2 |  | 668.87 | 1335.72 | 39.17 | 63-73 | + | + | 2 |
| **353** | YYQQKPVALINN | 12 | Kappa casein | 2 |  | 725.89 | 1449.76 | 38.00 | 63-74 | + | + | 2 |
| **354** | YYQQKPVALINNQFLPYPY | 19 | Kappa casein | 2 |  | 1180.11 | 2358.20 | 47.79 | 63-81 | + |  | 1 |
| **355** | YYQQKPVALINNQFLPYPYYAKPA | 24 | Kappa casein | 3 |  | 963.84 | 2888.49 | 45.39 | 63-86 | + |  | 1 |
| **356** | ALINNQFLPYPY | 12 | Kappa casein | 2 |  | 726.88 | 1451.74 | 50.42 | 70-81 |  | + | 1 |
| **357** | AQPTTMARHPHPH | 13 | Kappa casein | 2 |  | 740.86 | 1479.72 | 42.15 | 111-123 |  | + | 1 |
| **358** | AVESTVATL | 9 | Kappa casein | 2 |  | 445.75 | 889.48 | 42.03 | 159-167 |  | + | 1 |
| **359** | AVESTVATLE | 10 | Kappa casein | 1 |  | 1019.53 | 1018.52 | 41.97 | 159-168 |  | + | 1 |
| **360** | AVRSPAQ | 7 | Kappa casein | 2 |  | 364.71 | 727.40 | 15.67 | 87-93 |  | + | 1 |
| **361** | AVRSPAQILQWQ | 12 | Kappa casein | 2 |  | 698.89 | 1395.76 | 44.57 | 87-98 |  | + | 1 |
| **362** | DERFFSD | 7 | Kappa casein | 2 |  | 458.20 | 914.38 | 40.40 | 35-41 |  | + | 1 |
| **363** | DERFFSDKIA | 10 | Kappa casein | 2 |  | 614.30 | 1226.59 | 41.28 | 35-44 |  | + | 1 |
| **364** | DERFFSDKIAK | 11 | Kappa casein | 3 |  | 452.57 | 1354.69 | 33.87 | 35-45 |  | + | 1 |
| **365** | DKIAKYIP | 8 | Kappa casein | 2 |  | 474.28 | 946.55 | 36.60 | 41-48 |  | + | 1 |
| **366** | DKIAKYIPIQ | 10 | Kappa casein | 2 |  | 594.85 | 1187.69 | 41.12 | 41-50 |  | + | 1 |
| **367** | DKIAKYIPIQYVLS | 14 | Kappa casein | 2 |  | 825.98 | 1649.94 | 45.92 | 41-54 |  | + | 1 |
| **368** | EKDERFF | 7 | Kappa casein | 2 |  | 485.74 | 969.46 | 33.04 | 33-39 |  | + | 1 |
| **369** | EKDERFFS | 8 | Kappa casein | 2 |  | 529.25 | 1056.49 | 30.28 | 33-40 |  | + | 1 |
| **370** | EKDERFFSD | 9 | Kappa casein | 2 |  | 586.76 | 1171.51 | 31.48 | 33-41 |  | + | 1 |
| **371** | FFS | 3 | Kappa casein | 1 |  | 400.19 | 399.18 | 36.45 | 38-40 |  | + | 1 |
| **372** | IAKYIPI | 7 | Kappa casein | 2 |  | 409.26 | 816.51 | 42.26 | 43-49 |  | + | 1 |
| **373** | IAKYIPIQY | 9 | Kappa casein | 2 |  | 554.82 | 1107.63 | 42.83 | 43-51 |  | + | 1 |
| **374** | IAKYIPIQYVLS | 12 | Kappa casein | 2 |  | 704.42 | 1406.82 | 46.47 | 43-54 |  | + | 1 |
| **375** | KDERFFS | 7 | Kappa casein | 2 |  | 464.73 | 927.45 | 29.34 | 34-40 |  | + | 1 |
| **376** | KDERFFSD | 8 | Kappa casein | 2 |  | 522.24 | 1042.47 | 30.74 | 34-41 |  | + | 1 |
| **377** | KDERFFSDKIA | 11 | Kappa casein | 2 |  | 678.35 | 1354.69 | 33.87 | 34-44 |  | + | 1 |
| **378** | KIAKYIP | 7 | Kappa casein | 2 |  | 416.77 | 831.52 | 29.54 | 42-48 |  | + | 1 |
| **379** | KIAKYIPI | 8 | Kappa casein | 2 |  | 473.31 | 944.61 | 38.92 | 42-49 |  | + | 1 |
| **380** | KIAKYIPIQ | 9 | Kappa casein | 2 |  | 537.34 | 1072.66 | 35.46 | 42-50 |  | + | 1 |
| **381** | KYIPIQ | 6 | Kappa casein | 2 |  | 381.23 | 760.45 | 32.49 | 45-50 |  | + | 1 |
| **382** | KYIPIQYV | 8 | Kappa casein | 2 |  | 512.30 | 1022.58 | 42.54 | 45-52 |  | + | 1 |
| **383** | KYIPIQYVLS | 10 | Kappa casein | 2 |  | 612.36 | 1222.70 | 44.75 | 45-54 |  | + | 1 |
| **384** | LINNQFLPYPY | 11 | Kappa casein | 2 |  | 691.36 | 1380.71 | 49.44 | 71-81 |  | + | 1 |
| **385** | LPF | 3 | Kappa casein | 1 |  | 376.22 | 375.22 | 33.86 | 16-18 |  | + | 1 |
| **386** | LSRYPSYGLN | 10 | Kappa casein | 2 |  | 585.30 | 1168.59 | 36.66 | 53-62 |  | + | 1 |
| **387** | MAIPPKKN | 8 | Kappa casein | 2 |  | 449.76 | 897.51 | 18.53 | 127-134 |  | + | 1 |
| **388** | NNQFLPYPYYAKPA | 14 | Kappa casein | 2 |  | 843.42 | 1684.82 | 43.97 | 73-86 |  | + | 1 |
| **389** | NYYQQKPVALINN | 13 | Kappa casein | 2 |  | 782.91 | 1563.80 | 39.80 | 62-74 |  | + | 1 |
| **390** | QDKTEIPT | 8 | Kappa casein | 2 |  | 466.24 | 930.47 | 26.42 | 135-142 |  | + | 1 |
| **391** | QFLPYPY | 7 | Kappa casein | 2 |  | 464.23 | 926.45 | 47.74 | 75-81 |  | + | 1 |
| **392** | QFLPYPYYAK | 10 | Kappa casein | 2 |  | 645.33 | 1288.65 | 43.92 | 75-84 |  | + | 1 |
| **393** | QKPVALINN | 9 | Kappa casein | 2 |  | 498.80 | 995.58 | 31.65 | 66-74 |  | + | 1 |
| **394** | QQKPVALINN | 10 | Kappa casein | 2 |  | 562.82 | 1123.63 | 32.10 | 65-74 |  | + | 1 |
| **395** | QWQVLSNTVPA | 11 | Kappa casein | 2 |  | 621.83 | 1241.64 | 45.63 | 96-106 |  | + | 1 |
| **396** | QWQVLSNTVPAKS | 13 | Kappa casein | 2 |  | 729.39 | 1456.77 | 42.03 | 96-108 |  | + | 1 |
| **397** | QYV | 3 | Kappa casein | 1 |  | 409.21 | 408.20 | 28.73 | 50-52 |  | + | 1 |
| **398** | QYVLSRYPSYGL | 12 | Kappa casein | 2 |  | 723.37 | 1444.74 | 43.68 | 50-61 |  | + | 1 |
| **399** | SDKIAKYIPI | 10 | Kappa casein | 2 |  | 574.34 | 1146.66 | 42.09 | 40-49 |  | + | 1 |
| **400** | SDKIAKYIPIQY | 12 | Kappa casein | 2 |  | 719.90 | 1437.79 | 42.48 | 40-51 |  | + | 1 |
| **401** | SPPEINTVQVT | 11 | Kappa casein | 2 |  | 592.81 | 1183.61 | 41.74 | 176-186 |  | + | 1 |
| **402** | SRYPSYGLN | 9 | Kappa casein | 2 |  | 528.76 | 1055.50 | 33.60 | 54-62 |  | + | 1 |
| **403** | TIASGEPTSTPTTEA | 15 | Kappa casein | 2 |  | 731.85 | 1461.68 | 32.83 | 145-159 |  | + | 1 |
| **404** | VIESPPEIN | 9 | Kappa casein | 2 |  | 499.26 | 996.51 | 40.22 | 173-181 |  | + | 1 |
| **405** | VIESPPEINTVQ | 12 | Kappa casein | 2 |  | 663.35 | 1324.69 | 42.31 | 173-184 |  | + | 1 |
| **406** | VLSRYPS | 7 | Kappa casein | 2 |  | 411.23 | 820.44 | 25.38 | 52-58 |  | + | 1 |
| **407** | VLSRYPSYGLN | 11 | Kappa casein | 2 |  | 634.84 | 1267.66 | 39.04 | 52-62 |  | + | 1 |
| **408** | YAKPAAVRSPA | 11 | Kappa casein | 2 |  | 565.82 | 1129.62 | 22.61 | 82-92 |  | + | 1 |
| **409** | YAKPAAVRSPAQIL | 14 | Kappa casein | 2 |  | 742.93 | 1483.85 | 38.68 | 82-95 |  | + | 1 |
| **410** | YQQKPVA | 7 | Kappa casein | 2 |  | 417.23 | 832.44 | 18.12 | 64-70 |  | + | 1 |
| **411** | YQQKPVAL | 8 | Kappa casein | 2 |  | 473.77 | 945.53 | 31.31 | 64-71 |  | + | 1 |
| **412** | YQQKPVALIN | 10 | Kappa casein | 2 |  | 587.33 | 1172.66 | 36.15 | 64-73 |  | + | 1 |
| **413** | YVLSRYPSYGL | 11 | Kappa casein | 2 |  | 659.35 | 1316.68 | 43.45 | 51-61 |  | + | 1 |
| **414** | YVLSRYPSYGLN | 12 | Kappa casein | 2 |  | 716.37 | 1430.72 | 42.31 | 51-62 |  | + | 1 |
| **415** | YYA | 3 | Kappa casein | 1 |  | 416.18 | 415.17 | 23.37 | 81-83 |  | + | 1 |
| **416** | YYQQKPVALI | 10 | Kappa casein | 2 |  | 611.85 | 1221.68 | 42.26 | 63-72 |  | + | 1 |
| **417** | YYQQKPVALINNQ | 13 | Kappa casein | 2 |  | 789.92 | 1577.82 | 37.79 | 63-75 |  | + | 1 |
| **418** | LGEYGFQNALIVR | 13 | Serum albumin | 2 |  | 740.40 | 1478.79 | 45.07 | 421-433 | + |  | 1 |
| **419** | RHPEYAVSVLLR | 12 | Serum albumin | 3 |  | 480.61 | 1438.80 | 39.71 | 360-371 | + | + | 2 |
| **420** | FKDLGEEHFK | 10 | Serum albumin | 3 |  | 417.21 | 1248.61 | 28.68 | 35-44 |  | + | 1 |
| **421** | KYL | 3 | Serum albumin | 1 |  | 423.26 | 422.25 | 33.44 | 1609-1611 |  | + | 1 |
| **422** | RCC | 3 | Serum albumin | 1 |  | 495.18 | 494.17 | 29.26 | 459-461 |  | + | 1 |

**Supplementary Table 2.** Milk peptides identified throughout the 28 days of storage period using the *Lb. helveticus* 9B5.

|  | **Sequence** | **Length** | **Leading proteins** | **Charge** | **m/z** | **Mass** | **Retention time** | **Sequence** | **Day 1** | **Day 28** | **Incidence** |
| --- | --- | --- | --- | --- | --- | --- | --- | --- | --- | --- | --- |
| **1** | APSFSDIPNPIGSENSEKTTMPLW | 24 | Alpha S1 Casein | 2 | 1309.63 | 2617.24 | 48.38 | 191-214 | + | + | 2 |
| **2** | DIPNPIGSENSEKTTMPLW | 19 | Alpha S1 Casein | 2 | 1065.01 | 2128.01 | 47.77 | 196-214 | + | + | 2 |
| **3** | DVPSERYLGYLEQL | 14 | Alpha S1 Casein | 2 | 841.43 | 1680.84 | 48.67 | 100-113 | + |  | 1 |
| **4** | EVLNENLLR | 9 | Alpha S1 Casein | 2 | 550.31 | 1098.60 | 41.77 | 29-37 | + | + | 2 |
| **5** | EVLNENLLRF | 10 | Alpha S1 Casein | 2 | 623.84 | 1245.67 | 46.14 | 29-38 | + | + | 2 |
| **6** | EVLNENLLRFF | 11 | Alpha S1 Casein | 2 | 697.38 | 1392.74 | 50.30 | 29-39 | + | + | 2 |
| **7** | FSDIPNPIGSENSEKTTMP | 19 | Alpha S1 Casein | 2 | 1032.48 | 2062.95 | 43.26 | 194-212 | + | + | 2 |
| **8** | FSDIPNPIGSENSEKTTMPLW | 21 | Alpha S1 Casein | 2 | 1182.06 | 2362.12 | 47.48 | 194-214 | + | + | 2 |
| **9** | GKEKVNEL | 8 | Alpha S1 Casein | 2 | 458.76 | 915.50 | 18.36 | 48-55 | + | + | 2 |
| **10** | GLPQEVLNENLLR | 13 | Alpha S1 Casein | 2 | 747.92 | 1493.82 | 45.16 | 25-37 | + | + | 2 |
| **11** | GLPQEVLNENLLRF | 14 | Alpha S1 Casein | 2 | 821.45 | 1640.89 | 48.84 | 25-38 | + |  | 1 |
| **12** | GLPQEVLNENLLRFF | 15 | Alpha S1 Casein | 2 | 894.99 | 1787.96 | 52.51 | 25-39 | + |  | 1 |
| **13** | IPNPIGSENSEKTTMPLW | 18 | Alpha S1 Casein | 2 | 1007.50 | 2012.99 | 45.98 | 197-214 | + | + | 2 |
| **14** | NENLLRF | 7 | Alpha S1 Casein | 2 | 453.25 | 904.48 | 43.37 | 32-38 | + | + | 2 |
| **15** | NENLLRFF | 8 | Alpha S1 Casein | 2 | 526.78 | 1051.55 | 47.59 | 32-39 | + | + | 2 |
| **16** | NENLLRFFVAPFPE | 14 | Alpha S1 Casein | 2 | 846.94 | 1691.87 | 51.73 | 32-45 | + |  | 1 |
| **17** | QGLPQEVLNENLLR | 14 | Alpha S1 Casein | 2 | 811.95 | 1621.88 | 45.01 | 24-37 | + |  | 1 |
| **18** | QKEPMIGVNQEL | 12 | Alpha S1 Casein | 2 | 693.36 | 1384.70 | 41.46 | 146-157 | + | + | 2 |
| **19** | SDIPNPIGSENSEKTTMPLW | 20 | Alpha S1 Casein | 2 | 1108.53 | 2215.05 | 46.43 | 195-214 | + | + | 2 |
| **20** | SFSDIPNPIGSENSEKTTMP | 20 | Alpha S1 Casein | 2 | 1076.00 | 2149.98 | 43.79 | 193-212 | + |  | 1 |
| **21** | SFSDIPNPIGSENSEKTTMPLW | 22 | Alpha S1 Casein | 2 | 1225.58 | 2449.15 | 47.99 | 193-214 | + | + | 2 |
| **22** | SISSSEEIVPNSVEQK | 16 | Alpha S1 Casein | 2 | 866.93 | 1731.85 | 37.30 | 79-94 | + |  | 1 |
| **23** | TDAPSFSDIPNPIGSENSEKTTMP | 24 | Alpha S1 Casein | 2 | 1268.08 | 2534.15 | 45.05 | 189-212 | + |  | 1 |
| **24** | TDAPSFSDIPNPIGSENSEKTTMPLW | 26 | Alpha S1 Casein | 2 | 1417.66 | 2833.31 | 48.89 | 189-214 | + |  | 1 |
| **25** | VLNENLLRF | 9 | Alpha S1 Casein | 2 | 559.32 | 1116.63 | 45.24 | 30-38 | + | + | 2 |
| **26** | VLNENLLRFF | 10 | Alpha S1 Casein | 2 | 632.86 | 1263.70 | 49.43 | 30-39 | + | + | 2 |
| **27** | YLG | 3 | Alpha S1 Casein | 1 | 352.19 | 351.18 | 24.15 | 106-108 | + |  | 1 |
| **28** | YQL | 3 | Alpha S1 Casein | 1 | 423.22 | 422.22 | 28.55 | 169-171 | + |  | 1 |
| **29** | YVP | 3 | Alpha S1 Casein | 1 | 378.20 | 377.20 | 29.47 | 181-183 | + | + | 2 |
| **30** | AMEDIKQM | 8 | Alpha S1 Casein | 2 | 483.23 | 964.44 | 32.10 | 68-75 |  |  | 1 |
| **31** | APSFSDIPNPIGSENSEK | 18 | Alpha S1 Casein | 2 | 944.95 | 1887.89 | 43.57 | 191-208 |  | + | 1 |
| **32** | AYF | 3 | Alpha S1 Casein | 1 | 400.19 | 399.18 | 38.68 | 158-160 |  | + | 1 |
| **33** | DIKQMEAE | 8 | Alpha S1 Casein | 2 | 482.23 | 962.44 | 28.89 | 71-78 |  | + | 1 |
| **34** | DQAMEDIKQ | 9 | Alpha S1 Casein | 2 | 539.25 | 1076.48 | 27.69 | 66-74 |  | + | 1 |
| **35** | DQAMEDIKQM | 10 | Alpha S1 Casein | 2 | 604.77 | 1207.52 | 38.16 | 66-75 |  | + | 1 |
| **36** | DVPSERY | 7 | Alpha S1 Casein | 2 | 433.21 | 864.40 | 26.16 | 100-106 |  | + | 1 |
| **37** | DVPSERYL | 8 | Alpha S1 Casein | 2 | 489.75 | 977.48 | 38.27 | 100-107 |  | + | 1 |
| **38** | DVPSERYLG | 9 | Alpha S1 Casein | 2 | 518.26 | 1034.50 | 35.77 | 100-108 |  | + | 1 |
| **39** | EDIKQME | 7 | Alpha S1 Casein | 2 | 446.71 | 891.40 | 25.72 | 70-76 |  | + | 1 |
| **40** | EDIKQMEAE | 9 | Alpha S1 Casein | 2 | 546.75 | 1091.48 | 28.76 | 70-78 |  | + | 1 |
| **41** | ENLLRF | 6 | Alpha S1 Casein | 2 | 396.22 | 790.43 | 43.61 | 33-38 |  | + | 1 |
| **42** | EQLLRLK | 7 | Alpha S1 Casein | 2 | 450.29 | 898.56 | 32.85 | 111-117 |  | + | 1 |
| **43** | EQLLRLKK | 8 | Alpha S1 Casein | 2 | 514.33 | 1026.65 | 25.47 | 111-118 |  | + | 1 |
| **44** | FFVAPFPE | 8 | Alpha S1 Casein | 1 | 953.48 | 952.47 | 49.89 | 38-45 |  | + | 1 |
| **45** | GKEKVNELS | 9 | Alpha S1 Casein | 2 | 502.27 | 1002.53 | 17.58 | 48-56 |  | + | 1 |
| **46** | GLPQEVLNEN | 10 | Alpha S1 Casein | 2 | 556.78 | 1111.55 | 42.90 | 25-34 |  | + | 1 |
| **47** | GLPQEVLNENLL | 12 | Alpha S1 Casein | 2 | 669.87 | 1337.72 | 49.06 | 25-36 |  | + | 1 |
| **48** | GSENSEKTTMPLW | 13 | Alpha S1 Casein | 2 | 740.34 | 1478.67 | 43.69 | 202-214 |  | + | 1 |
| **49** | GYLEQLLRLK | 10 | Alpha S1 Casein | 2 | 616.87 | 1231.73 | 45.35 | 108-117 |  | + | 1 |
| **50** | HIQKEDVPS | 9 | Alpha S1 Casein | 2 | 526.77 | 1051.53 | 18.68 | 95-103 |  | + | 1 |
| **51** | HIQKEDVPSER | 11 | Alpha S1 Casein | 3 | 446.57 | 1336.67 | 17.82 | 95-105 |  | + | 1 |
| **52** | IKQMEAE | 7 | Alpha S1 Casein | 2 | 424.71 | 847.41 | 20.00 | 72-78 |  | + | 1 |
| **53** | IVPNSAEER | 9 | Alpha S1 Casein | 2 | 507.76 | 1013.51 | 21.42 | 126-134 |  | + | 1 |
| **54** | IVPNSAEERLH | 11 | Alpha S1 Casein | 3 | 422.23 | 1263.66 | 26.36 | 126-136 |  | + | 1 |
| **55** | KEDVPSER | 8 | Alpha S1 Casein | 2 | 480.24 | 958.47 | 15.85 | 98-105 |  | + | 1 |
| **56** | KEDVPSERY | 9 | Alpha S1 Casein | 2 | 561.77 | 1121.54 | 22.32 | 98-106 |  | + | 1 |
| **57** | KEPMIGVNQEL | 11 | Alpha S1 Casein | 2 | 629.33 | 1256.64 | 41.56 | 147-157 |  | + | 1 |
| **58** | KHQGLPQE | 8 | Alpha S1 Casein | 2 | 468.75 | 935.48 | 17.99 | 22-29 |  | + | 1 |
| **59** | KHQGLPQEVL | 10 | Alpha S1 Casein | 2 | 574.82 | 1147.63 | 33.40 | 22-31 |  | + | 1 |
| **60** | KVPQLEIVPN | 10 | Alpha S1 Casein | 2 | 568.84 | 1135.66 | 42.52 | 120-129 |  | + | 1 |
| **61** | KYKVPQLE | 8 | Alpha S1 Casein | 2 | 502.79 | 1003.57 | 30.65 | 118-125 |  | + | 1 |
| **62** | LNENLLR | 7 | Alpha S1 Casein | 2 | 436.25 | 870.49 | 34.90 | 31-37 |  | + | 1 |
| **63** | LNENLLRF | 8 | Alpha S1 Casein | 2 | 509.79 | 1017.56 | 44.63 | 31-38 |  | + | 1 |
| **64** | MEDIKQM | 7 | Alpha S1 Casein | 2 | 447.71 | 893.40 | 29.82 | 69-75 |  | + | 1 |
| **65** | MEDIKQME | 8 | Alpha S1 Casein | 2 | 512.23 | 1022.44 | 31.25 | 69-76 |  | + | 1 |
| **66** | MEDIKQMEAE | 10 | Alpha S1 Casein | 2 | 612.27 | 1222.52 | 33.76 | 69-78 |  | + | 1 |
| **67** | NENLLR | 6 | Alpha S1 Casein | 2 | 379.71 | 757.41 | 27.17 | 32-37 |  | + | 1 |
| **68** | NSEKTTMPLW | 10 | Alpha S1 Casein | 2 | 603.79 | 1205.58 | 43.56 | 205-214 |  | + | 1 |
| **69** | QAMEDIKQ | 8 | Alpha S1 Casein | 2 | 481.73 | 961.45 | 23.99 | 67-74 |  | + | 1 |
| **70** | QAMEDIKQM | 9 | Alpha S1 Casein | 2 | 547.25 | 1092.49 | 33.10 | 67-75 |  | + | 1 |
| **71** | QAMEDIKQME | 10 | Alpha S1 Casein | 2 | 611.78 | 1221.54 | 34.31 | 67-76 |  | + | 1 |
| **72** | QAMEDIKQMEAE | 12 | Alpha S1 Casein | 2 | 711.82 | 1421.62 | 36.70 | 67-78 |  | + | 1 |
| **73** | QKEPMIGVN | 9 | Alpha S1 Casein | 2 | 508.27 | 1014.52 | 31.77 | 146-154 |  | + | 1 |
| **74** | QKEPMIGVNQ | 10 | Alpha S1 Casein | 2 | 572.29 | 1142.58 | 31.67 | 146-155 |  | + | 1 |
| **75** | QKEPMIGVNQE | 11 | Alpha S1 Casein | 2 | 636.82 | 1271.62 | 32.04 | 146-156 |  | + | 1 |
| **76** | RFFVAPFPE | 9 | Alpha S1 Casein | 2 | 555.29 | 1108.57 | 45.75 | 37-45 |  | + | 1 |
| **77** | SEKTTMPLW | 9 | Alpha S1 Casein | 2 | 546.77 | 1091.53 | 43.67 | 206-214 |  | + | 1 |
| **78** | SFSDIPNPIGSENSEK | 16 | Alpha S1 Casein | 2 | 860.90 | 1719.80 | 42.52 | 193-208 |  | + | 1 |
| **79** | SKDIGSESTED | 11 | Alpha S1 Casein | 2 | 584.25 | 1166.49 | 18.23 | 56-66 |  | + | 1 |
| **80** | SMKEGIHAQ | 9 | Alpha S1 Casein | 2 | 500.75 | 999.48 | 17.40 | 137-145 |  | + | 1 |
| **81** | SVEQKHIQ | 8 | Alpha S1 Casein | 2 | 484.76 | 967.51 | 14.44 | 90-97 |  | + | 1 |
| **82** | VAPFPEVF | 8 | Alpha S1 Casein | 2 | 453.24 | 904.47 | 50.12 | 40-47 |  | + | 1 |
| **83** | VAPFPEVFGK | 10 | Alpha S1 Casein | 2 | 545.80 | 1089.59 | 44.75 | 40-49 |  | + | 1 |
| **84** | VFGKEKVN | 8 | Alpha S1 Casein | 2 | 460.76 | 919.51 | 18.99 | 46-53 |  | + | 1 |
| **85** | VFGKEKVNE | 9 | Alpha S1 Casein | 2 | 525.28 | 1048.56 | 20.73 | 46-54 |  | + | 1 |
| **86** | VFGKEKVNEL | 10 | Alpha S1 Casein | 2 | 581.83 | 1161.64 | 30.68 | 46-55 |  | + | 1 |
| **87** | VFGKEKVNELS | 11 | Alpha S1 Casein | 2 | 625.34 | 1248.67 | 28.89 | 46-56 |  | + | 1 |
| **88** | VLNENLLR | 8 | Alpha S1 Casein | 2 | 485.79 | 969.56 | 38.61 | 30-37 |  | + | 1 |
| **89** | VPQLEIVPN | 9 | Alpha S1 Casein | 2 | 504.79 | 1007.57 | 45.28 | 121-129 |  | + | 1 |
| **90** | YKVPQLE | 7 | Alpha S1 Casein | 2 | 438.74 | 875.48 | 37.06 | 119-125 |  | + | 1 |
| **91** | LYQGPIVLNPWDQVKR | 16 | Alpha S2 Casein | 2 | 963.53 | 1925.05 | 44.42 | 114-129 | + | + | 2 |
| **92** | LYQGPIVLNPWDQVKRN | 17 | Alpha S2 Casein | 3 | 680.71 | 2039.10 | 44.36 | 114-130 | + | + | 2 |
| **93** | QYLYQGPIVLNPWDQVKR | 18 | Alpha S2 Casein | 3 | 739.73 | 2216.17 | 45.29 | 112-129 | + |  | 1 |
| **94** | QYLYQGPIVLNPWDQVKRN | 19 | Alpha S2 Casein | 3 | 777.75 | 2330.22 | 45.29 | 112-130 | + |  | 1 |
| **95** | YQGPIVLNPWDQVK | 14 | Alpha S2 Casein | 2 | 828.94 | 1655.87 | 45.66 | 115-128 | + | + | 2 |
| **96** | YQGPIVLNPWDQVKRN | 16 | Alpha S2 Casein | 3 | 643.01 | 1926.01 | 43.60 | 115-130 | + |  | 1 |
| **97** | YVR | 3 | Alpha S2 Casein | 1 | 437.25 | 436.24 | 60.43 | 218-220 | + |  | 1 |
| **98** | AINPSKEN | 8 | Alpha S2 Casein | 2 | 436.73 | 871.44 | 16.49 | 42-49 |  | + | 1 |
| **99** | ALPQYLKT | 8 | Alpha S2 Casein | 2 | 467.27 | 932.53 | 38.27 | 190-197 |  | + | 1 |
| **100** | EKNMAINPSKE | 11 | Alpha S2 Casein | 2 | 630.82 | 1259.62 | 22.05 | 38-48 |  | + | 1 |
| **101** | EVVRNANEE | 9 | Alpha S2 Casein | 2 | 530.26 | 1058.50 | 17.42 | 57-65 |  | + | 1 |
| **102** | FLKKISQ | 7 | Alpha S2 Casein | 2 | 432.27 | 862.53 | 22.72 | 178-184 |  | + | 1 |
| **103** | IISQETYK | 8 | Alpha S2 Casein | 2 | 491.27 | 980.52 | 25.07 | 29-36 |  | + | 1 |
| **104** | IISQETYKQEK | 11 | Alpha S2 Casein | 2 | 683.86 | 1365.71 | 21.90 | 29-39 |  | + | 1 |
| **105** | LNREQL | 6 | Alpha S2 Casein | 2 | 386.72 | 771.42 | 24.57 | 138-143 |  | + | 1 |
| **106** | LYQGPIVLNPWDQVK | 15 | Alpha S2 Casein | 2 | 885.48 | 1768.95 | 46.62 | 114-128 |  | + | 1 |
| **107** | NMAINPSKE | 9 | Alpha S2 Casein | 2 | 502.25 | 1002.48 | 26.84 | 40-48 |  | + | 1 |
| **108** | NSKKTVDME | 9 | Alpha S2 Casein | 2 | 526.26 | 1050.50 | 15.63 | 149-157 |  | + | 1 |
| **109** | STEVFTKK | 8 | Alpha S2 Casein | 2 | 470.26 | 938.51 | 18.67 | 158-165 |  | + | 1 |
| **110** | TEEEKNRLN | 9 | Alpha S2 Casein | 2 | 566.78 | 1131.55 | 14.70 | 169-177 |  | + | 1 |
| **111** | TKVIPYVR | 8 | Alpha S2 Casein | 2 | 488.30 | 974.59 | 30.29 | 213-220 |  | + | 1 |
| **112** | TKVIPYVRYL | 10 | Alpha S2 Casein | 2 | 626.38 | 1250.74 | 42.36 | 213-222 |  | + | 1 |
| **113** | TYKQEKNM | 8 | Alpha S2 Casein | 2 | 521.26 | 1040.50 | 14.48 | 34-41 |  | + | 1 |
| **114** | TYKQEKNMA | 9 | Alpha S2 Casein | 2 | 556.77 | 1111.53 | 15.53 | 34-42 |  | + | 1 |
| **115** | VFTKKTKLTEE | 11 | Alpha S2 Casein | 3 | 441.92 | 1322.74 | 20.53 | 161-171 |  | + | 1 |
| **116** | VVRNANEE | 8 | Alpha S2 Casein | 2 | 465.74 | 929.46 | 14.61 | 58-65 |  | + | 1 |
| **117** | YQGPIVLNPWDQVKR | 15 | Alpha S2 Casein | 3 | 605.00 | 1811.97 | 43.72 | 115-129 |  | + | 1 |
| **118** | AFLLYQEPVLGPVRGPFPIIV | 21 | Beta Casein | 2 | 1163.17 | 2324.33 | 53.42 | 204-224 | + |  | 1 |
| **119** | AQTQSLVYPFPGPIPN | 16 | Beta Casein | 2 | 864.95 | 1727.89 | 48.72 | 68-83 | + | + | 2 |
| **120** | DELQDKIHPF | 10 | Beta Casein | 2 | 621.31 | 1240.61 | 41.61 | 58-67 | + | + | 2 |
| **121** | DELQDKIHPFAQTQ | 14 | Beta Casein | 2 | 835.41 | 1668.81 | 40.70 | 58-71 | + | + | 2 |
| **122** | DELQDKIHPFAQTQS | 15 | Beta Casein | 2 | 878.93 | 1755.84 | 40.66 | 58-72 | + |  | 1 |
| **123** | DELQDKIHPFAQTQSL | 16 | Beta Casein | 3 | 623.98 | 1868.93 | 42.84 | 58-73 | + |  | 1 |
| **124** | DKIHPF | 6 | Beta Casein | 2 | 378.71 | 755.40 | 28.54 | 62-67 | + | + | 2 |
| **125** | DKIHPFAQTQ | 10 | Beta Casein | 2 | 592.81 | 1183.60 | 27.93 | 62-71 | + | + | 2 |
| **126** | DKIHPFAQTQSL | 12 | Beta Casein | 2 | 692.86 | 1383.71 | 37.30 | 62-73 | + | + | 2 |
| **127** | DVENLHLPLPL | 11 | Beta Casein | 2 | 630.35 | 1258.69 | 50.48 | 144-154 | + | + | 2 |
| **128** | DVENLHLPLPLL | 12 | Beta Casein | 2 | 686.90 | 1371.78 | 52.89 | 144-155 | + | + | 2 |
| **129** | DVENLHLPLPLLQ | 13 | Beta Casein | 2 | 750.92 | 1499.83 | 52.20 | 144-156 | + | + | 2 |
| **130** | DVENLHLPLPLLQS | 14 | Beta Casein | 2 | 794.44 | 1586.87 | 51.32 | 144-157 | + | + | 2 |
| **131** | DVENLHLPLPLLQSW | 15 | Beta Casein | 2 | 887.48 | 1772.95 | 54.53 | 144-158 | + |  | 1 |
| **132** | EDELQDKIHPF | 11 | Beta Casein | 2 | 685.83 | 1369.65 | 41.44 | 57-67 | + | + | 2 |
| **133** | EDELQDKIHPFAQTQ | 15 | Beta Casein | 2 | 899.93 | 1797.85 | 40.49 | 57-71 | + |  | 1 |
| **134** | EEQQQTEDELQDKIHPF | 17 | Beta Casein | 3 | 705.33 | 2112.96 | 41.83 | 51-67 | + | + | 2 |
| **135** | ELQDKIHP | 8 | Beta Casein | 2 | 490.26 | 978.51 | 25.30 | 59-66 | + | + | 2 |
| **136** | ELQDKIHPF | 9 | Beta Casein | 2 | 563.80 | 1125.58 | 36.42 | 59-67 | + | + | 2 |
| **137** | ELQDKIHPFAQT | 12 | Beta Casein | 2 | 713.87 | 1425.73 | 37.03 | 59-70 | + |  | 1 |
| **138** | ELQDKIHPFAQTQ | 13 | Beta Casein | 2 | 777.90 | 1553.78 | 36.10 | 59-71 | + |  | 1 |
| **139** | ELQDKIHPFAQTQSL | 15 | Beta Casein | 2 | 877.96 | 1753.90 | 41.66 | 59-73 | + |  | 1 |
| **140** | EMPFPKYPVEPF | 12 | Beta Casein | 2 | 740.86 | 1479.71 | 46.73 | 123-134 | + | + | 2 |
| **141** | EPVLGPVRGPFPIIV | 15 | Beta Casein | 2 | 795.47 | 1588.93 | 49.79 | 210-224 | + | + | 2 |
| **142** | EQQQTEDELQ | 10 | Beta Casein | 2 | 624.27 | 1246.53 | 27.43 | 52-61 | + | + | 2 |
| **143** | EQQQTEDELQDK | 12 | Beta Casein | 2 | 745.83 | 1489.65 | 23.13 | 52-63 | + | + | 2 |
| **144** | EQQQTEDELQDKIHP | 15 | Beta Casein | 3 | 613.29 | 1836.85 | 34.18 | 52-66 | + |  | 1 |
| **145** | EQQQTEDELQDKIHPF | 16 | Beta Casein | 3 | 662.31 | 1983.92 | 40.02 | 52-67 | + | + | 2 |
| **146** | EQQQTEDELQDKIHPFAQT | 19 | Beta Casein | 3 | 762.36 | 2284.06 | 41.29 | 52-70 | + |  | 1 |
| **147** | EQQQTEDELQDKIHPFAQTQ | 20 | Beta Casein | 3 | 805.05 | 2412.12 | 41.04 | 52-71 | + | + | 2 |
| **148** | EQQQTEDELQDKIHPFAQTQS | 21 | Beta Casein | 3 | 834.06 | 2499.15 | 40.89 | 52-72 | + |  | 1 |
| **149** | EQQQTEDELQDKIHPFAQTQSL | 22 | Beta Casein | 3 | 871.75 | 2612.24 | 42.62 | 52-73 | + |  | 1 |
| **150** | FLLYQEPVLGPVRGPFPIIV | 20 | Beta Casein | 2 | 1127.65 | 2253.29 | 53.21 | 205-224 | + |  | 1 |
| **151** | FQSEEQQQTEDELQDK | 16 | Beta Casein | 2 | 991.43 | 1980.85 | 31.61 | 48-63 | + |  | 1 |
| **152** | GPVRGPFPIIV | 11 | Beta Casein | 2 | 576.35 | 1150.69 | 46.79 | 214-224 | + | + | 2 |
| **153** | GVSKVKEA | 8 | Beta Casein | 2 | 409.24 | 816.47 | 13.62 | 109-116 | + | + | 2 |
| **154** | GVSKVKEAMAPK | 12 | Beta Casein | 2 | 622.86 | 1243.70 | 19.10 | 109-120 | + | + | 2 |
| **155** | HKEMPFPKYPVEPF | 14 | Beta Casein | 3 | 582.63 | 1744.86 | 42.42 | 121-134 | + | + | 2 |
| **156** | HKEMPFPKYPVEPFTES | 17 | Beta Casein | 3 | 688.34 | 2061.99 | 42.27 | 121-137 | + |  | 1 |
| **157** | HQPHQPLPPT | 10 | Beta Casein | 2 | 576.30 | 1150.59 | 22.53 | 160-169 | + | + | 2 |
| **158** | HQPHQPLPPTVMFPPQ | 16 | Beta Casein | 3 | 617.65 | 1849.93 | 42.40 | 160-175 | + | + | 2 |
| **159** | HQPHQPLPPTVMFPPQS | 17 | Beta Casein | 3 | 646.66 | 1936.96 | 42.38 | 160-176 | + |  | 1 |
| **160** | HQPHQPLPPTVMFPPQSVL | 19 | Beta Casein | 3 | 717.38 | 2149.11 | 45.16 | 160-178 | + | + | 2 |
| **161** | IPPLTQTPVVVPPFLQPEVM | 20 | Beta Casein | 2 | 1101.62 | 2201.22 | 54.93 | 89-108 | + |  | 1 |
| **162** | KAVPYPQ | 7 | Beta Casein | 2 | 401.73 | 801.44 | 25.25 | 191-197 | + | + | 2 |
| **163** | KAVPYPQRDMPIQ | 13 | Beta Casein | 3 | 514.94 | 1541.80 | 33.44 | 191-203 | + | + | 2 |
| **164** | KAVPYPQRDMPIQA | 14 | Beta Casein | 2 | 807.43 | 1612.84 | 35.40 | 191-204 | + | + | 2 |
| **165** | KAVPYPQRDMPIQAF | 15 | Beta Casein | 3 | 587.64 | 1759.91 | 42.16 | 191-205 | + |  | 1 |
| **166** | KVLPVPQKAVPYPQ | 14 | Beta Casein | 3 | 521.98 | 1562.92 | 37.04 | 184-197 | + | + | 2 |
| **167** | LHLPLPLLQ | 9 | Beta Casein | 2 | 522.33 | 1042.65 | 49.06 | 148-156 | + | + | 2 |
| **168** | LLYQEPVLGPVRGPFP | 16 | Beta Casein | 2 | 891.50 | 1780.99 | 47.14 | 206-221 | + | + | 2 |
| **169** | LLYQEPVLGPVRGPFPI | 17 | Beta Casein | 2 | 948.04 | 1894.07 | 49.32 | 206-222 | + |  | 1 |
| **170** | LLYQEPVLGPVRGPFPII | 18 | Beta Casein | 2 | 1004.59 | 2007.16 | 50.76 | 206-223 | + |  | 1 |
| **171** | LLYQEPVLGPVRGPFPIIV | 19 | Beta Casein | 2 | 1054.12 | 2106.22 | 51.49 | 206-224 | + | + | 2 |
| **172** | LQDKIHPF | 8 | Beta Casein | 2 | 499.28 | 996.54 | 32.88 | 60-67 | + | + | 2 |
| **173** | LQPEVMGVSKVKEA | 14 | Beta Casein | 2 | 757.92 | 1513.82 | 34.02 | 103-116 | + |  | 1 |
| **174** | LQSWMHQPHQPLPPT | 15 | Beta Casein | 3 | 599.63 | 1795.88 | 38.67 | 155-169 | + | + | 2 |
| **175** | LQSWMHQPHQPLPPTVMFPPQ | 21 | Beta Casein | 3 | 832.75 | 2495.22 | 44.43 | 155-175 | + |  | 1 |
| **176** | LQSWMHQPHQPLPPTVMFPPQSVL | 24 | Beta Casein | 3 | 932.48 | 2794.41 | 46.54 | 155-178 | + |  | 1 |
| **177** | LTDVENLHLPLPLLQ | 15 | Beta Casein | 2 | 857.99 | 1713.97 | 51.06 | 142-156 | + |  | 1 |
| **178** | LVYPFPGPIPNSLPQ | 15 | Beta Casein | 2 | 819.95 | 1637.88 | 50.37 | 73-87 | + |  | 1 |
| **179** | LYQEPVLGPVRGPFP | 15 | Beta Casein | 2 | 834.96 | 1667.90 | 45.73 | 207-221 | + | + | 2 |
| **180** | LYQEPVLGPVRGPFPIIV | 18 | Beta Casein | 2 | 997.58 | 1993.14 | 50.48 | 207-224 | + | + | 2 |
| **181** | MAPKHKEMPFPKYPVEPF | 18 | Beta Casein | 3 | 725.04 | 2172.09 | 41.51 | 117-134 | + | + | 2 |
| **182** | MHQPHQPLPPTVMFPPQSVL | 20 | Beta Casein | 3 | 761.06 | 2280.15 | 45.23 | 159-178 | + |  | 1 |
| **183** | MPFPKYPVEPF | 11 | Beta Casein | 2 | 676.34 | 1350.67 | 46.12 | 124-134 | + | + | 2 |
| **184** | NIPPLTQTPV | 10 | Beta Casein | 2 | 540.31 | 1078.60 | 44.58 | 88-97 | + | + | 2 |
| **185** | NIPPLTQTPVVVPPFLQPE | 19 | Beta Casein | 2 | 1043.58 | 2085.15 | 53.22 | 88-106 | + | + | 2 |
| **186** | NIPPLTQTPVVVPPFLQPEVM | 21 | Beta Casein | 2 | 1166.63 | 2331.25 | 53.54 | 88-108 | + | + | 2 |
| **187** | NIPPLTQTPVVVPPFLQPEVMG | 22 | Beta Casein | 2 | 1187.15 | 2372.28 | 54.71 | 88-109 | + | + | 2 |
| **188** | NIPPLTQTPVVVPPFLQPEVMGV | 23 | Beta Casein | 2 | 1236.68 | 2471.35 | 55.05 | 88-110 | + | + | 2 |
| **189** | NIPPLTQTPVVVPPFLQPEVMGVS | 24 | Beta Casein | 2 | 1280.20 | 2558.38 | 54.88 | 88-111 | + |  | 1 |
| **190** | NIPPLTQTPVVVPPFLQPEVMGVSK | 25 | Beta Casein | 3 | 896.50 | 2686.48 | 51.32 | 88-112 | + | + | 2 |
| **191** | NIPPLTQTPVVVPPFLQPEVMGVSKV | 26 | Beta Casein | 3 | 929.52 | 2785.55 | 52.06 | 88-113 | + |  | 1 |
| **192** | NIPPLTQTPVVVPPFLQPEVMGVSKVK | 27 | Beta Casein | 3 | 972.22 | 2913.64 | 49.16 | 88-114 | + |  | 1 |
| **193** | NLHLPLPL | 8 | Beta Casein | 2 | 458.78 | 915.55 | 47.71 | 147-154 | + | + | 2 |
| **194** | NLHLPLPLL | 9 | Beta Casein | 2 | 515.33 | 1028.64 | 50.15 | 147-155 | + | + | 2 |
| **195** | NLHLPLPLLQ | 10 | Beta Casein | 2 | 579.36 | 1156.70 | 49.33 | 147-156 | + | + | 2 |
| **196** | PKH | 3 | Beta Casein | 1 | 381.22 | 380.22 | 63.23 | 119-121 | + |  | 1 |
| **197** | QDKIHPFAQTQ | 11 | Beta Casein | 2 | 656.84 | 1311.66 | 27.38 | 61-71 | + |  | 1 |
| **198** | QEPVLGPVRGPFPII | 15 | Beta Casein | 2 | 809.97 | 1617.92 | 48.61 | 209-223 | + | + | 2 |
| **199** | QEPVLGPVRGPFPIIV | 16 | Beta Casein | 2 | 859.50 | 1716.99 | 49.44 | 209-224 | + | + | 2 |
| **200** | QNIPPLTQTPVVVPPFLQPEVM | 22 | Beta Casein | 2 | 1222.67 | 2443.32 | 54.62 | 87-108 | + |  | 1 |
| **201** | QQQ | 3 | Beta Casein | 1 | 403.19 | 402.19 | 48.61 | 53-55 | + |  | 1 |
| **202** | QQQTEDELQDKIHPF | 15 | Beta Casein | 3 | 619.30 | 1854.87 | 41.24 | 53-67 | + | + | 2 |
| **203** | QQTEDELQDKIHPF | 14 | Beta Casein | 3 | 576.61 | 1726.82 | 41.29 | 54-67 | + | + | 2 |
| **204** | QSEEQQQTEDELQDK | 15 | Beta Casein | 2 | 917.90 | 1833.79 | 26.82 | 49-63 | + | + | 2 |
| **205** | QSEEQQQTEDELQDKIHPF | 19 | Beta Casein | 3 | 777.02 | 2328.05 | 42.11 | 49-67 | + | + | 2 |
| **206** | QSEEQQQTEDELQDKIHPFAQTQ | 23 | Beta Casein | 3 | 919.76 | 2756.25 | 41.72 | 49-71 | + |  | 1 |
| **207** | QSEEQQQTEDELQDKIHPFAQTQSL | 25 | Beta Casein | 3 | 986.46 | 2956.37 | 42.86 | 49-73 | + |  | 1 |
| **208** | QSKVLPVPQ | 9 | Beta Casein | 2 | 498.30 | 994.58 | 34.53 | 182-190 | + | + | 2 |
| **209** | QSKVLPVPQKAVPYPQ | 16 | Beta Casein | 3 | 593.68 | 1778.01 | 38.02 | 182-197 | + | + | 2 |
| **210** | QSLVYPFPGPIPNSLPQ | 17 | Beta Casein | 2 | 927.49 | 1852.97 | 50.37 | 71-87 | + |  | 1 |
| **211** | QSWMHQPHQPLPPT | 14 | Beta Casein | 3 | 561.94 | 1682.80 | 35.33 | 156-169 | + | + | 2 |
| **212** | QTEDELQDK | 9 | Beta Casein | 2 | 553.25 | 1104.49 | 21.05 | 55-63 | + | + | 2 |
| **213** | QTEDELQDKIHP | 12 | Beta Casein | 2 | 726.85 | 1451.69 | 32.68 | 55-66 | + | + | 2 |
| **214** | QTEDELQDKIHPF | 13 | Beta Casein | 2 | 800.39 | 1598.76 | 41.24 | 55-67 | + | + | 2 |
| **215** | QTEDELQDKIHPFAQT | 16 | Beta Casein | 3 | 633.97 | 1898.90 | 40.98 | 55-70 | + |  | 1 |
| **216** | QTEDELQDKIHPFAQTQ | 17 | Beta Casein | 3 | 676.66 | 2026.96 | 40.23 | 55-71 | + |  | 1 |
| **217** | QTPVVVPPFLQPEVM | 15 | Beta Casein | 2 | 840.96 | 1679.90 | 52.88 | 94-108 | + |  | 1 |
| **218** | RDMPIQAF | 8 | Beta Casein | 2 | 489.25 | 976.48 | 42.31 | 198-205 | + | + | 2 |
| **219** | RDMPIQAFLL | 10 | Beta Casein | 2 | 602.33 | 1202.65 | 48.84 | 198-207 | + | + | 2 |
| **220** | SITRINK | 7 | Beta Casein | 2 | 416.26 | 830.50 | 16.04 | 37-43 | + | + | 2 |
| **221** | SKVLPVPQKAVPYPQ | 15 | Beta Casein | 3 | 550.99 | 1649.95 | 37.86 | 183-197 | + |  | 1 |
| **222** | SLPQNIPPL | 9 | Beta Casein | 2 | 489.78 | 977.55 | 46.28 | 84-92 | + | + | 2 |
| **223** | SLPQNIPPLTQTPV | 14 | Beta Casein | 2 | 752.92 | 1503.83 | 46.29 | 84-97 | + |  | 1 |
| **224** | SLPQNIPPLTQTPVVVPP | 18 | Beta Casein | 2 | 949.04 | 1896.07 | 48.49 | 84-101 | + |  | 1 |
| **225** | SLPQNIPPLTQTPVVVPPF | 19 | Beta Casein | 2 | 1022.58 | 2043.14 | 52.45 | 84-102 | + |  | 1 |
| **226** | SLPQNIPPLTQTPVVVPPFLQPE | 23 | Beta Casein | 2 | 1256.20 | 2510.38 | 53.42 | 84-106 | + |  | 1 |
| **227** | SLPQNIPPLTQTPVVVPPFLQPEVM | 25 | Beta Casein | 3 | 914.50 | 2740.49 | 54.88 | 84-108 | + |  | 1 |
| **228** | SLPQNIPPLTQTPVVVPPFLQPEVMGVS | 28 | Beta Casein | 3 | 995.54 | 2983.61 | 54.96 | 84-111 | + |  | 1 |
| **229** | SLSQSKVLPVPQ | 12 | Beta Casein | 2 | 641.87 | 1281.73 | 39.87 | 179-190 | + | + | 2 |
| **230** | SLSQSKVLPVPQKAVPYPQ | 19 | Beta Casein | 3 | 689.39 | 2065.16 | 41.10 | 179-197 | + | + | 2 |
| **231** | SLVYPFPGPIPN | 12 | Beta Casein | 2 | 650.85 | 1299.69 | 49.50 | 72-83 | + | + | 2 |
| **232** | SLVYPFPGPIPNSLPQ | 16 | Beta Casein | 2 | 863.46 | 1724.91 | 50.60 | 72-87 | + | + | 2 |
| **233** | SQSKVLPVPQ | 10 | Beta Casein | 2 | 541.81 | 1081.61 | 34.52 | 181-190 | + | + | 2 |
| **234** | SQSKVLPVPQKAVPYPQ | 17 | Beta Casein | 3 | 622.69 | 1865.04 | 38.20 | 181-197 | + | + | 2 |
| **235** | SVLSLSQSKVLPVPQ | 15 | Beta Casein | 2 | 791.46 | 1580.91 | 44.41 | 176-190 | + | + | 2 |
| **236** | SVLSLSQSKVLPVPQK | 16 | Beta Casein | 2 | 855.51 | 1709.01 | 42.03 | 176-191 | + |  | 1 |
| **237** | SVLSLSQSKVLPVPQKAVPYPQ | 22 | Beta Casein | 3 | 789.12 | 2364.34 | 43.49 | 176-197 | + | + | 2 |
| **238** | SWMHQPHQPLPPT | 13 | Beta Casein | 3 | 519.25 | 1554.74 | 34.94 | 157-169 | + | + | 2 |
| **239** | SWMHQPHQPLPPTVMFPPQ | 19 | Beta Casein | 3 | 752.37 | 2254.08 | 43.88 | 157-175 | + |  | 1 |
| **240** | SWMHQPHQPLPPTVMFPPQSV | 21 | Beta Casein | 3 | 814.40 | 2440.18 | 44.56 | 157-177 | + |  | 1 |
| **241** | SWMHQPHQPLPPTVMFPPQSVL | 22 | Beta Casein | 3 | 852.10 | 2553.27 | 46.12 | 157-178 | + |  | 1 |
| **242** | TDVENLHLPLPL | 12 | Beta Casein | 2 | 680.88 | 1359.74 | 49.32 | 143-154 | + | + | 2 |
| **243** | TDVENLHLPLPLLQ | 14 | Beta Casein | 2 | 801.45 | 1600.88 | 50.48 | 143-156 | + | + | 2 |
| **244** | TEDELQDKIHPF | 12 | Beta Casein | 2 | 736.36 | 1470.70 | 41.29 | 56-67 | + | + | 2 |
| **245** | TEDELQDKIHPFAQTQ | 16 | Beta Casein | 2 | 950.46 | 1898.90 | 40.20 | 56-71 | + |  | 1 |
| **246** | TESQSLTLT | 9 | Beta Casein | 2 | 490.25 | 978.49 | 39.36 | 135-143 | + | + | 2 |
| **247** | TLTDVENLHLPLPL | 14 | Beta Casein | 2 | 787.94 | 1573.87 | 50.81 | 141-154 | + |  | 1 |
| **248** | TLTDVENLHLPLPLLQ | 16 | Beta Casein | 2 | 908.51 | 1815.01 | 52.59 | 141-156 | + |  | 1 |
| **249** | TQTPVVVPPFLQPE | 14 | Beta Casein | 2 | 776.42 | 1550.83 | 50.35 | 93-106 | + | + | 2 |
| **250** | TQTPVVVPPFLQPEVM | 16 | Beta Casein | 2 | 891.48 | 1780.94 | 52.82 | 93-108 | + | + | 2 |
| **251** | TQTPVVVPPFLQPEVMG | 17 | Beta Casein | 2 | 919.99 | 1837.96 | 52.43 | 93-109 | + | + | 2 |
| **252** | TQTPVVVPPFLQPEVMGV | 18 | Beta Casein | 2 | 969.52 | 1937.03 | 54.14 | 93-110 | + |  | 1 |
| **253** | TQTPVVVPPFLQPEVMGVS | 19 | Beta Casein | 2 | 1013.04 | 2024.07 | 53.03 | 93-111 | + |  | 1 |
| **254** | TQTPVVVPPFLQPEVMGVSK | 20 | Beta Casein | 2 | 1077.09 | 2152.16 | 49.10 | 93-112 | + |  | 1 |
| **255** | TQTPVVVPPFLQPEVMGVSKVK | 22 | Beta Casein | 3 | 794.12 | 2379.32 | 46.97 | 93-114 | + |  | 1 |
| **256** | TQTPVVVPPFLQPEVMGVSKVKEA | 24 | Beta Casein | 3 | 860.81 | 2579.40 | 46.90 | 93-116 | + |  | 1 |
| **257** | VENLHLPLPLLQ | 12 | Beta Casein | 2 | 693.41 | 1384.81 | 49.59 | 145-156 | + | + | 2 |
| **258** | VLGPVRGPFPIIV | 13 | Beta Casein | 2 | 682.43 | 1362.84 | 48.67 | 212-224 | + | + | 2 |
| **259** | VLPVPQKAVPYPQ | 13 | Beta Casein | 2 | 718.42 | 1434.82 | 41.83 | 185-197 | + | + | 2 |
| **260** | VMFPPQSVL | 9 | Beta Casein | 2 | 509.28 | 1016.54 | 48.17 | 170-178 | + | + | 2 |
| **261** | VMGVSKVKEAMAPK | 14 | Beta Casein | 3 | 492.28 | 1473.80 | 29.49 | 107-120 | + | + | 2 |
| **262** | VVPPFLQPE | 9 | Beta Casein | 2 | 513.29 | 1024.56 | 47.39 | 98-106 | + |  | 1 |
| **263** | VVPPFLQPEVM | 11 | Beta Casein | 2 | 628.34 | 1254.67 | 50.62 | 98-108 | + |  | 1 |
| **264** | VVPPFLQPEVMGVSK | 15 | Beta Casein | 2 | 813.95 | 1625.89 | 46.57 | 98-112 | + |  | 1 |
| **265** | VYPFPGPIPN | 10 | Beta Casein | 2 | 550.79 | 1099.57 | 47.21 | 74-83 | + | + | 2 |
| **266** | VYPFPGPIPNSLPQ | 14 | Beta Casein | 2 | 763.41 | 1524.80 | 48.78 | 74-87 | + | + | 2 |
| **267** | WMHQPHQPLPPTVMFPPQ | 18 | Beta Casein | 3 | 723.36 | 2167.05 | 43.68 | 158-175 | + |  | 1 |
| **268** | WMHQPHQPLPPTVMFPPQSVL | 21 | Beta Casein | 3 | 823.09 | 2466.23 | 45.92 | 158-178 | + |  | 1 |
| **269** | YPFP | 4 | Beta Casein | 1 | 523.26 | 522.25 | 44.19 | 75-78 | + |  | 1 |
| **270** | YPFPGPIPN | 9 | Beta Casein | 2 | 501.26 | 1000.50 | 46.13 | 75-83 | + | + | 2 |
| **271** | YPFPGPIPNSLPQ | 13 | Beta Casein | 2 | 713.87 | 1425.73 | 47.93 | 75-87 | + |  | 1 |
| **272** | YPV | 3 | Beta Casein | 1 | 378.20 | 377.20 | 29.47 | 129-131 | + |  | 1 |
| **273** | YQEPVLGPVRGPFP | 14 | Beta Casein | 2 | 778.42 | 1554.82 | 44.95 | 208-221 | + | + | 2 |
| **274** | YQEPVLGPVRGPFPI | 15 | Beta Casein | 2 | 834.96 | 1667.90 | 47.31 | 208-222 | + | + | 2 |
| **275** | YQEPVLGPVRGPFPII | 16 | Beta Casein | 2 | 891.50 | 1780.99 | 49.06 | 208-223 | + | + | 2 |
| **276** | YQEPVLGPVRGPFPIIV | 17 | Beta Casein | 2 | 941.04 | 1880.06 | 49.90 | 208-224 | + | + | 2 |
| **277** | AQTQSL | 6 | Beta Casein | 1 | 647.34 | 646.33 | 24.35 | 68-73 |  | + | 1 |
| **278** | AVPYPQRDMPIQ | 12 | Beta Casein | 2 | 707.86 | 1413.71 | 39.54 | 192-203 |  | + | 1 |
| **279** | AVPYPQRDMPIQA | 13 | Beta Casein | 2 | 743.38 | 1484.74 | 41.22 | 192-204 |  | + | 1 |
| **280** | DELQDKIHP | 9 | Beta Casein | 2 | 547.78 | 1093.54 | 32.14 | 58-66 |  | + | 1 |
| **281** | DKIHPFAQ | 8 | Beta Casein | 2 | 478.25 | 954.49 | 27.92 | 62-69 |  | + | 1 |
| **282** | DKIHPFAQT | 9 | Beta Casein | 2 | 528.78 | 1055.54 | 28.76 | 62-70 |  | + | 1 |
| **283** | DKIHPFAQTQS | 11 | Beta Casein | 2 | 636.32 | 1270.63 | 27.80 | 62-72 |  | + | 1 |
| **284** | DMPIQAFLL | 9 | Beta Casein | 2 | 524.28 | 1046.55 | 55.28 | 199-207 |  | + | 1 |
| **285** | DVENLHLP | 8 | Beta Casein | 2 | 468.74 | 935.47 | 43.56 | 144-151 |  | + | 1 |
| **286** | EMPFPKYP | 8 | Beta Casein | 2 | 504.75 | 1007.48 | 42.87 | 123-130 |  | + | 1 |
| **287** | ENLHLPLPL | 9 | Beta Casein | 2 | 523.31 | 1044.60 | 47.88 | 146-154 |  | + | 1 |
| **288** | ENLHLPLPLLQ | 11 | Beta Casein | 2 | 643.88 | 1285.74 | 49.45 | 146-156 |  | + | 1 |
| **289** | ESITRIN | 7 | Beta Casein | 2 | 416.73 | 831.45 | 26.02 | 36-42 |  | + | 1 |
| **290** | FPKYPVEPF | 9 | Beta Casein | 2 | 562.29 | 1122.57 | 44.37 | 126-134 |  | + | 1 |
| **291** | FPPQSVLSLSQS | 12 | Beta Casein | 2 | 645.34 | 1288.67 | 46.46 | 172-183 |  | + | 1 |
| **292** | GPVRGPFP | 8 | Beta Casein | 2 | 413.73 | 825.45 | 36.77 | 214-221 |  | + | 1 |
| **293** | GVSKVKEAM | 9 | Beta Casein | 2 | 474.76 | 947.51 | 19.40 | 109-117 |  | + | 1 |
| **294** | GVSKVKEAMAP | 11 | Beta Casein | 2 | 558.81 | 1115.60 | 23.86 | 109-119 |  | + | 1 |
| **295** | HLPLPLLQ | 8 | Beta Casein | 2 | 465.79 | 929.57 | 46.40 | 149-156 |  | + | 1 |
| **296** | HQPHQPLPP | 9 | Beta Casein | 2 | 525.78 | 1049.54 | 22.38 | 160-168 |  | + | 1 |
| **297** | IHPFAQTQ | 8 | Beta Casein | 2 | 471.25 | 940.48 | 29.15 | 64-71 |  | + | 1 |
| **298** | INKKIEK | 7 | Beta Casein | 2 | 436.78 | 871.55 | 13.33 | 41-47 |  | + | 1 |
| **299** | INKKIEKF | 8 | Beta Casein | 2 | 510.32 | 1018.62 | 19.99 | 41-48 |  | + | 1 |
| **300** | INKKIEKFQ | 9 | Beta Casein | 2 | 574.35 | 1146.68 | 19.71 | 41-49 |  | + | 1 |
| **301** | KAVPYPQR | 8 | Beta Casein | 2 | 479.78 | 957.54 | 20.10 | 191-198 |  | + | 1 |
| **302** | KAVPYPQRD | 9 | Beta Casein | 2 | 537.29 | 1072.57 | 20.65 | 191-199 |  | + | 1 |
| **303** | KEMPFPKYPVEPF | 13 | Beta Casein | 3 | 536.94 | 1607.81 | 43.78 | 122-134 |  | + | 1 |
| **304** | KIHPFAQTQ | 9 | Beta Casein | 2 | 535.29 | 1068.57 | 23.36 | 63-71 |  | + | 1 |
| **305** | KKIEKFQ | 7 | Beta Casein | 2 | 460.78 | 919.55 | 14.70 | 43-49 |  | + | 1 |
| **306** | KKIEKFQSE | 9 | Beta Casein | 2 | 568.82 | 1135.62 | 15.63 | 43-51 |  | + | 1 |
| **307** | KVKEAMAPK | 9 | Beta Casein | 2 | 501.29 | 1000.57 | 14.29 | 112-120 |  | + | 1 |
| **308** | KVLPVPQ | 7 | Beta Casein | 2 | 390.75 | 779.49 | 32.84 | 184-190 |  | + | 1 |
| **309** | KVLPVPQK | 8 | Beta Casein | 2 | 454.80 | 907.59 | 24.20 | 184-191 |  | + | 1 |
| **310** | LGPVRGPFPIIV | 12 | Beta Casein | 2 | 632.89 | 1263.77 | 48.20 | 213-224 |  | + | 1 |
| **311** | LHLPLPL | 7 | Beta Casein | 2 | 401.76 | 801.51 | 47.40 | 148-154 |  | + | 1 |
| **312** | LHLPLPLL | 8 | Beta Casein | 2 | 458.30 | 914.60 | 49.95 | 148-155 |  | + | 1 |
| **313** | LHLPLPLLQS | 10 | Beta Casein | 2 | 565.85 | 1129.69 | 48.83 | 148-157 |  | + | 1 |
| **314** | LLYQEPVLGPVR | 12 | Beta Casein | 2 | 692.40 | 1382.79 | 43.68 | 206-217 |  | + | 1 |
| **315** | LNVPGEIVE | 9 | Beta Casein | 2 | 485.27 | 968.52 | 44.09 | 21-29 |  | + | 1 |
| **316** | LQSW | 4 | Beta Casein | 1 | 533.27 | 532.26 | 37.38 | 155-158 |  | + | 1 |
| **317** | LSLSQSKVLPVPQ | 13 | Beta Casein | 2 | 698.41 | 1394.81 | 43.12 | 178-190 |  | + | 1 |
| **318** | LSQSKVLPVPQ | 11 | Beta Casein | 2 | 598.36 | 1194.70 | 38.13 | 180-190 |  | + | 1 |
| **319** | LSQSKVLPVPQKAVPYPQ | 18 | Beta Casein | 3 | 660.38 | 1978.13 | 40.30 | 180-197 |  | + | 1 |
| **320** | LTQTPVVVPPFLQPEVM | 17 | Beta Casein | 2 | 948.02 | 1894.03 | 53.41 | 92-108 |  | + | 1 |
| **321** | LVYPFPGPIPN | 11 | Beta Casein | 2 | 607.33 | 1212.65 | 49.06 | 73-83 |  | + | 1 |
| **322** | MFPPQSVL | 8 | Beta Casein | 2 | 459.74 | 917.47 | 46.64 | 171-178 |  | + | 1 |
| **323** | MHQPHQPLPPT | 11 | Beta Casein | 2 | 641.82 | 1281.63 | 25.87 | 159-169 |  | + | 1 |
| **324** | MPFPKYP | 7 | Beta Casein | 2 | 440.23 | 878.44 | 42.10 | 124-130 |  | + | 1 |
| **325** | MPIQA | 5 | Beta Casein | 1 | 559.29 | 558.28 | 34.20 | 200-204 |  | + | 1 |
| **326** | NIPPLTQTPVVVPPF | 15 | Beta Casein | 2 | 809.96 | 1617.91 | 52.05 | 88-102 |  | + | 1 |
| **327** | NIPPLTQTPVVVPPFLQP | 18 | Beta Casein | 2 | 979.06 | 1956.11 | 53.67 | 88-105 |  | + | 1 |
| **328** | NLHLPLP | 7 | Beta Casein | 2 | 402.24 | 802.47 | 44.69 | 147-153 |  | + | 1 |
| **329** | NLHLPLPLLQS | 11 | Beta Casein | 2 | 622.87 | 1243.73 | 49.11 | 147-157 |  | + | 1 |
| **330** | NVPGE | 5 | Beta Casein | 1 | 515.25 | 514.24 | 21.61 | 22-26 |  | + | 1 |
| **331** | PFPKYPVEPF | 10 | Beta Casein | 2 | 610.82 | 1219.63 | 44.71 | 125-134 |  | + | 1 |
| **332** | PPLTQTPVVVPPFLQPEVMGVS | 22 | Beta Casein | 2 | 1166.63 | 2331.25 | 53.56 | 90-111 |  | + | 1 |
| **333** | QDKIHPF | 7 | Beta Casein | 2 | 442.73 | 883.46 | 27.96 | 61-67 |  | + | 1 |
| **334** | QEPVLGPVRGPFP | 13 | Beta Casein | 2 | 696.89 | 1391.76 | 44.47 | 209-221 |  | + | 1 |
| **335** | QEPVLGPVRGPFPI | 14 | Beta Casein | 2 | 753.43 | 1504.84 | 46.76 | 209-222 |  | + | 1 |
| **336** | QPH | 3 | Beta Casein | 1 | 381.19 | 380.18 | 61.50 | 161-163 |  | + | 1 |
| **337** | QQQTEDELQ | 9 | Beta Casein | 2 | 559.75 | 1117.49 | 26.22 | 53-61 |  | + | 1 |
| **338** | QQQTEDELQDK | 11 | Beta Casein | 2 | 681.31 | 1360.61 | 22.14 | 53-63 |  | + | 1 |
| **339** | QQTEDELQ | 8 | Beta Casein | 2 | 495.72 | 989.43 | 26.29 | 54-61 |  | + | 1 |
| **340** | QQTEDELQDK | 10 | Beta Casein | 2 | 617.28 | 1232.55 | 21.84 | 54-63 |  | + | 1 |
| **341** | QSEEQQQTED | 10 | Beta Casein | 2 | 611.25 | 1220.48 | 16.43 | 49-58 |  | + | 1 |
| **342** | QSLVYPFPGPIPN | 13 | Beta Casein | 2 | 714.88 | 1427.74 | 49.22 | 71-83 |  | + | 1 |
| **343** | RDMPIQ | 6 | Beta Casein | 2 | 380.19 | 758.37 | 25.65 | 198-203 |  | + | 1 |
| **344** | RDMPIQA | 7 | Beta Casein | 2 | 415.71 | 829.41 | 29.94 | 198-204 |  | + | 1 |
| **345** | RDMPIQAFL | 9 | Beta Casein | 2 | 545.79 | 1089.56 | 46.33 | 198-206 |  | + | 1 |
| **346** | RELEEL | 6 | Beta Casein | 2 | 394.71 | 787.41 | 32.04 | 16-21 |  | + | 1 |
| **347** | RGPFPIIV | 8 | Beta Casein | 2 | 449.78 | 897.54 | 45.69 | 217-224 |  | + | 1 |
| **348** | RINKKIE | 7 | Beta Casein | 2 | 450.78 | 899.56 | 14.02 | 40-46 |  | + | 1 |
| **349** | SEEQQQTEDELQDK | 14 | Beta Casein | 2 | 853.87 | 1705.73 | 26.02 | 50-63 |  | + | 1 |
| **350** | SEEQQQTEDELQDKIHPF | 18 | Beta Casein | 3 | 734.34 | 2199.99 | 41.97 | 50-67 |  | + | 1 |
| **351** | SITRIN | 6 | Beta Casein | 2 | 352.21 | 702.40 | 22.84 | 37-42 |  | + | 1 |
| **352** | SWMHQPHQPLPP | 12 | Beta Casein | 3 | 485.57 | 1453.69 | 35.04 | 157-168 |  | + | 1 |
| **353** | TEDELQDK | 8 | Beta Casein | 2 | 489.22 | 976.43 | 19.94 | 56-63 |  | + | 1 |
| **354** | TEDELQDKIHP | 11 | Beta Casein | 2 | 662.82 | 1323.63 | 32.39 | 56-66 |  | + | 1 |
| **355** | TESQSLTLTDVE | 12 | Beta Casein | 2 | 661.82 | 1321.62 | 43.50 | 135-146 |  | + | 1 |
| **356** | TQTPVVVPPFL | 11 | Beta Casein | 2 | 599.35 | 1196.68 | 51.78 | 93-103 |  | + | 1 |
| **357** | TQTPVVVPPFLQP | 13 | Beta Casein | 2 | 711.90 | 1421.79 | 50.86 | 93-105 |  | + | 1 |
| **358** | TVMFPPQSVL | 10 | Beta Casein | 2 | 559.80 | 1117.58 | 48.66 | 169-178 |  | + | 1 |
| **359** | TVMFPPQSVLSL | 12 | Beta Casein | 2 | 659.86 | 1317.70 | 51.97 | 169-180 |  | + | 1 |
| **360** | VENLHLPLPL | 10 | Beta Casein | 2 | 572.84 | 1143.67 | 48.08 | 145-154 |  | + | 1 |
| **361** | VKEAMAPK | 8 | Beta Casein | 2 | 437.25 | 872.48 | 15.81 | 113-120 |  | + | 1 |
| **362** | VLGPVRGPFP | 10 | Beta Casein | 2 | 519.81 | 1037.60 | 43.22 | 212-221 |  | + | 1 |
| **363** | VLGPVRGPFPI | 11 | Beta Casein | 2 | 576.35 | 1150.69 | 45.73 | 212-222 |  | + | 1 |
| **364** | VLSLSQSKVLPVPQ | 14 | Beta Casein | 2 | 747.95 | 1493.88 | 43.78 | 177-190 |  | + | 1 |
| **365** | VMFPPQS | 7 | Beta Casein | 1 | 805.39 | 804.38 | 42.26 | 170-176 |  | + | 1 |
| **366** | VMFPPQSVLSL | 11 | Beta Casein | 2 | 609.33 | 1216.65 | 51.71 | 170-180 |  | + | 1 |
| **367** | VMFPPQSVLSLS | 12 | Beta Casein | 2 | 652.85 | 1303.68 | 50.28 | 170-181 |  | + | 1 |
| **368** | VMGVSKVK | 8 | Beta Casein | 2 | 424.26 | 846.50 | 20.15 | 107-114 |  | + | 1 |
| **369** | VMGVSKVKE | 9 | Beta Casein | 2 | 488.78 | 975.54 | 22.20 | 107-115 |  | + | 1 |
| **370** | VMGVSKVKEA | 10 | Beta Casein | 2 | 524.30 | 1046.58 | 23.07 | 107-116 |  | + | 1 |
| **371** | VPYPQRDMPIQ | 11 | Beta Casein | 2 | 672.34 | 1342.67 | 38.22 | 193-203 |  | + | 1 |
| **372** | VPYPQRDMPIQA | 12 | Beta Casein | 2 | 707.86 | 1413.71 | 40.37 | 193-204 |  | + | 1 |
| **373** | VRGPFPIIV | 9 | Beta Casein | 2 | 499.31 | 996.61 | 46.41 | 216-224 |  | + | 1 |
| **374** | VVPPFLQP | 8 | Beta Casein | 2 | 448.77 | 895.52 | 47.95 | 98-105 |  | + | 1 |
| **375** | VVVPPFLQPE | 10 | Beta Casein | 2 | 562.82 | 1123.63 | 49.55 | 97-106 |  | + | 1 |
| **376** | VVVPPFLQPEVM | 12 | Beta Casein | 2 | 677.88 | 1353.74 | 52.32 | 97-108 |  | + | 1 |
| **377** | WMHQPHQPLPP | 11 | Beta Casein | 3 | 456.56 | 1366.66 | 32.40 | 158-168 |  | + | 1 |
| **378** | WMHQPHQPLPPT | 12 | Beta Casein | 3 | 490.24 | 1467.71 | 32.77 | 158-169 |  | + | 1 |
| **379** | YQEPVLGPVR | 10 | Beta Casein | 2 | 579.32 | 1156.62 | 38.61 | 208-217 |  | + | 1 |
| **380** | MKT | 3 | CON__Q3T052 | 1 | 379.20 | 378.19 | 40.88 | 1-3 | + |  | 1 |
| **381** | AIPPKKNQ | 8 | Kappa Casein | 2 | 448.27 | 894.53 | 13.94 | 128-135 | + | + | 2 |
| **382** | AIPPKKNQD | 9 | Kappa Casein | 2 | 505.79 | 1009.56 | 14.22 | 128-136 | + | + | 2 |
| **383** | AIPPKKNQDKTEIPTIN | 17 | Kappa Casein | 3 | 636.36 | 1906.05 | 29.99 | 128-144 | + |  | 1 |
| **384** | ALINNQFLPYPYYAKPA | 17 | Kappa Casein | 2 | 992.02 | 1982.03 | 46.14 | 70-86 | + | + | 2 |
| **385** | AVRSPAQIL | 9 | Kappa Casein | 2 | 477.79 | 953.57 | 39.53 | 87-95 | + | + | 2 |
| **386** | AVRSPAQILQWQVL | 14 | Kappa Casein | 2 | 804.96 | 1607.91 | 48.13 | 87-100 | + | + | 2 |
| **387** | DKIAKYIPI | 9 | Kappa Casein | 2 | 530.82 | 1059.63 | 42.51 | 41-49 | + | + | 2 |
| **388** | DKIAKYIPIQY | 11 | Kappa Casein | 2 | 676.38 | 1350.75 | 42.77 | 41-51 | + | + | 2 |
| **389** | DKTEIPTIN | 9 | Kappa Casein | 2 | 515.77 | 1029.53 | 37.79 | 136-144 | + | + | 2 |
| **390** | DSPEVIESPPEINTVQVTSTAV | 22 | Kappa Casein | 2 | 1156.58 | 2311.14 | 46.97 | 169-190 | + |  | 1 |
| **391** | EVIESPPEINTVQVTSTAV | 19 | Kappa Casein | 2 | 1007.02 | 2012.03 | 45.55 | 172-190 | + |  | 1 |
| **392** | FFL | 3 | Kappa Casein | 1 | 426.24 | 425.23 | 55.94 | 5-7 | + |  | 1 |
| **393** | FMAIPPKKN | 9 | Kappa Casein | 2 | 523.30 | 1044.58 | 29.28 | 126-134 | + | + | 2 |
| **394** | IESPPEINTVQVTSTAV | 17 | Kappa Casein | 2 | 892.97 | 1783.92 | 44.80 | 174-190 | + |  | 1 |
| **395** | INNQFLPYPY | 10 | Kappa Casein | 2 | 634.82 | 1267.62 | 47.75 | 72-81 | + | + | 2 |
| **396** | INNQFLPYPYYAKPA | 15 | Kappa Casein | 2 | 899.96 | 1797.91 | 44.41 | 72-86 | + | + | 2 |
| **397** | KIAKYIPIQY | 10 | Kappa Casein | 2 | 618.87 | 1235.73 | 40.26 | 42-51 | + | + | 2 |
| **398** | KTEIPTIN | 8 | Kappa Casein | 2 | 458.26 | 914.51 | 34.62 | 137-144 | + | + | 2 |
| **399** | KYIPIQYVLS | 10 | Kappa Casein | 2 | 612.36 | 1222.70 | 44.59 | 45-54 | + | + | 2 |
| **400** | NYYQQKPVAL | 10 | Kappa Casein | 2 | 612.32 | 1222.63 | 36.80 | 62-71 | + | + | 2 |
| **401** | PEVIESPPEINTVQVTSTAV | 20 | Kappa Casein | 2 | 1055.55 | 2109.08 | 45.88 | 171-190 | + |  | 1 |
| **402** | QDKTEIPTIN | 10 | Kappa Casein | 2 | 579.80 | 1157.59 | 36.15 | 135-144 | + | + | 2 |
| **403** | QFLPYPYYAKPA | 12 | Kappa Casein | 2 | 729.38 | 1456.74 | 43.92 | 75-86 | + | + | 2 |
| **404** | QQK | 3 | Kappa Casein | 1 | 403.23 | 402.22 | 61.38 | 65-67 | + |  | 1 |
| **405** | QYVLSRYPSYGLN | 13 | Kappa Casein | 2 | 780.40 | 1558.78 | 42.43 | 50-62 | + | + | 2 |
| **406** | SDKIAKYIPIQY | 12 | Kappa Casein | 2 | 719.90 | 1437.79 | 42.45 | 40-51 | + | + | 2 |
| **407** | SPPEINTVQVTSTAV | 15 | Kappa Casein | 2 | 771.90 | 1541.79 | 43.60 | 176-190 | + | + | 2 |
| **408** | TIASGEPTSTPTTE | 14 | Kappa Casein | 2 | 696.33 | 1390.65 | 31.49 | 145-158 | + | + | 2 |
| **409** | TIASGEPTSTPTTEAVE | 17 | Kappa Casein | 2 | 845.90 | 1689.79 | 37.08 | 145-161 | + |  | 1 |
| **410** | TIASGEPTSTPTTEAVESTVAT | 22 | Kappa Casein | 2 | 1075.52 | 2149.03 | 42.31 | 145-166 | + |  | 1 |
| **411** | TIASGEPTSTPTTEAVESTVATL | 23 | Kappa Casein | 2 | 1132.06 | 2262.11 | 47.12 | 145-167 | + |  | 1 |
| **412** | VIESPPEIN | 9 | Kappa Casein | 2 | 499.26 | 996.51 | 39.76 | 173-181 | + | + | 2 |
| **413** | VIESPPEINTVQVT | 14 | Kappa Casein | 2 | 763.41 | 1524.80 | 43.92 | 173-186 | + | + | 2 |
| **414** | VIESPPEINTVQVTST | 16 | Kappa Casein | 2 | 857.45 | 1712.88 | 43.73 | 173-188 | + |  | 1 |
| **415** | VIESPPEINTVQVTSTAV | 18 | Kappa Casein | 2 | 942.50 | 1882.99 | 45.06 | 173-190 | + | + | 2 |
| **416** | VLSRYPSYGL | 10 | Kappa Casein | 2 | 577.81 | 1153.61 | 41.47 | 52-61 | + | + | 2 |
| **417** | VLSRYPSYGLN | 11 | Kappa Casein | 2 | 634.84 | 1267.66 | 38.27 | 52-62 | + | + | 2 |
| **418** | YQQKPVALINN | 11 | Kappa Casein | 2 | 644.36 | 1286.70 | 34.84 | 64-74 | + | + | 2 |
| **419** | YVLSRYPSYGLN | 12 | Kappa Casein | 2 | 716.37 | 1430.72 | 42.26 | 51-62 | + | + | 2 |
| **420** | YYQQKPV | 7 | Kappa Casein | 2 | 463.24 | 924.47 | 22.35 | 63-69 | + | + | 2 |
| **421** | YYQQKPVA | 8 | Kappa Casein | 2 | 498.76 | 995.51 | 22.58 | 63-70 | + | + | 2 |
| **422** | YYQQKPVAL | 9 | Kappa Casein | 2 | 555.30 | 1108.59 | 34.44 | 63-71 | + | + | 2 |
| **423** | YYQQKPVALINN | 12 | Kappa Casein | 2 | 725.89 | 1449.76 | 37.35 | 63-74 | + | + | 2 |
| **424** | ALINNQFLPYPY | 12 | Kappa Casein | 2 | 726.88 | 1451.74 | 50.12 | 70-81 |  | + | 1 |
| **425** | AVRSPAQ | 7 | Kappa Casein | 2 | 364.71 | 727.40 | 16.11 | 87-93 |  | + | 1 |
| **426** | DERFFSD | 7 | Kappa Casein | 2 | 458.20 | 914.38 | 39.60 | 35-41 |  | + | 1 |
| **427** | DERFFSDK | 8 | Kappa Casein | 2 | 522.24 | 1042.47 | 30.74 | 35-42 |  | + | 1 |
| **428** | DERFFSDKIA | 10 | Kappa Casein | 2 | 614.30 | 1226.59 | 40.79 | 35-44 |  | + | 1 |
| **429** | DKIAKYIP | 8 | Kappa Casein | 2 | 474.28 | 946.55 | 36.13 | 41-48 |  | + | 1 |
| **430** | DKIAKYIPIQ | 10 | Kappa Casein | 2 | 594.85 | 1187.69 | 40.80 | 41-50 |  | + | 1 |
| **431** | EKDERFF | 7 | Kappa Casein | 2 | 485.74 | 969.46 | 32.53 | 33-39 |  | + | 1 |
| **432** | EKDERFFSD | 9 | Kappa Casein | 2 | 586.76 | 1171.51 | 30.79 | 33-41 |  | + | 1 |
| **433** | EQNQEQPIR | 9 | Kappa Casein | 2 | 571.28 | 1140.55 | 19.62 | 23-31 |  | + | 1 |
| **434** | ERFFSDKIA | 9 | Kappa Casein | 2 | 556.79 | 1111.57 | 36.04 | 36-44 |  | + | 1 |
| **435** | FFSDK | 5 | Kappa Casein | 1 | 643.31 | 642.30 | 26.99 | 38-42 |  | + | 1 |
| **436** | FFSDKIA | 7 | Kappa Casein | 2 | 414.22 | 826.42 | 38.70 | 38-44 |  | + | 1 |
| **437** | FLPYPYYAKPA | 11 | Kappa Casein | 2 | 665.35 | 1328.68 | 43.28 | 76-86 |  | + | 1 |
| **438** | FSDKIAKYIPI | 11 | Kappa Casein | 2 | 647.87 | 1293.73 | 43.72 | 39-49 |  | + | 1 |
| **439** | IAKYIPI | 7 | Kappa Casein | 2 | 409.26 | 816.51 | 42.11 | 43-49 |  | + | 1 |
| **440** | IAKYIPIQ | 8 | Kappa Casein | 2 | 473.29 | 944.57 | 39.44 | 43-50 |  | + | 1 |
| **441** | IAKYIPIQY | 9 | Kappa Casein | 2 | 554.82 | 1107.63 | 42.67 | 43-51 |  | + | 1 |
| **442** | IESPPEINTVQVT | 13 | Kappa Casein | 2 | 713.87 | 1425.74 | 43.49 | 174-186 |  | + | 1 |
| **443** | INTVQVTSTAV | 11 | Kappa Casein | 2 | 566.81 | 1131.61 | 41.52 | 180-190 |  | + | 1 |
| **444** | KDERFFS | 7 | Kappa Casein | 2 | 464.73 | 927.45 | 28.74 | 34-40 |  | + | 1 |
| **445** | KDERFFSD | 8 | Kappa Casein | 2 | 522.24 | 1042.47 | 30.05 | 34-41 |  | + | 1 |
| **446** | KIAKYIPI | 8 | Kappa Casein | 2 | 473.31 | 944.61 | 38.33 | 42-49 |  | + | 1 |
| **447** | KIAKYIPIQ | 9 | Kappa Casein | 2 | 537.34 | 1072.66 | 34.96 | 42-50 |  | + | 1 |
| **448** | KYIPIQ | 6 | Kappa Casein | 2 | 381.23 | 760.45 | 32.46 | 45-50 |  | + | 1 |
| **449** | KYIPIQY | 7 | Kappa Casein | 2 | 462.76 | 923.51 | 39.60 | 45-51 |  | + | 1 |
| **450** | KYIPIQYV | 8 | Kappa Casein | 2 | 512.30 | 1022.58 | 42.41 | 45-52 |  | + | 1 |
| **451** | LINNQFLPYPY | 11 | Kappa Casein | 2 | 691.36 | 1380.71 | 49.16 | 71-81 |  | + | 1 |
| **452** | LINNQFLPYPYYAKPA | 16 | Kappa Casein | 2 | 956.50 | 1910.99 | 45.30 | 71-86 |  | + | 1 |
| **453** | LPYPYYAKPA | 10 | Kappa Casein | 2 | 591.81 | 1181.61 | 39.70 | 77-86 |  | + | 1 |
| **454** | LSRYPSYGL | 9 | Kappa Casein | 2 | 528.28 | 1054.54 | 39.28 | 53-61 |  | + | 1 |
| **455** | LSRYPSYGLN | 10 | Kappa Casein | 2 | 585.30 | 1168.59 | 35.94 | 53-62 |  | + | 1 |
| **456** | MAIPPKKN | 8 | Kappa Casein | 2 | 449.76 | 897.51 | 19.96 | 127-134 |  | + | 1 |
| **457** | NNQFLPYPY | 9 | Kappa Casein | 2 | 578.28 | 1154.54 | 46.99 | 73-81 |  | + | 1 |
| **458** | NNQFLPYPYYAKPA | 14 | Kappa Casein | 2 | 843.42 | 1684.82 | 43.84 | 73-86 |  | + | 1 |
| **459** | NQDKTEIPTIN | 11 | Kappa Casein | 2 | 636.83 | 1271.64 | 35.88 | 134-144 |  | + | 1 |
| **460** | NQFLPYPY | 8 | Kappa Casein | 2 | 521.26 | 1040.50 | 47.28 | 74-81 |  | + | 1 |
| **461** | NYYQQKPV | 8 | Kappa Casein | 2 | 520.26 | 1038.51 | 25.74 | 62-69 |  | + | 1 |
| **462** | NYYQQKPVA | 9 | Kappa Casein | 2 | 555.78 | 1109.55 | 26.06 | 62-70 |  | + | 1 |
| **463** | QDKTEIPT | 8 | Kappa Casein | 2 | 466.24 | 930.47 | 26.21 | 135-142 |  | + | 1 |
| **464** | QFLPYPY | 7 | Kappa Casein | 2 | 464.23 | 926.45 | 47.40 | 75-81 |  | + | 1 |
| **465** | QWQVLSNTVPA | 11 | Kappa Casein | 2 | 621.83 | 1241.64 | 45.46 | 96-106 |  | + | 1 |
| **466** | QWQVLSNTVPAK | 12 | Kappa Casein | 2 | 685.87 | 1369.74 | 41.94 | 96-107 |  | + | 1 |
| **467** | QWQVLSNTVPAKS | 13 | Kappa Casein | 2 | 729.39 | 1456.77 | 41.87 | 96-108 |  | + | 1 |
| **468** | QYVLSRYPS | 9 | Kappa Casein | 2 | 556.79 | 1111.57 | 35.37 | 50-58 |  | + | 1 |
| **469** | QYVLSRYPSYGL | 12 | Kappa Casein | 2 | 723.37 | 1444.74 | 43.45 | 50-61 |  | + | 1 |
| **470** | RFFSDKIA | 8 | Kappa Casein | 2 | 492.27 | 982.52 | 33.96 | 37-44 |  | + | 1 |
| **471** | RYPSYGLN | 8 | Kappa Casein | 2 | 485.24 | 968.47 | 32.67 | 55-62 |  | + | 1 |
| **472** | SDKIAKYIPI | 10 | Kappa Casein | 2 | 574.34 | 1146.66 | 41.94 | 40-49 |  | + | 1 |
| **473** | SPPEINTV | 8 | Kappa Casein | 2 | 428.72 | 855.43 | 38.00 | 176-183 |  | + | 1 |
| **474** | SPPEINTVQ | 9 | Kappa Casein | 2 | 492.75 | 983.49 | 34.57 | 176-184 |  | + | 1 |
| **475** | SPPEINTVQVT | 11 | Kappa Casein | 2 | 592.81 | 1183.61 | 41.66 | 176-186 |  | + | 1 |
| **476** | SPPEINTVQVTST | 13 | Kappa Casein | 2 | 686.85 | 1371.69 | 41.51 | 176-188 |  | + | 1 |
| **477** | SRYPSYGLN | 9 | Kappa Casein | 2 | 528.76 | 1055.50 | 32.92 | 54-62 |  | + | 1 |
| **478** | STVATL | 6 | Kappa Casein | 1 | 591.33 | 590.33 | 35.48 | 162-167 |  | + | 1 |
| **479** | TVQVTSTAV | 9 | Kappa Casein | 2 | 453.25 | 904.49 | 35.17 | 182-190 |  | + | 1 |
| **480** | VLSRYPS | 7 | Kappa Casein | 2 | 411.23 | 820.44 | 25.10 | 52-58 |  | + | 1 |
| **481** | VLSRYPSYG | 9 | Kappa Casein | 2 | 521.27 | 1040.53 | 31.25 | 52-60 |  | + | 1 |
| **482** | YAKPAAVR | 8 | Kappa Casein | 2 | 438.26 | 874.50 | 18.27 | 82-89 |  | + | 1 |
| **483** | YAKPAAVRSPA | 11 | Kappa Casein | 2 | 565.82 | 1129.62 | 22.42 | 82-92 |  | + | 1 |
| **484** | YPYY | 4 | Kappa Casein | 1 | 605.26 | 604.25 | 38.39 | 79-82 |  | + | 1 |
| **485** | YQQKPVA | 7 | Kappa Casein | 2 | 417.23 | 832.44 | 18.48 | 64-70 |  | + | 1 |
| **486** | YQQKPVAL | 8 | Kappa Casein | 2 | 473.77 | 945.53 | 30.87 | 64-71 |  | + | 1 |
| **487** | YQQKPVALIN | 10 | Kappa Casein | 2 | 587.33 | 1172.66 | 35.91 | 64-73 |  | + | 1 |
| **488** | YVLSRYPS | 8 | Kappa Casein | 2 | 492.76 | 983.51 | 34.03 | 51-58 |  | + | 1 |
| **489** | YVLSRYPSYGL | 11 | Kappa Casein | 2 | 659.35 | 1316.68 | 43.22 | 51-61 |  | + | 1 |
| **490** | YYA | 3 | Kappa Casein | 1 | 416.18 | 415.17 | 23.22 | 81-83 |  | + | 1 |
| **491** | YYQQKPVALIN | 11 | Kappa Casein | 2 | 668.87 | 1335.72 | 38.54 | 63-73 |  | + | 1 |
| **492** | YYQQKPVALINNQ | 13 | Kappa Casein | 2 | 789.92 | 1577.82 | 37.26 | 63-75 |  | + | 1 |
| **493** | DAFLGSFLYEYSR | 13 | Serum Albumin | 2 | 784.38 | 1566.74 | 49.84 | 347-359 | + |  | 1 |
| **494** | FKDLGEEHFK | 10 | Serum Albumin | 3 | 417.21 | 1248.61 | 28.02 | 35-44 | + |  | 1 |
| **495** | GLVLIAFSQYLQQCPFDEHVK | 21 | Serum Albumin | 3 | 831.43 | 2491.26 | 49.56 | 45-65 | + |  | 1 |
| **496** | HLVDEPQNLIK | 11 | Serum Albumin | 2 | 653.36 | 1304.71 | 36.81 | 402-412 | + |  | 1 |
| **497** | KQTALVELLK | 10 | Serum Albumin | 2 | 571.86 | 1141.71 | 41.44 | 548-557 | + |  | 1 |
| **498** | KVPQVSTPTLVEVSR | 15 | Serum Albumin | 3 | 547.32 | 1638.93 | 39.68 | 437-451 | + |  | 1 |
| **499** | LGEYGFQNALIVR | 13 | Serum Albumin | 2 | 740.40 | 1478.79 | 44.84 | 421-433 | + |  | 1 |
| **500** | LLF | 3 | Serum Albumin | 1 | 392.25 | 391.25 | 47.12 | 1758-1760 | + |  | 1 |
| **501** | LLL | 3 | Serum Albumin | 1 | 358.27 | 357.26 | 63.55 | 521-523 | + |  | 1 |
| **502** | LPKLKPD | 7 | Serum Albumin | 2 | 405.76 | 809.50 | 26.90 | 136-142 | + |  | 1 |
| **503** | MPCTEDYLSLILNR | 14 | Serum Albumin | 2 | 862.92 | 1723.83 | 49.23 | 469-482 | + |  | 1 |
| **504** | PFD | 3 | Serum Albumin | 1 | 378.17 | 377.16 | 46.46 | 59-61 | + |  | 1 |
| **505** | RDT | 3 | Serum Albumin | 1 | 391.19 | 390.19 | 9.98 | 428-430 | + |  | 1 |
| **506** | RHPEYAVSVLLR | 12 | Serum Albumin | 3 | 480.61 | 1438.80 | 38.62 | 360-371 | + |  | 1 |
| **507** | RPC | 3 | Serum Albumin | 1 | 432.20 | 431.20 | 10.30 | 508-510 | + |  | 1 |
| **508** | TVMENFVAFVDK | 12 | Serum Albumin | 2 | 700.35 | 1398.69 | 47.21 | 569-580 | + |  | 1 |
| **509** | VLH | 3 | Serum Albumin | 1 | 368.23 | 367.22 | 57.06 | 485-487 | + |  | 1 |
| **510** | YSR | 3 | Serum Albumin | 1 | 425.21 | 424.21 | 63.57 | 17-19 | + |  | 1 |
| **511** | YST | 3 | Serum Albumin | 1 | 370.16 | 369.15 | 61.32 | 393-395 | + |  | 1 |

**Supplementary Table 3.** Milk peptides identified throughout the 28 days of storage period using the *Lb. helveticus* 11B2.

|  | **Sequence** | **Length** | **Leading proteins** | **Charge** | **m/z** | **Mass** | **Retention time** | **Sequence** | **Day 1** | **Day 28** | **Incidence** |
| --- | --- | --- | --- | --- | --- | --- | --- | --- | --- | --- | --- |
| **1** | APSFSDIPNPIGSENSEKTTMPLW | 24 | Alpha S1 Casein | 2 | 1309.63 | 2617.24 | 48.34 | 191-214 | + | + | 2 |
| **2** | DAPSFSDIPNPIGSENSEKTTMPLW | 25 | Alpha S1 Casein | 2 | 1367.14 | 2732.26 | 49.73 | 190-214 | + |  | 1 |
| **3** | DIPNPIGSENSEKTTMPLW | 19 | Alpha S1 Casein | 2 | 1065.01 | 2128.01 | 47.74 | 196-214 | + | + | 2 |
| **4** | ENLLRF | 6 | Alpha S1 Casein | 2 | 396.22 | 790.43 | 43.53 | 33-38 | + | + | 2 |
| **5** | EVLNENLLRF | 10 | Alpha S1 Casein | 2 | 623.84 | 1245.67 | 46.16 | 29-38 | + | + | 2 |
| **6** | FSDIPNPIGSENSEKTTMP | 19 | Alpha S1 Casein | 2 | 1032.48 | 2062.95 | 43.20 | 194-212 | + |  | 1 |
| **7** | FSDIPNPIGSENSEKTTMPLW | 21 | Alpha S1 Casein | 2 | 1182.06 | 2362.12 | 47.49 | 194-214 | + | + | 2 |
| **8** | FVAPFPEVFGK | 11 | Alpha S1 Casein | 2 | 619.33 | 1236.65 | 47.13 | 39-49 | + | + | 2 |
| **9** | GLPQEVLNENLLR | 13 | Alpha S1 Casein | 2 | 747.92 | 1493.82 | 45.04 | 25-37 | + |  | 1 |
| **10** | GLPQEVLNENLLRF | 14 | Alpha S1 Casein | 2 | 821.45 | 1640.89 | 48.84 | 25-38 | + |  | 1 |
| **11** | KHQGLPQEVL | 10 | Alpha S1 Casein | 2 | 574.82 | 1147.63 | 33.28 | 22-31 | + | + | 2 |
| **12** | NENLLRF | 7 | Alpha S1 Casein | 2 | 453.25 | 904.48 | 43.31 | 32-38 | + | + | 2 |
| **13** | NENLLRFFVAPFPE | 14 | Alpha S1 Casein | 2 | 846.94 | 1691.87 | 51.74 | 32-45 | + | + | 2 |
| **14** | QKEPMIGVNQEL | 12 | Alpha S1 Casein | 2 | 693.36 | 1384.70 | 41.41 | 146-157 | + | + | 2 |
| **15** | SDIPNPIGSENSEKTTMPLW | 20 | Alpha S1 Casein | 2 | 1108.53 | 2215.05 | 46.42 | 195-214 | + |  | 1 |
| **16** | SFSDIPNPIGSENSEKTTMPLW | 22 | Alpha S1 Casein | 2 | 1225.58 | 2449.15 | 47.96 | 193-214 | + | + | 2 |
| **17** | VFGKEKVNEL | 10 | Alpha S1 Casein | 2 | 581.83 | 1161.64 | 30.55 | 46-55 | + | + | 2 |
| **18** | WYY | 3 | Alpha S1 Casein | 1 | 531.22 | 530.22 | 43.64 | 179-181 | + | + | 2 |
| **19** | RLK | 3 | Alpha S1 Casein | 1 | 416.30 | 415.29 | 17.52 | 115-117 |  |  | 1 |
| **20** | DIKQMEAE | 8 | Alpha S1 Casein | 2 | 482.23 | 962.44 | 28.56 | 71-78 |  | + | 1 |
| **21** | DQAMEDIKQM | 10 | Alpha S1 Casein | 2 | 604.77 | 1207.52 | 38.02 | 66-75 |  | + | 1 |
| **22** | DQAMEDIKQME | 11 | Alpha S1 Casein | 2 | 669.29 | 1336.56 | 39.83 | 66-76 |  | + | 1 |
| **23** | DVPSERYL | 8 | Alpha S1 Casein | 2 | 489.75 | 977.48 | 38.13 | 100-107 |  | + | 1 |
| **24** | DVPSERYLG | 9 | Alpha S1 Casein | 2 | 518.26 | 1034.50 | 35.55 | 100-108 |  | + | 1 |
| **25** | DVPSERYLGYL | 11 | Alpha S1 Casein | 2 | 656.33 | 1310.65 | 45.48 | 100-110 |  | + | 1 |
| **26** | DVPSERYLGYLE | 12 | Alpha S1 Casein | 2 | 720.85 | 1439.69 | 44.80 | 100-111 |  | + | 1 |
| **27** | DVPSERYLGYLEQL | 14 | Alpha S1 Casein | 2 | 841.43 | 1680.84 | 48.70 | 100-113 |  | + | 1 |
| **28** | DVPSERYLGYLEQLL | 15 | Alpha S1 Casein | 2 | 897.97 | 1793.92 | 53.61 | 100-114 |  | + | 1 |
| **29** | EDIKQM | 6 | Alpha S1 Casein | 2 | 382.19 | 762.36 | 23.74 | 70-75 |  | + | 1 |
| **30** | EDV | 3 | Alpha S1 Casein | 1 | 362.16 | 361.15 | 65.28 | 99-101 |  | + | 1 |
| **31** | EKVNELSKD | 9 | Alpha S1 Casein | 2 | 531.28 | 1060.54 | 18.12 | 50-58 |  | + | 1 |
| **32** | ENLLRFF | 7 | Alpha S1 Casein | 2 | 469.76 | 937.50 | 47.89 | 33-39 |  | + | 1 |
| **33** | ENLLRFFVAPFPE | 13 | Alpha S1 Casein | 2 | 789.92 | 1577.82 | 52.06 | 33-45 |  | + | 1 |
| **34** | EVLNENLL | 8 | Alpha S1 Casein | 1 | 943.51 | 942.50 | 45.87 | 29-36 |  | + | 1 |
| **35** | EVLNENLLR | 9 | Alpha S1 Casein | 2 | 550.31 | 1098.60 | 41.67 | 29-37 |  | + | 1 |
| **36** | EVLNENLLRFF | 11 | Alpha S1 Casein | 2 | 697.38 | 1392.74 | 50.29 | 29-39 |  | + | 1 |
| **37** | FFVAPFPE | 8 | Alpha S1 Casein | 1 | 953.48 | 952.47 | 49.81 | 38-45 |  | + | 1 |
| **38** | FVAPFPEVFGKE | 12 | Alpha S1 Casein | 2 | 683.86 | 1365.70 | 47.18 | 39-50 |  | + | 1 |
| **39** | GKEKVNEL | 8 | Alpha S1 Casein | 2 | 458.76 | 915.50 | 17.95 | 48-55 |  | + | 1 |
| **40** | GKEKVNELS | 9 | Alpha S1 Casein | 2 | 502.27 | 1002.53 | 17.15 | 48-56 |  | + | 1 |
| **41** | GLPQEVLNENLLRFF | 15 | Alpha S1 Casein | 2 | 894.99 | 1787.96 | 52.54 | 25-39 |  | + | 1 |
| **42** | GSENSEKTTMPLW | 13 | Alpha S1 Casein | 2 | 740.34 | 1478.67 | 43.62 | 202-214 |  | + | 1 |
| **43** | GYLEQLLRLK | 10 | Alpha S1 Casein | 2 | 616.87 | 1231.73 | 45.29 | 108-117 |  | + | 1 |
| **44** | HIQKEDVPS | 9 | Alpha S1 Casein | 2 | 526.77 | 1051.53 | 18.71 | 95-103 |  | + | 1 |
| **45** | HIQKEDVPSER | 11 | Alpha S1 Casein | 3 | 446.57 | 1336.67 | 17.78 | 95-105 |  | + | 1 |
| **46** | IPNPIGSENSEKTTMPLW | 18 | Alpha S1 Casein | 2 | 1007.50 | 2012.99 | 45.84 | 197-214 |  | + | 1 |
| **47** | KEDVPSER | 8 | Alpha S1 Casein | 2 | 480.24 | 958.47 | 15.49 | 98-105 |  | + | 1 |
| **48** | KEDVPSERY | 9 | Alpha S1 Casein | 2 | 561.77 | 1121.54 | 21.94 | 98-106 |  | + | 1 |
| **49** | KEDVPSERYL | 10 | Alpha S1 Casein | 2 | 618.32 | 1234.62 | 31.48 | 98-107 |  | + | 1 |
| **50** | KHQGLPQEVLN | 11 | Alpha S1 Casein | 2 | 631.85 | 1261.68 | 30.91 | 22-32 |  | + | 1 |
| **51** | KHQGLPQEVLNE | 12 | Alpha S1 Casein | 2 | 696.37 | 1390.72 | 33.72 | 22-33 |  | + | 1 |
| **52** | KHQGLPQEVLNENLLR | 16 | Alpha S1 Casein | 4 | 472.77 | 1887.03 | 41.58 | 22-37 |  | + | 1 |
| **53** | KKYKVPQLE | 9 | Alpha S1 Casein | 2 | 566.84 | 1131.67 | 25.67 | 117-125 |  | + | 1 |
| **54** | KVNELSKD | 8 | Alpha S1 Casein | 2 | 466.76 | 931.50 | 14.99 | 51-58 |  | + | 1 |
| **55** | KYKVPQLE | 8 | Alpha S1 Casein | 2 | 502.79 | 1003.57 | 30.38 | 118-125 |  | + | 1 |
| **56** | LEQLLRLK | 8 | Alpha S1 Casein | 2 | 506.83 | 1011.64 | 38.98 | 110-117 |  | + | 1 |
| **57** | LFRQFYQLD | 9 | Alpha S1 Casein | 2 | 615.32 | 1228.62 | 44.07 | 164-172 |  | + | 1 |
| **58** | LKKYKVPQLE | 10 | Alpha S1 Casein | 2 | 623.38 | 1244.75 | 29.13 | 116-125 |  | + | 1 |
| **59** | LLRFFVAPFPE | 11 | Alpha S1 Casein | 2 | 668.38 | 1334.74 | 49.50 | 35-45 |  | + | 1 |
| **60** | LRLKKYKVPQLE | 12 | Alpha S1 Casein | 3 | 505.65 | 1513.93 | 32.28 | 114-125 |  | + | 1 |
| **61** | NENLLRFF | 8 | Alpha S1 Casein | 2 | 526.78 | 1051.55 | 47.59 | 32-39 |  | + | 1 |
| **62** | NLLRFFVAPFPE | 12 | Alpha S1 Casein | 2 | 725.40 | 1448.78 | 51.01 | 34-45 |  | + | 1 |
| **63** | NSEKTTMPLW | 10 | Alpha S1 Casein | 2 | 603.79 | 1205.58 | 43.47 | 205-214 |  | + | 1 |
| **64** | QAMEDIKQ | 8 | Alpha S1 Casein | 2 | 481.73 | 961.45 | 23.67 | 67-74 |  | + | 1 |
| **65** | QAMEDIKQM | 9 | Alpha S1 Casein | 2 | 547.25 | 1092.49 | 32.87 | 67-75 |  | + | 1 |
| **66** | QAMEDIKQME | 10 | Alpha S1 Casein | 2 | 611.78 | 1221.54 | 34.35 | 67-76 |  | + | 1 |
| **67** | QAMEDIKQMEAE | 12 | Alpha S1 Casein | 2 | 711.82 | 1421.62 | 36.71 | 67-78 |  | + | 1 |
| **68** | QKEPMIGVN | 9 | Alpha S1 Casein | 2 | 508.27 | 1014.52 | 31.43 | 146-154 |  | + | 1 |
| **69** | QKEPMIGVNQ | 10 | Alpha S1 Casein | 2 | 572.29 | 1142.58 | 31.07 | 146-155 |  | + | 1 |
| **70** | QKEPMIGVNQE | 11 | Alpha S1 Casein | 2 | 636.82 | 1271.62 | 31.76 | 146-156 |  | + | 1 |
| **71** | QLLRLKK | 7 | Alpha S1 Casein | 2 | 449.81 | 897.61 | 22.08 | 112-118 |  | + | 1 |
| **72** | RFFVAPFPE | 9 | Alpha S1 Casein | 2 | 555.29 | 1108.57 | 45.68 | 37-45 |  | + | 1 |
| **73** | RLKKYKVPQLE | 11 | Alpha S1 Casein | 3 | 467.96 | 1400.85 | 26.39 | 115-125 |  | + | 1 |
| **74** | SMKEGIHAQ | 9 | Alpha S1 Casein | 2 | 500.75 | 999.48 | 17.01 | 137-145 |  | + | 1 |
| **75** | SVEQKHIQ | 8 | Alpha S1 Casein | 2 | 484.76 | 967.51 | 14.46 | 90-97 |  | + | 1 |
| **76** | VAPFPEVFGK | 10 | Alpha S1 Casein | 2 | 545.80 | 1089.59 | 44.69 | 40-49 |  | + | 1 |
| **77** | VAPFPEVFGKE | 11 | Alpha S1 Casein | 2 | 610.32 | 1218.63 | 44.70 | 40-50 |  | + | 1 |
| **78** | VFGKEKVN | 8 | Alpha S1 Casein | 2 | 460.76 | 919.51 | 18.61 | 46-53 |  | + | 1 |
| **79** | VNELSKDIGSE | 11 | Alpha S1 Casein | 2 | 595.80 | 1189.58 | 31.93 | 52-62 |  | + | 1 |
| **80** | YLE | 3 | Alpha S1 Casein | 1 | 424.21 | 423.20 | 24.98 | 109-111 |  | + | 1 |
| **81** | KFF | 3 | Alpha S2 Casein | 1 | 441.25 | 440.24 | 27.66 | 2-4 | + | + | 2 |
| **82** | LYQGPIVLNPWDQVK | 15 | Alpha S2 Casein | 2 | 885.48 | 1768.95 | 46.56 | 114-128 | + | + | 2 |
| **83** | LYQGPIVLNPWDQVKR | 16 | Alpha S2 Casein | 2 | 963.53 | 1925.05 | 44.41 | 114-129 | + | + | 2 |
| **84** | LYQGPIVLNPWDQVKRN | 17 | Alpha S2 Casein | 3 | 680.71 | 2039.10 | 44.35 | 114-130 | + | + | 2 |
| **85** | QGPIVLNPWDQVK | 13 | Alpha S2 Casein | 2 | 747.41 | 1492.80 | 44.89 | 116-128 | + |  | 1 |
| **86** | QGPIVLNPWDQVKRN | 15 | Alpha S2 Casein | 2 | 882.48 | 1762.95 | 42.86 | 116-130 | + | + | 2 |
| **87** | QYLYQGPIVLNPWDQVKRN | 19 | Alpha S2 Casein | 3 | 777.75 | 2330.22 | 45.24 | 112-130 | + | + | 2 |
| **88** | YLYQGPIVLNPWDQVKRN | 18 | Alpha S2 Casein | 2 | 1102.09 | 2202.16 | 45.18 | 113-130 | + | + | 2 |
| **89** | YQGPIVLNPWDQVK | 14 | Alpha S2 Casein | 2 | 828.94 | 1655.87 | 45.63 | 115-128 | + | + | 2 |
| **90** | YQGPIVLNPWDQVKR | 15 | Alpha S2 Casein | 3 | 605.00 | 1811.97 | 43.64 | 115-129 | + | + | 2 |
| **91** | YQGPIVLNPWDQVKRN | 16 | Alpha S2 Casein | 2 | 964.01 | 1926.01 | 43.60 | 115-130 | + | + | 2 |
| **92** | AINPSKEN | 8 | Alpha S2 Casein | 2 | 436.73 | 871.44 | 16.08 | 42-49 |  | + | 1 |
| **93** | ALPQYLKTVY | 10 | Alpha S2 Casein | 2 | 598.34 | 1194.66 | 43.26 | 190-199 |  | + | 1 |
| **94** | DKHYQKALNEIN | 12 | Alpha S2 Casein | 3 | 491.59 | 1471.74 | 24.60 | 90-101 |  | + | 1 |
| **95** | DQVKRNAVPITPT | 13 | Alpha S2 Casein | 2 | 719.90 | 1437.79 | 29.65 | 125-137 |  | + | 1 |
| **96** | EEY | 3 | Alpha S2 Casein | 1 | 440.17 | 439.16 | 38.67 | 65-67 |  | + | 1 |
| **97** | EVVRNANEE | 9 | Alpha S2 Casein | 2 | 530.26 | 1058.50 | 17.05 | 57-65 |  | + | 1 |
| **98** | FLKKISQRY | 9 | Alpha S2 Casein | 3 | 394.90 | 1181.69 | 24.60 | 178-186 |  | + | 1 |
| **99** | LNREQL | 6 | Alpha S2 Casein | 2 | 386.72 | 771.42 | 24.23 | 138-143 |  | + | 1 |
| **100** | LYQGPIVLNPWDQVKRNAVPITPT | 24 | Alpha S2 Casein | 3 | 907.17 | 2718.49 | 45.70 | 114-137 |  | + | 1 |
| **101** | NEE | 3 | Alpha S2 Casein | 1 | 391.15 | 390.14 | 32.32 | 63-65 |  | + | 1 |
| **102** | NSKKTVDME | 9 | Alpha S2 Casein | 2 | 526.26 | 1050.50 | 15.19 | 149-157 |  | + | 1 |
| **103** | PWD | 3 | Alpha S2 Casein | 1 | 417.18 | 416.17 | 28.46 | 123-125 |  | + | 1 |
| **104** | QFYQKFPQYL | 10 | Alpha S2 Casein | 2 | 681.35 | 1360.68 | 44.19 | 102-111 |  | + | 1 |
| **105** | QYLYQGPIVLNPWDQVKR | 18 | Alpha S2 Casein | 3 | 739.73 | 2216.17 | 45.31 | 112-129 |  | + | 1 |
| **106** | SKKTVDME | 8 | Alpha S2 Casein | 2 | 469.24 | 936.46 | 15.43 | 150-157 |  | + | 1 |
| **107** | TKVIPYVRYL | 10 | Alpha S2 Casein | 2 | 626.38 | 1250.74 | 42.36 | 213-222 |  | + | 1 |
| **108** | TYK | 3 | Alpha S2 Casein | 1 | 411.22 | 410.22 | 29.93 | 34-36 |  | + | 1 |
| **109** | VFTKKTKLTEE | 11 | Alpha S2 Casein | 2 | 662.38 | 1322.74 | 20.53 | 161-171 |  | + | 1 |
| **110** | VKITVDDKHYQK | 12 | Alpha S2 Casein | 3 | 491.94 | 1472.80 | 22.07 | 84-95 |  | + | 1 |
| **111** | VVRNANEE | 8 | Alpha S2 Casein | 2 | 465.74 | 929.46 | 13.85 | 58-65 |  | + | 1 |
| **112** | YLKTVYQH | 8 | Alpha S2 Casein | 2 | 526.28 | 1050.55 | 24.50 | 194-201 |  | + | 1 |
| **113** | AQTQSLVYPFPGPIPN | 16 | Beta Casein | 2 | 864.95 | 1727.89 | 48.68 | 68-83 | + | + | 2 |
| **114** | DELQDKIHP | 9 | Beta Casein | 2 | 547.78 | 1093.54 | 29.50 | 58-66 | + |  | 1 |
| **115** | DELQDKIHPF | 10 | Beta Casein | 2 | 621.31 | 1240.61 | 40.43 | 58-67 | + | + | 2 |
| **116** | DELQDKIHPFAQTQ | 14 | Beta Casein | 2 | 835.41 | 1668.81 | 40.64 | 58-71 | + | + | 2 |
| **117** | DELQDKIHPFAQTQSL | 16 | Beta Casein | 2 | 935.47 | 1868.93 | 42.77 | 58-73 | + |  | 1 |
| **118** | DKIHPF | 6 | Beta Casein | 2 | 378.71 | 755.40 | 28.33 | 62-67 | + | + | 2 |
| **119** | DKIHPFAQT | 9 | Beta Casein | 2 | 528.78 | 1055.54 | 28.54 | 62-70 | + | + | 2 |
| **120** | DKIHPFAQTQ | 10 | Beta Casein | 2 | 592.81 | 1183.60 | 27.75 | 62-71 | + | + | 2 |
| **121** | DKIHPFAQTQSL | 12 | Beta Casein | 2 | 692.86 | 1383.71 | 37.24 | 62-73 | + | + | 2 |
| **122** | DVENLHLPLPL | 11 | Beta Casein | 2 | 630.35 | 1258.69 | 64.44 | 144-154 | + | + | 2 |
| **123** | DVENLHLPLPLL | 12 | Beta Casein | 2 | 686.90 | 1371.78 | 52.85 | 144-155 | + | + | 2 |
| **124** | DVENLHLPLPLLQ | 13 | Beta Casein | 2 | 750.92 | 1499.83 | 51.69 | 144-156 | + | + | 2 |
| **125** | DVENLHLPLPLLQS | 14 | Beta Casein | 2 | 794.44 | 1586.87 | 51.20 | 144-157 | + | + | 2 |
| **126** | DVENLHLPLPLLQSW | 15 | Beta Casein | 2 | 887.48 | 1772.95 | 54.45 | 144-158 | + | + | 2 |
| **127** | EDELQDKIHPF | 11 | Beta Casein | 2 | 685.83 | 1369.65 | 41.41 | 57-67 | + | + | 2 |
| **128** | EEL | 3 | Beta Casein | 1 | 390.19 | 389.18 | 52.15 | 19-21 | + |  | 1 |
| **129** | ELQDKIHP | 8 | Beta Casein | 2 | 490.26 | 978.51 | 25.18 | 59-66 | + | + | 2 |
| **130** | ELQDKIHPF | 9 | Beta Casein | 2 | 563.80 | 1125.58 | 36.32 | 59-67 | + | + | 2 |
| **131** | ELQDKIHPFAQTQ | 13 | Beta Casein | 2 | 777.90 | 1553.78 | 36.01 | 59-71 | + |  | 1 |
| **132** | ELQDKIHPFAQTQSL | 15 | Beta Casein | 2 | 877.96 | 1753.90 | 41.47 | 59-73 | + |  | 1 |
| **133** | EMPFPKYPVEPF | 12 | Beta Casein | 2 | 740.86 | 1479.71 | 46.72 | 123-134 | + | + | 2 |
| **134** | EMPFPKYPVEPFTES | 15 | Beta Casein | 2 | 899.42 | 1796.83 | 45.86 | 123-137 | + | + | 2 |
| **135** | EPVLGPVRGPFPIIV | 15 | Beta Casein | 2 | 795.47 | 1588.93 | 49.73 | 210-224 | + | + | 2 |
| **136** | EQQQTEDELQ | 10 | Beta Casein | 2 | 624.27 | 1246.53 | 27.04 | 52-61 | + | + | 2 |
| **137** | EQQQTEDELQDK | 12 | Beta Casein | 2 | 745.83 | 1489.65 | 23.05 | 52-63 | + | + | 2 |
| **138** | EQQQTEDELQDKIHPF | 16 | Beta Casein | 3 | 662.31 | 1983.92 | 40.00 | 52-67 | + | + | 2 |
| **139** | EQQQTEDELQDKIHPFAQTQ | 20 | Beta Casein | 3 | 805.05 | 2412.12 | 40.96 | 52-71 | + |  | 1 |
| **140** | EQQQTEDELQDKIHPFAQTQSL | 22 | Beta Casein | 2 | 1307.13 | 2612.24 | 42.58 | 52-73 | + |  | 1 |
| **141** | FLLYQEPVLGPVRGPFPIIV | 20 | Beta Casein | 2 | 1127.65 | 2253.29 | 53.21 | 205-224 | + | + | 2 |
| **142** | GPVRGPFPIIV | 11 | Beta Casein | 2 | 576.35 | 1150.69 | 46.67 | 214-224 | + | + | 2 |
| **143** | GVSKVKEA | 8 | Beta Casein | 2 | 409.24 | 816.47 | 8.90 | 109-116 | + | + | 2 |
| **144** | HKEMPFPKYPVEPF | 14 | Beta Casein | 3 | 582.63 | 1744.86 | 42.45 | 121-134 | + | + | 2 |
| **145** | HKEMPFPKYPVEPFTES | 17 | Beta Casein | 3 | 688.34 | 2061.99 | 42.18 | 121-137 | + | + | 2 |
| **146** | HLPLPLLQ | 8 | Beta Casein | 2 | 465.79 | 929.57 | 46.28 | 149-156 | + | + | 2 |
| **147** | HQPHQPLPPTVMFPPQ | 16 | Beta Casein | 3 | 617.65 | 1849.93 | 42.36 | 160-175 | + | + | 2 |
| **148** | HQPHQPLPPTVMFPPQSVL | 19 | Beta Casein | 2 | 1075.56 | 2149.11 | 45.12 | 160-178 | + | + | 2 |
| **149** | KAVPYPQ | 7 | Beta Casein | 2 | 401.73 | 801.44 | 24.72 | 191-197 | + | + | 2 |
| **150** | KAVPYPQRDMPIQ | 13 | Beta Casein | 2 | 771.91 | 1541.80 | 33.27 | 191-203 | + | + | 2 |
| **151** | KAVPYPQRDMPIQA | 14 | Beta Casein | 2 | 807.43 | 1612.84 | 35.32 | 191-204 | + | + | 2 |
| **152** | KEMPFPKYPVEPF | 13 | Beta Casein | 2 | 804.91 | 1607.81 | 43.75 | 122-134 | + | + | 2 |
| **153** | KVLPVPQ | 7 | Beta Casein | 2 | 390.75 | 779.49 | 32.52 | 184-190 | + | + | 2 |
| **154** | KVLPVPQKAVPYPQ | 14 | Beta Casein | 3 | 521.98 | 1562.92 | 36.88 | 184-197 | + | + | 2 |
| **155** | LGPVRGPFPIIV | 12 | Beta Casein | 2 | 632.89 | 1263.77 | 48.12 | 213-224 | + | + | 2 |
| **156** | LHL | 3 | Beta Casein | 1 | 382.24 | 381.24 | 56.87 | 148-150 | + |  | 1 |
| **157** | LHLPLPLLQ | 9 | Beta Casein | 2 | 522.33 | 1042.65 | 49.01 | 148-156 | + | + | 2 |
| **158** | LLYQEPVLGPVRGPFP | 16 | Beta Casein | 2 | 891.50 | 1780.99 | 47.02 | 206-221 | + | + | 2 |
| **159** | LLYQEPVLGPVRGPFPI | 17 | Beta Casein | 2 | 948.04 | 1894.07 | 49.17 | 206-222 | + | + | 2 |
| **160** | LLYQEPVLGPVRGPFPII | 18 | Beta Casein | 2 | 1004.59 | 2007.16 | 50.75 | 206-223 | + | + | 2 |
| **161** | LLYQEPVLGPVRGPFPIIV | 19 | Beta Casein | 2 | 1054.12 | 2106.22 | 51.47 | 206-224 | + | + | 2 |
| **162** | LQSWMHQPHQPLPPT | 15 | Beta Casein | 3 | 599.63 | 1795.88 | 38.31 | 155-169 | + | + | 2 |
| **163** | LQSWMHQPHQPLPPTVMFPPQ | 21 | Beta Casein | 3 | 832.75 | 2495.22 | 44.35 | 155-175 | + | + | 2 |
| **164** | LQSWMHQPHQPLPPTVMFPPQSVL | 24 | Beta Casein | 3 | 932.48 | 2794.41 | 46.56 | 155-178 | + | + | 2 |
| **165** | LSLSQSKVLPVPQ | 13 | Beta Casein | 2 | 698.41 | 1394.81 | 42.92 | 178-190 | + |  | 1 |
| **166** | LYQEPVLGPVRGPFP | 15 | Beta Casein | 2 | 834.96 | 1667.90 | 45.59 | 207-221 | + |  | 1 |
| **167** | LYQEPVLGPVRGPFPII | 17 | Beta Casein | 2 | 948.04 | 1894.07 | 49.63 | 207-223 | + | + | 2 |
| **168** | LYQEPVLGPVRGPFPIIV | 18 | Beta Casein | 2 | 997.58 | 1993.14 | 50.44 | 207-224 | + | + | 2 |
| **169** | MAPKHKEMPFPKYPVEPF | 18 | Beta Casein | 3 | 725.04 | 2172.09 | 42.43 | 117-134 | + | + | 2 |
| **170** | MHQPHQPLPPTVMFPPQ | 17 | Beta Casein | 3 | 661.33 | 1980.97 | 42.73 | 159-175 | + | + | 2 |
| **171** | MPFPKYPVEPF | 11 | Beta Casein | 2 | 676.34 | 1350.67 | 46.02 | 124-134 | + | + | 2 |
| **172** | NIPPLTQTPV | 10 | Beta Casein | 2 | 540.31 | 1078.60 | 44.49 | 88-97 | + | + | 2 |
| **173** | NIPPLTQTPVVVPP | 14 | Beta Casein | 2 | 736.43 | 1470.84 | 47.29 | 88-101 | + |  | 1 |
| **174** | NIPPLTQTPVVVPPFLQPE | 19 | Beta Casein | 2 | 1043.58 | 2085.15 | 53.10 | 88-106 | + | + | 2 |
| **175** | NIPPLTQTPVVVPPFLQPEVM | 21 | Beta Casein | 2 | 1166.63 | 2331.25 | 53.49 | 88-108 | + | + | 2 |
| **176** | NIPPLTQTPVVVPPFLQPEVMGV | 23 | Beta Casein | 2 | 1236.68 | 2471.35 | 55.03 | 88-110 | + | + | 2 |
| **177** | NIPPLTQTPVVVPPFLQPEVMGVS | 24 | Beta Casein | 2 | 1280.20 | 2558.38 | 54.92 | 88-111 | + | + | 2 |
| **178** | NIPPLTQTPVVVPPFLQPEVMGVSK | 25 | Beta Casein | 3 | 896.50 | 2686.48 | 51.30 | 88-112 | + | + | 2 |
| **179** | NIPPLTQTPVVVPPFLQPEVMGVSKV | 26 | Beta Casein | 2 | 1393.78 | 2785.55 | 51.97 | 88-113 | + | + | 2 |
| **180** | NIPPLTQTPVVVPPFLQPEVMGVSKVK | 27 | Beta Casein | 3 | 972.22 | 2913.64 | 49.17 | 88-114 | + | + | 2 |
| **181** | NLHLPLPL | 8 | Beta Casein | 2 | 458.78 | 915.55 | 47.63 | 147-154 | + | + | 2 |
| **182** | NLHLPLPLL | 9 | Beta Casein | 2 | 515.33 | 1028.64 | 50.07 | 147-155 | + | + | 2 |
| **183** | NLHLPLPLLQ | 10 | Beta Casein | 2 | 579.36 | 1156.70 | 49.31 | 147-156 | + | + | 2 |
| **184** | PPFLQPEVMGVSKVKEA | 17 | Beta Casein | 2 | 928.50 | 1854.99 | 52.36 | 100-116 | + |  | 1 |
| **185** | PPLTQTPVVVPPFLQPEVMGVS | 22 | Beta Casein | 2 | 1166.63 | 2331.25 | 53.49 | 90-111 | + |  | 1 |
| **186** | QDKIHPFAQTQ | 11 | Beta Casein | 2 | 656.84 | 1311.66 | 27.20 | 61-71 | + |  | 1 |
| **187** | QDKIHPFAQTQSL | 13 | Beta Casein | 2 | 756.89 | 1511.77 | 36.17 | 61-73 | + |  | 1 |
| **188** | QEPVLGPV | 8 | Beta Casein | 1 | 838.47 | 837.46 | 43.52 | 209-216 | + | + | 2 |
| **189** | QEPVLGPVRGPFP | 13 | Beta Casein | 2 | 696.89 | 1391.76 | 44.38 | 209-221 | + | + | 2 |
| **190** | QEPVLGPVRGPFPII | 15 | Beta Casein | 2 | 809.97 | 1617.92 | 48.51 | 209-223 | + | + | 2 |
| **191** | QEPVLGPVRGPFPIIV | 16 | Beta Casein | 2 | 859.50 | 1716.99 | 49.39 | 209-224 | + | + | 2 |
| **192** | QSEEQQQTEDELQDK | 15 | Beta Casein | 2 | 917.90 | 1833.79 | 26.78 | 49-63 | + | + | 2 |
| **193** | QSEEQQQTEDELQDKIHPF | 19 | Beta Casein | 3 | 777.02 | 2328.05 | 41.98 | 49-67 | + | + | 2 |
| **194** | QSEEQQQTEDELQDKIHPFAQTQ | 23 | Beta Casein | 3 | 919.76 | 2756.25 | 41.57 | 49-71 | + |  | 1 |
| **195** | QSKVLPVPQ | 9 | Beta Casein | 2 | 498.30 | 994.58 | 34.00 | 182-190 | + | + | 2 |
| **196** | QSKVLPVPQKAVPYPQ | 16 | Beta Casein | 3 | 593.68 | 1778.01 | 37.86 | 182-197 | + | + | 2 |
| **197** | QSLVYPFPGPIPN | 13 | Beta Casein | 2 | 714.88 | 1427.74 | 49.10 | 71-83 | + | + | 2 |
| **198** | QTEDELQDK | 9 | Beta Casein | 2 | 553.25 | 1104.49 | 20.65 | 55-63 | + | + | 2 |
| **199** | QTEDELQDKIHPF | 13 | Beta Casein | 2 | 800.39 | 1598.76 | 41.22 | 55-67 | + | + | 2 |
| **200** | QTEDELQDKIHPFAQTQ | 17 | Beta Casein | 2 | 1014.49 | 2026.96 | 40.19 | 55-71 | + |  | 1 |
| **201** | QTEDELQDKIHPFAQTQS | 18 | Beta Casein | 2 | 1058.00 | 2113.99 | 37.56 | 55-72 | + |  | 1 |
| **202** | QTPVVVPPFLQPEVM | 15 | Beta Casein | 2 | 840.96 | 1679.90 | 52.73 | 94-108 | + |  | 1 |
| **203** | RDMPIQAF | 8 | Beta Casein | 2 | 489.25 | 976.48 | 42.23 | 198-205 | + | + | 2 |
| **204** | RDMPIQAFL | 9 | Beta Casein | 2 | 545.79 | 1089.56 | 46.31 | 198-206 | + | + | 2 |
| **205** | RDMPIQAFLL | 10 | Beta Casein | 2 | 602.33 | 1202.65 | 48.85 | 198-207 | + | + | 2 |
| **206** | RDMPIQAFLLY | 11 | Beta Casein | 2 | 683.86 | 1365.71 | 49.47 | 198-208 | + | + | 2 |
| **207** | RGPFPIIV | 8 | Beta Casein | 2 | 449.78 | 897.54 | 45.61 | 217-224 | + | + | 2 |
| **208** | SLPQNIPPL | 9 | Beta Casein | 2 | 489.78 | 977.55 | 46.04 | 84-92 | + | + | 2 |
| **209** | SLPQNIPPLTQTPV | 14 | Beta Casein | 2 | 752.92 | 1503.83 | 46.11 | 84-97 | + | + | 2 |
| **210** | SLPQNIPPLTQTPVVVPP | 18 | Beta Casein | 2 | 949.04 | 1896.07 | 48.34 | 84-101 | + |  | 1 |
| **211** | SLPQNIPPLTQTPVVVPPFLQPEVM | 25 | Beta Casein | 3 | 914.50 | 2740.49 | 54.86 | 84-108 | + | + | 2 |
| **212** | SLSQSKVLPVPQ | 12 | Beta Casein | 2 | 641.87 | 1281.73 | 39.66 | 179-190 | + | + | 2 |
| **213** | SLSQSKVLPVPQK | 13 | Beta Casein | 2 | 705.92 | 1409.82 | 32.02 | 179-191 | + |  | 1 |
| **214** | SLSQSKVLPVPQKAVPYPQ | 19 | Beta Casein | 3 | 689.39 | 2065.16 | 40.96 | 179-197 | + | + | 2 |
| **215** | SLVYPFPGPIPN | 12 | Beta Casein | 2 | 650.85 | 1299.69 | 49.50 | 72-83 | + | + | 2 |
| **216** | SQSKVLPVPQ | 10 | Beta Casein | 2 | 541.81 | 1081.61 | 34.26 | 181-190 | + | + | 2 |
| **217** | SQSKVLPVPQKAVPYPQ | 17 | Beta Casein | 2 | 933.53 | 1865.04 | 38.04 | 181-197 | + | + | 2 |
| **218** | SVLSLSQSKVLPVPQ | 15 | Beta Casein | 2 | 791.46 | 1580.91 | 44.30 | 176-190 | + | + | 2 |
| **219** | SVLSLSQSKVLPVPQKAVPYPQ | 22 | Beta Casein | 3 | 789.12 | 2364.34 | 43.42 | 176-197 | + | + | 2 |
| **220** | SWMHQPHQPLPPT | 13 | Beta Casein | 2 | 778.38 | 1554.74 | 34.95 | 157-169 | + | + | 2 |
| **221** | SWMHQPHQPLPPTVMFPPQ | 19 | Beta Casein | 3 | 752.37 | 2254.08 | 43.81 | 157-175 | + | + | 2 |
| **222** | SWMHQPHQPLPPTVMFPPQSVL | 22 | Beta Casein | 3 | 852.10 | 2553.27 | 46.12 | 157-178 | + | + | 2 |
| **223** | TDVENLHLPLPL | 12 | Beta Casein | 2 | 680.88 | 1359.74 | 49.17 | 143-154 | + | + | 2 |
| **224** | TDVENLHLPLPLLQ | 14 | Beta Casein | 2 | 801.45 | 1600.88 | 50.40 | 143-156 | + | + | 2 |
| **225** | TEDELQDKIHPF | 12 | Beta Casein | 2 | 736.36 | 1470.70 | 41.25 | 56-67 | + | + | 2 |
| **226** | TEDELQDKIHPFAQTQ | 16 | Beta Casein | 2 | 950.46 | 1898.90 | 40.17 | 56-71 | + |  | 1 |
| **227** | TESQSLTLTDVENLHLPLPLLQ | 22 | Beta Casein | 2 | 1231.16 | 2460.31 | 53.15 | 135-156 | + | + | 2 |
| **228** | TLTDVENLHLPLPL | 14 | Beta Casein | 2 | 787.94 | 1573.87 | 50.65 | 141-154 | + |  | 1 |
| **229** | TLTDVENLHLPLPLLQ | 16 | Beta Casein | 2 | 908.51 | 1815.01 | 51.69 | 141-156 | + | + | 2 |
| **230** | TPVVVPPFLQPEVM | 14 | Beta Casein | 2 | 776.93 | 1551.84 | 52.92 | 95-108 | + | + | 2 |
| **231** | TQTPVVVPPFL | 11 | Beta Casein | 2 | 599.35 | 1196.68 | 51.74 | 93-103 | + |  | 1 |
| **232** | TQTPVVVPPFLQPE | 14 | Beta Casein | 2 | 776.42 | 1550.83 | 50.27 | 93-106 | + | + | 2 |
| **233** | TQTPVVVPPFLQPEVM | 16 | Beta Casein | 2 | 891.48 | 1780.94 | 52.71 | 93-108 | + | + | 2 |
| **234** | TQTPVVVPPFLQPEVMG | 17 | Beta Casein | 2 | 919.99 | 1837.96 | 52.37 | 93-109 | + |  | 1 |
| **235** | TQTPVVVPPFLQPEVMGVS | 19 | Beta Casein | 2 | 1013.04 | 2024.07 | 53.02 | 93-111 | + |  | 1 |
| **236** | TQTPVVVPPFLQPEVMGVSK | 20 | Beta Casein | 2 | 1077.09 | 2152.16 | 48.96 | 93-112 | + | + | 2 |
| **237** | VENLHLPLPL | 10 | Beta Casein | 2 | 572.84 | 1143.67 | 47.97 | 145-154 | + | + | 2 |
| **238** | VENLHLPLPLLQ | 12 | Beta Casein | 2 | 693.41 | 1384.81 | 49.45 | 145-156 | + | + | 2 |
| **239** | VLGPVRGPFPIIV | 13 | Beta Casein | 2 | 682.43 | 1362.84 | 48.62 | 212-224 | + | + | 2 |
| **240** | VLPVPQKAVPYPQ | 13 | Beta Casein | 2 | 718.42 | 1434.82 | 41.57 | 185-197 | + | + | 2 |
| **241** | VLSLSQSKVLPVPQ | 14 | Beta Casein | 2 | 747.95 | 1493.88 | 43.63 | 177-190 | + | + | 2 |
| **242** | VMFPPQSVL | 9 | Beta Casein | 2 | 509.28 | 1016.54 | 48.06 | 170-178 | + | + | 2 |
| **243** | VPPFLQPEVM | 10 | Beta Casein | 1 | 1156.61 | 1155.60 | 49.16 | 99-108 | + |  | 1 |
| **244** | VYP | 3 | Beta Casein | 1 | 378.20 | 377.20 | 29.32 | 74-76 | + |  | 1 |
| **245** | VYPFPGPIPN | 10 | Beta Casein | 2 | 550.79 | 1099.57 | 47.15 | 74-83 | + | + | 2 |
| **246** | VYPFPGPIPNSLPQ | 14 | Beta Casein | 2 | 763.41 | 1524.80 | 48.73 | 74-87 | + | + | 2 |
| **247** | YPFPGPIPNSLPQ | 13 | Beta Casein | 2 | 713.87 | 1425.73 | 47.80 | 75-87 | + |  | 1 |
| **248** | YPV | 3 | Beta Casein | 1 | 378.20 | 377.20 | 29.23 | 129-131 | + |  | 1 |
| **249** | YQEPVLGPVRGPFP | 14 | Beta Casein | 2 | 778.42 | 1554.82 | 44.95 | 208-221 | + | + | 2 |
| **250** | YQEPVLGPVRGPFPI | 15 | Beta Casein | 2 | 834.96 | 1667.90 | 47.26 | 208-222 | + | + | 2 |
| **251** | YQEPVLGPVRGPFPII | 16 | Beta Casein | 2 | 891.50 | 1780.99 | 49.01 | 208-223 | + | + | 2 |
| **252** | YQEPVLGPVRGPFPIIV | 17 | Beta Casein | 2 | 941.04 | 1880.06 | 49.83 | 208-224 | + | + | 2 |
| **253** | APKHKEMPFPKYPVEPF | 17 | Beta Casein | 3 | 681.36 | 2041.05 | 41.16 | 118-134 |  | + | 1 |
| **254** | AVPYPQRDMPIQ | 12 | Beta Casein | 2 | 707.86 | 1413.71 | 39.53 | 192-203 |  | + | 1 |
| **255** | DKIHPFAQTQS | 11 | Beta Casein | 2 | 636.32 | 1270.63 | 27.43 | 62-72 |  | + | 1 |
| **256** | DMPIQAFLL | 9 | Beta Casein | 2 | 524.28 | 1046.55 | 55.26 | 199-207 |  | + | 1 |
| **257** | EAMAPKHKEMPFPKYPVEPF | 20 | Beta Casein | 3 | 791.73 | 2372.17 | 41.84 | 115-134 |  | + | 1 |
| **258** | EDE | 3 | Beta Casein | 1 | 392.13 | 391.12 | 45.54 | 57-59 |  | + | 1 |
| **259** | EEQQQTEDELQ | 11 | Beta Casein | 2 | 688.79 | 1375.57 | 28.26 | 51-61 |  | + | 1 |
| **260** | ENLHLPLPL | 9 | Beta Casein | 2 | 523.31 | 1044.60 | 47.78 | 146-154 |  | + | 1 |
| **261** | EQQQTEDELQD | 11 | Beta Casein | 2 | 681.79 | 1361.56 | 28.40 | 52-62 |  | + | 1 |
| **262** | FPKYPVEPF | 9 | Beta Casein | 2 | 562.29 | 1122.57 | 44.26 | 126-134 |  | + | 1 |
| **263** | GVSKVKEAM | 9 | Beta Casein | 2 | 474.76 | 947.51 | 19.01 | 109-117 |  | + | 1 |
| **264** | GVSKVKEAMAPK | 12 | Beta Casein | 3 | 415.57 | 1243.70 | 18.74 | 109-120 |  | + | 1 |
| **265** | GVSKVKEAMAPKHKEMPFPKYPVEPF | 26 | Beta Casein | 4 | 743.64 | 2970.55 | 41.16 | 109-134 |  | + | 1 |
| **266** | HKEMPFPKYPVEPFTE | 16 | Beta Casein | 3 | 659.33 | 1974.95 | 42.30 | 121-136 |  | + | 1 |
| **267** | HKEMPFPKYPVEPFTESQ | 18 | Beta Casein | 3 | 731.02 | 2190.05 | 42.07 | 121-138 |  | + | 1 |
| **268** | HQPHQPLPPT | 10 | Beta Casein | 2 | 576.30 | 1150.59 | 22.85 | 160-169 |  | + | 1 |
| **269** | HQPHQPLPPTVMFPPQSVLSLS | 22 | Beta Casein | 3 | 813.09 | 2436.26 | 46.60 | 160-181 |  | + | 1 |
| **270** | IHPFAQTQ | 8 | Beta Casein | 2 | 471.25 | 940.48 | 29.19 | 64-71 |  | + | 1 |
| **271** | IPPLTQTPVVVPPFLQPEVM | 20 | Beta Casein | 2 | 1101.62 | 2201.22 | 54.97 | 89-108 |  | + | 1 |
| **272** | KAVPYPQR | 8 | Beta Casein | 2 | 479.78 | 957.54 | 19.71 | 191-198 |  | + | 1 |
| **273** | KAVPYPQRD | 9 | Beta Casein | 2 | 537.29 | 1072.57 | 20.25 | 191-199 |  | + | 1 |
| **274** | KAVPYPQRDMPIQAF | 15 | Beta Casein | 3 | 587.64 | 1759.91 | 42.13 | 191-205 |  | + | 1 |
| **275** | KAVPYPQRDMPIQAFL | 16 | Beta Casein | 3 | 625.34 | 1872.99 | 44.80 | 191-206 |  | + | 1 |
| **276** | KIEKFQ | 6 | Beta Casein | 2 | 396.73 | 791.45 | 18.22 | 44-49 |  | + | 1 |
| **277** | KIHPFAQTQ | 9 | Beta Casein | 2 | 535.29 | 1068.57 | 23.35 | 63-71 |  | + | 1 |
| **278** | KKIEKF | 6 | Beta Casein | 2 | 396.75 | 791.49 | 14.66 | 43-48 |  | + | 1 |
| **279** | KKIEKFQ | 7 | Beta Casein | 2 | 460.78 | 919.55 | 14.48 | 43-49 |  | + | 1 |
| **280** | KVKEAMAPK | 9 | Beta Casein | 2 | 501.29 | 1000.57 | 14.13 | 112-120 |  | + | 1 |
| **281** | KVLPVPQK | 8 | Beta Casein | 2 | 454.80 | 907.59 | 24.27 | 184-191 |  | + | 1 |
| **282** | LHLPLPL | 7 | Beta Casein | 2 | 401.76 | 801.51 | 47.40 | 148-154 |  | + | 1 |
| **283** | LHLPLPLL | 8 | Beta Casein | 2 | 458.30 | 914.60 | 49.87 | 148-155 |  | + | 1 |
| **284** | LHLPLPLLQS | 10 | Beta Casein | 2 | 565.85 | 1129.69 | 48.73 | 148-157 |  | + | 1 |
| **285** | LSQSKVLPVPQ | 11 | Beta Casein | 2 | 598.36 | 1194.70 | 37.73 | 180-190 |  | + | 1 |
| **286** | LSQSKVLPVPQKAVPYPQ | 18 | Beta Casein | 3 | 660.38 | 1978.13 | 40.33 | 180-197 |  | + | 1 |
| **287** | LTQTPVVVPPFLQPEVM | 17 | Beta Casein | 2 | 948.02 | 1894.03 | 53.38 | 92-108 |  | + | 1 |
| **288** | LVYPFPGPIPN | 11 | Beta Casein | 2 | 607.33 | 1212.65 | 48.95 | 73-83 |  | + | 1 |
| **289** | MAPKHKEMPFPKYPVEPFTE | 20 | Beta Casein | 3 | 801.73 | 2402.18 | 41.16 | 117-136 |  | + | 1 |
| **290** | MAPKHKEMPFPKYPVEPFTES | 21 | Beta Casein | 3 | 830.74 | 2489.21 | 41.10 | 117-137 |  | + | 1 |
| **291** | MHQPHQPLPPTVMFPPQSVL | 20 | Beta Casein | 3 | 761.06 | 2280.15 | 45.19 | 159-178 |  | + | 1 |
| **292** | NIPPLTQTPVVVPPFLQPEVMG | 22 | Beta Casein | 2 | 1187.15 | 2372.28 | 54.74 | 88-109 |  | + | 1 |
| **293** | NLHLPLPLLQS | 11 | Beta Casein | 2 | 622.87 | 1243.73 | 49.00 | 147-157 |  | + | 1 |
| **294** | NLHLPLPLLQSW | 12 | Beta Casein | 2 | 715.91 | 1429.81 | 52.17 | 147-158 |  | + | 1 |
| **295** | PFPKYPVEPF | 10 | Beta Casein | 2 | 610.82 | 1219.63 | 44.76 | 125-134 |  | + | 1 |
| **296** | PVLGPVRGPFPIIV | 14 | Beta Casein | 2 | 730.95 | 1459.89 | 49.39 | 211-224 |  | + | 1 |
| **297** | QDKIHPF | 7 | Beta Casein | 2 | 442.73 | 883.46 | 27.62 | 61-67 |  | + | 1 |
| **298** | QEPVLGPVRGPFPI | 14 | Beta Casein | 2 | 753.43 | 1504.84 | 46.72 | 209-222 |  | + | 1 |
| **299** | QPH | 3 | Beta Casein | 1 | 381.19 | 380.18 | 52.92 | 161-163 |  | + | 1 |
| **300** | QQQTEDELQDKIHPF | 15 | Beta Casein | 3 | 619.30 | 1854.87 | 41.33 | 53-67 |  | + | 1 |
| **301** | QSEEQQQTED | 10 | Beta Casein | 2 | 611.25 | 1220.48 | 16.05 | 49-58 |  | + | 1 |
| **302** | QSEEQQQTEDELQ | 13 | Beta Casein | 2 | 796.34 | 1590.66 | 29.57 | 49-61 |  | + | 1 |
| **303** | QSLVYPFPGPIPNSLPQ | 17 | Beta Casein | 2 | 927.49 | 1852.97 | 50.28 | 71-87 |  | + | 1 |
| **304** | QSWMHQPHQPLPPT | 14 | Beta Casein | 3 | 561.94 | 1682.80 | 35.35 | 156-169 |  | + | 1 |
| **305** | QSWMHQPHQPLPPTVMFPPQSVLSLS | 26 | Beta Casein | 3 | 990.50 | 2968.47 | 46.96 | 156-181 |  | + | 1 |
| **306** | RDMPIQ | 6 | Beta Casein | 2 | 380.19 | 758.37 | 25.70 | 198-203 |  | + | 1 |
| **307** | RDMPIQA | 7 | Beta Casein | 2 | 415.71 | 829.41 | 29.52 | 198-204 |  | + | 1 |
| **308** | RELEEL | 6 | Beta Casein | 2 | 394.71 | 787.41 | 31.79 | 16-21 |  | + | 1 |
| **309** | RINKKIE | 7 | Beta Casein | 2 | 450.78 | 899.56 | 13.61 | 40-46 |  | + | 1 |
| **310** | SITRIN | 6 | Beta Casein | 2 | 352.21 | 702.40 | 22.09 | 37-42 |  | + | 1 |
| **311** | SITRINK | 7 | Beta Casein | 2 | 416.26 | 830.50 | 16.29 | 37-43 |  | + | 1 |
| **312** | SITRINKKIE | 10 | Beta Casein | 3 | 401.25 | 1200.72 | 20.64 | 37-46 |  | + | 1 |
| **313** | SITRINKKIEKF | 12 | Beta Casein | 3 | 492.97 | 1475.88 | 25.78 | 37-48 |  | + | 1 |
| **314** | SKVLPVPQ | 8 | Beta Casein | 2 | 434.27 | 866.52 | 34.18 | 183-190 |  | + | 1 |
| **315** | SKVLPVPQKAVPYPQ | 15 | Beta Casein | 3 | 550.99 | 1649.95 | 37.92 | 183-197 |  | + | 1 |
| **316** | SLPQNIPPLTQTPVVVPPFLQPE | 23 | Beta Casein | 3 | 837.80 | 2510.38 | 53.44 | 84-106 |  | + | 1 |
| **317** | SLSQSKVLPVPQKAVPYPQRDMPIQ | 25 | Beta Casein | 3 | 936.18 | 2805.52 | 41.56 | 179-203 |  | + | 1 |
| **318** | SLVYPFPGPIPNSLPQ | 16 | Beta Casein | 2 | 863.46 | 1724.91 | 50.63 | 72-87 |  | + | 1 |
| **319** | SWMHQPHQPLPPTVM | 15 | Beta Casein | 3 | 595.96 | 1784.85 | 41.51 | 157-171 |  | + | 1 |
| **320** | SWMHQPHQPLPPTVMFPPQSV | 21 | Beta Casein | 3 | 814.40 | 2440.18 | 44.52 | 157-177 |  | + | 1 |
| **321** | SWMHQPHQPLPPTVMFPPQSVLSL | 24 | Beta Casein | 3 | 918.80 | 2753.38 | 48.15 | 157-180 |  | + | 1 |
| **322** | SWMHQPHQPLPPTVMFPPQSVLSLS | 25 | Beta Casein | 3 | 947.81 | 2840.41 | 47.45 | 157-181 |  | + | 1 |
| **323** | TEDELQDK | 8 | Beta Casein | 2 | 489.22 | 976.43 | 19.53 | 56-63 |  | + | 1 |
| **324** | TESQSLTLT | 9 | Beta Casein | 2 | 490.25 | 978.49 | 38.86 | 135-143 |  | + | 1 |
| **325** | TESQSLTLTDVE | 12 | Beta Casein | 2 | 661.82 | 1321.62 | 43.37 | 135-146 |  | + | 1 |
| **326** | TESQSLTLTDVEN | 13 | Beta Casein | 2 | 718.84 | 1435.67 | 42.64 | 135-147 |  | + | 1 |
| **327** | TQTPVVVPPFLQPEVMGV | 18 | Beta Casein | 2 | 969.52 | 1937.03 | 54.11 | 93-110 |  | + | 1 |
| **328** | TQTPVVVPPFLQPEVMGVSKV | 21 | Beta Casein | 3 | 751.42 | 2251.23 | 49.95 | 93-113 |  | + | 1 |
| **329** | TQTPVVVPPFLQPEVMGVSKVK | 22 | Beta Casein | 3 | 794.12 | 2379.32 | 46.88 | 93-114 |  | + | 1 |
| **330** | TQTPVVVPPFLQPEVMGVSKVKEA | 24 | Beta Casein | 3 | 860.81 | 2579.40 | 46.89 | 93-116 |  | + | 1 |
| **331** | TVMFPPQSVL | 10 | Beta Casein | 1 | 1118.59 | 1117.58 | 48.59 | 169-178 |  | + | 1 |
| **332** | VKEAMAPK | 8 | Beta Casein | 2 | 437.25 | 872.48 | 15.84 | 113-120 |  | + | 1 |
| **333** | VMFPPQSVLSL | 11 | Beta Casein | 2 | 609.33 | 1216.65 | 51.62 | 170-180 |  | + | 1 |
| **334** | VMFPPQSVLSLS | 12 | Beta Casein | 2 | 652.85 | 1303.68 | 50.26 | 170-181 |  | + | 1 |
| **335** | VMFPPQSVLSLSQS | 14 | Beta Casein | 2 | 760.39 | 1518.78 | 49.27 | 170-183 |  | + | 1 |
| **336** | VMGVSKVK | 8 | Beta Casein | 2 | 424.26 | 846.50 | 20.20 | 107-114 |  | + | 1 |
| **337** | VMGVSKVKEA | 10 | Beta Casein | 2 | 524.30 | 1046.58 | 22.66 | 107-116 |  | + | 1 |
| **338** | VMGVSKVKEAMAPK | 14 | Beta Casein | 3 | 492.28 | 1473.80 | 29.19 | 107-120 |  | + | 1 |
| **339** | VVPPFLQPE | 9 | Beta Casein | 2 | 513.29 | 1024.56 | 47.35 | 98-106 |  | + | 1 |
| **340** | VVPPFLQPEVMGVS | 14 | Beta Casein | 2 | 749.90 | 1497.79 | 50.85 | 98-111 |  | + | 1 |
| **341** | WMHQPHQPLPPT | 12 | Beta Casein | 3 | 490.24 | 1467.71 | 32.49 | 158-169 |  | + | 1 |
| **342** | WMHQPHQPLPPTVMFPPQ | 18 | Beta Casein | 3 | 723.36 | 2167.05 | 43.55 | 158-175 |  | + | 1 |
| **343** | WMHQPHQPLPPTVMFPPQSVL | 21 | Beta Casein | 3 | 823.09 | 2466.23 | 45.82 | 158-178 |  | + | 1 |
| **344** | WMHQPHQPLPPTVMFPPQSVLSLS | 24 | Beta Casein | 3 | 918.80 | 2753.38 | 47.18 | 158-181 |  | + | 1 |
| **345** | YQEPVLGPV | 9 | Beta Casein | 2 | 501.27 | 1000.52 | 44.80 | 208-216 |  | + | 1 |
| **346** | GEC | 3 | Beta Lactoglobulin | 1 | 365.11 | 364.11 | 49.17 | 153-155 |  | + | 1 |
| **347** | LIVTQTMKGL | 10 | Beta Lactoglobulin | 2 | 552.33 | 1102.64 | 42.83 | 17-26 |  | + | 1 |
| **348** | YKK | 3 | Beta Lactoglobulin | 1 | 438.27 | 437.26 | 59.64 | 115-117 |  | + | 1 |
| **349** | YLL | 3 | Beta Lactoglobulin | 1 | 408.25 | 407.24 | 41.96 | 118-120 |  | + | 1 |
| **350** | AIPPKKNQ | 8 | Kappa Casein | 2 | 448.27 | 894.53 | 15.96 | 128-135 | + | + | 2 |
| **351** | AIPPKKNQD | 9 | Kappa Casein | 2 | 505.79 | 1009.56 | 13.19 | 128-136 | + | + | 2 |
| **352** | ALINNQFLPYPY | 12 | Kappa Casein | 2 | 726.88 | 1451.74 | 50.07 | 70-81 | + | + | 2 |
| **353** | ALINNQFLPYPYYAKPA | 17 | Kappa Casein | 2 | 992.02 | 1982.03 | 46.11 | 70-86 | + | + | 2 |
| **354** | AVRSPAQIL | 9 | Kappa Casein | 2 | 477.79 | 953.57 | 39.42 | 87-95 | + | + | 2 |
| **355** | DKIAKYIPIQY | 11 | Kappa Casein | 2 | 676.38 | 1350.75 | 42.70 | 41-51 | + | + | 2 |
| **356** | DKTEIPTIN | 9 | Kappa Casein | 2 | 515.77 | 1029.53 | 37.66 | 136-144 | + | + | 2 |
| **357** | FFL | 3 | Kappa Casein | 1 | 426.24 | 425.23 | 8.21 | 5-7 | + |  | 1 |
| **358** | FMAIPPKKN | 9 | Kappa Casein | 2 | 523.30 | 1044.58 | 28.62 | 126-134 | + |  | 1 |
| **359** | IAKYIPIQY | 9 | Kappa Casein | 2 | 554.82 | 1107.63 | 42.58 | 43-51 | + | + | 2 |
| **360** | IESPPEINTVQVT | 13 | Kappa Casein | 2 | 713.87 | 1425.74 | 43.32 | 174-186 | + |  | 1 |
| **361** | INNQFLPYPYYAKPA | 15 | Kappa Casein | 2 | 899.96 | 1797.91 | 44.35 | 72-86 | + | + | 2 |
| **362** | KDERFFSD | 8 | Kappa Casein | 2 | 522.24 | 1042.47 | 29.87 | 34-41 | + | + | 2 |
| **363** | KIAKYIPIQY | 10 | Kappa Casein | 2 | 618.87 | 1235.73 | 40.22 | 42-51 | + | + | 2 |
| **364** | KTEIPTIN | 8 | Kappa Casein | 2 | 458.26 | 914.51 | 34.45 | 137-144 | + | + | 2 |
| **365** | KYIPIQY | 7 | Kappa Casein | 2 | 462.76 | 923.51 | 39.43 | 45-51 | + | + | 2 |
| **366** | KYIPIQYVLS | 10 | Kappa Casein | 2 | 612.36 | 1222.70 | 44.54 | 45-54 | + | + | 2 |
| **367** | LINNQFLPYPYYAKPA | 16 | Kappa Casein | 2 | 956.50 | 1910.99 | 45.24 | 71-86 | + |  | 1 |
| **368** | NYYQQKPVAL | 10 | Kappa Casein | 2 | 612.32 | 1222.63 | 36.51 | 62-71 | + | + | 2 |
| **369** | NYYQQKPVALINN | 13 | Kappa Casein | 2 | 782.91 | 1563.80 | 39.07 | 62-74 | + | + | 2 |
| **370** | QDKTEIPTIN | 10 | Kappa Casein | 2 | 579.80 | 1157.59 | 36.02 | 135-144 | + | + | 2 |
| **371** | QFLPYPYYAKPA | 12 | Kappa Casein | 2 | 729.38 | 1456.74 | 43.88 | 75-86 | + | + | 2 |
| **372** | QWQVLSNTVPA | 11 | Kappa Casein | 2 | 621.83 | 1241.64 | 45.36 | 96-106 | + | + | 2 |
| **373** | QWQVLSNTVPAKS | 13 | Kappa Casein | 2 | 729.39 | 1456.77 | 41.79 | 96-108 | + | + | 2 |
| **374** | QYVLSRYPSYGLN | 13 | Kappa Casein | 2 | 780.40 | 1558.78 | 42.40 | 50-62 | + | + | 2 |
| **375** | SPPEINTVQVTSTAV | 15 | Kappa Casein | 2 | 771.90 | 1541.79 | 43.53 | 176-190 | + | + | 2 |
| **376** | SRYPSYGLN | 9 | Kappa Casein | 2 | 528.76 | 1055.50 | 32.87 | 54-62 | + | + | 2 |
| **377** | TIASGEPTSTPTTE | 14 | Kappa Casein | 2 | 696.33 | 1390.65 | 30.97 | 145-158 | + | + | 2 |
| **378** | TIASGEPTSTPTTEAVE | 17 | Kappa Casein | 2 | 845.90 | 1689.79 | 36.63 | 145-161 | + | + | 2 |
| **379** | VIESPPEINTVQVT | 14 | Kappa Casein | 2 | 763.41 | 1524.80 | 43.83 | 173-186 | + | + | 2 |
| **380** | VIESPPEINTVQVTSTAV | 18 | Kappa Casein | 2 | 942.50 | 1882.99 | 45.02 | 173-190 | + | + | 2 |
| **381** | VLSRYPSYGLN | 11 | Kappa Casein | 2 | 634.84 | 1267.66 | 38.25 | 52-62 | + | + | 2 |
| **382** | VRS | 3 | Kappa Casein | 1 | 361.22 | 360.21 | 59.49 | 88-90 | + | + | 2 |
| **383** | YQQKPVALINN | 11 | Kappa Casein | 2 | 644.36 | 1286.70 | 34.39 | 64-74 | + | + | 2 |
| **384** | YYQQKPVA | 8 | Kappa Casein | 2 | 498.76 | 995.51 | 22.49 | 63-70 | + | + | 2 |
| **385** | YYQQKPVAL | 9 | Kappa Casein | 2 | 555.30 | 1108.59 | 34.31 | 63-71 | + | + | 2 |
| **386** | YYQQKPVALIN | 11 | Kappa Casein | 2 | 668.87 | 1335.72 | 38.36 | 63-73 | + | + | 2 |
| **387** | YYQQKPVALINN | 12 | Kappa Casein | 2 | 725.89 | 1449.76 | 37.24 | 63-74 | + | + | 2 |
| **388** | DERFFSDK | 8 | Kappa Casein | 2 | 522.24 | 1042.47 | 30.52 | 35-42 |  | + | 1 |
| **389** | DERFFSDKIA | 10 | Kappa Casein | 2 | 614.30 | 1226.59 | 40.62 | 35-44 |  | + | 1 |
| **390** | DKIAKYIPI | 9 | Kappa Casein | 2 | 530.82 | 1059.63 | 42.47 | 41-49 |  | + | 1 |
| **391** | DKIAKYIPIQ | 10 | Kappa Casein | 2 | 594.85 | 1187.69 | 40.82 | 41-50 |  | + | 1 |
| **392** | DKIAKYIPIQYVL | 13 | Kappa Casein | 2 | 782.46 | 1562.91 | 46.33 | 41-53 |  | + | 1 |
| **393** | DKTEIPTINTIA | 12 | Kappa Casein | 2 | 658.36 | 1314.70 | 43.72 | 136-147 |  | + | 1 |
| **394** | EKDERFF | 7 | Kappa Casein | 2 | 485.74 | 969.46 | 32.28 | 33-39 |  | + | 1 |
| **395** | EKDERFFS | 8 | Kappa Casein | 2 | 529.25 | 1056.49 | 29.35 | 33-40 |  | + | 1 |
| **396** | EKDERFFSD | 9 | Kappa Casein | 2 | 586.76 | 1171.51 | 30.54 | 33-41 |  | + | 1 |
| **397** | FLPYPYYAKPA | 11 | Kappa Casein | 2 | 665.35 | 1328.68 | 43.26 | 76-86 |  | + | 1 |
| **398** | FSDKIAKYIPI | 11 | Kappa Casein | 2 | 647.87 | 1293.73 | 43.56 | 39-49 |  | + | 1 |
| **399** | IAKYIPIQYVLS | 12 | Kappa Casein | 2 | 704.42 | 1406.82 | 46.13 | 43-54 |  | + | 1 |
| **400** | IESPPEIN | 8 | Kappa Casein | 1 | 898.45 | 897.44 | 36.91 | 174-181 |  | + | 1 |
| **401** | INNQFLPYPY | 10 | Kappa Casein | 2 | 634.82 | 1267.62 | 47.72 | 72-81 |  | + | 1 |
| **402** | IPIQY | 5 | Kappa Casein | 1 | 633.36 | 632.35 | 41.58 | 47-51 |  | + | 1 |
| **403** | KDERFFS | 7 | Kappa Casein | 2 | 464.73 | 927.45 | 28.46 | 34-40 |  | + | 1 |
| **404** | KDERFFSDK | 9 | Kappa Casein | 2 | 586.29 | 1170.57 | 23.12 | 34-42 |  | + | 1 |
| **405** | KIAKYIPI | 8 | Kappa Casein | 2 | 473.31 | 944.61 | 38.05 | 42-49 |  | + | 1 |
| **406** | KIAKYIPIQ | 9 | Kappa Casein | 2 | 537.34 | 1072.66 | 34.65 | 42-50 |  | + | 1 |
| **407** | KYIPIQ | 6 | Kappa Casein | 2 | 381.23 | 760.45 | 31.86 | 45-50 |  | + | 1 |
| **408** | KYIPIQYV | 8 | Kappa Casein | 1 | 1023.59 | 1022.58 | 42.29 | 45-52 |  | + | 1 |
| **409** | KYIPIQYVL | 9 | Kappa Casein | 2 | 568.84 | 1135.66 | 45.36 | 45-53 |  | + | 1 |
| **410** | LSRYPSYGLN | 10 | Kappa Casein | 2 | 585.30 | 1168.59 | 35.76 | 53-62 |  | + | 1 |
| **411** | MAIPPKKN | 8 | Kappa Casein | 2 | 449.76 | 897.51 | 21.59 | 127-134 |  | + | 1 |
| **412** | NNQFLPYPYYAKPA | 14 | Kappa Casein | 2 | 843.42 | 1684.82 | 43.84 | 73-86 |  | + | 1 |
| **413** | NQDKTEIPTIN | 11 | Kappa Casein | 2 | 636.83 | 1271.64 | 35.93 | 134-144 |  | + | 1 |
| **414** | QFL | 3 | Kappa Casein | 1 | 407.23 | 406.22 | 19.56 | 75-77 |  | + | 1 |
| **415** | QFLPYPY | 7 | Kappa Casein | 1 | 927.46 | 926.45 | 47.40 | 75-81 |  | + | 1 |
| **416** | QQKPVALINN | 10 | Kappa Casein | 2 | 562.82 | 1123.63 | 31.98 | 65-74 |  | + | 1 |
| **417** | QWQVLSN | 7 | Kappa Casein | 1 | 874.44 | 873.43 | 43.21 | 96-102 |  | + | 1 |
| **418** | RYPSYGLN | 8 | Kappa Casein | 2 | 485.24 | 968.47 | 32.68 | 55-62 |  | + | 1 |
| **419** | SDKIAKYIPIQY | 12 | Kappa Casein | 2 | 719.90 | 1437.79 | 42.29 | 40-51 |  | + | 1 |
| **420** | SPPEIN | 6 | Kappa Casein | 1 | 656.32 | 655.32 | 27.11 | 176-181 |  | + | 1 |
| **421** | SPPEINTVQVT | 11 | Kappa Casein | 2 | 592.81 | 1183.61 | 41.47 | 176-186 |  | + | 1 |
| **422** | TVQVTSTAV | 9 | Kappa Casein | 2 | 453.25 | 904.49 | 35.18 | 182-190 |  | + | 1 |
| **423** | VIESPPEIN | 9 | Kappa Casein | 2 | 499.26 | 996.51 | 39.50 | 173-181 |  | + | 1 |
| **424** | VIESPPEINTVQVTST | 16 | Kappa Casein | 2 | 857.45 | 1712.88 | 43.62 | 173-188 |  | + | 1 |
| **425** | VLSRYPSYGL | 10 | Kappa Casein | 2 | 577.81 | 1153.61 | 41.26 | 52-61 |  | + | 1 |
| **426** | VTSTAV | 6 | Kappa Casein | 1 | 577.32 | 576.31 | 23.23 | 185-190 |  | + | 1 |
| **427** | YAKPAAVRSPA | 11 | Kappa Casein | 2 | 565.82 | 1129.62 | 22.38 | 82-92 |  | + | 1 |
| **428** | YAKPAAVRSPAQIL | 14 | Kappa Casein | 2 | 742.93 | 1483.85 | 37.86 | 82-95 |  | + | 1 |
| **429** | YQQKPVAL | 8 | Kappa Casein | 2 | 473.77 | 945.53 | 30.52 | 64-71 |  | + | 1 |
| **430** | YQQKPVALIN | 10 | Kappa Casein | 2 | 587.33 | 1172.66 | 35.77 | 64-73 |  | + | 1 |
| **431** | YVL | 3 | Kappa Casein | 1 | 394.23 | 393.23 | 18.43 | 51-53 |  | + | 1 |
| **432** | YYQQKPV | 7 | Kappa Casein | 2 | 463.24 | 924.47 | 22.04 | 63-69 |  | + | 1 |
| **433** | YYQQKPVALI | 10 | Kappa Casein | 2 | 611.85 | 1221.68 | 41.95 | 63-72 |  | + | 1 |
| **434** | YYQQKPVALINNQ | 13 | Kappa Casein | 2 | 789.92 | 1577.82 | 37.25 | 63-75 |  | + | 1 |
| **435** | YYQQKPVALINNQFLPYPY | 19 | Kappa Casein | 2 | 1180.11 | 2358.20 | 47.46 | 63-81 |  | + | 1 |
| **436** | FKDLGEEHFK | 10 | Serum Albumin | 2 | 625.31 | 1248.61 | 27.94 | 35-44 | + | + | 2 |
| **437** | GLVLIAFSQYLQQCPFDEHVK | 21 | Serum Albumin | 3 | 831.43 | 2491.26 | 49.55 | 45-65 | + | + | 2 |
| **438** | KFG | 3 | Serum Albumin | 1 | 351.20 | 350.20 | 58.80 | 228-230 | + |  | 1 |
| **439** | QNC | 3 | Serum Albumin | 1 | 421.15 | 420.14 | 62.12 | 413-415 | + | + | 2 |
| **440** | RRD | 3 | Serum Albumin | 1 | 446.25 | 445.24 | 53.96 | 23-25 | + | + | 2 |
| **441** | RRH | 3 | Serum Albumin | 1 | 468.28 | 467.27 | 55.67 | 167-169 | + | + | 2 |
| **442** | SRR | 3 | Serum Albumin | 1 | 418.25 | 417.24 | 60.56 | 358-360 | + |  | 1 |
| **443** | VSR | 3 | Serum Albumin | 1 | 361.22 | 360.21 | 61.58 | 449-451 | + | + | 2 |
| **444** | YLQ | 3 | Serum Albumin | 1 | 423.22 | 422.22 | 30.83 | 110-112 | + |  | 1 |
| **445** | YVP | 3 | Serum Albumin | 1 | 378.20 | 377.20 | 29.32 | 181-183 | + |  | 1 |
| **446** | ARR | 3 | Serum Albumin | 1 | 402.26 | 401.25 | 58.80 | 166-168 |  | + | 1 |
| **447** | DAFLGSFLYEYSR | 13 | Serum Albumin | 2 | 784.38 | 1566.74 | 49.86 | 347-359 |  | + | 1 |
| **448** | HLVDEPQNLIK | 11 | Serum Albumin | 2 | 653.36 | 1304.71 | 36.86 | 402-412 |  | + | 1 |
| **449** | HRF | 3 | Serum Albumin | 1 | 459.25 | 458.24 | 50.33 | 33-35 |  | + | 1 |
| **450** | KHL | 3 | Serum Albumin | 1 | 397.26 | 396.25 | 15.29 | 401-403 |  | + | 1 |
| **451** | KQTALVELLK | 10 | Serum Albumin | 2 | 571.86 | 1141.71 | 41.50 | 548-557 |  | + | 1 |
| **452** | KVPQVSTPTLVEVSR | 15 | Serum Albumin | 3 | 547.32 | 1638.93 | 39.74 | 437-451 |  | + | 1 |
| **453** | LGEYGFQNALIVR | 13 | Serum Albumin | 2 | 740.40 | 1478.79 | 44.84 | 421-433 |  | + | 1 |
| **454** | LKH | 3 | Serum Albumin | 1 | 397.26 | 396.25 | 45.72 | 400-402 |  | + | 1 |
| **455** | PFD | 3 | Serum Albumin | 1 | 378.17 | 377.16 | 46.19 | 59-61 |  | + | 1 |
| **456** | RDT | 3 | Serum Albumin | 1 | 391.19 | 390.19 | 50.04 | 24-26 |  | + | 1 |
| **457** | RHPEYAVSVLLR | 12 | Serum Albumin | 3 | 480.61 | 1438.80 | 38.53 | 360-371 |  | + | 1 |
| **458** | RHPYFYAPELLYYANK | 16 | Serum Albumin | 3 | 682.35 | 2044.02 | 43.61 | 168-183 |  | + | 1 |
| **459** | RLC | 3 | Serum Albumin | 1 | 448.23 | 447.23 | 51.83 | 482-484 |  | + | 1 |
| **460** | SLHTLFGDELCK | 12 | Serum Albumin | 2 | 710.35 | 1418.69 | 42.30 | 89-100 |  | + | 1 |
| **461** | TRC | 3 | Serum Albumin | 1 | 436.20 | 435.19 | 47.73 | 458-460 |  | + | 1 |
| **462** | TRK | 3 | Serum Albumin | 1 | 404.26 | 403.25 | 52.05 | 435-437 |  | + | 1 |
| **463** | TVMENFVAFVDK | 12 | Serum Albumin | 2 | 700.35 | 1398.69 | 47.18 | 569-580 |  | + | 1 |
| **464** | YSR | 3 | Serum Albumin | 1 | 425.21 | 424.21 | 63.69 | 17-19 |  | + | 1 |
| **465** | YST | 3 | Serum Albumin | 1 | 370.16 | 369.15 | 61.43 | 393-395 |  | + | 1 |
| **466** | YTR | 3 | Serum Albumin | 1 | 439.23 | 438.22 | 57.67 | 434-436 |  | + | 1 |

**Supplementary Table 4.** Milk peptides identified throughout the 28 days of storage period using the *L. plantarum* NCCB 100524.

|  | **Sequence** | **Length** | **Leading proteins** | **Charge** | **m/z** | **Mass** | **Retention time** | **Sequence** | **Day 1** | **Day 28** | **Incidence** |
| --- | --- | --- | --- | --- | --- | --- | --- | --- | --- | --- | --- |
| **1** | APFPEVFGKE | 10 | Alpha S1 casein | 2 | 560.79 | 1119.56 | 44.01 | 41-50 | + | + | 2 |
| **2** | APFPEVFGKEK | 11 | Alpha S1 casein | 2 | 624.83 | 1247.66 | 41.08 | 41-51 | + | + | 2 |
| **3** | DIPNPIGSENSEKTTMPLW | 19 | Alpha S1 casein | 2 | 1065.01 | 2128.01 | 47.99 | 196-214 | + | + | 2 |
| **4** | FSDIPNPIGSENSEK | 15 | Alpha S1 casein | 2 | 817.39 | 1632.76 | 41.90 | 194-208 | + | + | 2 |
| **5** | FSDIPNPIGSENSEKT | 16 | Alpha S1 casein | 2 | 867.91 | 1733.81 | 41.98 | 194-209 | + | + | 2 |
| **6** | FSDIPNPIGSENSEKTT | 17 | Alpha S1 casein | 2 | 918.44 | 1834.86 | 42.02 | 194-210 | + | + | 2 |
| **7** | FSDIPNPIGSENSEKTTMP | 19 | Alpha S1 casein | 2 | 1032.48 | 2062.95 | 43.39 | 194-212 | + | + | 2 |
| **8** | FSDIPNPIGSENSEKTTMPLW | 21 | Alpha S1 casein | 2 | 1190.06 | 2378.11 | 46.04 | 194-214 | + | + | 2 |
| **9** | FVAPFPEVFGK | 11 | Alpha S1 casein | 2 | 619.33 | 1236.65 | 47.39 | 39-49 | + | + | 2 |
| **10** | GLPQEVLNENLLR | 13 | Alpha S1 casein | 2 | 747.92 | 1493.82 | 45.30 | 25-37 | + | + | 2 |
| **11** | IPNPIGSENSEKTTMPLW | 18 | Alpha S1 casein | 2 | 1015.50 | 2028.98 | 44.41 | 197-214 | + | + | 2 |
| **12** | KEDVPSERYLG | 11 | Alpha S1 casein | 2 | 646.83 | 1291.64 | 30.22 | 98-108 | + | + | 2 |
| **13** | KVPQLEIVPN | 10 | Alpha S1 casein | 2 | 568.84 | 1135.66 | 42.66 | 120-129 | + |  | 1 |
| **14** | LEQ | 3 | Alpha S1 casein | 1 | 389.20 | 388.20 | 58.76 | 276-278 | + |  | 1 |
| **15** | LKK | 3 | Alpha S1 casein | 1 | 388.29 | 387.28 | 53.00 | 679-681 | + |  | 1 |
| **16** | NENLLRF | 7 | Alpha S1 casein | 2 | 453.25 | 904.48 | 43.53 | 32-38 | + | + | 2 |
| **17** | NSEKTTMPLW | 10 | Alpha S1 casein | 2 | 603.79 | 1205.58 | 43.68 | 205-214 | + |  | 1 |
| **18** | PIGSENSEKTTMPLW | 15 | Alpha S1 casein | 2 | 853.41 | 1704.80 | 42.43 | 200-214 | + | + | 2 |
| **19** | QKEPMIGVNQEL | 12 | Alpha S1 casein | 2 | 693.36 | 1384.70 | 41.67 | 146-157 | + | + | 2 |
| **20** | QKEPMIGVNQELAY | 14 | Alpha S1 casein | 2 | 810.41 | 1618.80 | 42.80 | 146-159 | + |  | 1 |
| **21** | RLH | 3 | Alpha S1 casein | 1 | 425.26 | 424.25 | 16.20 | 1814-1816 | + |  | 1 |
| **22** | RPK | 3 | Alpha S1 casein | 1 | 400.27 | 399.26 | 31.33 | 674-676 | + |  | 1 |
| **23** | SDIPNPIGSENSEKTTMPLW | 20 | Alpha S1 casein | 2 | 1108.53 | 2215.05 | 46.67 | 195-214 | + | + | 2 |
| **24** | SEKTTMPLW | 9 | Alpha S1 casein | 2 | 546.77 | 1091.53 | 43.80 | 206-214 | + | + | 2 |
| **25** | TED | 3 | Alpha S1 casein | 1 | 364.14 | 363.13 | 49.01 | 199-201 | + |  | 1 |
| **26** | VAPFPEVFGK | 10 | Alpha S1 casein | 2 | 545.80 | 1089.59 | 44.89 | 40-49 | + | + | 2 |
| **27** | VAPFPEVFGKE | 11 | Alpha S1 casein | 2 | 610.32 | 1218.63 | 44.96 | 40-50 | + | + | 2 |
| **28** | VAPFPEVFGKEK | 12 | Alpha S1 casein | 2 | 674.37 | 1346.72 | 42.38 | 40-51 | + | + | 2 |
| **29** | VAPFPEVFGKEKV | 13 | Alpha S1 casein | 2 | 723.90 | 1445.79 | 43.22 | 40-52 | + | + | 2 |
| **30** | YKVPQLEIVPN | 11 | Alpha S1 casein | 2 | 650.37 | 1298.72 | 44.01 | 119-129 | + |  | 1 |
| **31** | APFPEVFGK | 9 | Alpha S1 Casein | 2 | 496.27 | 990.52 | 43.96 | 41-49 |  |  | 1 |
| **32** | DVPSERY | 7 | Alpha S1 Casein | 2 | 433.21 | 864.40 | 26.46 | 100-106 |  | + | 1 |
| **33** | DVPSERYLG | 9 | Alpha S1 Casein | 2 | 518.26 | 1034.50 | 36.14 | 100-108 |  | + | 1 |
| **34** | DVPSERYLGY | 10 | Alpha S1 Casein | 2 | 599.79 | 1197.57 | 41.99 | 100-109 |  | + | 1 |
| **35** | EDIKQMEAE | 9 | Alpha S1 Casein | 2 | 546.75 | 1091.48 | 28.98 | 70-78 |  | + | 1 |
| **36** | EDVPSER | 7 | Alpha S1 Casein | 2 | 416.20 | 830.38 | 19.54 | 99-105 |  | + | 1 |
| **37** | EDVPSERYLG | 10 | Alpha S1 Casein | 2 | 582.78 | 1163.55 | 35.83 | 99-108 |  | + | 1 |
| **38** | EDVPSERYLGY | 11 | Alpha S1 Casein | 2 | 664.31 | 1326.61 | 41.51 | 99-109 |  | + | 1 |
| **39** | EGIHAQQ | 7 | Alpha S1 Casein | 2 | 391.69 | 781.37 | 17.03 | 140-146 |  | + | 1 |
| **40** | EIVPNSAEER | 10 | Alpha S1 Casein | 2 | 572.29 | 1142.56 | 27.40 | 125-134 |  | + | 1 |
| **41** | ENLLRF | 6 | Alpha S1 Casein | 2 | 396.22 | 790.43 | 43.73 | 33-38 |  | + | 1 |
| **42** | EQLLRL | 6 | Alpha S1 Casein | 2 | 386.24 | 770.47 | 42.97 | 111-116 |  | + | 1 |
| **43** | ERYLGYLEQL | 10 | Alpha S1 Casein | 2 | 642.34 | 1282.66 | 45.26 | 104-113 |  | + | 1 |
| **44** | EVFGKEKVNE | 10 | Alpha S1 Casein | 2 | 589.81 | 1177.60 | 24.71 | 45-54 |  | + | 1 |
| **45** | EVFGKEKVNEL | 11 | Alpha S1 Casein | 2 | 646.35 | 1290.68 | 34.64 | 45-55 |  | + | 1 |
| **46** | EVFGKEKVNELS | 12 | Alpha S1 Casein | 2 | 689.86 | 1377.71 | 32.62 | 45-56 |  | + | 1 |
| **47** | EVLNENLLR | 9 | Alpha S1 Casein | 2 | 550.31 | 1098.60 | 41.98 | 29-37 |  | + | 1 |
| **48** | EVLNENLLRF | 10 | Alpha S1 Casein | 2 | 623.84 | 1245.67 | 46.39 | 29-38 |  | + | 1 |
| **49** | FFV | 3 | Alpha S1 Casein | 1 | 412.22 | 411.22 | 45.52 | 38-40 |  | + | 1 |
| **50** | FFVAP | 5 | Alpha S1 Casein | 1 | 580.31 | 579.31 | 45.29 | 38-42 |  | + | 1 |
| **51** | FFVAPFPEVFGK | 12 | Alpha S1 Casein | 2 | 692.87 | 1383.72 | 49.92 | 38-49 |  | + | 1 |
| **52** | FPEVFGK | 7 | Alpha S1 Casein | 2 | 412.22 | 822.43 | 42.38 | 43-49 |  | + | 1 |
| **53** | FPEVFGKE | 8 | Alpha S1 Casein | 2 | 476.74 | 951.47 | 42.49 | 43-50 |  | + | 1 |
| **54** | FPEVFGKEK | 9 | Alpha S1 Casein | 2 | 540.79 | 1079.57 | 36.42 | 43-51 |  | + | 1 |
| **55** | FPEVFGKEKV | 10 | Alpha S1 Casein | 2 | 590.32 | 1178.63 | 40.64 | 43-52 |  | + | 1 |
| **56** | FPEVFGKEKVN | 11 | Alpha S1 Casein | 2 | 647.35 | 1292.68 | 38.57 | 43-53 |  | + | 1 |
| **57** | FPEVFGKEKVNE | 12 | Alpha S1 Casein | 2 | 711.87 | 1421.72 | 38.86 | 43-54 |  | + | 1 |
| **58** | FPEVFGKEKVNEL | 13 | Alpha S1 Casein | 2 | 768.41 | 1534.80 | 42.67 | 43-55 |  | + | 1 |
| **59** | FSDIPNPIGSENSE | 14 | Alpha S1 Casein | 2 | 753.34 | 1504.67 | 44.47 | 194-207 |  | + | 1 |
| **60** | FSDIPNPIGSENSEKTTMPL | 20 | Alpha S1 Casein | 2 | 1089.03 | 2176.04 | 45.05 | 194-213 |  | + | 1 |
| **61** | FVAPFPEVFGKE | 12 | Alpha S1 Casein | 2 | 683.86 | 1365.70 | 47.45 | 39-50 |  | + | 1 |
| **62** | FVAPFPEVFGKEK | 13 | Alpha S1 Casein | 2 | 747.90 | 1493.79 | 44.58 | 39-51 |  | + | 1 |
| **63** | FVAPFPEVFGKEKV | 14 | Alpha S1 Casein | 3 | 531.96 | 1592.86 | 45.10 | 39-52 |  | + | 1 |
| **64** | FVAPFPEVFGKEKVN | 15 | Alpha S1 Casein | 3 | 569.98 | 1706.90 | 44.72 | 39-53 |  | + | 1 |
| **65** | GLPQEVLNE | 9 | Alpha S1 Casein | 2 | 499.76 | 997.51 | 43.59 | 25-33 |  | + | 1 |
| **66** | GLPQEVLNENLL | 12 | Alpha S1 Casein | 2 | 669.87 | 1337.72 | 49.25 | 25-36 |  | + | 1 |
| **67** | HIQKEDVPSE | 10 | Alpha S1 Casein | 2 | 591.29 | 1180.57 | 20.50 | 95-104 |  | + | 1 |
| **68** | HQGLPQEVLN | 10 | Alpha S1 Casein | 2 | 567.80 | 1133.58 | 36.85 | 23-32 |  | + | 1 |
| **69** | HQGLPQEVLNE | 11 | Alpha S1 Casein | 2 | 632.32 | 1262.63 | 39.37 | 23-33 |  | + | 1 |
| **70** | HQGLPQEVLNEN | 12 | Alpha S1 Casein | 2 | 689.34 | 1376.67 | 38.29 | 23-34 |  | + | 1 |
| **71** | HQGLPQEVLNENL | 13 | Alpha S1 Casein | 2 | 745.88 | 1489.75 | 43.34 | 23-35 |  | + | 1 |
| **72** | HQGLPQEVLNENLL | 14 | Alpha S1 Casein | 2 | 802.43 | 1602.84 | 45.43 | 23-36 |  | + | 1 |
| **73** | HQGLPQEVLNENLLR | 15 | Alpha S1 Casein | 2 | 880.48 | 1758.94 | 43.28 | 23-37 |  | + | 1 |
| **74** | IPNPIGSENSE | 11 | Alpha S1 Casein | 2 | 578.78 | 1155.54 | 40.15 | 197-207 |  | + | 1 |
| **75** | IPNPIGSENSEK | 12 | Alpha S1 Casein | 2 | 642.83 | 1283.64 | 33.00 | 197-208 |  | + | 1 |
| **76** | IPNPIGSENSEKT | 13 | Alpha S1 Casein | 2 | 693.35 | 1384.68 | 33.63 | 197-209 |  | + | 1 |
| **77** | IPNPIGSENSEKTT | 14 | Alpha S1 Casein | 2 | 743.87 | 1485.73 | 33.83 | 197-210 |  | + | 1 |
| **78** | IPNPIGSENSEKTTMP | 16 | Alpha S1 Casein | 2 | 857.92 | 1713.82 | 41.04 | 197-212 |  | + | 1 |
| **79** | IPNPIGSENSEKTTMPL | 17 | Alpha S1 Casein | 2 | 922.46 | 1842.90 | 41.49 | 197-213 |  | + | 1 |
| **80** | IQKEDVPSE | 9 | Alpha S1 Casein | 2 | 522.76 | 1043.51 | 23.86 | 96-104 |  | + | 1 |
| **81** | KEDVPSE | 7 | Alpha S1 Casein | 2 | 402.19 | 802.37 | 19.28 | 98-104 |  | + | 1 |
| **82** | KEDVPSER | 8 | Alpha S1 Casein | 2 | 480.24 | 958.47 | 16.20 | 98-105 |  | + | 1 |
| **83** | KEDVPSERY | 9 | Alpha S1 Casein | 2 | 561.77 | 1121.54 | 22.56 | 98-106 |  | + | 1 |
| **84** | KEDVPSERYL | 10 | Alpha S1 Casein | 2 | 618.32 | 1234.62 | 31.95 | 98-107 |  | + | 1 |
| **85** | KEDVPSERYLGY | 12 | Alpha S1 Casein | 2 | 728.36 | 1454.70 | 36.14 | 98-109 |  | + | 1 |
| **86** | KEPMIGVNQEL | 11 | Alpha S1 Casein | 2 | 629.33 | 1256.64 | 41.66 | 147-157 |  | + | 1 |
| **87** | KTTMPLW | 7 | Alpha S1 Casein | 2 | 438.74 | 875.46 | 43.49 | 208-214 |  | + | 1 |
| **88** | KYKVPQL | 7 | Alpha S1 Casein | 2 | 438.27 | 874.53 | 32.51 | 118-124 |  | + | 1 |
| **89** | LNENLLR | 7 | Alpha S1 Casein | 2 | 436.25 | 870.49 | 35.32 | 31-37 |  | + | 1 |
| **90** | LNENLLRF | 8 | Alpha S1 Casein | 2 | 509.79 | 1017.56 | 44.80 | 31-38 |  | + | 1 |
| **91** | NPIGSENSEKTT | 12 | Alpha S1 Casein | 2 | 638.80 | 1275.59 | 20.54 | 199-210 |  | + | 1 |
| **92** | NPIGSENSEKTTMP | 14 | Alpha S1 Casein | 2 | 752.85 | 1503.69 | 31.59 | 199-212 |  | + | 1 |
| **93** | NPIGSENSEKTTMPL | 15 | Alpha S1 Casein | 2 | 809.39 | 1616.77 | 40.00 | 199-213 |  | + | 1 |
| **94** | NPIGSENSEKTTMPLW | 16 | Alpha S1 Casein | 2 | 902.43 | 1802.85 | 44.64 | 199-214 |  | + | 1 |
| **95** | PIGSENSEKTT | 11 | Alpha S1 Casein | 2 | 581.78 | 1161.55 | 17.90 | 200-210 |  | + | 1 |
| **96** | QEVLNENL | 8 | Alpha S1 Casein | 2 | 479.75 | 957.48 | 42.33 | 28-35 |  | + | 1 |
| **97** | QEVLNENLL | 9 | Alpha S1 Casein | 2 | 536.29 | 1070.56 | 45.94 | 28-36 |  | + | 1 |
| **98** | QEVLNENLLR | 10 | Alpha S1 Casein | 2 | 614.34 | 1226.66 | 42.14 | 28-37 |  | + | 1 |
| **99** | QEVLNENLLRF | 11 | Alpha S1 Casein | 2 | 687.87 | 1373.73 | 46.24 | 28-38 |  | + | 1 |
| **100** | QGLPQEVLNENLLR | 14 | Alpha S1 Casein | 2 | 811.95 | 1621.88 | 45.16 | 24-37 |  | + | 1 |
| **101** | QKEPMIGV | 8 | Alpha S1 Casein | 2 | 451.24 | 900.47 | 36.02 | 146-153 |  | + | 1 |
| **102** | QQKEPMIGVNQE | 12 | Alpha S1 Casein | 2 | 700.85 | 1399.68 | 32.50 | 145-156 |  | + | 1 |
| **103** | SDIPNPIGSENSE | 13 | Alpha S1 Casein | 2 | 679.81 | 1357.60 | 42.38 | 195-207 |  | + | 1 |
| **104** | SDIPNPIGSENSEK | 14 | Alpha S1 Casein | 2 | 743.85 | 1485.69 | 36.79 | 195-208 |  | + | 1 |
| **105** | SDIPNPIGSENSEKT | 15 | Alpha S1 Casein | 2 | 794.38 | 1586.74 | 37.26 | 195-209 |  | + | 1 |
| **106** | SDIPNPIGSENSEKTTMPL | 19 | Alpha S1 Casein | 2 | 1015.49 | 2028.97 | 43.96 | 195-213 |  | + | 1 |
| **107** | SMKEGIHA | 8 | Alpha S1 Casein | 2 | 436.72 | 871.42 | 19.54 | 137-144 |  | + | 1 |
| **108** | SMKEGIHAQQ | 10 | Alpha S1 Casein | 2 | 564.78 | 1127.54 | 18.04 | 137-146 |  | + | 1 |
| **109** | VAPFPEVFGKEKVN | 14 | Alpha S1 Casein | 2 | 780.92 | 1559.83 | 42.71 | 40-53 |  | + | 1 |
| **110** | VAPFPEVFGKEKVNE | 15 | Alpha S1 Casein | 2 | 845.45 | 1688.88 | 42.70 | 40-54 |  | + | 1 |
| **111** | VAPFPEVFGKEKVNEL | 16 | Alpha S1 Casein | 2 | 901.99 | 1801.96 | 44.24 | 40-55 |  | + | 1 |
| **112** | VFGKEKVN | 8 | Alpha S1 Casein | 2 | 460.76 | 919.51 | 19.36 | 46-53 |  | + | 1 |
| **113** | VFGKEKVNE | 9 | Alpha S1 Casein | 2 | 525.28 | 1048.56 | 20.96 | 46-54 |  | + | 1 |
| **114** | VFGKEKVNEL | 10 | Alpha S1 Casein | 2 | 581.83 | 1161.64 | 31.10 | 46-55 |  | + | 1 |
| **115** | VLNENLLRF | 9 | Alpha S1 Casein | 2 | 559.32 | 1116.63 | 45.42 | 30-38 |  | + | 1 |
| **116** | YKVPQLEIVPNSAEE | 15 | Alpha S1 Casein | 2 | 858.45 | 1714.88 | 43.96 | 119-133 |  | + | 1 |
| **117** | ALPQYLKTVYQHQ | 13 | Alpha S2 casein | 2 | 794.93 | 1587.84 | 41.66 | 190-202 | + | + | 2 |
| **118** | EEEKNRLNFL | 10 | Alpha S2 casein | 2 | 646.34 | 1290.66 | 40.55 | 170-179 | + | + | 2 |
| **119** | GPIVLNPWDQVK | 12 | Alpha S2 casein | 2 | 683.38 | 1364.75 | 45.11 | 117-128 | + | + | 2 |
| **120** | IQPKTKVIPY | 10 | Alpha S2 casein | 2 | 593.86 | 1185.71 | 32.07 | 209-218 | + |  | 1 |
| **121** | KFF | 3 | Alpha S2 casein | 1 | 441.25 | 440.24 | 61.64 | 2-4 | + |  | 1 |
| **122** | KKT | 3 | Alpha S2 casein | 1 | 376.26 | 375.25 | 50.14 | 331-333 | + |  | 1 |
| **123** | MKF | 3 | Alpha S2 casein | 1 | 425.22 | 424.21 | 56.11 | 1-3 | + |  | 1 |
| **124** | QGPIVLNPWDQVK | 13 | Alpha S2 casein | 2 | 747.41 | 1492.80 | 45.14 | 116-128 | + | + | 2 |
| **125** | RNA | 3 | Alpha S2 casein | 1 | 360.20 | 359.19 | 60.97 | 691-693 | + |  | 1 |
| **126** | VFT | 3 | Alpha S2 casein | 1 | 366.20 | 365.20 | 57.04 | 161-163 | + |  | 1 |
| **127** | VKR | 3 | Alpha S2 casein | 1 | 402.28 | 401.28 | 50.94 | 572-574 | + |  | 1 |
| **128** | VVR | 3 | Alpha S2 casein | 1 | 373.26 | 372.25 | 60.73 | 833-835 | + |  | 1 |
| **129** | YLY | 3 | Alpha S2 casein | 1 | 458.23 | 457.22 | 28.36 | 322-324 | + |  | 1 |
| **130** | ALPQYLK | 7 | Alpha S2 Casein | 2 | 416.75 | 831.49 | 36.44 | 190-196 |  | + | 1 |
| **131** | ALPQYLKT | 8 | Alpha S2 Casein | 2 | 467.27 | 932.53 | 38.75 | 190-197 |  | + | 1 |
| **132** | ALPQYLKTVY | 10 | Alpha S2 Casein | 2 | 598.34 | 1194.66 | 43.54 | 190-199 |  | + | 1 |
| **133** | ALPQYLKTVYQ | 11 | Alpha S2 Casein | 2 | 662.37 | 1322.72 | 43.17 | 190-200 |  | + | 1 |
| **134** | ALPQYLKTVYQH | 12 | Alpha S2 Casein | 2 | 730.90 | 1459.78 | 41.66 | 190-201 |  | + | 1 |
| **135** | AMKPWIQPKT | 10 | Alpha S2 Casein | 2 | 600.33 | 1198.65 | 33.61 | 204-213 |  | + | 1 |
| **136** | AVPITPTLNR | 10 | Alpha S2 Casein | 2 | 541.32 | 1080.63 | 37.94 | 131-140 |  | + | 1 |
| **137** | EEEKNRLNF | 9 | Alpha S2 Casein | 2 | 589.79 | 1177.57 | 29.58 | 170-178 |  | + | 1 |
| **138** | EEKNRLNF | 8 | Alpha S2 Casein | 2 | 525.27 | 1048.53 | 28.54 | 171-178 |  | + | 1 |
| **139** | EEKNRLNFL | 9 | Alpha S2 Casein | 2 | 581.81 | 1161.61 | 39.81 | 171-179 |  | + | 1 |
| **140** | EKNRLNF | 7 | Alpha S2 Casein | 2 | 460.75 | 919.49 | 27.07 | 172-178 |  | + | 1 |
| **141** | EKNRLNFL | 8 | Alpha S2 Casein | 2 | 517.29 | 1032.57 | 38.57 | 172-179 |  | + | 1 |
| **142** | EVKITVDDK | 9 | Alpha S2 Casein | 2 | 523.79 | 1045.57 | 27.06 | 83-91 |  | + | 1 |
| **143** | FALPQYLK | 8 | Alpha S2 Casein | 2 | 490.28 | 978.55 | 44.11 | 189-196 |  | + | 1 |
| **144** | FALPQYLKT | 9 | Alpha S2 Casein | 2 | 540.81 | 1079.60 | 44.36 | 189-197 |  | + | 1 |
| **145** | FLKKISQ | 7 | Alpha S2 Casein | 2 | 432.27 | 862.53 | 22.74 | 178-184 |  | + | 1 |
| **146** | IQPKTKVIP | 9 | Alpha S2 Casein | 2 | 512.33 | 1022.65 | 26.96 | 209-217 |  | + | 1 |
| **147** | IQPKTKVIPYV | 11 | Alpha S2 Casein | 2 | 643.40 | 1284.78 | 37.89 | 209-219 |  | + | 1 |
| **148** | ISQRYQ | 6 | Alpha S2 Casein | 2 | 397.71 | 793.41 | 17.68 | 182-187 |  | + | 1 |
| **149** | ISQRYQK | 7 | Alpha S2 Casein | 2 | 461.76 | 921.50 | 14.57 | 182-188 |  | + | 1 |
| **150** | ITPTLNR | 7 | Alpha S2 Casein | 2 | 407.74 | 813.47 | 27.49 | 134-140 |  | + | 1 |
| **151** | ITVDDKHYQ | 9 | Alpha S2 Casein | 2 | 559.78 | 1117.54 | 24.12 | 86-94 |  | + | 1 |
| **152** | KFALPQY | 7 | Alpha S2 Casein | 2 | 433.74 | 865.47 | 42.26 | 188-194 |  | + | 1 |
| **153** | KISQRYQ | 7 | Alpha S2 Casein | 2 | 461.76 | 921.50 | 15.41 | 181-187 |  | + | 1 |
| **154** | KNRLNF | 6 | Alpha S2 Casein | 2 | 396.23 | 790.44 | 24.70 | 173-178 |  | + | 1 |
| **155** | KTVYQHQ | 7 | Alpha S2 Casein | 2 | 452.24 | 902.46 | 14.10 | 196-202 |  | + | 1 |
| **156** | LKTVYQH | 7 | Alpha S2 Casein | 2 | 444.75 | 887.49 | 20.23 | 195-201 |  | + | 1 |
| **157** | LKTVYQHQ | 8 | Alpha S2 Casein | 2 | 508.78 | 1015.55 | 19.88 | 195-202 |  | + | 1 |
| **158** | LPQY | 4 | Alpha S2 Casein | 1 | 520.28 | 519.27 | 34.10 | 191-194 |  | + | 1 |
| **159** | LPQYLK | 6 | Alpha S2 Casein | 2 | 381.23 | 760.45 | 33.91 | 191-196 |  | + | 1 |
| **160** | LTEEEKNRLN | 10 | Alpha S2 Casein | 2 | 623.33 | 1244.64 | 20.01 | 168-177 |  | + | 1 |
| **161** | LTEEEKNRLNF | 11 | Alpha S2 Casein | 2 | 696.86 | 1391.70 | 33.49 | 168-178 |  | + | 1 |
| **162** | LYQGPIVLNPWDQVK | 15 | Alpha S2 Casein | 2 | 885.48 | 1768.95 | 46.83 | 114-128 |  | + | 1 |
| **163** | NAVPITPTLNR | 11 | Alpha S2 Casein | 2 | 598.34 | 1194.67 | 38.81 | 130-140 |  | + | 1 |
| **164** | PWDQVK | 6 | Alpha S2 Casein | 2 | 386.70 | 771.39 | 27.41 | 123-128 |  | + | 1 |
| **165** | PWIQPK | 6 | Alpha S2 Casein | 2 | 384.72 | 767.43 | 32.00 | 207-212 |  | + | 1 |
| **166** | PWIQPKT | 7 | Alpha S2 Casein | 2 | 435.25 | 868.48 | 34.11 | 207-213 |  | + | 1 |
| **167** | PWIQPKTKVIPY | 12 | Alpha S2 Casein | 2 | 735.43 | 1468.84 | 41.94 | 207-218 |  | + | 1 |
| **168** | QGPIVLNPWDQV | 12 | Alpha S2 Casein | 2 | 683.36 | 1364.71 | 49.75 | 116-127 |  | + | 1 |
| **169** | QKFALPQY | 8 | Alpha S2 Casein | 2 | 497.77 | 993.53 | 42.03 | 187-194 |  | + | 1 |
| **170** | QKFALPQYL | 9 | Alpha S2 Casein | 2 | 554.31 | 1106.61 | 44.88 | 187-195 |  | + | 1 |
| **171** | QKFALPQYLK | 10 | Alpha S2 Casein | 2 | 618.36 | 1234.71 | 41.66 | 187-196 |  | + | 1 |
| **172** | QKFPQYL | 7 | Alpha S2 Casein | 2 | 462.25 | 922.49 | 41.35 | 105-111 |  | + | 1 |
| **173** | QKFPQYLQY | 9 | Alpha S2 Casein | 2 | 607.81 | 1213.61 | 42.49 | 105-113 |  | + | 1 |
| **174** | QYLKTVYQH | 9 | Alpha S2 Casein | 2 | 590.31 | 1178.61 | 27.87 | 193-201 |  | + | 1 |
| **175** | RNAVPITPT | 9 | Alpha S2 Casein | 2 | 484.78 | 967.55 | 30.57 | 129-137 |  | + | 1 |
| **176** | RNAVPITPTL | 10 | Alpha S2 Casein | 2 | 541.32 | 1080.63 | 42.14 | 129-138 |  | + | 1 |
| **177** | RNAVPITPTLN | 11 | Alpha S2 Casein | 2 | 598.34 | 1194.67 | 39.66 | 129-139 |  | + | 1 |
| **178** | TEEEKNRL | 8 | Alpha S2 Casein | 2 | 509.76 | 1017.51 | 16.25 | 169-176 |  | + | 1 |
| **179** | TEEEKNRLN | 9 | Alpha S2 Casein | 2 | 566.78 | 1131.55 | 15.01 | 169-177 |  | + | 1 |
| **180** | TEEEKNRLNF | 10 | Alpha S2 Casein | 2 | 640.32 | 1278.62 | 29.61 | 169-178 |  | + | 1 |
| **181** | TEEEKNRLNFL | 11 | Alpha S2 Casein | 2 | 696.86 | 1391.70 | 40.09 | 169-179 |  | + | 1 |
| **182** | TKVIPYVR | 8 | Alpha S2 Casein | 2 | 488.30 | 974.59 | 30.69 | 213-220 |  | + | 1 |
| **183** | TKVIPYVRYL | 10 | Alpha S2 Casein | 2 | 626.38 | 1250.74 | 42.58 | 213-222 |  | + | 1 |
| **184** | TVYQHQK | 7 | Alpha S2 Casein | 2 | 452.24 | 902.46 | 13.61 | 197-203 |  | + | 1 |
| **185** | VIPYVR | 6 | Alpha S2 Casein | 2 | 373.73 | 745.45 | 34.68 | 215-220 |  | + | 1 |
| **186** | VIPYVRY | 7 | Alpha S2 Casein | 2 | 455.26 | 908.51 | 41.39 | 215-221 |  | + | 1 |
| **187** | VIPYVRYL | 8 | Alpha S2 Casein | 2 | 511.81 | 1021.60 | 44.88 | 215-222 |  | + | 1 |
| **188** | VLNPWDQVK | 9 | Alpha S2 Casein | 2 | 549.80 | 1097.59 | 42.44 | 120-128 |  | + | 1 |
| **189** | VPITPTLNR | 9 | Alpha S2 Casein | 2 | 505.80 | 1009.59 | 35.94 | 132-140 |  | + | 1 |
| **190** | VPITPTLNRE | 10 | Alpha S2 Casein | 2 | 570.32 | 1138.63 | 36.43 | 132-141 |  | + | 1 |
| **191** | WIQPKTKVIP | 10 | Alpha S2 Casein | 2 | 605.37 | 1208.73 | 37.44 | 208-217 |  | + | 1 |
| **192** | WIQPKTKVIPY | 11 | Alpha S2 Casein | 2 | 686.90 | 1371.79 | 41.00 | 208-218 |  | + | 1 |
| **193** | WIQPKTKVIPYV | 12 | Alpha S2 Casein | 2 | 736.44 | 1470.86 | 42.62 | 208-219 |  | + | 1 |
| **194** | YLKTVYQH | 8 | Alpha S2 Casein | 2 | 526.28 | 1050.55 | 24.80 | 194-201 |  | + | 1 |
| **195** | YQGPIVLNPWDQVK | 14 | Alpha S2 Casein | 2 | 828.94 | 1655.87 | 45.88 | 115-128 |  | + | 1 |
| **196** | YQKFALP | 7 | Alpha S2 Casein | 2 | 433.74 | 865.47 | 42.26 | 186-192 |  | + | 1 |
| **197** | YQKFALPQ | 8 | Alpha S2 Casein | 2 | 497.77 | 993.53 | 39.47 | 186-193 |  | + | 1 |
| **198** | YQKFALPQY | 9 | Alpha S2 Casein | 2 | 579.30 | 1156.59 | 42.81 | 186-194 |  | + | 1 |
| **199** | YQKFALPQYL | 10 | Alpha S2 Casein | 2 | 635.85 | 1269.68 | 45.37 | 186-195 |  | + | 1 |
| **200** | YQKFALPQYLK | 11 | Alpha S2 Casein | 2 | 699.89 | 1397.77 | 42.38 | 186-196 |  | + | 1 |
| **201** | YQKFPQY | 7 | Alpha S2 Casein | 2 | 487.24 | 972.47 | 34.36 | 104-110 |  | + | 1 |
| **202** | DELQDKIHPF | 10 | Beta casein | 2 | 621.31 | 1240.61 | 41.82 | 58-67 | + | + | 2 |
| **203** | DKIHPFAQT | 9 | Beta casein | 2 | 528.78 | 1055.54 | 29.08 | 62-70 | + | + | 2 |
| **204** | DKIHPFAQTQ | 10 | Beta casein | 2 | 592.81 | 1183.60 | 28.32 | 62-71 | + | + | 2 |
| **205** | DKIHPFAQTQS | 11 | Beta casein | 2 | 636.32 | 1270.63 | 28.11 | 62-72 | + | + | 2 |
| **206** | EEL | 3 | Beta casein | 1 | 390.19 | 389.18 | 46.75 | 19-21 | + |  | 1 |
| **207** | EMPFPKYPVEPF | 12 | Beta casein | 2 | 740.86 | 1479.71 | 46.98 | 123-134 | + |  | 1 |
| **208** | EMPFPKYPVEPFTE | 14 | Beta casein | 2 | 855.91 | 1709.80 | 46.34 | 123-136 | + |  | 1 |
| **209** | EPVLGPVRGPFP | 12 | Beta casein | 2 | 632.86 | 1263.70 | 44.78 | 210-221 | + | + | 2 |
| **210** | EPVLGPVRGPFPI | 13 | Beta casein | 2 | 689.40 | 1376.78 | 47.23 | 210-222 | + | + | 2 |
| **211** | EPVLGPVRGPFPII | 14 | Beta casein | 2 | 745.94 | 1489.87 | 49.19 | 210-223 | + | + | 2 |
| **212** | EPVLGPVRGPFPIIV | 15 | Beta casein | 2 | 795.47 | 1588.93 | 50.09 | 210-224 | + | + | 2 |
| **213** | FPKYPVEP | 8 | Beta casein | 2 | 488.76 | 975.51 | 40.44 | 126-133 | + |  | 1 |
| **214** | GPVRGPFPIIV | 11 | Beta casein | 2 | 576.35 | 1150.69 | 46.99 | 214-224 | + |  | 1 |
| **215** | LGPVRGPFPIIV | 12 | Beta casein | 2 | 632.89 | 1263.77 | 48.43 | 213-224 | + | + | 2 |
| **216** | LPVPQKAVPYPQ | 12 | Beta casein | 2 | 668.88 | 1335.76 | 40.55 | 186-197 | + | + | 2 |
| **217** | MPFPKYPVEP | 10 | Beta casein | 2 | 602.81 | 1203.60 | 43.45 | 124-133 | + | + | 2 |
| **218** | MPFPKYPVEPF | 11 | Beta casein | 2 | 676.34 | 1350.67 | 46.25 | 124-134 | + |  | 1 |
| **219** | MPFPKYPVEPFTESQSL | 17 | Beta casein | 2 | 998.99 | 1995.97 | 46.50 | 124-140 | + |  | 1 |
| **220** | QEPVLGPVRGPFPII | 15 | Beta casein | 2 | 809.97 | 1617.92 | 48.86 | 209-223 | + | + | 2 |
| **221** | QEPVLGPVRGPFPIIV | 16 | Beta casein | 2 | 859.50 | 1716.99 | 49.74 | 209-224 | + | + | 2 |
| **222** | QQQ | 3 | Beta casein | 1 | 403.19 | 402.19 | 41.02 | 53-55 | + |  | 1 |
| **223** | QQQTEDELQDK | 11 | Beta casein | 2 | 681.31 | 1360.61 | 22.28 | 53-63 | + | + | 2 |
| **224** | QTEDELQDK | 9 | Beta casein | 2 | 553.25 | 1104.49 | 21.01 | 55-63 | + |  | 1 |
| **225** | TEDELQDK | 8 | Beta casein | 2 | 489.22 | 976.43 | 20.16 | 56-63 | + | + | 2 |
| **226** | VLGPVRGPFPIIV | 13 | Beta casein | 2 | 682.43 | 1362.84 | 48.95 | 212-224 | + | + | 2 |
| **227** | VLPVPQKAVPYPQ | 13 | Beta casein | 2 | 718.42 | 1434.82 | 41.86 | 185-197 | + | + | 2 |
| **228** | VPYPQRDMPIQA | 12 | Beta casein | 2 | 707.86 | 1413.71 | 40.69 | 193-204 | + | + | 2 |
| **229** | VPYPQRDMPIQAF | 13 | Beta casein | 2 | 781.40 | 1560.78 | 44.23 | 193-205 | + |  | 1 |
| **230** | YQEPVLGPVRGPFP | 14 | Beta casein | 2 | 778.42 | 1554.82 | 45.17 | 208-221 | + | + | 2 |
| **231** | YQEPVLGPVRGPFPIIV | 17 | Beta casein | 2 | 941.04 | 1880.06 | 50.20 | 208-224 | + | + | 2 |
| **232** | AVPYPQRDMP | 10 | Beta Casein | 2 | 587.29 | 1172.56 | 35.91 | 192-201 |  | + | 1 |
| **233** | AVPYPQRDMPIQA | 13 | Beta Casein | 2 | 743.38 | 1484.74 | 41.43 | 192-204 |  | + | 1 |
| **234** | DELQDKIHPFA | 11 | Beta Casein | 2 | 656.83 | 1311.65 | 41.66 | 58-68 |  | + | 1 |
| **235** | DELQDKIHPFAQTQ | 14 | Beta Casein | 2 | 835.41 | 1668.81 | 41.04 | 58-71 |  | + | 1 |
| **236** | DKIHPF | 6 | Beta Casein | 2 | 378.71 | 755.40 | 28.95 | 62-67 |  | + | 1 |
| **237** | DKIHPFA | 7 | Beta Casein | 2 | 414.22 | 826.43 | 28.78 | 62-68 |  | + | 1 |
| **238** | DKIHPFAQ | 8 | Beta Casein | 2 | 478.25 | 954.49 | 28.05 | 62-69 |  | + | 1 |
| **239** | DKIHPFAQTQSL | 12 | Beta Casein | 2 | 692.86 | 1383.71 | 37.79 | 62-73 |  | + | 1 |
| **240** | DVENLHLP | 8 | Beta Casein | 2 | 468.74 | 935.47 | 43.69 | 144-151 |  | + | 1 |
| **241** | DVENLHLPLP | 10 | Beta Casein | 2 | 573.81 | 1145.61 | 47.56 | 144-153 |  | + | 1 |
| **242** | DVENLHLPLPL | 11 | Beta Casein | 2 | 630.35 | 1258.69 | 50.78 | 144-154 |  | + | 1 |
| **243** | DVENLHLPLPLL | 12 | Beta Casein | 2 | 686.90 | 1371.78 | 53.20 | 144-155 |  | + | 1 |
| **244** | DVENLHLPLPLLQ | 13 | Beta Casein | 2 | 750.92 | 1499.83 | 52.01 | 144-156 |  | + | 1 |
| **245** | DVENLHLPLPLLQS | 14 | Beta Casein | 2 | 794.44 | 1586.87 | 51.59 | 144-157 |  | + | 1 |
| **246** | EDELQDKIHPFAQTQ | 15 | Beta Casein | 2 | 899.93 | 1797.85 | 40.90 | 57-71 |  | + | 1 |
| **247** | ELQDKIHPF | 9 | Beta Casein | 2 | 563.80 | 1125.58 | 37.90 | 59-67 |  | + | 1 |
| **248** | ELQDKIHPFAQTQ | 13 | Beta Casein | 2 | 777.90 | 1553.78 | 36.52 | 59-71 |  | + | 1 |
| **249** | ELQDKIHPFAQTQS | 14 | Beta Casein | 2 | 821.42 | 1640.82 | 36.27 | 59-72 |  | + | 1 |
| **250** | EMPFPKY | 7 | Beta Casein | 2 | 456.22 | 910.43 | 42.03 | 123-129 |  | + | 1 |
| **251** | ENLHLPLPLLQ | 11 | Beta Casein | 2 | 643.88 | 1285.74 | 49.66 | 146-156 |  | + | 1 |
| **252** | ENLHLPLPLLQS | 12 | Beta Casein | 2 | 687.39 | 1372.77 | 49.37 | 146-157 |  | + | 1 |
| **253** | EPVLGPVRGP | 10 | Beta Casein | 2 | 510.80 | 1019.58 | 39.10 | 210-219 |  | + | 1 |
| **254** | EVMGVSKVKEA | 11 | Beta Casein | 2 | 588.82 | 1175.62 | 26.21 | 106-116 |  | + | 1 |
| **255** | FPKYPVEPFTESQS | 14 | Beta Casein | 2 | 828.40 | 1654.79 | 43.59 | 126-139 |  | + | 1 |
| **256** | FQSEEQQQTEDELQDK | 16 | Beta Casein | 2 | 991.43 | 1980.85 | 31.73 | 48-63 |  | + | 1 |
| **257** | GPVRGPFP | 8 | Beta Casein | 2 | 413.73 | 825.45 | 37.13 | 214-221 |  | + | 1 |
| **258** | HLPLPLLQS | 9 | Beta Casein | 2 | 509.31 | 1016.60 | 46.28 | 149-157 |  | + | 1 |
| **259** | IHPFAQTQ | 8 | Beta Casein | 2 | 471.25 | 940.48 | 29.43 | 64-71 |  | + | 1 |
| **260** | IHPFAQTQS | 9 | Beta Casein | 2 | 514.76 | 1027.51 | 29.13 | 64-72 |  | + | 1 |
| **261** | IHPFAQTQSL | 10 | Beta Casein | 2 | 571.30 | 1140.59 | 39.62 | 64-73 |  | + | 1 |
| **262** | KEMPFPKYPVEP | 12 | Beta Casein | 2 | 731.38 | 1460.74 | 41.39 | 122-133 |  | + | 1 |
| **263** | KVKEAMAP | 8 | Beta Casein | 2 | 437.25 | 872.48 | 18.48 | 112-119 |  | + | 1 |
| **264** | KVKEAMAPK | 9 | Beta Casein | 2 | 501.29 | 1000.57 | 15.40 | 112-120 |  | + | 1 |
| **265** | KVLPVPQK | 8 | Beta Casein | 2 | 454.80 | 907.59 | 24.61 | 184-191 |  | + | 1 |
| **266** | KYPVEPFTESQ | 11 | Beta Casein | 2 | 662.82 | 1323.63 | 39.76 | 128-138 |  | + | 1 |
| **267** | KYPVEPFTESQS | 12 | Beta Casein | 2 | 706.34 | 1410.67 | 39.32 | 128-139 |  | + | 1 |
| **268** | LGPVRGPFP | 9 | Beta Casein | 2 | 470.27 | 938.53 | 42.62 | 213-221 |  | + | 1 |
| **269** | LHLPLPLLQ | 9 | Beta Casein | 2 | 522.33 | 1042.65 | 49.36 | 148-156 |  | + | 1 |
| **270** | LHLPLPLLQS | 10 | Beta Casein | 2 | 565.85 | 1129.69 | 49.08 | 148-157 |  | + | 1 |
| **271** | LPPTVMFPPQS | 11 | Beta Casein | 2 | 607.32 | 1212.62 | 46.51 | 166-176 |  | + | 1 |
| **272** | LPPTVMFPPQSVL | 13 | Beta Casein | 2 | 713.39 | 1424.77 | 51.26 | 166-178 |  | + | 1 |
| **273** | LQDKIHPFAQTQ | 12 | Beta Casein | 2 | 713.38 | 1424.74 | 32.27 | 60-71 |  | + | 1 |
| **274** | LSSSEESITRIN | 12 | Beta Casein | 2 | 668.34 | 1334.67 | 34.00 | 31-42 |  | + | 1 |
| **275** | LTDVENLH | 8 | Beta Casein | 2 | 470.74 | 939.47 | 31.62 | 142-149 |  | + | 1 |
| **276** | LYQEPVLGPVR | 11 | Beta Casein | 2 | 635.86 | 1269.71 | 41.77 | 207-217 |  | + | 1 |
| **277** | LYQEPVLGPVRGP | 13 | Beta Casein | 2 | 712.90 | 1423.78 | 42.81 | 207-219 |  | + | 1 |
| **278** | LYQEPVLGPVRGPFP | 15 | Beta Casein | 2 | 834.96 | 1667.90 | 45.88 | 207-221 |  | + | 1 |
| **279** | LYQEPVLGPVRGPFPIIV | 18 | Beta Casein | 2 | 997.58 | 1993.14 | 50.76 | 207-224 |  | + | 1 |
| **280** | MPFPKY | 6 | Beta Casein | 2 | 391.70 | 781.38 | 40.36 | 124-129 |  | + | 1 |
| **281** | NLHLPLPL | 8 | Beta Casein | 2 | 458.78 | 915.55 | 47.94 | 147-154 |  | + | 1 |
| **282** | NVPGEIVESLSSSEESITR | 19 | Beta Casein | 2 | 1017.01 | 2032.00 | 45.76 | 22-40 |  | + | 1 |
| **283** | NVPGEIVESLSSSEESITRIN | 21 | Beta Casein | 2 | 1130.57 | 2259.12 | 47.27 | 22-42 |  | + | 1 |
| **284** | PPF | 3 | Beta Casein | 1 | 360.19 | 359.18 | 43.05 | 100-102 |  | + | 1 |
| **285** | PVEPFTESQ | 9 | Beta Casein | 2 | 517.25 | 1032.48 | 40.35 | 130-138 |  | + | 1 |
| **286** | PVEPFTESQS | 10 | Beta Casein | 2 | 560.76 | 1119.51 | 39.85 | 130-139 |  | + | 1 |
| **287** | PVEPFTESQSL | 11 | Beta Casein | 2 | 617.30 | 1232.59 | 44.64 | 130-140 |  | + | 1 |
| **288** | PVEPFTESQSLT | 12 | Beta Casein | 2 | 667.83 | 1333.64 | 44.11 | 130-141 |  | + | 1 |
| **289** | PVEPFTESQSLTLT | 14 | Beta Casein | 2 | 774.89 | 1547.77 | 46.60 | 130-143 |  | + | 1 |
| **290** | PVEPFTESQSLTLTDVEN | 18 | Beta Casein | 2 | 1003.48 | 2004.95 | 46.87 | 130-147 |  | + | 1 |
| **291** | PVLGPVRGPFPIIV | 14 | Beta Casein | 2 | 730.95 | 1459.89 | 49.70 | 211-224 |  | + | 1 |
| **292** | PVPQKAVPYPQ | 11 | Beta Casein | 2 | 612.34 | 1222.67 | 32.75 | 187-197 |  | + | 1 |
| **293** | QDKIHPF | 7 | Beta Casein | 2 | 442.73 | 883.46 | 28.31 | 61-67 |  | + | 1 |
| **294** | QDKIHPFAQTQS | 12 | Beta Casein | 2 | 700.35 | 1398.69 | 27.56 | 61-72 |  | + | 1 |
| **295** | QEPVLGPVR | 9 | Beta Casein | 2 | 497.79 | 993.56 | 34.55 | 209-217 |  | + | 1 |
| **296** | QEPVLGPVRGP | 11 | Beta Casein | 2 | 574.82 | 1147.63 | 39.24 | 209-219 |  | + | 1 |
| **297** | QEPVLGPVRGPF | 12 | Beta Casein | 2 | 648.36 | 1294.70 | 44.30 | 209-220 |  | + | 1 |
| **298** | QEPVLGPVRGPFP | 13 | Beta Casein | 2 | 696.89 | 1391.76 | 44.58 | 209-221 |  | + | 1 |
| **299** | QEPVLGPVRGPFPI | 14 | Beta Casein | 2 | 753.43 | 1504.84 | 46.95 | 209-222 |  | + | 1 |
| **300** | QQTEDELQDK | 10 | Beta Casein | 2 | 617.28 | 1232.55 | 22.01 | 54-63 |  | + | 1 |
| **301** | QRDMPIQ | 7 | Beta Casein | 2 | 444.22 | 886.43 | 26.66 | 197-203 |  | + | 1 |
| **302** | QSKVLPVPQ | 9 | Beta Casein | 2 | 498.30 | 994.58 | 34.70 | 182-190 |  | + | 1 |
| **303** | RDMPIQA | 7 | Beta Casein | 2 | 415.71 | 829.41 | 30.23 | 198-204 |  | + | 1 |
| **304** | RDMPIQAF | 8 | Beta Casein | 2 | 489.25 | 976.48 | 42.49 | 198-205 |  | + | 1 |
| **305** | RDMPIQAFL | 9 | Beta Casein | 2 | 545.79 | 1089.56 | 46.56 | 198-206 |  | + | 1 |
| **306** | SEEQQQTEDELQDK | 14 | Beta Casein | 2 | 853.87 | 1705.73 | 26.26 | 50-63 |  | + | 1 |
| **307** | SLSQSKVLPVPQK | 13 | Beta Casein | 2 | 705.92 | 1409.82 | 32.67 | 179-191 |  | + | 1 |
| **308** | SLVYPFPGPIPN | 12 | Beta Casein | 2 | 650.85 | 1299.69 | 49.78 | 72-83 |  | + | 1 |
| **309** | SSEESITRIN | 10 | Beta Casein | 2 | 568.28 | 1134.55 | 29.35 | 33-42 |  | + | 1 |
| **310** | SSSEESITRIN | 11 | Beta Casein | 2 | 611.80 | 1221.58 | 29.62 | 32-42 |  | + | 1 |
| **311** | TDVENLH | 7 | Beta Casein | 2 | 414.20 | 826.38 | 24.49 | 143-149 |  | + | 1 |
| **312** | TDVENLHLP | 9 | Beta Casein | 2 | 519.27 | 1036.52 | 43.12 | 143-151 |  | + | 1 |
| **313** | TDVENLHLPLP | 11 | Beta Casein | 2 | 624.34 | 1246.66 | 46.54 | 143-153 |  | + | 1 |
| **314** | TDVENLHLPLPL | 12 | Beta Casein | 2 | 680.88 | 1359.74 | 49.51 | 143-154 |  | + | 1 |
| **315** | TDVENLHLPLPLL | 13 | Beta Casein | 2 | 737.42 | 1472.82 | 51.77 | 143-155 |  | + | 1 |
| **316** | TDVENLHLPLPLLQ | 14 | Beta Casein | 2 | 801.45 | 1600.88 | 50.76 | 143-156 |  | + | 1 |
| **317** | TDVENLHLPLPLLQS | 15 | Beta Casein | 2 | 844.96 | 1687.91 | 50.40 | 143-157 |  | + | 1 |
| **318** | TEDELQDKIHP | 11 | Beta Casein | 2 | 662.82 | 1323.63 | 32.75 | 56-66 |  | + | 1 |
| **319** | TEDELQDKIHPF | 12 | Beta Casein | 2 | 736.36 | 1470.70 | 41.49 | 56-67 |  | + | 1 |
| **320** | TEDELQDKIHPFAQTQ | 16 | Beta Casein | 2 | 950.46 | 1898.90 | 40.70 | 56-71 |  | + | 1 |
| **321** | VENLHLPLPL | 10 | Beta Casein | 2 | 572.84 | 1143.67 | 48.29 | 145-154 |  | + | 1 |
| **322** | VENLHLPLPLLQS | 13 | Beta Casein | 2 | 736.93 | 1471.84 | 49.47 | 145-157 |  | + | 1 |
| **323** | VEPFTESQS | 9 | Beta Casein | 2 | 512.24 | 1022.46 | 37.38 | 131-139 |  | + | 1 |
| **324** | VKEAMAPK | 8 | Beta Casein | 2 | 437.25 | 872.48 | 16.18 | 113-120 |  | + | 1 |
| **325** | VLGPVRGP | 8 | Beta Casein | 2 | 397.75 | 793.48 | 33.64 | 212-219 |  | + | 1 |
| **326** | VLGPVRGPF | 9 | Beta Casein | 2 | 471.28 | 940.55 | 42.93 | 212-220 |  | + | 1 |
| **327** | VLGPVRGPFP | 10 | Beta Casein | 2 | 519.81 | 1037.60 | 43.38 | 212-221 |  | + | 1 |
| **328** | VLGPVRGPFPII | 12 | Beta Casein | 2 | 632.89 | 1263.77 | 47.95 | 212-223 |  | + | 1 |
| **329** | VLPVPQK | 7 | Beta Casein | 2 | 390.75 | 779.49 | 29.52 | 185-191 |  | + | 1 |
| **330** | VLPVPQKAVP | 10 | Beta Casein | 2 | 524.33 | 1046.65 | 39.44 | 185-194 |  | + | 1 |
| **331** | VLPVPQKAVPYP | 12 | Beta Casein | 2 | 654.39 | 1306.76 | 42.62 | 185-196 |  | + | 1 |
| **332** | VLPVPQKAVPYPQR | 14 | Beta Casein | 2 | 796.47 | 1590.92 | 37.81 | 185-198 |  | + | 1 |
| **333** | VMGVSKVKEA | 10 | Beta Casein | 2 | 524.30 | 1046.58 | 23.33 | 107-116 |  | + | 1 |
| **334** | VPYPQRDMPIQ | 11 | Beta Casein | 2 | 672.34 | 1342.67 | 38.49 | 193-203 |  | + | 1 |
| **335** | YPF | 3 | Beta Casein | 1 | 426.20 | 425.20 | 42.78 | 75-77 |  | + | 1 |
| **336** | YPFPGP | 6 | Beta Casein | 1 | 677.33 | 676.32 | 44.41 | 75-80 |  | + | 1 |
| **337** | YPQRDMPIQ | 9 | Beta Casein | 2 | 574.28 | 1146.55 | 32.40 | 195-203 |  | + | 1 |
| **338** | YPQRDMPIQA | 10 | Beta Casein | 2 | 609.80 | 1217.59 | 35.39 | 195-204 |  | + | 1 |
| **339** | YPQRDMPIQAF | 11 | Beta Casein | 2 | 683.33 | 1364.65 | 43.38 | 195-205 |  | + | 1 |
| **340** | YQEPVLGPVR | 10 | Beta Casein | 2 | 579.32 | 1156.62 | 39.00 | 208-217 |  | + | 1 |
| **341** | YQEPVLGPVRG | 11 | Beta Casein | 2 | 607.83 | 1213.65 | 39.19 | 208-218 |  | + | 1 |
| **342** | YQEPVLGPVRGP | 12 | Beta Casein | 2 | 656.36 | 1310.70 | 41.77 | 208-219 |  | + | 1 |
| **343** | YQEPVLGPVRGPF | 13 | Beta Casein | 2 | 729.89 | 1457.77 | 44.94 | 208-220 |  | + | 1 |
| **344** | YQEPVLGPVRGPFPI | 15 | Beta Casein | 2 | 834.96 | 1667.90 | 47.55 | 208-222 |  | + | 1 |
| **345** | YQEPVLGPVRGPFPII | 16 | Beta Casein | 2 | 891.50 | 1780.99 | 49.36 | 208-223 |  | + | 1 |
| **346** | ALPMHIR | 7 | Beta Lactoglobulin | 2 | 419.24 | 836.47 | 30.32 | 142-148 |  | + | 1 |
| **347** | DEALEKFDKA | 10 | Beta Lactoglobulin | 2 | 583.29 | 1164.57 | 33.14 | 130-139 |  | + | 1 |
| **348** | TQTMKGLDIQ | 10 | Beta Lactoglobulin | 2 | 567.79 | 1133.58 | 36.49 | 4-13 |  | + | 1 |
| **349** | YKK | 3 | Beta Lactoglobulin | 1 | 438.27 | 437.26 | 59.74 | 391-393 |  | + | 1 |
| **350** | MKS | 3 | Kappa casein | 1 | 365.19 | 364.18 | 49.96 | 2-4 | + |  | 1 |
| **351** | SPPEINTVQV | 10 | Kappa casein | 2 | 542.29 | 1082.56 | 42.97 | 176-185 | + |  | 1 |
| **352** | SPPEINTVQVTSTAV | 15 | Kappa casein | 2 | 771.90 | 1541.79 | 43.75 | 176-190 | + | + | 2 |
| **353** | DKIAKYIPIQY | 11 | Kappa Casein | 2 | 676.38 | 1350.75 | 42.96 | 41-51 |  | + | 1 |
| **354** | DKTEIPTINT | 10 | Kappa Casein | 2 | 566.30 | 1130.58 | 40.03 | 136-145 |  | + | 1 |
| **355** | ERFFSDKIA | 9 | Kappa Casein | 2 | 556.79 | 1111.57 | 36.48 | 36-44 |  | + | 1 |
| **356** | ESPPEINTVQVT | 12 | Kappa Casein | 2 | 657.33 | 1312.65 | 42.33 | 175-186 |  | + | 1 |
| **357** | ESPPEINTVQVTSTAV | 16 | Kappa Casein | 2 | 836.43 | 1670.84 | 44.06 | 175-190 |  | + | 1 |
| **358** | FFS | 3 | Kappa Casein | 1 | 400.19 | 399.18 | 36.18 | 38-40 |  | + | 1 |
| **359** | IAKYIPIQYVL | 11 | Kappa Casein | 2 | 660.90 | 1319.79 | 47.16 | 43-53 |  | + | 1 |
| **360** | IPIQYVLSR | 9 | Kappa Casein | 2 | 544.83 | 1087.64 | 43.69 | 47-55 |  | + | 1 |
| **361** | KYIPIQY | 7 | Kappa Casein | 2 | 462.76 | 923.51 | 40.03 | 45-51 |  | + | 1 |
| **362** | KYIPIQYVLS | 10 | Kappa Casein | 2 | 612.36 | 1222.70 | 44.74 | 45-54 |  | + | 1 |
| **363** | KYIPIQYVLSR | 11 | Kappa Casein | 2 | 690.41 | 1378.80 | 42.81 | 45-55 |  | + | 1 |
| **364** | NQFLPYPYYAK | 11 | Kappa Casein | 2 | 702.35 | 1402.69 | 43.83 | 74-84 |  | + | 1 |
| **365** | QWQVL | 5 | Kappa Casein | 1 | 673.37 | 672.36 | 45.88 | 96-100 |  | + | 1 |
| **366** | RYPSYGLN | 8 | Kappa Casein | 2 | 485.24 | 968.47 | 33.02 | 55-62 |  | + | 1 |
| **367** | SPPEINTVQVT | 11 | Kappa Casein | 2 | 592.81 | 1183.61 | 41.83 | 176-186 |  | + | 1 |
| **368** | SRYPSYGLNY | 10 | Kappa Casein | 2 | 610.29 | 1218.57 | 39.93 | 54-63 |  | + | 1 |
| **369** | YQQKPVAL | 8 | Kappa Casein | 2 | 473.77 | 945.53 | 31.23 | 64-71 |  | + | 1 |
| **370** | YYQQKPVAL | 9 | Kappa Casein | 2 | 555.30 | 1108.59 | 34.89 | 63-71 |  | + | 1 |

**Supplementary Table 5.** Milk peptides identified throughout the 28 days of storage period using the *L. paraplantarum* NCCB 100523.

|  | **Sequence** | **Length** | **Leading proteins** | **Charge** | **m/z** | **Mass** | **Retention time** | **Sequence** | **Day 1** | **Day 28** | **Incidence** |
| --- | --- | --- | --- | --- | --- | --- | --- | --- | --- | --- | --- |
| **1** | APFPEVFGK | 9 | Alpha S1 Casein | 2 | 496.27 | 990.52 | 43.94 | 41-49 |  | + | 1 |
| **2** | APFPEVFGKE | 10 | Alpha S1 Casein | 2 | 560.79 | 1119.56 | 44.00 | 41-50 |  | + | 1 |
| **3** | APFPEVFGKEK | 11 | Alpha S1 Casein | 2 | 624.83 | 1247.66 | 41.02 | 41-51 |  | + | 1 |
| **4** | APFPEVFGKEKV | 12 | Alpha S1 Casein | 2 | 674.37 | 1346.72 | 42.37 | 41-52 |  | + | 1 |
| **5** | ERYLGYLEQ | 9 | Alpha S1 Casein | 2 | 585.79 | 1169.57 | 41.56 | 104-112 |  | + | 1 |
| **6** | EVLNENLLR | 9 | Alpha S1 Casein | 2 | 550.31 | 1098.60 | 41.95 | 29-37 |  | + | 1 |
| **7** | FFVAP | 5 | Alpha S1 Casein | 1 | 580.31 | 579.31 | 45.28 | 38-42 |  | + | 1 |
| **8** | FSDIPNPIGSENSE | 14 | Alpha S1 Casein | 2 | 753.34 | 1504.67 | 44.49 | 194-207 |  | + | 1 |
| **9** | FSDIPNPIGSENSEK | 15 | Alpha S1 Casein | 2 | 817.39 | 1632.76 | 41.87 | 194-208 |  | + | 1 |
| **10** | FSDIPNPIGSENSEKT | 16 | Alpha S1 Casein | 2 | 867.91 | 1733.81 | 41.94 | 194-209 |  | + | 1 |
| **11** | FSDIPNPIGSENSEKTT | 17 | Alpha S1 Casein | 2 | 918.44 | 1834.86 | 41.99 | 194-210 |  | + | 1 |
| **12** | FSDIPNPIGSENSEKTTMPLW | 21 | Alpha S1 Casein | 2 | 1182.06 | 2362.12 | 47.72 | 194-214 |  | + | 1 |
| **13** | FVAPFPEVFGK | 11 | Alpha S1 Casein | 2 | 619.33 | 1236.65 | 47.35 | 39-49 |  | + | 1 |
| **14** | GLPQEVLNENLLR | 13 | Alpha S1 Casein | 2 | 747.92 | 1493.82 | 45.25 | 25-37 |  | + | 1 |
| **15** | IPNPIGSENSEK | 12 | Alpha S1 Casein | 2 | 642.83 | 1283.64 | 32.70 | 197-208 |  | + | 1 |
| **16** | IPNPIGSENSEKTT | 14 | Alpha S1 Casein | 2 | 743.87 | 1485.73 | 33.72 | 197-210 |  | + | 1 |
| **17** | IPNPIGSENSEKTTMPLW | 18 | Alpha S1 Casein | 2 | 1015.50 | 2028.98 | 44.42 | 197-214 |  | + | 1 |
| **18** | KTTMPLW | 7 | Alpha S1 Casein | 2 | 438.74 | 875.46 | 43.47 | 208-214 |  | + | 1 |
| **19** | KYK | 3 | Alpha S1 Casein | 1 | 438.27 | 437.26 | 59.70 | 118-120 |  | + | 1 |
| **20** | LKK | 3 | Alpha S1 Casein | 1 | 388.29 | 387.28 | 61.45 | 116-118 |  | + | 1 |
| **21** | PIGSENSEKTT | 11 | Alpha S1 Casein | 2 | 581.78 | 1161.55 | 17.80 | 200-210 |  | + | 1 |
| **22** | PIGSENSEKTTMPL | 14 | Alpha S1 Casein | 2 | 752.37 | 1502.73 | 38.93 | 200-213 |  | + | 1 |
| **23** | PIGSENSEKTTMPLW | 15 | Alpha S1 Casein | 2 | 853.41 | 1704.80 | 42.41 | 200-214 |  | + | 1 |
| **24** | QEVLNENLLR | 10 | Alpha S1 Casein | 2 | 614.34 | 1226.66 | 42.10 | 28-37 |  | + | 1 |
| **25** | QGLPQEVLNENLLR | 14 | Alpha S1 Casein | 2 | 811.95 | 1621.88 | 45.13 | 24-37 |  | + | 1 |
| **26** | RLK | 3 | Alpha S1 Casein | 1 | 416.30 | 415.29 | 58.48 | 1006-1008 |  | + | 1 |
| **27** | RPK | 3 | Alpha S1 Casein | 1 | 400.27 | 399.26 | 57.83 | 16-18 |  | + | 1 |
| **28** | SDIPNPIGSENSEKTTMPLW | 20 | Alpha S1 Casein | 2 | 1108.53 | 2215.05 | 46.64 | 195-214 |  | + | 1 |
| **29** | SEKTTMPLW | 9 | Alpha S1 Casein | 2 | 546.77 | 1091.53 | 43.78 | 206-214 |  | + | 1 |
| **30** | SMK | 3 | Alpha S1 Casein | 1 | 365.19 | 364.18 | 45.85 | 137-139 |  | + | 1 |
| **31** | VAPFPEVFGK | 10 | Alpha S1 Casein | 2 | 545.80 | 1089.59 | 44.90 | 40-49 |  | + | 1 |
| **32** | VAPFPEVFGKE | 11 | Alpha S1 Casein | 2 | 610.32 | 1218.63 | 44.85 | 40-50 |  | + | 1 |
| **33** | VAPFPEVFGKEK | 12 | Alpha S1 Casein | 2 | 674.37 | 1346.72 | 42.37 | 40-51 |  | + | 1 |
| **34** | VAPFPEVFGKEKV | 13 | Alpha S1 Casein | 2 | 723.90 | 1445.79 | 43.20 | 40-52 |  | + | 1 |
| **35** | VAPFPEVFGKEKVN | 14 | Alpha S1 Casein | 2 | 780.92 | 1559.83 | 42.51 | 40-53 |  | + | 1 |
| **36** | YLE | 3 | Alpha S1 Casein | 1 | 424.21 | 423.20 | 52.67 | 109-111 |  | + | 1 |
| **37** | YQL | 3 | Alpha S1 Casein | 1 | 423.22 | 422.22 | 55.25 | 169-171 |  | + | 1 |
| **38** | RFF | 3 | Alpha S1 Casein | 1 | 469.26 | 468.25 | 38.80 | 37-39 |  | + | 1 |
| **39** | ALPQYLK | 7 | Alpha S2 Casein | 2 | 416.75 | 831.49 | 36.39 | 190-196 |  |  | 1 |
| **40** | ALPQYLKTVY | 10 | Alpha S2 Casein | 2 | 598.34 | 1194.66 | 43.50 | 190-199 |  | + | 1 |
| **41** | ALPQYLKTVYQ | 11 | Alpha S2 Casein | 2 | 662.37 | 1322.72 | 43.13 | 190-200 |  | + | 1 |
| **42** | ALPQYLKTVYQH | 12 | Alpha S2 Casein | 2 | 730.90 | 1459.78 | 41.69 | 190-201 |  | + | 1 |
| **43** | FALPQYLK | 8 | Alpha S2 Casein | 2 | 490.28 | 978.55 | 44.07 | 189-196 |  | + | 1 |
| **44** | FALPQYLKT | 9 | Alpha S2 Casein | 2 | 540.81 | 1079.60 | 44.37 | 189-197 |  | + | 1 |
| **45** | LPQYLKT | 7 | Alpha S2 Casein | 2 | 431.76 | 861.50 | 36.29 | 191-197 |  | + | 1 |
| **46** | LPQYLKTVYQ | 10 | Alpha S2 Casein | 2 | 626.85 | 1251.69 | 42.34 | 191-200 |  | + | 1 |
| **47** | MKF | 3 | Alpha S2 Casein | 1 | 425.22 | 424.21 | 56.03 | 1-3 |  | + | 1 |
| **48** | PWD | 3 | Alpha S2 Casein | 1 | 417.18 | 416.17 | 50.57 | 123-125 |  | + | 1 |
| **49** | VIPYVRY | 7 | Alpha S2 Casein | 2 | 455.26 | 908.51 | 41.37 | 215-221 |  | + | 1 |
| **50** | VPITPTLN | 8 | Alpha S2 Casein | 1 | 854.50 | 853.49 | 42.63 | 132-139 |  | + | 1 |
| **51** | VRN | 3 | Alpha S2 Casein | 1 | 388.23 | 387.22 | 58.31 | 59-61 |  | + | 1 |
| **52** | YLKTVYQ | 7 | Alpha S2 Casein | 2 | 457.75 | 913.49 | 29.87 | 194-200 |  | + | 1 |
| **53** | YQGPIVLNPWDQVK | 14 | Alpha S2 Casein | 2 | 828.94 | 1655.87 | 45.84 | 115-128 |  | + | 1 |
| **54** | YQKFALPQYL | 10 | Alpha S2 Casein | 2 | 635.85 | 1269.68 | 45.36 | 186-195 |  | + | 1 |
| **55** | YVR | 3 | Alpha S2 Casein | 1 | 437.25 | 436.24 | 48.41 | 218-220 |  | + | 1 |
| **56** | DKIHPFAQTQ | 10 | Beta Casein | 2 | 592.81 | 1183.60 | 28.18 | 62-71 | + |  | 1 |
| **57** | DKIHPFAQTQSL | 12 | Beta Casein | 2 | 692.86 | 1383.71 | 37.73 | 62-73 |  | + | 1 |
| **58** | EEL | 3 | Beta Casein | 1 | 390.19 | 389.18 | 46.29 | 19-21 |  | + | 1 |
| **59** | EPVLGPVRGPFP | 12 | Beta Casein | 2 | 632.86 | 1263.70 | 44.79 | 210-221 |  | + | 1 |
| **60** | EPVLGPVRGPFPI | 13 | Beta Casein | 2 | 689.40 | 1376.78 | 47.19 | 210-222 |  | + | 1 |
| **61** | EPVLGPVRGPFPII | 14 | Beta Casein | 2 | 745.94 | 1489.87 | 49.15 | 210-223 |  | + | 1 |
| **62** | EPVLGPVRGPFPIIV | 15 | Beta Casein | 2 | 795.47 | 1588.93 | 50.04 | 210-224 |  | + | 1 |
| **63** | GPVRGPFPIIV | 11 | Beta Casein | 2 | 576.35 | 1150.69 | 46.95 | 214-224 |  | + | 1 |
| **64** | LGPVRGPFPIIV | 12 | Beta Casein | 2 | 632.89 | 1263.77 | 48.41 | 213-224 |  | + | 1 |
| **65** | LPVPQKAVPYPQ | 12 | Beta Casein | 2 | 668.88 | 1335.76 | 40.46 | 186-197 |  | + | 1 |
| **66** | MPFPKYPVEP | 10 | Beta Casein | 2 | 602.81 | 1203.60 | 43.44 | 124-133 |  | + | 1 |
| **67** | QEPVLGPVRGPFP | 13 | Beta Casein | 2 | 696.89 | 1391.76 | 44.60 | 209-221 |  | + | 1 |
| **68** | QEPVLGPVRGPFPII | 15 | Beta Casein | 2 | 809.97 | 1617.92 | 48.82 | 209-223 |  | + | 1 |
| **69** | QEPVLGPVRGPFPIIV | 16 | Beta Casein | 2 | 859.50 | 1716.99 | 49.69 | 209-224 |  | + | 1 |
| **70** | QQQ | 3 | Beta Casein | 1 | 403.19 | 402.19 | 53.98 | 53-55 |  | + | 1 |
| **71** | QQQTEDELQDK | 11 | Beta Casein | 2 | 681.31 | 1360.61 | 22.23 | 53-63 |  | + | 1 |
| **72** | REL | 3 | Beta Casein | 1 | 417.25 | 416.24 | 60.22 | 16-18 |  | + | 1 |
| **73** | TEDELQDK | 8 | Beta Casein | 2 | 489.22 | 976.43 | 20.12 | 56-63 |  | + | 1 |
| **74** | VKEAMAPK | 8 | Beta Casein | 2 | 437.25 | 872.48 | 16.08 | 113-120 |  | + | 1 |
| **75** | VLGPVRGPFP | 10 | Beta Casein | 2 | 519.81 | 1037.60 | 43.37 | 212-221 |  | + | 1 |
| **76** | VLPVPQKAVPYPQ | 13 | Beta Casein | 2 | 718.42 | 1434.82 | 41.86 | 185-197 |  | + | 1 |
| **77** | YPFP | 4 | Beta Casein | 1 | 523.26 | 522.25 | 44.24 | 75-78 |  | + | 1 |
| **78** | YQEPVLGPVRGP | 12 | Beta Casein | 2 | 656.36 | 1310.70 | 41.75 | 208-219 |  | + | 1 |
| **79** | YQEPVLGPVRGPFP | 14 | Beta Casein | 2 | 778.42 | 1554.82 | 45.14 | 208-221 |  | + | 1 |
| **80** | YQEPVLGPVRGPFPIIV | 17 | Beta Casein | 2 | 941.04 | 1880.06 | 50.14 | 208-224 |  | + | 1 |
| **81** | ESPPEINTVQVTSTAV | 16 | Kappa Casein | 2 | 836.43 | 1670.84 | 44.04 | 175-190 |  | + | 1 |
| **82** | QWQ | 3 | Kappa Casein | 1 | 461.21 | 460.21 | 23.06 | 96-98 |  | + | 1 |
| **83** | SPPEINTVQVT | 11 | Kappa Casein | 2 | 592.81 | 1183.61 | 41.82 | 176-186 |  | + | 1 |
| **84** | SPPEINTVQVTSTAV | 15 | Kappa Casein | 2 | 771.90 | 1541.79 | 43.73 | 176-190 |  | + | 1 |

**Supplementary Table 6.** Milk peptides identified throughout the 28 days of storage period using the *S. thermophilus* 1K4.

|  | **Sequence** | **Length** | **Leading proteins** | **Charge** | **m/z** | **Mass** | **Retention time** | **Sequence** | **Day 1** | **Day 28** | **Incidence** |
| --- | --- | --- | --- | --- | --- | --- | --- | --- | --- | --- | --- |
| **1** | EEL | 3 | Beta Casein | 1 | 390.19 | 389.18 | 56.66 | 19-21 |  | + | 1 |
| **2** | QQQ | 3 | Beta Casein | 1 | 403.19 | 402.19 | 27.60 | 235-237 |  | + | 1 |
| **3** | TQSLVYPFPGPIPN | 14 | Beta Casein | 2 | 765.40 | 1528.79 | 43.85 | 70-83 |  | + | 1 |

**Supplementary Table 7.** Milk peptides identified throughout the 28 days of storage period using the *S. thermophilus* K16.

|  | **Sequence** | **Length** | **Leading proteins** | **Charge** | **m/z** | **Mass** | **Retention time** | **Sequence** | **Day 1** | **Day 28** | **Incidence** |
| --- | --- | --- | --- | --- | --- | --- | --- | --- | --- | --- | --- |
| **1** | FSDIPNPIGSENSEK | 15 | Alpha S1 Casein | 2 | 817.39 | 1632.76 | 41.70 | 194-208 | + | + | 2 |
| **2** | FVAPFPEVFGK | 11 | Alpha S1 Casein | 2 | 619.33 | 1236.65 | 47.10 | 39-49 | + | + | 2 |
| **3** | LEIVPNSAEERLHSM | 15 | Alpha S1 Casein | 3 | 575.63 | 1723.86 | 41.71 | 124-138 | + | + | 2 |
| **4** | PIGSENSEKTT | 11 | Alpha S1 Casein | 2 | 581.78 | 1161.55 | 17.62 | 200-210 | + | + | 2 |
| **5** | SEKTTMPLW | 9 | Alpha S1 Casein | 2 | 546.77 | 1091.53 | 43.63 | 206-214 | + | + | 2 |
| **6** | VAPFPEVFGKE | 11 | Alpha S1 Casein | 2 | 610.32 | 1218.63 | 44.77 | 40-50 | + | + | 2 |
| **7** | VAPFPEVFGKEKV | 13 | Alpha S1 Casein | 2 | 723.90 | 1445.79 | 43.01 | 40-52 | + | + | 2 |
| **8** | ENLLRF | 6 | Alpha S1 Casein | 2 | 396.22 | 790.43 | 43.65 | 33-38 |  | + | 1 |
| **9** | ENSEKTTMPLW | 11 | Alpha S1 Casein | 2 | 668.32 | 1334.62 | 43.98 | 204-214 |  | + | 1 |
| **10** | ERYLGYLEQ | 9 | Alpha S1 Casein | 2 | 585.79 | 1169.57 | 41.42 | 104-112 |  | + | 1 |
| **11** | FFV | 3 | Alpha S1 Casein | 1 | 412.22 | 411.22 | 51.78 | 38-40 |  | + | 1 |
| **12** | FSDIPNPIGSENSEKTT | 17 | Alpha S1 Casein | 2 | 918.44 | 1834.86 | 41.91 | 194-210 |  | + | 1 |
| **13** | FSDIPNPIGSENSEKTTMPLW | 21 | Alpha S1 Casein | 2 | 1190.06 | 2378.11 | 45.86 | 194-214 |  | + | 1 |
| **14** | GLPQEVLNENLLR | 13 | Alpha S1 Casein | 2 | 747.92 | 1493.82 | 45.16 | 25-37 |  | + | 1 |
| **15** | IGSENSEKTTMP | 12 | Alpha S1 Casein | 2 | 655.30 | 1308.59 | 20.46 | 201-212 |  | + | 1 |
| **16** | IGSENSEKTTMPLW | 14 | Alpha S1 Casein | 2 | 804.88 | 1607.75 | 41.73 | 201-214 |  | + | 1 |
| **17** | IKHQGLPQE | 9 | Alpha S1 Casein | 2 | 525.29 | 1048.57 | 20.96 | 21-29 |  | + | 1 |
| **18** | IPNPIGSENSEK | 12 | Alpha S1 Casein | 2 | 642.83 | 1283.64 | 32.67 | 197-208 |  | + | 1 |
| **19** | IPNPIGSENSEKTTMPLW | 18 | Alpha S1 Casein | 2 | 1007.50 | 2012.99 | 45.92 | 197-214 |  | + | 1 |
| **20** | IQKEDVPS | 8 | Alpha S1 Casein | 2 | 458.24 | 914.47 | 22.32 | 96-103 |  | + | 1 |
| **21** | IVPNSAEER | 9 | Alpha S1 Casein | 2 | 507.76 | 1013.51 | 21.49 | 126-134 |  | + | 1 |
| **22** | KEDVPSERY | 9 | Alpha S1 Casein | 2 | 561.77 | 1121.54 | 22.40 | 98-106 |  | + | 1 |
| **23** | KEDVPSERYLG | 11 | Alpha S1 Casein | 2 | 646.83 | 1291.64 | 30.03 | 98-108 |  | + | 1 |
| **24** | KEPMIGVNQEL | 11 | Alpha S1 Casein | 2 | 629.33 | 1256.64 | 41.52 | 147-157 |  | + | 1 |
| **25** | KYKVPQLE | 8 | Alpha S1 Casein | 2 | 502.79 | 1003.57 | 30.77 | 118-125 |  | + | 1 |
| **26** | NENLLRF | 7 | Alpha S1 Casein | 2 | 453.25 | 904.48 | 43.43 | 32-38 |  | + | 1 |
| **27** | PIGSENSEKTTMP | 13 | Alpha S1 Casein | 2 | 703.83 | 1405.64 | 22.63 | 200-212 |  | + | 1 |
| **28** | PIGSENSEKTTMPLW | 15 | Alpha S1 Casein | 2 | 845.41 | 1688.81 | 44.49 | 200-214 |  | + | 1 |
| **29** | QKEPMIGVNQEL | 12 | Alpha S1 Casein | 2 | 693.36 | 1384.70 | 41.53 | 146-157 |  | + | 1 |
| **30** | QKH | 3 | Alpha S1 Casein | 1 | 412.23 | 411.22 | 56.13 | 93-95 |  | + | 1 |
| **31** | RLK | 3 | Alpha S1 Casein | 1 | 416.30 | 415.29 | 51.84 | 115-117 |  | + | 1 |
| **32** | RQF | 3 | Alpha S1 Casein | 1 | 450.25 | 449.24 | 17.29 | 166-168 |  | + | 1 |
| **33** | SDIPNPIGSENSEKTTMP | 18 | Alpha S1 Casein | 2 | 958.95 | 1915.88 | 41.95 | 195-212 |  | + | 1 |
| **34** | VAPFPEVFGK | 10 | Alpha S1 Casein | 2 | 545.80 | 1089.59 | 44.81 | 40-49 |  | + | 1 |
| **35** | VAPFPEVFGKEK | 12 | Alpha S1 Casein | 2 | 674.37 | 1346.72 | 42.26 | 40-51 |  | + | 1 |
| **36** | VLNENLLR | 8 | Alpha S1 Casein | 2 | 485.79 | 969.56 | 38.66 | 30-37 |  | + | 1 |
| **37** | VLNENLLRF | 9 | Alpha S1 Casein | 2 | 559.32 | 1116.63 | 45.29 | 30-38 |  | + | 1 |
| **38** | YLE | 3 | Alpha S1 Casein | 1 | 424.21 | 423.20 | 54.37 | 109-111 |  | + | 1 |
| **39** | YTD | 3 | Alpha S1 Casein | 1 | 398.16 | 397.15 | 55.62 | 188-190 |  | + | 1 |
| **40** | YYVP | 4 | Alpha S1 Casein | 1 | 541.27 | 540.26 | 38.83 | 180-183 |  | + | 1 |
| **41** | GPIVLNPWDQVK | 12 | Alpha S2 Casein | 2 | 683.38 | 1364.75 | 44.93 | 117-128 | + | + | 2 |
| **42** | MESTEVFTKK | 10 | Alpha S2 Casein | 2 | 600.30 | 1198.59 | 26.73 | 156-165 | + | + | 2 |
| **43** | QGPIVLNPWDQVK | 13 | Alpha S2 Casein | 2 | 747.41 | 1492.80 | 44.93 | 116-128 | + | + | 2 |
| **44** | SIISQETYKQEK | 12 | Alpha S2 Casein | 2 | 727.38 | 1452.75 | 27.01 | 28-39 | + |  | 1 |
| **45** | TEEEKNRLNFL | 11 | Alpha S2 Casein | 2 | 696.86 | 1391.70 | 39.50 | 169-179 | + | + | 2 |
| **46** | ALPQYLKT | 8 | Alpha S2 Casein | 2 | 467.27 | 932.53 | 38.36 | 190-197 |  | + | 1 |
| **47** | DKH | 3 | Alpha S2 Casein | 1 | 399.20 | 398.19 | 59.75 | 90-92 |  | + | 1 |
| **48** | EEKNRLNFL | 9 | Alpha S2 Casein | 2 | 581.81 | 1161.61 | 39.42 | 171-179 |  | + | 1 |
| **49** | FALPQYLK | 8 | Alpha S2 Casein | 2 | 490.28 | 978.55 | 43.99 | 189-196 |  | + | 1 |
| **50** | FALPQYLKT | 9 | Alpha S2 Casein | 2 | 540.81 | 1079.60 | 44.25 | 189-197 |  | + | 1 |
| **51** | FALPQYLKTVYQHQK | 15 | Alpha S2 Casein | 3 | 622.01 | 1863.00 | 42.93 | 189-203 |  | + | 1 |
| **52** | IISQETYK | 8 | Alpha S2 Casein | 2 | 491.27 | 980.52 | 25.04 | 29-36 |  | + | 1 |
| **53** | IQPKTKVIP | 9 | Alpha S2 Casein | 2 | 512.33 | 1022.65 | 26.72 | 209-217 |  | + | 1 |
| **54** | IQPKTKVIPY | 10 | Alpha S2 Casein | 2 | 593.86 | 1185.71 | 31.76 | 209-218 |  | + | 1 |
| **55** | KFALPQYLKT | 10 | Alpha S2 Casein | 2 | 604.86 | 1207.70 | 42.11 | 188-197 |  | + | 1 |
| **56** | KISQRYQK | 8 | Alpha S2 Casein | 2 | 525.81 | 1049.60 | 13.94 | 181-188 |  | + | 1 |
| **57** | KKT | 3 | Alpha S2 Casein | 1 | 376.26 | 375.25 | 56.87 | 151-153 |  | + | 1 |
| **58** | LKK | 3 | Alpha S2 Casein | 1 | 388.29 | 387.28 | 61.52 | 116-118 |  | + | 1 |
| **59** | PWD | 3 | Alpha S2 Casein | 1 | 417.18 | 416.17 | 49.96 | 123-125 |  | + | 1 |
| **60** | PWIQPKTKVIPY | 12 | Alpha S2 Casein | 3 | 490.62 | 1468.84 | 41.81 | 207-218 |  | + | 1 |
| **61** | QKF | 3 | Alpha S2 Casein | 1 | 422.24 | 421.23 | 58.20 | 105-107 |  | + | 1 |
| **62** | QKFALPQYLKT | 11 | Alpha S2 Casein | 2 | 668.88 | 1335.76 | 41.97 | 187-197 |  | + | 1 |
| **63** | QKFPQYLQ | 8 | Alpha S2 Casein | 2 | 526.28 | 1050.55 | 37.80 | 105-112 |  | + | 1 |
| **64** | QKFPQYLQY | 9 | Alpha S2 Casein | 2 | 607.81 | 1213.61 | 42.37 | 105-113 |  | + | 1 |
| **65** | STF | 3 | Alpha S2 Casein | 1 | 354.17 | 353.16 | 56.82 | 52-54 |  | + | 1 |
| **66** | VFT | 3 | Alpha S2 Casein | 1 | 366.20 | 365.20 | 37.31 | 161-163 |  | + | 1 |
| **67** | VRN | 3 | Alpha S2 Casein | 1 | 388.23 | 387.22 | 59.45 | 59-61 |  | + | 1 |
| **68** | YQKFALPQ | 8 | Alpha S2 Casein | 2 | 497.77 | 993.53 | 39.11 | 186-193 |  | + | 1 |
| **69** | YQKFALPQYLKT | 12 | Alpha S2 Casein | 2 | 750.42 | 1498.82 | 42.55 | 186-197 |  | + | 1 |
| **70** | YVR | 3 | Alpha S2 Casein | 1 | 437.25 | 436.24 | 57.63 | 218-220 |  | + | 1 |
| **71** | YVRYL | 5 | Alpha S2 Casein | 2 | 357.20 | 712.39 | 33.19 | 218-222 |  | + | 1 |
| **72** | EEQQQTEDELQDK | 13 | Beta Casein | 2 | 810.36 | 1618.70 | 24.55 | 51-63 | + |  | 1 |
| **73** | EPVLGPVRGPFP | 12 | Beta Casein | 2 | 632.86 | 1263.70 | 44.61 | 210-221 | + | + | 2 |
| **74** | EPVLGPVRGPFPI | 13 | Beta Casein | 2 | 689.40 | 1376.78 | 46.95 | 210-222 | + | + | 2 |
| **75** | EQQQTEDELQDK | 12 | Beta Casein | 2 | 745.83 | 1489.65 | 23.15 | 52-63 | + | + | 2 |
| **76** | LGPVRGPFPIIV | 12 | Beta Casein | 2 | 632.89 | 1263.77 | 48.15 | 213-224 | + | + | 2 |
| **77** | QQTEDELQDK | 10 | Beta Casein | 2 | 617.28 | 1232.55 | 21.77 | 54-63 | + | + | 2 |
| **78** | TEDELQDKIHP | 11 | Beta Casein | 2 | 662.82 | 1323.63 | 32.33 | 56-66 | + |  | 1 |
| **79** | TEDELQDKIHPF | 12 | Beta Casein | 2 | 736.36 | 1470.70 | 41.26 | 56-67 | + | + | 2 |
| **80** | VLGPVRGPFPIIV | 13 | Beta Casein | 2 | 682.43 | 1362.84 | 48.63 | 212-224 | + | + | 2 |
| **81** | YQEPVLGPVRGPFPI | 15 | Beta Casein | 2 | 834.96 | 1667.90 | 47.25 | 208-222 | + | + | 2 |
| **82** | AVPYPQRD | 8 | Beta Casein | 2 | 473.24 | 944.47 | 25.98 | 192-199 |  | + | 1 |
| **83** | AVPYPQRDMP | 10 | Beta Casein | 2 | 587.29 | 1172.56 | 35.61 | 192-201 |  | + | 1 |
| **84** | AVPYPQRDMPIQ | 12 | Beta Casein | 2 | 707.86 | 1413.71 | 39.60 | 192-203 |  | + | 1 |
| **85** | AVPYPQRDMPIQA | 13 | Beta Casein | 2 | 743.38 | 1484.74 | 41.28 | 192-204 |  | + | 1 |
| **86** | DELQDKIHPF | 10 | Beta Casein | 2 | 621.31 | 1240.61 | 41.69 | 58-67 |  | + | 1 |
| **87** | DKIHPF | 6 | Beta Casein | 2 | 378.71 | 755.40 | 28.74 | 62-67 |  | + | 1 |
| **88** | DKIHPFAQTQ | 10 | Beta Casein | 2 | 592.81 | 1183.60 | 28.07 | 62-71 |  | + | 1 |
| **89** | DKIHPFAQTQS | 11 | Beta Casein | 2 | 636.32 | 1270.63 | 27.87 | 62-72 |  | + | 1 |
| **90** | DKIHPFAQTQSL | 12 | Beta Casein | 2 | 692.86 | 1383.71 | 37.42 | 62-73 |  | + | 1 |
| **91** | DVENLHLPLPL | 11 | Beta Casein | 2 | 630.35 | 1258.69 | 50.54 | 144-154 |  | + | 1 |
| **92** | DVENLHLPLPLLQ | 13 | Beta Casein | 2 | 750.92 | 1499.83 | 51.74 | 144-156 |  | + | 1 |
| **93** | EDELQDKIHPF | 11 | Beta Casein | 2 | 685.83 | 1369.65 | 41.51 | 57-67 |  | + | 1 |
| **94** | EDELQDKIHPFA | 12 | Beta Casein | 2 | 721.35 | 1440.69 | 41.41 | 57-68 |  | + | 1 |
| **95** | EDELQDKIHPFAQTQ | 15 | Beta Casein | 2 | 899.93 | 1797.85 | 40.61 | 57-71 |  | + | 1 |
| **96** | EDELQDKIHPFAQTQS | 16 | Beta Casein | 2 | 943.45 | 1884.89 | 40.34 | 57-72 |  | + | 1 |
| **97** | EEL | 3 | Beta Casein | 1 | 390.19 | 389.18 | 49.73 | 19-21 |  | + | 1 |
| **98** | ELQDKIHPF | 9 | Beta Casein | 2 | 563.80 | 1125.58 | 37.52 | 59-67 |  | + | 1 |
| **99** | ELQDKIHPFAQ | 11 | Beta Casein | 2 | 663.35 | 1324.68 | 36.47 | 59-69 |  | + | 1 |
| **100** | ELQDKIHPFAQT | 12 | Beta Casein | 2 | 713.87 | 1425.73 | 36.92 | 59-70 |  | + | 1 |
| **101** | ELQDKIHPFAQTQ | 13 | Beta Casein | 2 | 777.90 | 1553.78 | 36.20 | 59-71 |  | + | 1 |
| **102** | ELQDKIHPFAQTQS | 14 | Beta Casein | 2 | 821.42 | 1640.82 | 35.94 | 59-72 |  | + | 1 |
| **103** | EPVLGPVRGPFPII | 14 | Beta Casein | 2 | 745.94 | 1489.87 | 48.94 | 210-223 |  | + | 1 |
| **104** | FPKYPVEP | 8 | Beta Casein | 2 | 488.76 | 975.51 | 40.04 | 126-133 |  | + | 1 |
| **105** | FPKYPVEPFTESQS | 14 | Beta Casein | 2 | 828.40 | 1654.79 | 43.48 | 126-139 |  | + | 1 |
| **106** | FQSEEQQQTEDELQDK | 16 | Beta Casein | 2 | 991.43 | 1980.85 | 31.45 | 48-63 |  | + | 1 |
| **107** | GPVRGPFPIIV | 11 | Beta Casein | 2 | 576.35 | 1150.69 | 46.77 | 214-224 |  | + | 1 |
| **108** | HKEMPFPKYPVEPF | 14 | Beta Casein | 3 | 582.63 | 1744.86 | 64.05 | 121-134 |  | + | 1 |
| **109** | IHPFAQTQ | 8 | Beta Casein | 2 | 471.25 | 940.48 | 29.26 | 64-71 |  | + | 1 |
| **110** | IHPFAQTQS | 9 | Beta Casein | 2 | 514.76 | 1027.51 | 28.94 | 64-72 |  | + | 1 |
| **111** | ITRINK | 6 | Beta Casein | 2 | 372.74 | 743.47 | 14.84 | 38-43 |  | + | 1 |
| **112** | KAVPYPQRD | 9 | Beta Casein | 2 | 537.29 | 1072.57 | 20.84 | 191-199 |  | + | 1 |
| **113** | KFQ | 3 | Beta Casein | 1 | 422.24 | 421.23 | 60.41 | 47-49 |  | + | 1 |
| **114** | LGPVRGPFPI | 10 | Beta Casein | 2 | 526.82 | 1051.62 | 45.18 | 213-222 |  | + | 1 |
| **115** | LHL | 3 | Beta Casein | 1 | 382.24 | 381.24 | 57.44 | 148-150 |  | + | 1 |
| **116** | LSSSEESITR | 10 | Beta Casein | 2 | 554.78 | 1107.54 | 25.14 | 31-40 |  | + | 1 |
| **117** | LYQEPVLGPVRGPFPI | 16 | Beta Casein | 2 | 891.50 | 1780.99 | 48.01 | 207-222 |  | + | 1 |
| **118** | LYQEPVLGPVRGPFPIIV | 18 | Beta Casein | 2 | 997.58 | 1993.14 | 50.50 | 207-224 |  | + | 1 |
| **119** | NLHLPLPL | 8 | Beta Casein | 2 | 458.78 | 915.55 | 47.74 | 147-154 |  | + | 1 |
| **120** | NLHLPLPLLQ | 10 | Beta Casein | 2 | 579.36 | 1156.70 | 49.39 | 147-156 |  | + | 1 |
| **121** | NVPGEIVESLSSSEESITR | 19 | Beta Casein | 2 | 1017.01 | 2032.00 | 45.55 | 22-40 |  | + | 1 |
| **122** | QEPVLGPVRGPFPI | 14 | Beta Casein | 2 | 753.43 | 1504.84 | 46.76 | 209-222 |  | + | 1 |
| **123** | QEPVLGPVRGPFPII | 15 | Beta Casein | 2 | 809.97 | 1617.92 | 48.61 | 209-223 |  | + | 1 |
| **124** | QEPVLGPVRGPFPIIV | 16 | Beta Casein | 2 | 859.50 | 1716.99 | 49.49 | 209-224 |  | + | 1 |
| **125** | QQQ | 3 | Beta Casein | 1 | 403.19 | 402.19 | 63.19 | 53-55 |  | + | 1 |
| **126** | QQQTEDELQDK | 11 | Beta Casein | 2 | 681.31 | 1360.61 | 22.18 | 53-63 |  | + | 1 |
| **127** | QQTEDELQD | 9 | Beta Casein | 2 | 553.24 | 1104.46 | 27.50 | 54-62 |  | + | 1 |
| **128** | RDMPIQAF | 8 | Beta Casein | 2 | 489.25 | 976.48 | 42.38 | 198-205 |  | + | 1 |
| **129** | RDMPIQAFL | 9 | Beta Casein | 2 | 545.79 | 1089.56 | 46.36 | 198-206 |  | + | 1 |
| **130** | RELEELN | 7 | Beta Casein | 2 | 451.73 | 901.45 | 28.14 | 16-22 |  | + | 1 |
| **131** | SLPQNIPPLTQTPV | 14 | Beta Casein | 2 | 752.92 | 1503.83 | 46.23 | 84-97 |  | + | 1 |
| **132** | SLSQSKVLPVPQ | 12 | Beta Casein | 2 | 641.87 | 1281.73 | 39.97 | 179-190 |  | + | 1 |
| **133** | SSSEESITRIN | 11 | Beta Casein | 2 | 611.80 | 1221.58 | 29.44 | 32-42 |  | + | 1 |
| **134** | VLGPVRGPFP | 10 | Beta Casein | 2 | 519.81 | 1037.60 | 43.27 | 212-221 |  | + | 1 |
| **135** | VLGPVRGPFPI | 11 | Beta Casein | 2 | 576.35 | 1150.69 | 45.69 | 212-222 |  | + | 1 |
| **136** | VLPVPQKAVPYPQ | 13 | Beta Casein | 2 | 718.42 | 1434.82 | 41.77 | 185-197 |  | + | 1 |
| **137** | YQEPVLGPVRGPFPIIV | 17 | Beta Casein | 2 | 941.04 | 1880.06 | 49.90 | 208-224 |  | + | 1 |
| **138** | MKGLDIQK | 8 | Beta Lactoglobulin | 2 | 466.77 | 931.52 | 27.10 | 23-30 |  | + | 1 |
| **139** | YKK | 3 | Beta Lactoglobulin | 1 | 438.27 | 437.26 | 59.64 | 115-117 |  | + | 1 |
| **140** | YLL | 3 | Beta Lactoglobulin | 1 | 408.25 | 407.24 | 39.98 | 118-120 |  | + | 1 |
| **141** | ESPPEINTVQVTSTAV | 16 | Kappa Casein | 2 | 836.43 | 1670.84 | 43.99 | 175-190 |  | + | 1 |
| **142** | FFL | 3 | Kappa Casein | 1 | 426.24 | 425.23 | 58.14 | 5-7 |  | + | 1 |
| **143** | FLPYPYYAKPA | 11 | Kappa Casein | 2 | 665.35 | 1328.68 | 43.30 | 76-86 |  | + | 1 |
| **144** | FLPYPYYAKPAA | 12 | Kappa Casein | 2 | 700.87 | 1399.72 | 43.30 | 76-87 |  | + | 1 |
| **145** | IAKYIPIQY | 9 | Kappa Casein | 2 | 554.82 | 1107.63 | 42.72 | 43-51 |  | + | 1 |
| **146** | KTEIPTIN | 8 | Kappa Casein | 2 | 458.26 | 914.51 | 34.75 | 137-144 |  | + | 1 |
| **147** | KYIPIQY | 7 | Kappa Casein | 2 | 462.76 | 923.51 | 39.63 | 45-51 |  | + | 1 |
| **148** | LSRYPSYGLN | 10 | Kappa Casein | 2 | 585.30 | 1168.59 | 36.04 | 53-62 |  | + | 1 |
| **149** | QFLPYPYYAKPA | 12 | Kappa Casein | 2 | 729.38 | 1456.74 | 43.99 | 75-86 |  | + | 1 |
| **150** | QQK | 3 | Kappa Casein | 1 | 403.23 | 402.22 | 58.78 | 145-147 |  | + | 1 |
| **151** | SPPEINTVQVTSTAV | 15 | Kappa Casein | 2 | 771.90 | 1541.79 | 43.69 | 176-190 |  | + | 1 |
| **152** | VRS | 3 | Kappa Casein | 1 | 361.22 | 360.21 | 63.30 | 88-90 |  | + | 1 |
| **153** | YVL | 3 | Kappa Casein | 1 | 394.23 | 393.23 | 49.88 | 51-53 |  | + | 1 |
| **154** | YYQQKPVALINN | 12 | Kappa Casein | 2 | 725.89 | 1449.76 | 37.47 | 63-74 |  | + | 1 |
| **155** | LLFSSAYSR | 9 | Serum Albumin | 2 | 522.28 | 1042.54 | 13.32 | 11-19 | + |  | 1 |
| **156** | EEC | 3 | Serum Albumin | 1 | 437.13 | 436.13 | 48.92 | 381-383 |  | + | 1 |
| **157** | GLVLIAFSQYLQQCPFDEHVK | 21 | Serum Albumin | 3 | 831.43 | 2491.26 | 49.61 | 45-65 |  | + | 1 |
| **158** | KCC | 3 | Serum Albumin | 1 | 467.17 | 466.17 | 48.04 | 498-500 |  | + | 1 |
| **159** | KFG | 3 | Serum Albumin | 1 | 351.20 | 350.20 | 53.07 | 228-230 |  | + | 1 |
| **160** | LGEYGFQNALIVR | 13 | Serum Albumin | 2 | 740.40 | 1478.79 | 44.90 | 421-433 |  | + | 1 |
| **161** | LLK | 3 | Serum Albumin | 1 | 373.28 | 372.27 | 60.26 | 555-557 |  | + | 1 |
| **162** | LLL | 3 | Serum Albumin | 1 | 358.27 | 357.26 | 65.43 | 9-11 |  | + | 1 |
| **163** | LVNELTEFAK | 10 | Serum Albumin | 2 | 582.32 | 1162.62 | 43.01 | 66-75 |  | + | 1 |
| **164** | PFD | 3 | Serum Albumin | 1 | 378.17 | 377.16 | 46.39 | 59-61 |  | + | 1 |
| **165** | QEC | 3 | Serum Albumin | 1 | 436.15 | 435.14 | 46.08 | 189-191 |  | + | 1 |
| **166** | RCA | 3 | Serum Albumin | 1 | 406.19 | 405.18 | 65.80 | 222-224 |  | + | 1 |
| **167** | RLA | 3 | Serum Albumin | 1 | 359.24 | 358.23 | 63.52 | 371-373 |  | + | 1 |
| **168** | RLC | 3 | Serum Albumin | 1 | 448.23 | 447.23 | 17.32 | 482-484 |  | + | 1 |
| **169** | RLR | 3 | Serum Albumin | 1 | 444.30 | 443.30 | 61.07 | 220-222 |  | + | 1 |
| **170** | SRS | 3 | Serum Albumin | 1 | 349.18 | 348.18 | 63.56 | 450-452 |  | + | 1 |
| **171** | THK | 3 | Serum Albumin | 1 | 385.22 | 384.21 | 58.35 | 26-28 |  | + | 1 |
| **172** | TRC | 3 | Serum Albumin | 1 | 436.20 | 435.19 | 42.61 | 458-460 |  | + | 1 |
| **173** | TVMENFVAFVDK | 12 | Serum Albumin | 2 | 700.35 | 1398.69 | 47.23 | 569-580 |  | + | 1 |
| **174** | VLH | 3 | Serum Albumin | 1 | 368.23 | 367.22 | 63.33 | 485-487 |  | + | 1 |
| **175** | VSR | 3 | Serum Albumin | 1 | 361.22 | 360.21 | 58.17 | 449-451 |  | + | 1 |
| **176** | YSR | 3 | Serum Albumin | 1 | 425.21 | 424.21 | 63.16 | 17-19 |  | + | 1 |

**Supplementary Table 8.** Milk peptides identified throughout the 28 days of storage period using the *Lc. lactis* NCCB 100539.

|  | **Sequence** | **Length** | **Leading proteins** | **Charge** | **m/z** | **Mass** | **Retention time** | **Sequence** | **Day 1** | **Day 2** | **Incidence** |
| --- | --- | --- | --- | --- | --- | --- | --- | --- | --- | --- | --- |
| **1** | FFVAP | 5 | Alpha S1 Casein | 1 | 580.31 | 579.31 | 52.16 | 38-42 | + | + | 2 |
| **2** | LKK | 3 | Alpha S1 Casein | 1 | 388.29 | 387.28 | 61.20 | 116-118 | + |  | 1 |
| **3** | LRL | 3 | Alpha S1 Casein | 1 | 401.29 | 400.28 | 58.00 | 679-681 | + |  | 1 |
| **4** | QLE | 3 | Alpha S1 Casein | 1 | 389.20 | 388.20 | 61.34 | 234-236 | + |  | 1 |
| **5** | QQK | 3 | Alpha S1 Casein | 1 | 403.23 | 402.22 | 57.54 | 922-924 | + |  | 1 |
| **6** | RQF | 3 | Alpha S1 Casein | 1 | 450.25 | 449.24 | 11.18 | 32-34 | + |  | 1 |
| **7** | YLE | 3 | Alpha S1 Casein | 1 | 424.21 | 423.20 | 53.87 | 109-111 | + |  | 1 |
| **8** | APSFSDIPNPIGSENSEK | 18 | Alpha S1 Casein | 2 | 944.95 | 1887.89 | 43.56 | 191-208 |  | + | 1 |
| **9** | APSFSDIPNPIGSENSEKT | 19 | Alpha S1 Casein | 2 | 995.47 | 1988.93 | 43.59 | 191-209 |  | + | 1 |
| **10** | APSFSDIPNPIGSENSEKTTMP | 22 | Alpha S1 Casein | 2 | 1160.04 | 2318.07 | 44.57 | 191-212 |  | + | 1 |
| **11** | APSFSDIPNPIGSENSEKTTMPLW | 24 | Alpha S1 Casein | 2 | 1309.63 | 2617.24 | 48.53 | 191-214 |  | + | 1 |
| **12** | DAPSFSDIPNPIGSENSEKTTMPLW | 25 | Alpha S1 Casein | 2 | 1367.14 | 2732.26 | 49.91 | 190-214 |  | + | 1 |
| **13** | DIPNPIGSENSEKTTMP | 17 | Alpha S1 Casein | 2 | 915.43 | 1828.85 | 42.73 | 196-212 |  | + | 1 |
| **14** | DIPNPIGSENSEKTTMPL | 18 | Alpha S1 Casein | 2 | 971.97 | 1941.94 | 44.81 | 196-213 |  | + | 1 |
| **15** | DIPNPIGSENSEKTTMPLW | 19 | Alpha S1 Casein | 2 | 1065.01 | 2128.01 | 47.85 | 196-214 |  | + | 1 |
| **16** | EVLNENLLR | 9 | Alpha S1 Casein | 2 | 550.31 | 1098.60 | 41.87 | 29-37 |  | + | 1 |
| **17** | EVLNENLLRF | 10 | Alpha S1 Casein | 2 | 623.84 | 1245.67 | 46.29 | 29-38 |  | + | 1 |
| **18** | FFVAPFPEVFGK | 12 | Alpha S1 Casein | 2 | 692.87 | 1383.72 | 49.78 | 38-49 |  | + | 1 |
| **19** | FFVAPFPEVFGKEK | 14 | Alpha S1 Casein | 2 | 821.44 | 1640.86 | 46.50 | 38-51 |  | + | 1 |
| **20** | FFVAPFPEVFGKEKV | 15 | Alpha S1 Casein | 2 | 870.97 | 1739.93 | 46.99 | 38-52 |  | + | 1 |
| **21** | FSDIPNPIGSEN | 12 | Alpha S1 Casein | 2 | 645.30 | 1288.59 | 44.50 | 194-205 |  | + | 1 |
| **22** | FSDIPNPIGSENSEK | 15 | Alpha S1 Casein | 2 | 817.39 | 1632.76 | 41.78 | 194-208 |  | + | 1 |
| **23** | FSDIPNPIGSENSEKT | 16 | Alpha S1 Casein | 2 | 867.91 | 1733.81 | 41.87 | 194-209 |  | + | 1 |
| **24** | FSDIPNPIGSENSEKTT | 17 | Alpha S1 Casein | 2 | 918.44 | 1834.86 | 41.90 | 194-210 |  | + | 1 |
| **25** | FSDIPNPIGSENSEKTTMP | 19 | Alpha S1 Casein | 2 | 1040.48 | 2078.95 | 42.12 | 194-212 |  | + | 1 |
| **26** | FSDIPNPIGSENSEKTTMPL | 20 | Alpha S1 Casein | 2 | 1089.03 | 2176.04 | 45.00 | 194-213 |  | + | 1 |
| **27** | FSDIPNPIGSENSEKTTMPLW | 21 | Alpha S1 Casein | 2 | 1190.06 | 2378.11 | 45.92 | 194-214 |  | + | 1 |
| **28** | FVAPFPEVFGK | 11 | Alpha S1 Casein | 2 | 619.33 | 1236.65 | 47.26 | 39-49 |  | + | 1 |
| **29** | FVAPFPEVFGKE | 12 | Alpha S1 Casein | 2 | 683.86 | 1365.70 | 47.34 | 39-50 |  | + | 1 |
| **30** | FVAPFPEVFGKEK | 13 | Alpha S1 Casein | 2 | 747.90 | 1493.79 | 44.49 | 39-51 |  | + | 1 |
| **31** | FVAPFPEVFGKEKV | 14 | Alpha S1 Casein | 3 | 531.96 | 1592.86 | 45.04 | 39-52 |  | + | 1 |
| **32** | IPNPIGSENSEKTTMPLW | 18 | Alpha S1 Casein | 2 | 1007.50 | 2012.99 | 45.96 | 197-214 |  | + | 1 |
| **33** | NPIGSENSEKTTMPLW | 16 | Alpha S1 Casein | 2 | 902.43 | 1802.85 | 44.54 | 199-214 |  | + | 1 |
| **34** | PSFSDIPNPIGSENSEKTTMPLW | 23 | Alpha S1 Casein | 2 | 1274.11 | 2546.20 | 48.44 | 192-214 |  | + | 1 |
| **35** | QEVLNENLL | 9 | Alpha S1 Casein | 2 | 536.29 | 1070.56 | 45.86 | 28-36 |  | + | 1 |
| **36** | QEVLNENLLR | 10 | Alpha S1 Casein | 2 | 614.34 | 1226.66 | 42.03 | 28-37 |  | + | 1 |
| **37** | QEVLNENLLRF | 11 | Alpha S1 Casein | 2 | 687.87 | 1373.73 | 46.16 | 28-38 |  | + | 1 |
| **38** | QGLPQEVLNENLL | 13 | Alpha S1 Casein | 2 | 733.90 | 1465.78 | 48.90 | 24-36 |  | + | 1 |
| **39** | QGLPQEVLNENLLR | 14 | Alpha S1 Casein | 2 | 811.95 | 1621.88 | 45.05 | 24-37 |  | + | 1 |
| **40** | QGLPQEVLNENLLRF | 15 | Alpha S1 Casein | 2 | 885.48 | 1768.95 | 48.74 | 24-38 |  | + | 1 |
| **41** | QGLPQEVLNENLLRFF | 16 | Alpha S1 Casein | 2 | 959.02 | 1916.02 | 52.45 | 24-39 |  | + | 1 |
| **42** | QYTDAPSFSDIPNPIGSENSEKT | 23 | Alpha S1 Casein | 2 | 1249.07 | 2496.13 | 47.47 | 187-209 |  | + | 1 |
| **43** | SDIPNPIGSENSEK | 14 | Alpha S1 Casein | 2 | 743.85 | 1485.69 | 36.61 | 195-208 |  | + | 1 |
| **44** | SDIPNPIGSENSEKTTMP | 18 | Alpha S1 Casein | 2 | 958.95 | 1915.88 | 41.94 | 195-212 |  | + | 1 |
| **45** | SDIPNPIGSENSEKTTMPLW | 20 | Alpha S1 Casein | 2 | 1108.53 | 2215.05 | 46.57 | 195-214 |  | + | 1 |
| **46** | TDAPSFSDIPNPIGSENSEKTTMPLW | 26 | Alpha S1 Casein | 2 | 1417.66 | 2833.31 | 49.04 | 189-214 |  | + | 1 |
| **47** | VAPFPEVFGK | 10 | Alpha S1 Casein | 2 | 545.80 | 1089.59 | 44.84 | 40-49 |  | + | 1 |
| **48** | VAPFPEVFGKEK | 12 | Alpha S1 Casein | 2 | 674.37 | 1346.72 | 42.28 | 40-51 |  | + | 1 |
| **49** | VAPFPEVFGKEKV | 13 | Alpha S1 Casein | 2 | 723.90 | 1445.79 | 43.15 | 40-52 |  | + | 1 |
| **50** | YTDAPSFSDIPNPIGSENSEKTTMP | 25 | Alpha S1 Casein | 2 | 1349.61 | 2697.21 | 45.22 | 188-212 |  | + | 1 |
| **51** | YTDAPSFSDIPNPIGSENSEKTTMPLW | 27 | Alpha S1 Casein | 2 | 1499.19 | 2996.37 | 49.15 | 188-214 |  | + | 1 |
| **52** | FALPQYLK | 8 | Alpha S2 Casein | 2 | 490.28 | 978.55 | 44.00 | 189-196 |  | + | 1 |
| **53** | FALPQYLKT | 9 | Alpha S2 Casein | 2 | 540.81 | 1079.60 | 44.28 | 189-197 |  | + | 1 |
| **54** | FALPQYLKTVYQH | 13 | Alpha S2 Casein | 2 | 804.43 | 1606.85 | 44.50 | 189-201 |  | + | 1 |
| **55** | FALPQYLKTVYQHQ | 14 | Alpha S2 Casein | 3 | 579.31 | 1734.91 | 44.44 | 189-202 |  | + | 1 |
| **56** | NAVPITPTLN | 10 | Alpha S2 Casein | 2 | 520.29 | 1038.57 | 43.47 | 130-139 |  | + | 1 |
| **57** | NAVPITPTLNR | 11 | Alpha S2 Casein | 2 | 598.34 | 1194.67 | 38.57 | 130-140 |  | + | 1 |
| **58** | QKFALPQYL | 9 | Alpha S2 Casein | 2 | 554.31 | 1106.61 | 44.80 | 187-195 |  | + | 1 |
| **59** | VIPYVRYL | 8 | Alpha S2 Casein | 2 | 511.81 | 1021.60 | 44.82 | 215-222 |  | + | 1 |
| **60** | YQGPIVLNPWDQVK | 14 | Alpha S2 Casein | 2 | 828.94 | 1655.87 | 45.79 | 115-128 |  | + | 1 |
| **61** | YQGPIVLNPWDQVKR | 15 | Alpha S2 Casein | 3 | 605.00 | 1811.97 | 43.77 | 115-129 |  | + | 1 |
| **62** | YQKFALPQY | 9 | Alpha S2 Casein | 2 | 579.30 | 1156.59 | 42.71 | 186-194 |  | + | 1 |
| **63** | YQKFALPQYL | 10 | Alpha S2 Casein | 2 | 635.85 | 1269.68 | 45.31 | 186-195 |  | + | 1 |
| **64** | YQKFALPQYLK | 11 | Alpha S2 Casein | 2 | 699.89 | 1397.77 | 42.26 | 186-196 |  | + | 1 |
| **65** | YQKFALPQYLKT | 12 | Alpha S2 Casein | 2 | 750.42 | 1498.82 | 42.58 | 186-197 |  | + | 1 |
| **66** | MKV | 3 | Beta Casein | 1 | 377.22 | 376.21 | 43.57 | 98-100 | + |  | 1 |
| **67** | QQQ | 3 | Beta Casein | 1 | 403.19 | 402.19 | 51.21 | 53-55 | + |  | 1 |
| **68** | SEEQQQTEDELQDK | 14 | Beta Casein | 2 | 853.87 | 1705.73 | 26.11 | 50-63 | + |  | 1 |
| **69** | AVPYPQRDMPIQA | 13 | Beta Casein | 2 | 743.38 | 1484.74 | 41.31 | 192-204 |  | + | 1 |
| **70** | AVPYPQRDMPIQAF | 14 | Beta Casein | 2 | 816.91 | 1631.81 | 44.40 | 192-205 |  | + | 1 |
| **71** | AVPYPQRDMPIQAFL | 15 | Beta Casein | 2 | 873.46 | 1744.90 | 47.65 | 192-206 |  | + | 1 |
| **72** | DKIHPFAQTQ | 10 | Beta Casein | 2 | 592.81 | 1183.60 | 28.18 | 62-71 |  | + | 1 |
| **73** | DKIHPFAQTQS | 11 | Beta Casein | 2 | 636.32 | 1270.63 | 27.91 | 62-72 |  | + | 1 |
| **74** | DKIHPFAQTQSL | 12 | Beta Casein | 2 | 692.86 | 1383.71 | 37.61 | 62-73 |  | + | 1 |
| **75** | DVENLHLPLPL | 11 | Beta Casein | 2 | 630.35 | 1258.69 | 50.63 | 144-154 |  | + | 1 |
| **76** | DVENLHLPLPLLQ | 13 | Beta Casein | 2 | 750.92 | 1499.83 | 51.88 | 144-156 |  | + | 1 |
| **77** | ELQDKIHPF | 9 | Beta Casein | 2 | 563.80 | 1125.58 | 37.64 | 59-67 |  | + | 1 |
| **78** | EPVLGPVRGPFP | 12 | Beta Casein | 2 | 632.86 | 1263.70 | 44.72 | 210-221 |  | + | 1 |
| **79** | EPVLGPVRGPFPI | 13 | Beta Casein | 2 | 689.40 | 1376.78 | 47.13 | 210-222 |  | + | 1 |
| **80** | EPVLGPVRGPFPII | 14 | Beta Casein | 2 | 745.94 | 1489.87 | 49.09 | 210-223 |  | + | 1 |
| **81** | EPVLGPVRGPFPIIV | 15 | Beta Casein | 2 | 795.47 | 1588.93 | 49.95 | 210-224 |  | + | 1 |
| **82** | IHPFAQTQS | 9 | Beta Casein | 2 | 514.76 | 1027.51 | 28.93 | 64-72 |  | + | 1 |
| **83** | KAVPYPQRDMPIQAFL | 16 | Beta Casein | 3 | 625.34 | 1872.99 | 44.90 | 191-206 |  | + | 1 |
| **84** | LLYQEPVLGPVRGPFPIIV | 19 | Beta Casein | 2 | 1054.12 | 2106.22 | 51.64 | 206-224 |  | + | 1 |
| **85** | LYQEPVLGPVRGPFP | 15 | Beta Casein | 2 | 834.96 | 1667.90 | 45.75 | 207-221 |  | + | 1 |
| **86** | LYQEPVLGPVRGPFPI | 16 | Beta Casein | 2 | 891.50 | 1780.99 | 48.09 | 207-222 |  | + | 1 |
| **87** | LYQEPVLGPVRGPFPII | 17 | Beta Casein | 2 | 948.04 | 1894.07 | 49.85 | 207-223 |  | + | 1 |
| **88** | LYQEPVLGPVRGPFPIIV | 18 | Beta Casein | 2 | 997.58 | 1993.14 | 50.63 | 207-224 |  | + | 1 |
| **89** | NLHLPLPLLQ | 10 | Beta Casein | 2 | 579.36 | 1156.70 | 49.49 | 147-156 |  | + | 1 |
| **90** | NVPGEIVESLSSSEESITR | 19 | Beta Casein | 2 | 1017.01 | 2032.00 | 45.61 | 22-40 |  | + | 1 |
| **91** | NVPGEIVESLSSSEESITRIN | 21 | Beta Casein | 2 | 1130.57 | 2259.12 | 47.14 | 22-42 |  | + | 1 |
| **92** | NVPGEIVESLSSSEESITRINK | 22 | Beta Casein | 3 | 796.75 | 2387.22 | 45.34 | 22-43 |  | + | 1 |
| **93** | QEPVLGPVRGPFP | 13 | Beta Casein | 2 | 696.89 | 1391.76 | 44.56 | 209-221 |  | + | 1 |
| **94** | QEPVLGPVRGPFPI | 14 | Beta Casein | 2 | 753.43 | 1504.84 | 46.86 | 209-222 |  | + | 1 |
| **95** | QEPVLGPVRGPFPII | 15 | Beta Casein | 2 | 809.97 | 1617.92 | 48.72 | 209-223 |  | + | 1 |
| **96** | QEPVLGPVRGPFPIIV | 16 | Beta Casein | 2 | 859.50 | 1716.99 | 49.61 | 209-224 |  | + | 1 |
| **97** | VLGPVRGPFPIIV | 13 | Beta Casein | 2 | 682.43 | 1362.84 | 48.82 | 212-224 |  | + | 1 |
| **98** | VLPVPQKAVPYPQ | 13 | Beta Casein | 2 | 718.42 | 1434.82 | 41.80 | 185-197 |  | + | 1 |
| **99** | VPPF | 4 | Beta Casein | 1 | 459.26 | 458.25 | 42.49 | 99-102 |  | + | 1 |
| **100** | YQEPVLGPVRGPFP | 14 | Beta Casein | 2 | 778.42 | 1554.82 | 45.05 | 208-221 |  | + | 1 |
| **101** | YQEPVLGPVRGPFPI | 15 | Beta Casein | 2 | 834.96 | 1667.90 | 47.39 | 208-222 |  | + | 1 |
| **102** | YQEPVLGPVRGPFPII | 16 | Beta Casein | 2 | 891.50 | 1780.99 | 49.21 | 208-223 |  | + | 1 |
| **103** | YQEPVLGPVRGPFPIIV | 17 | Beta Casein | 2 | 941.04 | 1880.06 | 50.05 | 208-224 |  | + | 1 |
| **104** | PEVIESPPEINTVQVTSTAV | 20 | Kappa Casein | 2 | 1055.55 | 2109.08 | 46.01 | 171-190 |  | + | 1 |
| **105** | SPPEINTVQVTSTAV | 15 | Kappa Casein | 2 | 771.90 | 1541.79 | 43.69 | 176-190 |  | + | 1 |
| **106** | LGEYGFQNALIVR | 13 | Serum Albumin | 2 | 740.40 | 1478.79 | 44.92 | 421-433 |  | + | 1 |
| **107** | LRE | 3 | Serum Albumin | 1 | 417.25 | 416.24 | 25.66 | 1629-1631 |  | + | 1 |
| **108** | VLH | 3 | Serum Albumin | 1 | 368.23 | 367.22 | 54.22 | 1647-1649 |  | + | 1 |

**Supplementary Table 9.** Milk peptides identified throughout the 28 days of storage period using the *Lb. bulgaricus* 23.

|  | **Sequence** | **Length** | **Leading proteins** | **Charge** | **m/z** | **Mass** | **Retention time** | **Sequence** | **Day 1** | **Day 28** | **Incidence** |
| --- | --- | --- | --- | --- | --- | --- | --- | --- | --- | --- | --- |
| **1** | APFPEVFGK | 9 | Alpha S1 Casein | 2 | 496.27 | 990.52 | 44.00 | 41-49 |  | + | 1 |
| **2** | DIPNPIGSENSEKTTMPLW | 19 | Alpha S1 Casein | 2 | 1065.01 | 2128.01 | 47.96 | 196-214 |  | + | 1 |
| **3** | FFVAPFPEVFGK | 12 | Alpha S1 Casein | 2 | 692.87 | 1383.72 | 49.82 | 38-49 |  | + | 1 |
| **4** | FSDIPNPIGSENSEK | 15 | Alpha S1 Casein | 2 | 817.39 | 1632.76 | 41.94 | 194-208 |  | + | 1 |
| **5** | FSDIPNPIGSENSEKTT | 17 | Alpha S1 Casein | 2 | 918.44 | 1834.86 | 42.05 | 194-210 |  | + | 1 |
| **6** | FSDIPNPIGSENSEKTTMPLW | 21 | Alpha S1 Casein | 2 | 1190.06 | 2378.11 | 46.00 | 194-214 |  | + | 1 |
| **7** | FVAPFPEVFGK | 11 | Alpha S1 Casein | 2 | 619.33 | 1236.65 | 47.34 | 39-49 |  | + | 1 |
| **8** | GLPQEVLNENLLR | 13 | Alpha S1 Casein | 2 | 747.92 | 1493.82 | 45.29 | 25-37 |  | + | 1 |
| **9** | IPNPIGSENSEKT | 13 | Alpha S1 Casein | 2 | 693.35 | 1384.68 | 33.58 | 197-209 |  | + | 1 |
| **10** | IPNPIGSENSEKTT | 14 | Alpha S1 Casein | 2 | 743.87 | 1485.73 | 33.74 | 197-210 |  | + | 1 |
| **11** | IPNPIGSENSEKTTMPLW | 18 | Alpha S1 Casein | 2 | 1015.50 | 2028.98 | 44.46 | 197-214 |  | + | 1 |
| **12** | LRL | 3 | Alpha S1 Casein | 1 | 401.29 | 400.28 | 28.00 | 114-116 |  | + | 1 |
| **13** | PIGSENSEKTTMPLW | 15 | Alpha S1 Casein | 2 | 853.41 | 1704.80 | 42.46 | 200-214 |  | + | 1 |
| **14** | QFY | 3 | Alpha S1 Casein | 1 | 457.21 | 456.20 | 34.10 | 167-169 |  | + | 1 |
| **15** | QGLPQEVLNENLL | 13 | Alpha S1 Casein | 2 | 733.90 | 1465.78 | 48.88 | 24-36 |  | + | 1 |
| **16** | QGLPQEVLNENLLR | 14 | Alpha S1 Casein | 2 | 811.95 | 1621.88 | 45.16 | 24-37 |  | + | 1 |
| **17** | SDIPNPIGSENSEKTTMPLW | 20 | Alpha S1 Casein | 2 | 1108.53 | 2215.05 | 46.63 | 195-214 |  | + | 1 |
| **18** | SEKTTMPLW | 9 | Alpha S1 Casein | 2 | 546.77 | 1091.53 | 43.84 | 206-214 |  | + | 1 |
| **19** | VAPFPEVFGK | 10 | Alpha S1 Casein | 2 | 545.80 | 1089.59 | 44.92 | 40-49 |  | + | 1 |
| **20** | VAPFPEVFGKEKV | 13 | Alpha S1 Casein | 2 | 723.90 | 1445.79 | 43.27 | 40-52 |  | + | 1 |
| **21** | VAPFPEVFGKEKVN | 14 | Alpha S1 Casein | 2 | 780.92 | 1559.83 | 42.76 | 40-53 |  | + | 1 |
| **22** | YTD | 3 | Alpha S1 Casein | 1 | 398.16 | 397.15 | 55.46 | 188-190 |  | + | 1 |
| **23** | ALPQYLKTVYQ | 11 | Alpha S2 Casein | 2 | 662.37 | 1322.72 | 43.20 | 190-200 |  | + | 1 |
| **24** | FALPQYLKT | 9 | Alpha S2 Casein | 2 | 540.81 | 1079.60 | 44.40 | 189-197 |  | + | 1 |
| **25** | FALPQYLKTVYQ | 12 | Alpha S2 Casein | 2 | 735.90 | 1469.79 | 46.09 | 189-200 |  | + | 1 |
| **26** | LNF | 3 | Alpha S2 Casein | 1 | 393.21 | 392.21 | 41.48 | 570-572 |  | + | 1 |
| **27** | QPK | 3 | Alpha S2 Casein | 1 | 372.22 | 371.22 | 29.70 | 210-212 |  | + | 1 |
| **28** | VVR | 3 | Alpha S2 Casein | 1 | 373.26 | 372.25 | 55.36 | 1124-1126 |  | + | 1 |
| **29** | YLK | 3 | Alpha S2 Casein | 1 | 423.26 | 422.25 | 2.16 | 194-196 |  | + | 1 |
| **30** | DKIHPFAQTQ | 10 | Beta Casein | 2 | 592.81 | 1183.60 | 28.40 | 62-71 |  | + | 1 |
| **31** | DKIHPFAQTQS | 11 | Beta Casein | 2 | 636.32 | 1270.63 | 28.21 | 62-72 |  | + | 1 |
| **32** | EEL | 3 | Beta Casein | 1 | 390.19 | 389.18 | 25.27 | 19-21 |  | + | 1 |
| **33** | EPVLGPVRGPFPII | 14 | Beta Casein | 2 | 745.94 | 1489.87 | 49.07 | 210-223 |  | + | 1 |
| **34** | EPVLGPVRGPFPIIV | 15 | Beta Casein | 2 | 795.47 | 1588.93 | 49.97 | 210-224 |  | + | 1 |
| **35** | GPVRGPFPIIV | 11 | Beta Casein | 2 | 576.35 | 1150.69 | 46.95 | 214-224 |  | + | 1 |
| **36** | IHPFAQTQS | 9 | Beta Casein | 2 | 514.76 | 1027.51 | 29.21 | 64-72 |  | + | 1 |
| **37** | LGPVRGPFPIIV | 12 | Beta Casein | 2 | 632.89 | 1263.77 | 48.36 | 213-224 |  | + | 1 |
| **38** | MPFPKYPVEPFTESQSL | 17 | Beta Casein | 2 | 998.99 | 1995.97 | 46.44 | 124-140 |  | + | 1 |
| **39** | NVPGEIVESLSSSEESITR | 19 | Beta Casein | 2 | 1017.01 | 2032.00 | 45.72 | 22-40 |  | + | 1 |
| **40** | QEPVLGPVRGPFPI | 14 | Beta Casein | 2 | 753.43 | 1504.84 | 46.91 | 209-222 |  | + | 1 |
| **41** | QEPVLGPVRGPFPII | 15 | Beta Casein | 2 | 809.97 | 1617.92 | 48.76 | 209-223 |  | + | 1 |
| **42** | QEPVLGPVRGPFPIIV | 16 | Beta Casein | 2 | 859.50 | 1716.99 | 49.64 | 209-224 |  | + | 1 |
| **43** | QQQ | 3 | Beta Casein | 1 | 403.19 | 402.19 | 39.61 | 53-55 |  | + | 1 |
| **44** | QQQTEDELQDK | 11 | Beta Casein | 2 | 681.31 | 1360.61 | 22.45 | 53-63 |  | + | 1 |
| **45** | QQTEDELQDK | 10 | Beta Casein | 2 | 617.28 | 1232.55 | 22.16 | 54-63 |  | + | 1 |
| **46** | TEDELQDK | 8 | Beta Casein | 2 | 489.22 | 976.43 | 20.34 | 56-63 |  | + | 1 |
| **47** | YQEPVLGPVRGPFP | 14 | Beta Casein | 2 | 778.42 | 1554.82 | 45.16 | 208-221 |  | + | 1 |
| **48** | YQEPVLGPVRGPFPI | 15 | Beta Casein | 2 | 834.96 | 1667.90 | 47.47 | 208-222 |  | + | 1 |
| **49** | YQEPVLGPVRGPFPII | 16 | Beta Casein | 2 | 891.50 | 1780.99 | 49.23 | 208-223 |  | + | 1 |
| **50** | YQEPVLGPVRGPFPIIV | 17 | Beta Casein | 2 | 941.04 | 1880.06 | 50.07 | 208-224 |  | + | 1 |
| **51** | EQNQEQPIR | 9 | Kappa Casein | 2 | 571.28 | 1140.55 | 20.00 | 23-31 |  | + | 1 |
| **52** | ESPPEINTVQVTSTAV | 16 | Kappa Casein | 2 | 836.43 | 1670.84 | 44.10 | 175-190 |  | + | 1 |
| **53** | FFL | 3 | Kappa Casein | 1 | 426.24 | 425.23 | 56.69 | 5-7 |  | + | 1 |
| **54** | PEVIESPPEINTVQVTSTAV | 20 | Kappa Casein | 2 | 1055.55 | 2109.08 | 46.06 | 171-190 |  | + | 1 |
| **55** | SPPEINTVQVTSTAV | 15 | Kappa Casein | 2 | 771.90 | 1541.79 | 43.79 | 176-190 |  | + | 1 |
| **56** | VQVTSTAV | 8 | Kappa Casein | 1 | 804.45 | 803.44 | 33.52 | 183-190 |  | + | 1 |

**Supplementary Table 10.** Milk peptides identified throughout the 28 days of storage period using the *Lb. brevis* NCCB 100526.

|  | **Sequence** | **Length** | **Leading proteins** | **Charge** | **m/z** | **Mass** | **Retention time** | **Sequence** | **Day 1** | **Day 28** | **Incidence** |
| --- | --- | --- | --- | --- | --- | --- | --- | --- | --- | --- | --- |
| **1** | FHTSGYDTQA | 10 | Alpha Lactalbumin | 2 | 563.74 | 1125.47 | 26.64 | 50-59 | + |  | 1 |
| **2** | LFH | 3 | Alpha Lactalbumin | 1 | 416.23 | 415.22 | 35.48 | 13-15 | + |  | 1 |
| **3** | LKDLKGYGG | 9 | Alpha Lactalbumin | 2 | 475.77 | 949.52 | 24.72 | 31-39 | + |  | 1 |
| **4** | QNN | 3 | Alpha Lactalbumin | 1 | 375.16 | 374.15 | 49.49 | 62-64 | + |  | 1 |
| **5** | VKK | 3 | Alpha Lactalbumin | 1 | 374.28 | 373.27 | 56.90 | 111-113 | + |  | 1 |
| **6** | WVC | 3 | Alpha Lactalbumin | 1 | 464.20 | 463.19 | 37.68 | 45-47 | + |  | 1 |
| **7** | YDT | 3 | Alpha Lactalbumin | 1 | 398.16 | 397.15 | 59.77 | 55-57 | + |  | 1 |
| **8** | AEERLHSM | 8 | Alpha S1 Casein | 2 | 486.73 | 971.45 | 20.27 | 131-138 | + |  | 2 |
| **9** | AMEDIKQ | 7 | Alpha S1 Casein | 2 | 417.70 | 833.40 | 23.07 | 68-74 | + | + | 2 |
| **10** | APFPEVFGK | 9 | Alpha S1 Casein | 2 | 496.27 | 990.52 | 43.90 | 41-49 | + | + | 2 |
| **11** | APFPEVFGKEK | 11 | Alpha S1 Casein | 2 | 624.83 | 1247.66 | 40.90 | 41-51 | + | + | 2 |
| **12** | APSFSDIPNPIGSEN | 15 | Alpha S1 Casein | 2 | 772.87 | 1543.72 | 45.99 | 191-205 | + | + | 2 |
| **13** | DQAMEDIKQ | 9 | Alpha S1 Casein | 2 | 539.25 | 1076.48 | 27.95 | 66-74 | + |  | 1 |
| **14** | EERLHSM | 7 | Alpha S1 Casein | 2 | 451.21 | 900.41 | 19.71 | 132-138 | + | + | 2 |
| **15** | EKTTMPLW | 8 | Alpha S1 Casein | 2 | 503.26 | 1004.50 | 43.96 | 207-214 | + | + | 2 |
| **16** | EVFGKEKV | 8 | Alpha S1 Casein | 2 | 468.26 | 934.51 | 25.71 | 45-52 | + |  | 1 |
| **17** | FFVAPFPEVFGK | 12 | Alpha S1 Casein | 2 | 692.87 | 1383.72 | 49.73 | 38-49 | + | + | 2 |
| **18** | FFVAPFPEVFGKEK | 14 | Alpha S1 Casein | 3 | 547.96 | 1640.86 | 46.50 | 38-51 | + | + | 2 |
| **19** | FFVAPFPEVFGKEKV | 15 | Alpha S1 Casein | 3 | 580.98 | 1739.93 | 46.99 | 38-52 | + | + | 2 |
| **20** | FFVAPFPEVFGKEKVNE | 17 | Alpha S1 Casein | 3 | 662.01 | 1983.01 | 46.55 | 38-54 | + | + | 2 |
| **21** | FPEVFGK | 7 | Alpha S1 Casein | 2 | 412.22 | 822.43 | 42.33 | 43-49 | + | + | 2 |
| **22** | FPEVFGKEK | 9 | Alpha S1 Casein | 2 | 540.79 | 1079.57 | 36.31 | 43-51 | + | + | 2 |
| **23** | FPEVFGKEKV | 10 | Alpha S1 Casein | 2 | 590.32 | 1178.63 | 40.38 | 43-52 | + | + | 2 |
| **24** | FPEVFGKEKVNE | 12 | Alpha S1 Casein | 2 | 711.87 | 1421.72 | 38.66 | 43-54 | + |  | 1 |
| **25** | FSDIPNPIGSEN | 12 | Alpha S1 Casein | 2 | 645.30 | 1288.59 | 44.52 | 194-205 | + | + | 2 |
| **26** | FSDIPNPIGSENSEK | 15 | Alpha S1 Casein | 2 | 817.39 | 1632.76 | 41.88 | 194-208 | + | + | 2 |
| **27** | FSDIPNPIGSENSEKTTMPLW | 21 | Alpha S1 Casein | 2 | 1182.06 | 2362.12 | 47.62 | 194-214 | + | + | 2 |
| **28** | FVAPFPEVFGK | 11 | Alpha S1 Casein | 2 | 619.33 | 1236.65 | 47.26 | 39-49 | + | + | 2 |
| **29** | FVAPFPEVFGKEK | 13 | Alpha S1 Casein | 3 | 498.94 | 1493.79 | 44.51 | 39-51 | + | + | 2 |
| **30** | FVAPFPEVFGKEKV | 14 | Alpha S1 Casein | 3 | 531.96 | 1592.86 | 45.03 | 39-52 | + | + | 2 |
| **31** | FVAPFPEVFGKEKVN | 15 | Alpha S1 Casein | 2 | 854.46 | 1706.90 | 44.66 | 39-53 | + | + | 2 |
| **32** | FVAPFPEVFGKEKVNE | 16 | Alpha S1 Casein | 3 | 612.99 | 1835.95 | 44.62 | 39-54 | + | + | 2 |
| **33** | FYPELFRQ | 8 | Alpha S1 Casein | 2 | 550.28 | 1098.55 | 44.23 | 160-167 | + |  | 1 |
| **34** | GLPQEVLNENLLR | 13 | Alpha S1 Casein | 2 | 747.92 | 1493.82 | 45.23 | 25-37 | + | + | 2 |
| **35** | HIQKEDVPS | 9 | Alpha S1 Casein | 2 | 526.77 | 1051.53 | 19.10 | 95-103 | + |  | 1 |
| **36** | IGSENSEKTTMPLW | 14 | Alpha S1 Casein | 2 | 796.88 | 1591.76 | 44.31 | 201-214 | + |  | 1 |
| **37** | IHAQQKEP | 8 | Alpha S1 Casein | 2 | 475.76 | 949.50 | 15.14 | 142-149 | + | + | 2 |
| **38** | IHAQQKEPM | 9 | Alpha S1 Casein | 2 | 541.28 | 1080.54 | 18.43 | 142-150 | + |  | 1 |
| **39** | IHAQQKEPMIGVNQEL | 16 | Alpha S1 Casein | 3 | 612.32 | 1833.94 | 38.18 | 142-157 | + |  | 1 |
| **40** | IKHQGLPQE | 9 | Alpha S1 Casein | 2 | 525.29 | 1048.57 | 21.13 | 21-29 | + | + | 2 |
| **41** | IKHQGLPQEV | 10 | Alpha S1 Casein | 2 | 574.82 | 1147.63 | 27.41 | 21-30 | + |  | 1 |
| **42** | IPNPIGSEN | 9 | Alpha S1 Casein | 2 | 470.74 | 939.47 | 39.64 | 197-205 | + | + | 2 |
| **43** | IPNPIGSENSEK | 12 | Alpha S1 Casein | 2 | 642.83 | 1283.64 | 32.90 | 197-208 | + | + | 2 |
| **44** | IVPNSVEQK | 9 | Alpha S1 Casein | 2 | 507.28 | 1012.56 | 24.43 | 86-94 | + |  | 1 |
| **45** | KEDVPSER | 8 | Alpha S1 Casein | 2 | 480.24 | 958.47 | 16.33 | 98-105 | + |  | 1 |
| **46** | KHQGLPQE | 8 | Alpha S1 Casein | 2 | 468.75 | 935.48 | 18.05 | 22-29 | + |  | 1 |
| **47** | KYKVPQL | 7 | Alpha S1 Casein | 2 | 438.27 | 874.53 | 32.41 | 118-124 | + | + | 2 |
| **48** | KYKVPQLE | 8 | Alpha S1 Casein | 2 | 502.79 | 1003.57 | 30.98 | 118-125 | + | + | 2 |
| **49** | LEQLLR | 6 | Alpha S1 Casein | 2 | 386.24 | 770.47 | 35.11 | 110-115 | + | + | 2 |
| **50** | LNENLLR | 7 | Alpha S1 Casein | 2 | 436.25 | 870.49 | 35.26 | 31-37 | + | + | 2 |
| **51** | LNENLLRF | 8 | Alpha S1 Casein | 2 | 509.79 | 1017.56 | 44.74 | 31-38 | + | + | 2 |
| **52** | LPQEVLNENLLR | 12 | Alpha S1 Casein | 2 | 719.41 | 1436.80 | 44.01 | 26-37 | + | + | 2 |
| **53** | MEDIKQ | 6 | Alpha S1 Casein | 2 | 382.19 | 762.36 | 20.74 | 69-74 | + |  | 1 |
| **54** | MEDIKQME | 8 | Alpha S1 Casein | 2 | 520.23 | 1038.44 | 22.96 | 69-76 | + | + | 2 |
| **55** | NSEKTTMPLW | 10 | Alpha S1 Casein | 2 | 603.79 | 1205.58 | 43.62 | 205-214 | + | + | 2 |
| **56** | PIGSENSEK | 9 | Alpha S1 Casein | 2 | 480.74 | 959.46 | 15.96 | 200-208 | + | + | 2 |
| **57** | PIGSENSEKTT | 11 | Alpha S1 Casein | 2 | 581.78 | 1161.55 | 18.08 | 200-210 | + |  | 1 |
| **58** | PIGSENSEKTTMP | 13 | Alpha S1 Casein | 2 | 695.83 | 1389.64 | 29.87 | 200-212 | + | + | 2 |
| **59** | PIGSENSEKTTMPLW | 15 | Alpha S1 Casein | 2 | 853.41 | 1704.80 | 42.38 | 200-214 | + | + | 2 |
| **60** | QAMEDIKQ | 8 | Alpha S1 Casein | 2 | 481.73 | 961.45 | 24.32 | 67-74 | + | + | 2 |
| **61** | QLLRLK | 6 | Alpha S1 Casein | 2 | 385.77 | 769.52 | 29.79 | 112-117 | + | + | 2 |
| **62** | RPK | 3 | Alpha S1 Casein | 1 | 400.27 | 399.26 | 58.81 | 16-18 | + | + | 2 |
| **63** | RQF | 3 | Alpha S1 Casein | 1 | 450.25 | 449.24 | 43.11 | 166-168 | + | + | 2 |
| **64** | SDIPNPIGSEN | 11 | Alpha S1 Casein | 2 | 571.77 | 1141.53 | 42.27 | 195-205 | + | + | 2 |
| **65** | SDIPNPIGSENSEK | 14 | Alpha S1 Casein | 2 | 743.85 | 1485.69 | 36.66 | 195-208 | + | + | 2 |
| **66** | SDIPNPIGSENSEKTTMPLW | 20 | Alpha S1 Casein | 2 | 1108.53 | 2215.05 | 46.55 | 195-214 | + | + | 2 |
| **67** | SEKTTMPLW | 9 | Alpha S1 Casein | 2 | 554.77 | 1107.53 | 39.50 | 206-214 | + | + | 2 |
| **68** | SENSEKTTMPLW | 12 | Alpha S1 Casein | 2 | 711.83 | 1421.65 | 43.85 | 203-214 | + | + | 2 |
| **69** | SERYLGYLE | 9 | Alpha S1 Casein | 2 | 565.28 | 1128.55 | 42.02 | 103-111 | + | + | 2 |
| **70** | SERYLGYLEQ | 10 | Alpha S1 Casein | 2 | 629.31 | 1256.60 | 41.66 | 103-112 | + | + | 2 |
| **71** | TDAPSFSD | 8 | Alpha S1 Casein | 1 | 839.34 | 838.33 | 34.87 | 189-196 | + |  | 1 |
| **72** | VAPFPEVFGK | 10 | Alpha S1 Casein | 2 | 545.80 | 1089.59 | 44.86 | 40-49 | + | + | 2 |
| **73** | VAPFPEVFGKE | 11 | Alpha S1 Casein | 2 | 610.32 | 1218.63 | 44.92 | 40-50 | + | + | 2 |
| **74** | VAPFPEVFGKEK | 12 | Alpha S1 Casein | 2 | 674.37 | 1346.72 | 42.33 | 40-51 | + | + | 2 |
| **75** | VAPFPEVFGKEKV | 13 | Alpha S1 Casein | 2 | 723.90 | 1445.79 | 43.16 | 40-52 | + | + | 2 |
| **76** | VAPFPEVFGKEKVN | 14 | Alpha S1 Casein | 2 | 780.92 | 1559.83 | 42.42 | 40-53 | + | + | 2 |
| **77** | VAPFPEVFGKEKVNE | 15 | Alpha S1 Casein | 3 | 563.97 | 1688.88 | 42.67 | 40-54 | + | + | 2 |
| **78** | VEQKHIQKED | 10 | Alpha S1 Casein | 2 | 627.33 | 1252.64 | 13.87 | 91-100 | + |  | 1 |
| **79** | VFGKEKV | 7 | Alpha S1 Casein | 2 | 403.74 | 805.47 | 21.48 | 46-52 | + |  | 1 |
| **80** | VFGKEKVN | 8 | Alpha S1 Casein | 2 | 460.76 | 919.51 | 20.04 | 46-53 | + |  | 1 |
| **81** | VLNENLLR | 8 | Alpha S1 Casein | 2 | 485.79 | 969.56 | 38.85 | 30-37 | + | + | 2 |
| **82** | VLNENLLRF | 9 | Alpha S1 Casein | 2 | 559.32 | 1116.63 | 45.35 | 30-38 | + | + | 2 |
| **83** | VLNENLLRFF | 10 | Alpha S1 Casein | 2 | 632.86 | 1263.70 | 49.44 | 30-39 | + | + | 2 |
| **84** | VPSERYL | 7 | Alpha S1 Casein | 2 | 432.23 | 862.45 | 32.39 | 101-107 | + | + | 2 |
| **85** | VPSERYLG | 8 | Alpha S1 Casein | 2 | 460.75 | 919.48 | 30.31 | 101-108 | + | + | 2 |
| **86** | VPSERYLGY | 9 | Alpha S1 Casein | 2 | 542.28 | 1082.54 | 37.36 | 101-109 | + | + | 2 |
| **87** | VPSERYLGYLE | 11 | Alpha S1 Casein | 2 | 663.34 | 1324.67 | 43.05 | 101-111 | + | + | 2 |
| **88** | VPSERYLGYLEQ | 12 | Alpha S1 Casein | 2 | 727.37 | 1452.72 | 42.78 | 101-112 | + | + | 2 |
| **89** | WYY | 3 | Alpha S1 Casein | 1 | 531.22 | 530.22 | 43.31 | 179-181 | + |  | 1 |
| **90** | YKVPQLE | 7 | Alpha S1 Casein | 2 | 438.74 | 875.48 | 37.34 | 119-125 | + | + | 2 |
| **91** | YLEQLLR | 7 | Alpha S1 Casein | 2 | 467.77 | 933.53 | 43.11 | 109-115 | + | + | 2 |
| **92** | AMEDIKQME | 9 | Alpha S1 Casein | 2 | 547.75 | 1093.48 | 33.29 | 68-76 |  | + | 1 |
| **93** | APFPEVFGKE | 10 | Alpha S1 Casein | 2 | 560.79 | 1119.56 | 43.89 | 41-50 |  | + | 1 |
| **94** | APFPEVFGKEKVN | 13 | Alpha S1 Casein | 2 | 731.39 | 1460.77 | 41.54 | 41-53 |  | + | 1 |
| **95** | APSFSDIPNPIGSENSEK | 18 | Alpha S1 Casein | 2 | 944.95 | 1887.89 | 43.50 | 191-208 |  | + | 1 |
| **96** | EDV | 3 | Alpha S1 Casein | 1 | 362.16 | 361.15 | 30.77 | 99-101 |  | + | 1 |
| **97** | EER | 3 | Alpha S1 Casein | 1 | 433.20 | 432.20 | 40.34 | 132-134 |  | + | 1 |
| **98** | EIVPNSVEQK | 10 | Alpha S1 Casein | 2 | 571.81 | 1141.60 | 30.10 | 85-94 |  | + | 1 |
| **99** | ENLLRF | 6 | Alpha S1 Casein | 2 | 396.22 | 790.43 | 43.62 | 33-38 |  | + | 1 |
| **100** | ENLLRFF | 7 | Alpha S1 Casein | 2 | 469.76 | 937.50 | 47.89 | 33-39 |  | + | 1 |
| **101** | EVLNENLLR | 9 | Alpha S1 Casein | 2 | 550.31 | 1098.60 | 41.83 | 29-37 |  | + | 1 |
| **102** | EVLNENLLRF | 10 | Alpha S1 Casein | 2 | 623.84 | 1245.67 | 46.14 | 29-38 |  | + | 1 |
| **103** | IVPNSAEER | 9 | Alpha S1 Casein | 2 | 507.76 | 1013.51 | 21.47 | 126-134 |  | + | 1 |
| **104** | KEDVPSERY | 9 | Alpha S1 Casein | 2 | 561.77 | 1121.54 | 22.37 | 98-106 |  | + | 1 |
| **105** | LEQLLRL | 7 | Alpha S1 Casein | 2 | 442.78 | 883.55 | 44.52 | 110-116 |  | + | 1 |
| **106** | LEQLLRLK | 8 | Alpha S1 Casein | 2 | 506.83 | 1011.64 | 38.95 | 110-117 |  | + | 1 |
| **107** | LGYLEQLLR | 9 | Alpha S1 Casein | 2 | 552.82 | 1103.63 | 46.30 | 107-115 |  | + | 1 |
| **108** | LLR | 3 | Alpha S1 Casein | 1 | 401.29 | 400.28 | 17.80 | 35-37 |  | + | 1 |
| **109** | LNENLLRFF | 9 | Alpha S1 Casein | 2 | 583.32 | 1164.63 | 48.73 | 31-39 |  | + | 1 |
| **110** | LRL | 3 | Alpha S1 Casein | 1 | 401.29 | 400.28 | 18.50 | 114-116 |  | + | 1 |
| **111** | NENLLRF | 7 | Alpha S1 Casein | 2 | 453.25 | 904.48 | 43.39 | 32-38 |  | + | 1 |
| **112** | NENLLRFF | 8 | Alpha S1 Casein | 2 | 526.78 | 1051.55 | 47.60 | 32-39 |  | + | 1 |
| **113** | QGLPQEVLNENLLR | 14 | Alpha S1 Casein | 2 | 811.95 | 1621.88 | 45.04 | 24-37 |  | + | 1 |
| **114** | RLK | 3 | Alpha S1 Casein | 1 | 416.30 | 415.29 | 25.47 | 115-117 |  | + | 1 |
| **115** | SDIPNPIGSENSEKTT | 16 | Alpha S1 Casein | 2 | 844.90 | 1687.79 | 37.02 | 195-210 |  | + | 1 |
| **116** | SERYLGY | 7 | Alpha S1 Casein | 2 | 444.22 | 886.42 | 32.79 | 103-109 |  | + | 1 |
| **117** | SFSDIPNPIGSEN | 13 | Alpha S1 Casein | 2 | 688.82 | 1375.63 | 45.09 | 193-205 |  | + | 1 |
| **118** | VPNSAEER | 8 | Alpha S1 Casein | 2 | 451.22 | 900.43 | 16.17 | 127-134 |  | + | 1 |
| **119** | VPSERY | 6 | Alpha S1 Casein | 2 | 375.69 | 749.37 | 21.40 | 101-106 |  | + | 1 |
| **120** | YPELFRQ | 7 | Alpha S1 Casein | 2 | 476.75 | 951.48 | 41.55 | 161-167 |  | + | 1 |
| **121** | YTD | 3 | Alpha S1 Casein | 1 | 398.16 | 397.15 | 59.13 | 188-190 |  | + | 1 |
| **122** | ALPQYLKT | 8 | Alpha S2 Casein | 2 | 467.27 | 932.53 | 38.58 | 190-197 | + | + | 2 |
| **123** | AMKPWIQPK | 9 | Alpha S2 Casein | 2 | 549.81 | 1097.61 | 32.15 | 204-212 | + | + | 2 |
| **124** | AMKPWIQPKTK | 11 | Alpha S2 Casein | 3 | 443.26 | 1326.75 | 27.79 | 204-214 | + |  | 1 |
| **125** | AVPITPTL | 8 | Alpha S2 Casein | 1 | 811.49 | 810.49 | 45.23 | 131-138 | + |  | 1 |
| **126** | AVPITPTLNREQ | 12 | Alpha S2 Casein | 2 | 669.87 | 1337.73 | 37.53 | 131-142 | + |  | 1 |
| **127** | EEKNRLNF | 8 | Alpha S2 Casein | 2 | 525.27 | 1048.53 | 28.55 | 171-178 | + | + | 2 |
| **128** | EEKNRLNFLK | 10 | Alpha S2 Casein | 3 | 430.91 | 1289.71 | 30.79 | 171-180 | + |  | 1 |
| **129** | EEY | 3 | Alpha S2 Casein | 1 | 440.17 | 439.16 | 24.23 | 65-67 | + |  | 1 |
| **130** | EKNRLN | 6 | Alpha S2 Casein | 2 | 387.22 | 772.42 | 12.72 | 172-177 | + |  | 1 |
| **131** | EKNRLNF | 7 | Alpha S2 Casein | 2 | 460.75 | 919.49 | 27.07 | 172-178 | + | + | 2 |
| **132** | EVVRNANEEE | 10 | Alpha S2 Casein | 2 | 594.78 | 1187.54 | 19.22 | 57-66 | + | + | 2 |
| **133** | EVVRNANEEEY | 11 | Alpha S2 Casein | 2 | 676.31 | 1350.61 | 26.14 | 57-67 | + |  | 1 |
| **134** | FALPQYLK | 8 | Alpha S2 Casein | 2 | 490.28 | 978.55 | 44.01 | 189-196 | + | + | 2 |
| **135** | FALPQYLKT | 9 | Alpha S2 Casein | 2 | 540.81 | 1079.60 | 44.31 | 189-197 | + | + | 2 |
| **136** | FALPQYLKTVYQHQK | 15 | Alpha S2 Casein | 3 | 622.01 | 1863.00 | 42.99 | 189-203 | + | + | 2 |
| **137** | FLKKISQR | 8 | Alpha S2 Casein | 2 | 510.32 | 1018.63 | 19.51 | 178-185 | + |  | 1 |
| **138** | FYQKFPQY | 8 | Alpha S2 Casein | 2 | 560.78 | 1119.54 | 40.44 | 103-110 | + |  | 1 |
| **139** | FYQKFPQYL | 9 | Alpha S2 Casein | 2 | 617.32 | 1232.62 | 43.86 | 103-111 | + |  | 1 |
| **140** | FYQKFPQYLQ | 10 | Alpha S2 Casein | 2 | 681.35 | 1360.68 | 42.82 | 103-112 | + |  | 1 |
| **141** | IISQETYK | 8 | Alpha S2 Casein | 2 | 491.27 | 980.52 | 25.16 | 29-36 | + | + | 2 |
| **142** | IQPKTKVIPY | 10 | Alpha S2 Casein | 2 | 593.86 | 1185.71 | 31.97 | 209-218 | + |  | 1 |
| **143** | ISQRYQK | 7 | Alpha S2 Casein | 2 | 461.76 | 921.50 | 14.75 | 182-188 | + | + | 2 |
| **144** | ITVDDKHY | 8 | Alpha S2 Casein | 2 | 495.75 | 989.48 | 25.45 | 86-93 | + |  | 1 |
| **145** | IVLNPWDQVKRN | 12 | Alpha S2 Casein | 2 | 741.41 | 1480.82 | 42.08 | 119-130 | + |  | 1 |
| **146** | KFALPQY | 7 | Alpha S2 Casein | 2 | 433.74 | 865.47 | 42.22 | 188-194 | + | + | 2 |
| **147** | KFALPQYLK | 9 | Alpha S2 Casein | 2 | 554.33 | 1106.65 | 41.75 | 188-196 | + | + | 2 |
| **148** | KISQRYQK | 8 | Alpha S2 Casein | 2 | 525.81 | 1049.60 | 14.00 | 181-188 | + | + | 2 |
| **149** | KITVDDKHY | 9 | Alpha S2 Casein | 2 | 559.80 | 1117.58 | 21.60 | 85-93 | + |  | 1 |
| **150** | KKISQRYQ | 8 | Alpha S2 Casein | 2 | 525.81 | 1049.60 | 14.00 | 180-187 | + |  | 1 |
| **151** | KPWIQPK | 7 | Alpha S2 Casein | 2 | 448.77 | 895.53 | 24.84 | 206-212 | + |  | 1 |
| **152** | LKTVYQH | 7 | Alpha S2 Casein | 2 | 444.75 | 887.49 | 20.38 | 195-201 | + | + | 2 |
| **153** | LKTVYQHQ | 8 | Alpha S2 Casein | 2 | 508.78 | 1015.55 | 19.97 | 195-202 | + | + | 2 |
| **154** | LKTVYQHQK | 9 | Alpha S2 Casein | 3 | 382.22 | 1143.64 | 15.80 | 195-203 | + | + | 2 |
| **155** | LNFLKKISQ | 9 | Alpha S2 Casein | 2 | 545.83 | 1089.65 | 38.32 | 176-184 | + |  | 1 |
| **156** | LNFLKKISQR | 10 | Alpha S2 Casein | 3 | 416.26 | 1245.76 | 34.50 | 176-185 | + |  | 1 |
| **157** | LTEEEKNR | 8 | Alpha S2 Casein | 2 | 509.76 | 1017.51 | 14.19 | 168-175 | + |  | 1 |
| **158** | LTEEEKNRLN | 10 | Alpha S2 Casein | 2 | 623.33 | 1244.64 | 20.14 | 168-177 | + | + | 2 |
| **159** | LTEEEKNRLNF | 11 | Alpha S2 Casein | 2 | 696.86 | 1391.70 | 33.37 | 168-178 | + |  | 1 |
| **160** | LTEEEKNRLNFLK | 13 | Alpha S2 Casein | 3 | 545.30 | 1632.88 | 35.31 | 168-180 | + |  | 1 |
| **161** | MKPWIQPK | 8 | Alpha S2 Casein | 2 | 514.29 | 1026.57 | 31.45 | 205-212 | + |  | 1 |
| **162** | NMAINPSKEN | 10 | Alpha S2 Casein | 2 | 559.27 | 1116.52 | 26.26 | 40-49 | + | + | 2 |
| **163** | QGPIVLNPWDQVK | 13 | Alpha S2 Casein | 2 | 747.41 | 1492.80 | 45.08 | 116-128 | + |  | 1 |
| **164** | QGPIVLNPWDQVKRN | 15 | Alpha S2 Casein | 3 | 588.66 | 1762.95 | 43.05 | 116-130 | + |  | 1 |
| **165** | QKALNEINQ | 9 | Alpha S2 Casein | 2 | 529.29 | 1056.56 | 25.21 | 94-102 | + |  | 1 |
| **166** | STEVFTKK | 8 | Alpha S2 Casein | 2 | 470.26 | 938.51 | 19.08 | 158-165 | + |  | 1 |
| **167** | TEEEKNRL | 8 | Alpha S2 Casein | 2 | 509.76 | 1017.51 | 16.43 | 169-176 | + |  | 1 |
| **168** | TEEEKNRLN | 9 | Alpha S2 Casein | 2 | 566.78 | 1131.55 | 15.19 | 169-177 | + | + | 2 |
| **169** | TEVFTKK | 7 | Alpha S2 Casein | 2 | 426.74 | 851.48 | 18.08 | 159-165 | + | + | 2 |
| **170** | TKLTEEEK | 8 | Alpha S2 Casein | 2 | 489.26 | 976.51 | 15.89 | 166-173 | + | + | 2 |
| **171** | TKLTEEEKNR | 10 | Alpha S2 Casein | 3 | 416.56 | 1246.65 | 15.25 | 166-175 | + | + | 2 |
| **172** | TKLTEEEKNRLN | 12 | Alpha S2 Casein | 3 | 492.27 | 1473.78 | 21.36 | 166-177 | + |  | 1 |
| **173** | TKVIPYVR | 8 | Alpha S2 Casein | 2 | 488.30 | 974.59 | 30.61 | 213-220 | + | + | 2 |
| **174** | TKVIPYVRYL | 10 | Alpha S2 Casein | 2 | 626.38 | 1250.74 | 42.49 | 213-222 | + | + | 2 |
| **175** | TVYQHQK | 7 | Alpha S2 Casein | 2 | 452.24 | 902.46 | 13.78 | 197-203 | + | + | 2 |
| **176** | TVYQHQKA | 8 | Alpha S2 Casein | 2 | 487.76 | 973.50 | 14.43 | 197-204 | + |  | 1 |
| **177** | TYKQEKNM | 8 | Alpha S2 Casein | 2 | 521.26 | 1040.50 | 14.99 | 34-41 | + |  | 1 |
| **178** | TYKQEKNMA | 9 | Alpha S2 Casein | 2 | 556.77 | 1111.53 | 15.64 | 34-42 | + | + | 2 |
| **179** | VVRNANEEE | 9 | Alpha S2 Casein | 2 | 530.26 | 1058.50 | 15.79 | 58-66 | + |  | 1 |
| **180** | VVRNANEEEY | 10 | Alpha S2 Casein | 2 | 611.79 | 1221.56 | 22.57 | 58-67 | + | + | 2 |
| **181** | WIQPKTK | 7 | Alpha S2 Casein | 2 | 450.77 | 899.52 | 22.62 | 208-214 | + |  | 1 |
| **182** | WIQPKTKVIPY | 11 | Alpha S2 Casein | 2 | 686.90 | 1371.79 | 40.84 | 208-218 | + | + | 2 |
| **183** | YLQ | 3 | Alpha S2 Casein | 1 | 423.22 | 422.22 | 30.22 | 110-112 | + |  | 1 |
| **184** | YQKFPQY | 7 | Alpha S2 Casein | 2 | 487.24 | 972.47 | 34.26 | 104-110 | + | + | 2 |
| **185** | YQKFPQYL | 8 | Alpha S2 Casein | 2 | 543.78 | 1085.55 | 42.39 | 104-111 | + | + | 2 |
| **186** | YQKFPQYLQ | 9 | Alpha S2 Casein | 2 | 607.81 | 1213.61 | 40.69 | 104-112 | + |  | 1 |
| **187** | EEEKNRLNFL | 10 | Alpha S2 Casein | 2 | 646.34 | 1290.66 | 40.04 | 170-179 |  | + | 1 |
| **188** | EEKNRLNFL | 9 | Alpha S2 Casein | 2 | 581.81 | 1161.61 | 39.29 | 171-179 |  | + | 1 |
| **189** | EKNRLNFL | 8 | Alpha S2 Casein | 2 | 517.29 | 1032.57 | 38.01 | 172-179 |  | + | 1 |
| **190** | EVK | 3 | Alpha S2 Casein | 1 | 375.22 | 374.22 | 63.28 | 83-85 |  | + | 1 |
| **191** | FALPQYLKTVY | 11 | Alpha S2 Casein | 2 | 671.87 | 1341.73 | 46.30 | 189-199 |  | + | 1 |
| **192** | FALPQYLKTVYQ | 12 | Alpha S2 Casein | 2 | 735.90 | 1469.79 | 45.87 | 189-200 |  | + | 1 |
| **193** | FLKKISQ | 7 | Alpha S2 Casein | 2 | 432.27 | 862.53 | 22.51 | 178-184 |  | + | 1 |
| **194** | GPIVLNPWDQVK | 12 | Alpha S2 Casein | 2 | 683.38 | 1364.75 | 44.98 | 117-128 |  | + | 1 |
| **195** | ISQRYQ | 6 | Alpha S2 Casein | 2 | 397.71 | 793.41 | 17.63 | 182-187 |  | + | 1 |
| **196** | KFALPQYLKT | 10 | Alpha S2 Casein | 2 | 604.86 | 1207.70 | 42.10 | 188-197 |  | + | 1 |
| **197** | KISQRYQ | 7 | Alpha S2 Casein | 2 | 461.76 | 921.50 | 15.15 | 181-187 |  | + | 1 |
| **198** | KKT | 3 | Alpha S2 Casein | 1 | 376.26 | 375.25 | 17.45 | 151-153 |  | + | 1 |
| **199** | LPQYLK | 6 | Alpha S2 Casein | 2 | 381.23 | 760.45 | 33.57 | 191-196 |  | + | 1 |
| **200** | LPQYLKT | 7 | Alpha S2 Casein | 2 | 431.76 | 861.50 | 36.01 | 191-197 |  | + | 1 |
| **201** | LPQYLKTVY | 9 | Alpha S2 Casein | 2 | 562.82 | 1123.63 | 42.65 | 191-199 |  | + | 1 |
| **202** | LPQYLKTVYQ | 10 | Alpha S2 Casein | 2 | 626.85 | 1251.69 | 42.26 | 191-200 |  | + | 1 |
| **203** | LTEEEKNRLNFL | 12 | Alpha S2 Casein | 2 | 753.40 | 1504.79 | 41.48 | 168-179 |  | + | 1 |
| **204** | MESTEVFTK | 9 | Alpha S2 Casein | 2 | 536.25 | 1070.50 | 32.37 | 156-164 |  | + | 1 |
| **205** | NAVPITPTLNR | 11 | Alpha S2 Casein | 2 | 598.34 | 1194.67 | 38.37 | 130-140 |  | + | 1 |
| **206** | NMAINPSKE | 9 | Alpha S2 Casein | 2 | 502.25 | 1002.48 | 26.88 | 40-48 |  | + | 1 |
| **207** | QKFALPQY | 8 | Alpha S2 Casein | 2 | 497.77 | 993.53 | 41.84 | 187-194 |  | + | 1 |
| **208** | SIISQETYK | 9 | Alpha S2 Casein | 2 | 534.78 | 1067.55 | 31.65 | 28-36 |  | + | 1 |
| **209** | TYKQEKNMAIN | 11 | Alpha S2 Casein | 2 | 670.34 | 1338.66 | 21.79 | 34-44 |  | + | 1 |
| **210** | VRN | 3 | Alpha S2 Casein | 1 | 388.23 | 387.22 | 37.65 | 59-61 |  | + | 1 |
| **211** | VVR | 3 | Alpha S2 Casein | 1 | 373.26 | 372.25 | 55.32 | 58-60 |  | + | 1 |
| **212** | YQKFPQYLQY | 10 | Alpha S2 Casein | 2 | 689.35 | 1376.68 | 42.98 | 104-113 |  | + | 1 |
| **213** | YVR | 3 | Alpha S2 Casein | 1 | 437.25 | 436.24 | 58.96 | 218-220 |  | + | 1 |
| **214** | AVPYPQR | 7 | Beta Casein | 2 | 415.73 | 829.44 | 25.57 | 192-198 | + | + | 2 |
| **215** | AVPYPQRD | 8 | Beta Casein | 2 | 473.24 | 944.47 | 26.20 | 192-199 | + | + | 2 |
| **216** | AVPYPQRDMP | 10 | Beta Casein | 2 | 587.29 | 1172.56 | 35.82 | 192-201 | + | + | 2 |
| **217** | AVPYPQRDMPIQA | 13 | Beta Casein | 2 | 743.38 | 1484.74 | 41.38 | 192-204 | + | + | 2 |
| **218** | DELQDKIHP | 9 | Beta Casein | 2 | 547.78 | 1093.54 | 32.39 | 58-66 | + | + | 2 |
| **219** | DELQDKIHPF | 10 | Beta Casein | 2 | 621.31 | 1240.61 | 41.77 | 58-67 | + |  | 1 |
| **220** | DELQDKIHPFAQTQS | 15 | Beta Casein | 3 | 586.29 | 1755.84 | 40.67 | 58-72 | + | + | 2 |
| **221** | DKIHPFAQTQS | 11 | Beta Casein | 2 | 636.32 | 1270.63 | 28.11 | 62-72 | + | + | 2 |
| **222** | EDELQDKIHP | 10 | Beta Casein | 2 | 612.30 | 1222.58 | 32.55 | 57-66 | + | + | 2 |
| **223** | EESITRIN | 8 | Beta Casein | 2 | 481.25 | 960.49 | 28.49 | 35-42 | + |  | 1 |
| **224** | EESITRINK | 9 | Beta Casein | 2 | 545.30 | 1088.58 | 22.62 | 35-43 | + | + | 2 |
| **225** | ELNVPGEIVES | 11 | Beta Casein | 2 | 593.30 | 1184.59 | 44.45 | 20-30 | + |  | 1 |
| **226** | ELQDKIHP | 8 | Beta Casein | 2 | 490.26 | 978.51 | 25.52 | 59-66 | + | + | 2 |
| **227** | ELQDKIHPF | 9 | Beta Casein | 2 | 563.80 | 1125.58 | 37.72 | 59-67 | + | + | 2 |
| **228** | ELQDKIHPFAQTQ | 13 | Beta Casein | 2 | 777.90 | 1553.78 | 36.38 | 59-71 | + | + | 2 |
| **229** | ELQDKIHPFAQTQS | 14 | Beta Casein | 2 | 821.42 | 1640.82 | 34.67 | 59-72 | + | + | 2 |
| **230** | EMPFPKYP | 8 | Beta Casein | 2 | 504.75 | 1007.48 | 42.95 | 123-130 | + |  | 1 |
| **231** | EMPFPKYPVEP | 11 | Beta Casein | 2 | 667.33 | 1332.64 | 44.07 | 123-133 | + | + | 2 |
| **232** | EMPFPKYPVEPFTESQS | 17 | Beta Casein | 2 | 1006.97 | 2011.92 | 45.61 | 123-139 | + |  | 1 |
| **233** | EPVLGPVR | 8 | Beta Casein | 2 | 433.76 | 865.50 | 34.13 | 210-217 | + | + | 2 |
| **234** | EPVLGPVRGPFP | 12 | Beta Casein | 2 | 632.86 | 1263.70 | 44.74 | 210-221 | + | + | 2 |
| **235** | EPVLGPVRGPFPII | 14 | Beta Casein | 2 | 745.94 | 1489.87 | 49.02 | 210-223 | + | + | 2 |
| **236** | EPVLGPVRGPFPIIV | 15 | Beta Casein | 2 | 795.47 | 1588.93 | 49.90 | 210-224 | + | + | 2 |
| **237** | EQQQTEDELQDK | 12 | Beta Casein | 2 | 745.83 | 1489.65 | 23.50 | 52-63 | + | + | 2 |
| **238** | EQQQTEDELQDKIHP | 15 | Beta Casein | 2 | 919.43 | 1836.85 | 34.50 | 52-66 | + | + | 2 |
| **239** | EQQQTEDELQDKIHPFAQTQS | 21 | Beta Casein | 3 | 834.06 | 2499.15 | 41.07 | 52-72 | + | + | 2 |
| **240** | ESITRIN | 7 | Beta Casein | 2 | 416.73 | 831.45 | 26.41 | 36-42 | + | + | 2 |
| **241** | ESITRINK | 8 | Beta Casein | 2 | 480.78 | 959.54 | 20.45 | 36-43 | + | + | 2 |
| **242** | FLQPEVMGVSK | 11 | Beta Casein | 2 | 617.83 | 1233.64 | 42.27 | 102-112 | + | + | 2 |
| **243** | FLQPEVMGVSKVKE | 14 | Beta Casein | 2 | 795.93 | 1589.85 | 40.67 | 102-115 | + |  | 1 |
| **244** | FLQPEVMGVSKVKEA | 15 | Beta Casein | 2 | 831.45 | 1660.89 | 40.96 | 102-116 | + |  | 1 |
| **245** | FPKYPVEP | 8 | Beta Casein | 2 | 488.76 | 975.51 | 40.27 | 126-133 | + | + | 2 |
| **246** | FPKYPVEPF | 9 | Beta Casein | 2 | 562.29 | 1122.57 | 44.45 | 126-134 | + | + | 2 |
| **247** | FPKYPVEPFTE | 11 | Beta Casein | 2 | 677.34 | 1352.67 | 44.01 | 126-136 | + | + | 2 |
| **248** | FPKYPVEPFTES | 12 | Beta Casein | 2 | 720.86 | 1439.70 | 43.85 | 126-137 | + | + | 2 |
| **249** | FPKYPVEPFTESQS | 14 | Beta Casein | 2 | 828.40 | 1654.79 | 43.51 | 126-139 | + | + | 2 |
| **250** | FPKYPVEPFTESQSL | 15 | Beta Casein | 2 | 884.94 | 1767.87 | 44.91 | 126-140 | + | + | 2 |
| **251** | FQSEEQQQTEDELQDK | 16 | Beta Casein | 2 | 991.43 | 1980.85 | 31.61 | 48-63 | + |  | 1 |
| **252** | FTESQSLTLTDVEN | 14 | Beta Casein | 2 | 792.38 | 1582.74 | 44.19 | 134-147 | + |  | 1 |
| **253** | GPIPNSLPQN | 10 | Beta Casein | 2 | 518.77 | 1035.53 | 40.79 | 79-88 | + | + | 2 |
| **254** | GPVRGPFP | 8 | Beta Casein | 2 | 413.73 | 825.45 | 37.01 | 214-221 | + | + | 2 |
| **255** | GPVRGPFPII | 10 | Beta Casein | 2 | 526.82 | 1051.62 | 45.78 | 214-223 | + |  | 1 |
| **256** | GPVRGPFPIIV | 11 | Beta Casein | 2 | 576.35 | 1150.69 | 46.88 | 214-224 | + | + | 2 |
| **257** | HPFAQTQSL | 9 | Beta Casein | 2 | 514.76 | 1027.51 | 32.93 | 65-73 | + | + | 2 |
| **258** | IEKFQSE | 7 | Beta Casein | 2 | 440.72 | 879.43 | 23.97 | 45-51 | + | + | 2 |
| **259** | IEKFQSEE | 8 | Beta Casein | 2 | 505.25 | 1008.48 | 25.16 | 45-52 | + | + | 2 |
| **260** | IHPFAQTQ | 8 | Beta Casein | 2 | 471.25 | 940.48 | 29.48 | 64-71 | + | + | 2 |
| **261** | IHPFAQTQS | 9 | Beta Casein | 2 | 514.76 | 1027.51 | 29.19 | 64-72 | + | + | 2 |
| **262** | IHPFAQTQSL | 10 | Beta Casein | 2 | 571.30 | 1140.59 | 39.46 | 64-73 | + | + | 2 |
| **263** | IPPLTQTP | 8 | Beta Casein | 2 | 433.75 | 865.49 | 42.82 | 89-96 | + | + | 2 |
| **264** | IPPLTQTPVVVPPF | 14 | Beta Casein | 2 | 752.94 | 1503.87 | 52.30 | 89-102 | + |  | 1 |
| **265** | ITRINK | 6 | Beta Casein | 2 | 372.74 | 743.47 | 15.02 | 38-43 | + | + | 2 |
| **266** | KAVPYPQRD | 9 | Beta Casein | 2 | 537.29 | 1072.57 | 21.02 | 191-199 | + | + | 2 |
| **267** | KIEKFQSE | 8 | Beta Casein | 2 | 504.77 | 1007.53 | 19.75 | 44-51 | + | + | 2 |
| **268** | KIEKFQSEE | 9 | Beta Casein | 2 | 569.29 | 1136.57 | 21.01 | 44-52 | + | + | 2 |
| **269** | LGPVRGPFPIIV | 12 | Beta Casein | 2 | 632.89 | 1263.77 | 48.30 | 213-224 | + | + | 2 |
| **270** | LHLPLPLLQ | 9 | Beta Casein | 2 | 522.33 | 1042.65 | 49.17 | 148-156 | + | + | 2 |
| **271** | LHLPLPLLQS | 10 | Beta Casein | 2 | 565.85 | 1129.69 | 48.91 | 148-157 | + | + | 2 |
| **272** | LLYQEP | 6 | Beta Casein | 1 | 762.40 | 761.40 | 41.01 | 206-211 | + |  | 1 |
| **273** | LPLPLLQS | 8 | Beta Casein | 2 | 440.78 | 879.54 | 50.12 | 150-157 | + |  | 1 |
| **274** | LPQNIPPLTQTP | 12 | Beta Casein | 2 | 659.87 | 1317.73 | 44.77 | 85-96 | + |  | 1 |
| **275** | LQDKIHP | 7 | Beta Casein | 2 | 425.74 | 849.47 | 22.45 | 60-66 | + | + | 2 |
| **276** | LQDKIHPF | 8 | Beta Casein | 2 | 499.28 | 996.54 | 33.24 | 60-67 | + | + | 2 |
| **277** | LQDKIHPFAQTQ | 12 | Beta Casein | 2 | 713.38 | 1424.74 | 32.17 | 60-71 | + | + | 2 |
| **278** | LQDKIHPFAQTQS | 13 | Beta Casein | 2 | 756.89 | 1511.77 | 31.92 | 60-72 | + | + | 2 |
| **279** | LQPEVMGVSKVKEA | 14 | Beta Casein | 2 | 757.92 | 1513.82 | 34.32 | 103-116 | + |  | 1 |
| **280** | LSQSKVLP | 8 | Beta Casein | 2 | 436.27 | 870.52 | 32.03 | 180-187 | + | + | 2 |
| **281** | LSQSKVLPVPQK | 12 | Beta Casein | 3 | 441.94 | 1322.79 | 30.65 | 180-191 | + |  | 1 |
| **282** | LTDVENLH | 8 | Beta Casein | 2 | 470.74 | 939.47 | 31.56 | 142-149 | + | + | 2 |
| **283** | LTLTDVENLH | 10 | Beta Casein | 2 | 577.81 | 1153.60 | 43.74 | 140-149 | + | + | 2 |
| **284** | LVYPFPGPIPN | 11 | Beta Casein | 2 | 607.33 | 1212.65 | 49.12 | 73-83 | + | + | 2 |
| **285** | LVYPFPGPIPNS | 12 | Beta Casein | 2 | 650.85 | 1299.69 | 48.80 | 73-84 | + | + | 2 |
| **286** | LVYPFPGPIPNSLPQN | 16 | Beta Casein | 2 | 876.97 | 1751.92 | 49.59 | 73-88 | + | + | 2 |
| **287** | LYQEPVLGPVRGPFPIIV | 18 | Beta Casein | 2 | 997.58 | 1993.14 | 50.59 | 207-224 | + | + | 2 |
| **288** | MAPKHKEMP | 9 | Beta Casein | 2 | 534.77 | 1067.53 | 16.24 | 117-125 | + |  | 1 |
| **289** | MGVSKVKEA | 9 | Beta Casein | 2 | 474.76 | 947.51 | 19.14 | 108-116 | + |  | 1 |
| **290** | MHQPHQPLPPT | 11 | Beta Casein | 2 | 641.82 | 1281.63 | 26.14 | 159-169 | + |  | 1 |
| **291** | MPFPKYP | 7 | Beta Casein | 2 | 440.23 | 878.44 | 42.17 | 124-130 | + | + | 2 |
| **292** | MPFPKYPVEP | 10 | Beta Casein | 2 | 602.81 | 1203.60 | 43.39 | 124-133 | + | + | 2 |
| **293** | NLHLPLP | 7 | Beta Casein | 2 | 402.24 | 802.47 | 44.80 | 147-153 | + |  | 1 |
| **294** | NLHLPLPL | 8 | Beta Casein | 2 | 458.78 | 915.55 | 47.81 | 147-154 | + |  | 1 |
| **295** | NLHLPLPLLQS | 11 | Beta Casein | 2 | 622.87 | 1243.73 | 49.17 | 147-157 | + |  | 1 |
| **296** | PPFLQPEV | 8 | Beta Casein | 2 | 463.75 | 925.49 | 45.89 | 100-107 | + |  | 1 |
| **297** | PPLTQTPVVVPPFL | 14 | Beta Casein | 2 | 752.94 | 1503.87 | 52.30 | 90-103 | + |  | 1 |
| **298** | PVLGPVRGPFPIIV | 14 | Beta Casein | 2 | 730.95 | 1459.89 | 49.53 | 211-224 | + | + | 2 |
| **299** | PVRGPFPIIV | 10 | Beta Casein | 2 | 547.84 | 1093.66 | 46.85 | 215-224 | + | + | 2 |
| **300** | QDKIHPFAQTQS | 12 | Beta Casein | 2 | 700.35 | 1398.69 | 27.52 | 61-72 | + | + | 2 |
| **301** | QDKIHPFAQTQSL | 13 | Beta Casein | 3 | 504.93 | 1511.77 | 31.92 | 61-73 | + |  | 1 |
| **302** | QEPVLGPVRGPFPIIV | 16 | Beta Casein | 2 | 859.50 | 1716.99 | 49.58 | 209-224 | + | + | 2 |
| **303** | QPEVMGVSK | 9 | Beta Casein | 2 | 487.75 | 973.49 | 28.49 | 104-112 | + |  | 1 |
| **304** | QPH | 3 | Beta Casein | 1 | 381.19 | 380.18 | 65.59 | 161-163 | + | + | 2 |
| **305** | QQQ | 3 | Beta Casein | 1 | 403.19 | 402.19 | 31.14 | 53-55 | + |  | 1 |
| **306** | QQQTEDELQDKIHP | 14 | Beta Casein | 3 | 570.28 | 1707.81 | 30.86 | 53-66 | + | + | 2 |
| **307** | QQQTEDELQDKIHPFAQTQS | 20 | Beta Casein | 3 | 791.04 | 2370.11 | 37.84 | 53-72 | + | + | 2 |
| **308** | QQTEDELQDK | 10 | Beta Casein | 2 | 617.28 | 1232.55 | 22.14 | 54-63 | + | + | 2 |
| **309** | QQTEDELQDKIHP | 13 | Beta Casein | 2 | 790.88 | 1579.75 | 33.53 | 54-66 | + | + | 2 |
| **310** | QQTEDELQDKIHPFAQTQS | 19 | Beta Casein | 3 | 748.36 | 2242.05 | 40.52 | 54-72 | + | + | 2 |
| **311** | QSKVLPVPQK | 10 | Beta Casein | 2 | 562.35 | 1122.68 | 26.36 | 182-191 | + |  | 1 |
| **312** | QTEDELQDKIHP | 12 | Beta Casein | 2 | 726.85 | 1451.69 | 32.99 | 55-66 | + |  | 1 |
| **313** | RELEELN | 7 | Beta Casein | 2 | 451.73 | 901.45 | 28.34 | 16-22 | + | + | 2 |
| **314** | RELEELNVPGE | 11 | Beta Casein | 2 | 642.83 | 1283.64 | 40.62 | 16-26 | + | + | 2 |
| **315** | RGPFPIIV | 8 | Beta Casein | 2 | 449.78 | 897.54 | 45.84 | 217-224 | + | + | 2 |
| **316** | SEEQQQTEDELQDK | 14 | Beta Casein | 2 | 853.87 | 1705.73 | 26.31 | 50-63 | + |  | 1 |
| **317** | SEEQQQTEDELQDKIHPFAQTQS | 23 | Beta Casein | 3 | 906.08 | 2715.23 | 41.55 | 50-72 | + | + | 2 |
| **318** | SITRINK | 7 | Beta Casein | 2 | 416.26 | 830.50 | 17.26 | 37-43 | + | + | 2 |
| **319** | SKVLPVPQK | 9 | Beta Casein | 2 | 498.32 | 994.62 | 25.93 | 183-191 | + | + | 2 |
| **320** | SLPQNIPPLTQTP | 13 | Beta Casein | 2 | 703.39 | 1404.76 | 45.14 | 84-96 | + |  | 1 |
| **321** | SQSKVLPVPQK | 11 | Beta Casein | 2 | 605.86 | 1209.71 | 26.74 | 181-191 | + |  | 1 |
| **322** | SSSEESITRINK | 12 | Beta Casein | 2 | 675.85 | 1349.68 | 24.38 | 32-43 | + | + | 2 |
| **323** | TEDELQDK | 8 | Beta Casein | 2 | 489.22 | 976.43 | 20.33 | 56-63 | + | + | 2 |
| **324** | TEDELQDKIHP | 11 | Beta Casein | 2 | 662.82 | 1323.63 | 32.66 | 56-66 | + | + | 2 |
| **325** | TEDELQDKIHPF | 12 | Beta Casein | 2 | 736.36 | 1470.70 | 41.44 | 56-67 | + | + | 2 |
| **326** | TEDELQDKIHPFAQTQS | 17 | Beta Casein | 3 | 662.98 | 1985.93 | 40.21 | 56-72 | + | + | 2 |
| **327** | TLTDVENLH | 9 | Beta Casein | 2 | 521.26 | 1040.51 | 36.52 | 141-149 | + | + | 2 |
| **328** | TPVVVPPFL | 9 | Beta Casein | 2 | 484.79 | 967.57 | 52.15 | 95-103 | + |  | 1 |
| **329** | TPVVVPPFLQPE | 12 | Beta Casein | 2 | 661.87 | 1321.73 | 50.52 | 95-106 | + | + | 2 |
| **330** | VENLHLPLP | 9 | Beta Casein | 2 | 516.30 | 1030.58 | 45.23 | 145-153 | + |  | 1 |
| **331** | VENLHLPLPLLQS | 13 | Beta Casein | 2 | 736.93 | 1471.84 | 49.29 | 145-157 | + |  | 1 |
| **332** | VEPFTESQS | 9 | Beta Casein | 2 | 512.24 | 1022.46 | 37.28 | 131-139 | + |  | 1 |
| **333** | VKEAMAPK | 8 | Beta Casein | 2 | 437.25 | 872.48 | 16.29 | 113-120 | + | + | 2 |
| **334** | VLGPVRGPFP | 10 | Beta Casein | 2 | 519.81 | 1037.60 | 43.33 | 212-221 | + | + | 2 |
| **335** | VLGPVRGPFPI | 11 | Beta Casein | 2 | 576.35 | 1150.69 | 45.78 | 212-222 | + | + | 2 |
| **336** | VLGPVRGPFPII | 12 | Beta Casein | 2 | 632.89 | 1263.77 | 47.85 | 212-223 | + | + | 2 |
| **337** | VLGPVRGPFPIIV | 13 | Beta Casein | 2 | 682.43 | 1362.84 | 48.80 | 212-224 | + | + | 2 |
| **338** | VLPVPQK | 7 | Beta Casein | 2 | 390.75 | 779.49 | 29.53 | 185-191 | + | + | 2 |
| **339** | VLPVPQKA | 8 | Beta Casein | 2 | 426.27 | 850.53 | 31.68 | 185-192 | + |  | 1 |
| **340** | VLPVPQKAVPYPQRD | 15 | Beta Casein | 3 | 569.66 | 1705.95 | 37.97 | 185-199 | + | + | 2 |
| **341** | VLSLSQSK | 8 | Beta Casein | 2 | 431.26 | 860.50 | 29.14 | 177-184 | + | + | 2 |
| **342** | VLSLSQSKVLP | 11 | Beta Casein | 2 | 585.86 | 1169.70 | 42.99 | 177-187 | + | + | 2 |
| **343** | VLSLSQSKVLPVPQ | 14 | Beta Casein | 2 | 747.95 | 1493.88 | 43.85 | 177-190 | + | + | 2 |
| **344** | VLSLSQSKVLPVPQK | 15 | Beta Casein | 3 | 541.67 | 1621.98 | 41.38 | 177-191 | + | + | 2 |
| **345** | VMFPPQS | 7 | Beta Casein | 1 | 805.39 | 804.38 | 42.41 | 170-176 | + |  | 1 |
| **346** | VMGVSKVK | 8 | Beta Casein | 2 | 424.26 | 846.50 | 20.57 | 107-114 | + | + | 2 |
| **347** | VMGVSKVKE | 9 | Beta Casein | 2 | 488.78 | 975.54 | 22.51 | 107-115 | + | + | 2 |
| **348** | VMGVSKVKEA | 10 | Beta Casein | 2 | 524.30 | 1046.58 | 23.40 | 107-116 | + | + | 2 |
| **349** | VMGVSKVKEAM | 11 | Beta Casein | 2 | 589.82 | 1177.62 | 30.71 | 107-117 | + | + | 2 |
| **350** | VPGEIVESL | 9 | Beta Casein | 1 | 942.51 | 941.51 | 45.40 | 23-31 | + |  | 1 |
| **351** | VPYPQRD | 7 | Beta Casein | 2 | 437.72 | 873.43 | 23.94 | 193-199 | + | + | 2 |
| **352** | VPYPQRDMP | 9 | Beta Casein | 2 | 551.77 | 1101.53 | 34.03 | 193-201 | + | + | 2 |
| **353** | VSKVKEA | 7 | Beta Casein | 2 | 380.73 | 759.45 | 12.77 | 110-116 | + |  | 1 |
| **354** | VSKVKEAMAP | 10 | Beta Casein | 2 | 530.30 | 1058.58 | 22.04 | 110-119 | + |  | 1 |
| **355** | VSKVKEAMAPK | 11 | Beta Casein | 3 | 396.57 | 1186.67 | 17.31 | 110-120 | + |  | 1 |
| **356** | VVVPPFLQP | 9 | Beta Casein | 2 | 498.30 | 994.59 | 50.13 | 97-105 | + | + | 2 |
| **357** | VVVPPFLQPE | 10 | Beta Casein | 2 | 562.82 | 1123.63 | 49.58 | 97-106 | + | + | 2 |
| **358** | VVVPPFLQPEVMG | 13 | Beta Casein | 2 | 706.39 | 1410.76 | 51.96 | 97-109 | + | + | 2 |
| **359** | VVVPPFLQPEVMGVSK | 16 | Beta Casein | 2 | 863.48 | 1724.95 | 48.29 | 97-112 | + |  | 1 |
| **360** | WMH | 3 | Beta Casein | 1 | 473.20 | 472.19 | 39.77 | 158-160 | + |  | 1 |
| **361** | WMHQPH | 6 | Beta Casein | 2 | 418.19 | 834.36 | 20.57 | 158-163 | + |  | 1 |
| **362** | WMHQPHQPLPPT | 12 | Beta Casein | 2 | 734.86 | 1467.71 | 32.92 | 158-169 | + |  | 1 |
| **363** | YQEPVLGPVRGPFPIIV | 17 | Beta Casein | 2 | 941.04 | 1880.06 | 50.00 | 208-224 | + | + | 2 |
| **364** | DELQDKIHPFA | 11 | Beta Casein | 2 | 656.83 | 1311.65 | 41.54 | 58-68 |  | + | 1 |
| **365** | DKIHPFAQT | 9 | Beta Casein | 2 | 528.78 | 1055.54 | 28.85 | 62-70 |  | + | 1 |
| **366** | EDE | 3 | Beta Casein | 1 | 392.13 | 391.12 | 46.66 | 57-59 |  | + | 1 |
| **367** | EDELQDKIHPF | 11 | Beta Casein | 2 | 685.83 | 1369.65 | 41.49 | 57-67 |  | + | 1 |
| **368** | EDELQDKIHPFAQTQS | 16 | Beta Casein | 2 | 943.45 | 1884.89 | 40.26 | 57-72 |  | + | 1 |
| **369** | EEL | 3 | Beta Casein | 1 | 390.19 | 389.18 | 52.55 | 19-21 |  | + | 1 |
| **370** | EEQQQTEDELQDKIHPFAQTQS | 22 | Beta Casein | 3 | 877.07 | 2628.19 | 41.31 | 51-72 |  | + | 1 |
| **371** | ELQDKIHPFAQ | 11 | Beta Casein | 2 | 663.35 | 1324.68 | 36.41 | 59-69 |  | + | 1 |
| **372** | EPVLGPVRGPFPI | 13 | Beta Casein | 2 | 689.40 | 1376.78 | 47.00 | 210-222 |  | + | 1 |
| **373** | EQQQTEDELQDKIHPF | 16 | Beta Casein | 3 | 662.31 | 1983.92 | 41.64 | 52-67 |  | + | 1 |
| **374** | HPF | 3 | Beta Casein | 1 | 400.20 | 399.19 | 21.03 | 65-67 |  | + | 1 |
| **375** | HPFAQTQS | 8 | Beta Casein | 2 | 458.22 | 914.42 | 20.35 | 65-72 |  | + | 1 |
| **376** | IPPLTQTPVVVPP | 13 | Beta Casein | 2 | 679.41 | 1356.80 | 47.42 | 89-101 |  | + | 1 |
| **377** | KIEKFQ | 6 | Beta Casein | 2 | 396.73 | 791.45 | 18.44 | 44-49 |  | + | 1 |
| **378** | KVLPVPQKA | 9 | Beta Casein | 2 | 490.32 | 978.62 | 25.85 | 184-192 |  | + | 1 |
| **379** | LHLPLP | 6 | Beta Casein | 1 | 689.43 | 688.43 | 44.32 | 148-153 |  | + | 1 |
| **380** | LHLPLPLL | 8 | Beta Casein | 2 | 458.30 | 914.60 | 49.90 | 148-155 |  | + | 1 |
| **381** | LLYQEPVLGPVRGPFPIIV | 19 | Beta Casein | 2 | 1054.12 | 2106.22 | 51.47 | 206-224 |  | + | 1 |
| **382** | LQDKIHPFAQ | 10 | Beta Casein | 2 | 598.82 | 1195.63 | 32.04 | 60-69 |  | + | 1 |
| **383** | LSLSQSKVLP | 10 | Beta Casein | 2 | 536.32 | 1070.63 | 41.95 | 178-187 |  | + | 1 |
| **384** | LTDVENLHLPLP | 12 | Beta Casein | 2 | 680.88 | 1359.74 | 47.11 | 142-153 |  | + | 1 |
| **385** | PPF | 3 | Beta Casein | 1 | 360.19 | 359.18 | 35.26 | 100-102 |  | + | 1 |
| **386** | PVLGPVRGPFPII | 13 | Beta Casein | 2 | 681.42 | 1360.82 | 48.52 | 211-223 |  | + | 1 |
| **387** | QEPVLGPVRGPFP | 13 | Beta Casein | 2 | 696.89 | 1391.76 | 44.47 | 209-221 |  | + | 1 |
| **388** | QPEVMGVS | 8 | Beta Casein | 1 | 846.40 | 845.40 | 37.55 | 104-111 |  | + | 1 |
| **389** | QQQTEDELQ | 9 | Beta Casein | 2 | 559.75 | 1117.49 | 26.27 | 53-61 |  | + | 1 |
| **390** | QQQTEDELQDK | 11 | Beta Casein | 2 | 681.31 | 1360.61 | 22.16 | 53-63 |  | + | 1 |
| **391** | QTEDELQDK | 9 | Beta Casein | 2 | 553.25 | 1104.49 | 20.92 | 55-63 |  | + | 1 |
| **392** | QTEDELQDKIHPFAQTQS | 18 | Beta Casein | 3 | 705.67 | 2113.99 | 40.04 | 55-72 |  | + | 1 |
| **393** | RELEEL | 6 | Beta Casein | 2 | 394.71 | 787.41 | 32.09 | 16-21 |  | + | 1 |
| **394** | RELEELNVPG | 10 | Beta Casein | 2 | 578.30 | 1154.59 | 39.36 | 16-25 |  | + | 1 |
| **395** | SKVKEAM | 7 | Beta Casein | 2 | 396.72 | 791.42 | 14.87 | 111-117 |  | + | 1 |
| **396** | SSEESITRIN | 10 | Beta Casein | 2 | 568.28 | 1134.55 | 29.11 | 33-42 |  | + | 1 |
| **397** | SSEESITRINK | 11 | Beta Casein | 2 | 632.33 | 1262.65 | 23.72 | 33-43 |  | + | 1 |
| **398** | SSSEESITRIN | 11 | Beta Casein | 2 | 611.80 | 1221.58 | 29.37 | 32-42 |  | + | 1 |
| **399** | VLPVPQKAVPYPQ | 13 | Beta Casein | 2 | 718.42 | 1434.82 | 41.73 | 185-197 |  | + | 1 |
| **400** | VPPFLQPEVMGV | 12 | Beta Casein | 2 | 656.85 | 1311.69 | 50.98 | 99-110 |  | + | 1 |
| **401** | VPYPQRDMPIQA | 12 | Beta Casein | 2 | 707.86 | 1413.71 | 40.21 | 193-204 |  | + | 1 |
| **402** | VRGPFPIIV | 9 | Beta Casein | 2 | 499.31 | 996.61 | 46.35 | 216-224 |  | + | 1 |
| **403** | VSKVKEAM | 8 | Beta Casein | 2 | 446.25 | 890.49 | 17.26 | 110-117 |  | + | 1 |
| **404** | VVPPFLQPE | 9 | Beta Casein | 2 | 513.29 | 1024.56 | 47.34 | 98-106 |  | + | 1 |
| **405** | VVVPPFL | 7 | Beta Casein | 2 | 385.74 | 769.47 | 51.18 | 97-103 |  | + | 1 |
| **406** | YQEPVLGPVRGPFP | 14 | Beta Casein | 2 | 778.42 | 1554.82 | 45.01 | 208-221 |  | + | 1 |
| **407** | YQEPVLGPVRGPFPII | 16 | Beta Casein | 2 | 891.50 | 1780.99 | 49.07 | 208-223 |  | + | 1 |
| **408** | ALKALPMH | 8 | Beta Lactoglobulin | 2 | 440.76 | 879.50 | 29.53 | 155-162 | + | + | 2 |
| **409** | ALPMHIRLS | 9 | Beta Lactoglobulin | 2 | 519.30 | 1036.59 | 40.52 | 158-166 | + | + | 2 |
| **410** | DEALEKFDK | 9 | Beta Lactoglobulin | 2 | 547.77 | 1093.53 | 30.26 | 146-154 | + | + | 2 |
| **411** | DEALEKFDKA | 10 | Beta Lactoglobulin | 2 | 583.29 | 1164.57 | 33.05 | 146-155 | + |  | 1 |
| **412** | FDKALK | 6 | Beta Lactoglobulin | 2 | 361.22 | 720.42 | 18.99 | 152-157 | + |  | 1 |
| **413** | FNPTQLEE | 8 | Beta Lactoglobulin | 2 | 489.23 | 976.45 | 42.67 | 167-174 | + |  | 1 |
| **414** | LKPTPEGDL | 9 | Beta Lactoglobulin | 2 | 485.27 | 968.52 | 32.04 | 62-70 | + |  | 1 |
| **415** | LPMHIRLS | 8 | Beta Lactoglobulin | 2 | 483.78 | 965.55 | 38.38 | 159-166 | + |  | 1 |
| **416** | LVR | 3 | Beta Lactoglobulin | 1 | 387.27 | 386.26 | 27.13 | 138-140 | + |  | 1 |
| **417** | MKGLDIQK | 8 | Beta Lactoglobulin | 2 | 466.77 | 931.52 | 27.30 | 23-30 | + | + | 2 |
| **418** | PMH | 3 | Beta Lactoglobulin | 1 | 384.17 | 383.16 | 25.00 | 160-162 | + | + | 2 |
| **419** | APLRVYVE | 8 | Beta Lactoglobulin | 2 | 473.77 | 945.53 | 37.45 | 53-60 |  | + | 1 |
| **420** | LLF | 3 | Beta Lactoglobulin | 1 | 392.25 | 391.25 | 47.01 | 18-20 |  | + | 1 |
| **421** | QKK | 3 | Beta Lactoglobulin | 1 | 403.27 | 402.26 | 60.36 | 86-88 |  | + | 1 |
| **422** | YKK | 3 | Beta Lactoglobulin | 1 | 438.27 | 437.26 | 36.48 | 115-117 |  | + | 1 |
| **423** | YLL | 3 | Beta Lactoglobulin | 1 | 408.25 | 407.24 | 39.89 | 118-120 |  | + | 1 |
| **424** | AVRSPAQILQ | 10 | Kappa Casein | 2 | 541.82 | 1081.62 | 38.32 | 87-96 | + |  | 1 |
| **425** | AVRSPAQILQWQ | 12 | Kappa Casein | 2 | 698.89 | 1395.76 | 44.48 | 87-98 | + |  | 1 |
| **426** | EINTVQVTSTAV | 12 | Kappa Casein | 2 | 631.34 | 1260.66 | 42.49 | 179-190 | + | + | 2 |
| **427** | EVIESPPEINTVQVTSTAV | 19 | Kappa Casein | 2 | 1007.02 | 2012.03 | 45.69 | 172-190 | + |  | 1 |
| **428** | FFSDKIAK | 8 | Kappa Casein | 2 | 478.27 | 954.52 | 30.55 | 38-45 | + | + | 2 |
| **429** | FFSDKIAKY | 9 | Kappa Casein | 2 | 559.80 | 1117.58 | 37.06 | 38-46 | + | + | 2 |
| **430** | FLPYPYY | 7 | Kappa Casein | 2 | 481.74 | 961.46 | 47.61 | 76-82 | + |  | 1 |
| **431** | FLPYPYYAKPA | 11 | Kappa Casein | 2 | 665.35 | 1328.68 | 43.36 | 76-86 | + |  | 1 |
| **432** | FMAIPPKKN | 9 | Kappa Casein | 2 | 523.30 | 1044.58 | 29.38 | 126-134 | + | + | 2 |
| **433** | FMAIPPKKNQD | 11 | Kappa Casein | 2 | 644.84 | 1287.66 | 29.29 | 126-136 | + |  | 1 |
| **434** | FMAIPPKKNQDKTE | 14 | Kappa Casein | 3 | 554.96 | 1661.84 | 21.13 | 126-139 | + | + | 2 |
| **435** | FSDKIAK | 7 | Kappa Casein | 2 | 404.73 | 807.45 | 21.02 | 39-45 | + |  | 1 |
| **436** | FSDKIAKY | 8 | Kappa Casein | 2 | 486.26 | 970.51 | 27.85 | 39-46 | + | + | 2 |
| **437** | IAKYIPIQY | 9 | Kappa Casein | 2 | 554.82 | 1107.63 | 42.78 | 43-51 | + | + | 2 |
| **438** | IASGEPTSTPTTEA | 14 | Kappa Casein | 2 | 681.33 | 1360.64 | 30.26 | 146-159 | + |  | 1 |
| **439** | IASGEPTSTPTTEAVEST | 18 | Kappa Casein | 2 | 889.42 | 1776.83 | 36.14 | 146-163 | + |  | 1 |
| **440** | IESPPEINT | 9 | Kappa Casein | 2 | 500.25 | 998.49 | 39.10 | 174-182 | + | + | 2 |
| **441** | IESPPEINTVQVTSTAV | 17 | Kappa Casein | 2 | 892.97 | 1783.92 | 44.80 | 174-190 | + | + | 2 |
| **442** | ILQWQVLSNTVPAK | 14 | Kappa Casein | 2 | 798.96 | 1595.90 | 45.09 | 94-107 | + |  | 1 |
| **443** | INTVQVTSTAV | 11 | Kappa Casein | 2 | 566.81 | 1131.61 | 41.55 | 180-190 | + | + | 2 |
| **444** | IPPKKNQDKTE | 11 | Kappa Casein | 3 | 433.24 | 1296.70 | 13.67 | 129-139 | + | + | 2 |
| **445** | IPTINTIASGEPTSTPTTEA | 20 | Kappa Casein | 2 | 1001.00 | 1999.99 | 44.30 | 140-159 | + |  | 1 |
| **446** | KTEIPTIN | 8 | Kappa Casein | 2 | 458.26 | 914.51 | 34.99 | 137-144 | + | + | 2 |
| **447** | LNYYQQKP | 8 | Kappa Casein | 2 | 527.27 | 1052.53 | 28.75 | 61-68 | + | + | 2 |
| **448** | LNYYQQKPVA | 10 | Kappa Casein | 2 | 612.32 | 1222.63 | 31.85 | 61-70 | + | + | 2 |
| **449** | LNYYQQKPVALINNQ | 15 | Kappa Casein | 2 | 903.48 | 1804.95 | 41.88 | 61-75 | + | + | 2 |
| **450** | LPYPYYAKPAA | 11 | Kappa Casein | 2 | 627.33 | 1252.65 | 40.14 | 77-87 | + | + | 2 |
| **451** | QKPVALINNQ | 10 | Kappa Casein | 2 | 562.82 | 1123.63 | 31.61 | 66-75 | + |  | 1 |
| **452** | QQKPVALINNQ | 11 | Kappa Casein | 2 | 626.85 | 1251.69 | 32.18 | 65-75 | + | + | 2 |
| **453** | SDKIAKY | 7 | Kappa Casein | 2 | 412.73 | 823.44 | 18.47 | 40-46 | + | + | 2 |
| **454** | SPPEINT | 7 | Kappa Casein | 2 | 379.19 | 756.37 | 31.24 | 176-182 | + |  | 1 |
| **455** | SPPEINTVQ | 9 | Kappa Casein | 2 | 492.75 | 983.49 | 34.81 | 176-184 | + | + | 2 |
| **456** | SPPEINTVQVTSTAV | 15 | Kappa Casein | 2 | 771.90 | 1541.79 | 43.68 | 176-190 | + | + | 2 |
| **457** | TIASGEPTSTPTTEA | 15 | Kappa Casein | 2 | 731.85 | 1461.68 | 32.83 | 145-159 | + |  | 1 |
| **458** | TIASGEPTSTPTTEAVEST | 19 | Kappa Casein | 2 | 939.94 | 1877.87 | 37.98 | 145-163 | + |  | 1 |
| **459** | VIESPPEIN | 9 | Kappa Casein | 2 | 499.26 | 996.51 | 40.03 | 173-181 | + |  | 1 |
| **460** | VIESPPEINT | 10 | Kappa Casein | 2 | 549.79 | 1097.56 | 41.38 | 173-182 | + | + | 2 |
| **461** | VIESPPEINTVQ | 12 | Kappa Casein | 2 | 663.35 | 1324.69 | 42.38 | 173-184 | + |  | 1 |
| **462** | VIESPPEINTVQVTSTAV | 18 | Kappa Casein | 2 | 942.50 | 1882.99 | 45.21 | 173-190 | + | + | 2 |
| **463** | VLSNTVPAK | 9 | Kappa Casein | 2 | 464.78 | 927.54 | 24.98 | 99-107 | + | + | 2 |
| **464** | VLSNTVPAKS | 10 | Kappa Casein | 2 | 508.29 | 1014.57 | 25.00 | 99-108 | + |  | 1 |
| **465** | VLSRYPS | 7 | Kappa Casein | 2 | 411.23 | 820.44 | 25.45 | 52-58 | + | + | 2 |
| **466** | VLSRYPSYG | 9 | Kappa Casein | 2 | 521.27 | 1040.53 | 31.56 | 52-60 | + | + | 2 |
| **467** | VLSRYPSYGLN | 11 | Kappa Casein | 2 | 634.84 | 1267.66 | 38.58 | 52-62 | + | + | 2 |
| **468** | VLSRYPSYGLNY | 12 | Kappa Casein | 2 | 716.37 | 1430.72 | 42.18 | 52-63 | + | + | 2 |
| **469** | VRSPAQILQ | 9 | Kappa Casein | 2 | 506.30 | 1010.59 | 37.25 | 88-96 | + | + | 2 |
| **470** | VRSPAQILQW | 10 | Kappa Casein | 2 | 599.34 | 1196.67 | 45.21 | 88-97 | + |  | 1 |
| **471** | VRSPAQILQWQ | 11 | Kappa Casein | 2 | 663.37 | 1324.73 | 44.30 | 88-98 | + | + | 2 |
| **472** | WQVLSNTVPAK | 11 | Kappa Casein | 2 | 621.85 | 1241.68 | 41.49 | 97-107 | + | + | 2 |
| **473** | WQVLSNTVPAKS | 12 | Kappa Casein | 2 | 665.36 | 1328.71 | 41.44 | 97-108 | + | + | 2 |
| **474** | YGLNY | 5 | Kappa Casein | 1 | 629.29 | 628.29 | 40.49 | 59-63 | + |  | 1 |
| **475** | YGLNYYQQKPVA | 12 | Kappa Casein | 2 | 722.37 | 1442.72 | 38.59 | 59-70 | + |  | 1 |
| **476** | YGLNYYQQKPVALINNQ | 17 | Kappa Casein | 2 | 1013.52 | 2025.03 | 43.31 | 59-75 | + |  | 1 |
| **477** | YQQKPVAL | 8 | Kappa Casein | 2 | 473.77 | 945.53 | 31.24 | 64-71 | + |  | 1 |
| **478** | YQQKPVALINNQ | 12 | Kappa Casein | 2 | 708.39 | 1414.76 | 34.86 | 64-75 | + | + | 2 |
| **479** | YYQQKPVALINNQ | 13 | Kappa Casein | 2 | 789.92 | 1577.82 | 37.53 | 63-75 | + | + | 2 |
| **480** | FFL | 3 | Kappa Casein | 1 | 426.24 | 425.23 | 14.54 | 5-7 |  | + | 1 |
| **481** | FLPYPYYAKPAA | 12 | Kappa Casein | 2 | 700.87 | 1399.72 | 43.24 | 76-87 |  | + | 1 |
| **482** | IPTINTIASGEPTSTPTTEAVEST | 24 | Kappa Casein | 2 | 1209.10 | 2416.19 | 44.74 | 140-163 |  | + | 1 |
| **483** | LPYPYYAKPA | 10 | Kappa Casein | 2 | 591.81 | 1181.61 | 39.57 | 77-86 |  | + | 1 |
| **484** | PKK | 3 | Kappa Casein | 1 | 372.26 | 371.25 | 64.07 | 131-133 |  | + | 1 |
| **485** | QVLSNTVPAK | 10 | Kappa Casein | 2 | 528.81 | 1055.60 | 27.84 | 98-107 |  | + | 1 |
| **486** | SPPEINTVQVT | 11 | Kappa Casein | 2 | 592.81 | 1183.61 | 41.67 | 176-186 |  | + | 1 |
| **487** | VLSRYPSY | 8 | Kappa Casein | 2 | 492.76 | 983.51 | 32.55 | 52-59 |  | + | 1 |
| **488** | VRS | 3 | Kappa Casein | 1 | 361.22 | 360.21 | 59.85 | 88-90 |  | + | 1 |
| **489** | YYQQKPVA | 8 | Kappa Casein | 2 | 498.76 | 995.51 | 22.77 | 63-70 |  | + | 1 |

**Tablo 11.** Milk peptides identified throughout the 28 days of storage period using the *Lb. coryniformis* NCCB 100543.

|  | **Sequence** | **Length** | **Leading proteins** | **Charge** | **m/z** | **Mass** | **Retention time** | **Sequence** | **Day 1** | **Day 28** | **Incidence** |
| --- | --- | --- | --- | --- | --- | --- | --- | --- | --- | --- | --- |
| **1** | QKEPMIGVNQELAY | 14 | Alpha S1 Casein | 2 | 810.41 | 1618.80 | 42.71 | 146-159 | + |  | 1 |
| **2** | VAPFPEVFGKEKV | 13 | Alpha S1 Casein | 2 | 723.90 | 1445.79 | 43.12 | 40-52 | + | + | 2 |
| **3** | APFPEVFGKE | 10 | Alpha S1 Casein | 2 | 560.79 | 1119.56 | 43.97 | 41-50 |  |  | 1 |
| **4** | APFPEVFGKEKVNE | 14 | Alpha S1 Casein | 3 | 530.94 | 1589.81 | 41.72 | 41-54 |  | + | 1 |
| **5** | AQQKEPMIG | 9 | Alpha S1 Casein | 2 | 501.26 | 1000.50 | 27.09 | 144-152 |  | + | 1 |
| **6** | AQQKEPMIGVNQEL | 14 | Alpha S1 Casein | 2 | 792.91 | 1583.80 | 41.61 | 144-157 |  | + | 1 |
| **7** | DIKQMEAE | 8 | Alpha S1 Casein | 2 | 482.23 | 962.44 | 29.08 | 71-78 |  | + | 1 |
| **8** | DIPNPIGSENSE | 12 | Alpha S1 Casein | 2 | 636.29 | 1270.57 | 43.26 | 196-207 |  | + | 1 |
| **9** | DVPSERY | 7 | Alpha S1 Casein | 2 | 433.21 | 864.40 | 26.34 | 100-106 |  | + | 1 |
| **10** | DVPSERYL | 8 | Alpha S1 Casein | 2 | 489.75 | 977.48 | 38.54 | 100-107 |  | + | 1 |
| **11** | DVPSERYLG | 9 | Alpha S1 Casein | 2 | 518.26 | 1034.50 | 36.04 | 100-108 |  | + | 1 |
| **12** | EDVPSER | 7 | Alpha S1 Casein | 2 | 416.20 | 830.38 | 19.48 | 99-105 |  | + | 1 |
| **13** | EDVPSERY | 8 | Alpha S1 Casein | 2 | 497.73 | 993.44 | 27.11 | 99-106 |  | + | 1 |
| **14** | EDVPSERYL | 9 | Alpha S1 Casein | 2 | 554.27 | 1106.52 | 37.94 | 99-107 |  | + | 1 |
| **15** | EDVPSERYLG | 10 | Alpha S1 Casein | 2 | 582.78 | 1163.55 | 35.70 | 99-108 |  | + | 1 |
| **16** | EDVPSERYLGYLEQ | 14 | Alpha S1 Casein | 2 | 849.40 | 1696.79 | 44.14 | 99-112 |  | + | 1 |
| **17** | EGIHAQQ | 7 | Alpha S1 Casein | 2 | 391.69 | 781.37 | 16.95 | 140-146 |  | + | 1 |
| **18** | EGIHAQQK | 8 | Alpha S1 Casein | 2 | 455.74 | 909.47 | 13.93 | 140-147 |  | + | 1 |
| **19** | ENLLRF | 6 | Alpha S1 Casein | 2 | 396.22 | 790.43 | 43.69 | 33-38 |  | + | 1 |
| **20** | EPMIGVNQELAY | 12 | Alpha S1 Casein | 2 | 682.33 | 1362.65 | 45.78 | 148-159 |  | + | 1 |
| **21** | EVFGKEKVN | 9 | Alpha S1 Casein | 2 | 525.28 | 1048.56 | 23.10 | 45-53 |  | + | 1 |
| **22** | EVFGKEKVNE | 10 | Alpha S1 Casein | 2 | 589.81 | 1177.60 | 24.64 | 45-54 |  | + | 1 |
| **23** | EVFGKEKVNEL | 11 | Alpha S1 Casein | 2 | 646.35 | 1290.68 | 34.46 | 45-55 |  | + | 1 |
| **24** | EVLNENLLR | 9 | Alpha S1 Casein | 2 | 550.31 | 1098.60 | 41.93 | 29-37 |  | + | 1 |
| **25** | FFVAPFPEVFG | 11 | Alpha S1 Casein | 2 | 628.82 | 1255.63 | 55.02 | 38-48 |  | + | 1 |
| **26** | FFVAPFPEVFGK | 12 | Alpha S1 Casein | 2 | 692.87 | 1383.72 | 49.81 | 38-49 |  | + | 1 |
| **27** | FFVAPFPEVFGKE | 13 | Alpha S1 Casein | 2 | 757.39 | 1512.77 | 49.81 | 38-50 |  | + | 1 |
| **28** | FFVAPFPEVFGKEK | 14 | Alpha S1 Casein | 3 | 547.96 | 1640.86 | 46.53 | 38-51 |  | + | 1 |
| **29** | FPEVFGK | 7 | Alpha S1 Casein | 2 | 412.22 | 822.43 | 42.29 | 43-49 |  | + | 1 |
| **30** | FPEVFGKE | 8 | Alpha S1 Casein | 2 | 476.74 | 951.47 | 42.46 | 43-50 |  | + | 1 |
| **31** | FPEVFGKEK | 9 | Alpha S1 Casein | 2 | 540.79 | 1079.57 | 36.29 | 43-51 |  | + | 1 |
| **32** | FPEVFGKEKV | 10 | Alpha S1 Casein | 2 | 590.32 | 1178.63 | 40.46 | 43-52 |  | + | 1 |
| **33** | FPEVFGKEKVN | 11 | Alpha S1 Casein | 2 | 647.35 | 1292.68 | 38.35 | 43-53 |  | + | 1 |
| **34** | FPEVFGKEKVNE | 12 | Alpha S1 Casein | 2 | 711.87 | 1421.72 | 38.65 | 43-54 |  | + | 1 |
| **35** | FPEVFGKEKVNEL | 13 | Alpha S1 Casein | 2 | 768.41 | 1534.80 | 42.62 | 43-55 |  | + | 1 |
| **36** | FPEVFGKEKVNELS | 14 | Alpha S1 Casein | 2 | 811.92 | 1621.84 | 41.99 | 43-56 |  | + | 1 |
| **37** | FSDIPNPIGSEN | 12 | Alpha S1 Casein | 2 | 645.30 | 1288.59 | 44.53 | 194-205 |  | + | 1 |
| **38** | FSDIPNPIGSENSE | 14 | Alpha S1 Casein | 2 | 753.34 | 1504.67 | 44.44 | 194-207 |  | + | 1 |
| **39** | FSDIPNPIGSENSEK | 15 | Alpha S1 Casein | 2 | 817.39 | 1632.76 | 41.83 | 194-208 |  | + | 1 |
| **40** | FSDIPNPIGSENSEKT | 16 | Alpha S1 Casein | 2 | 867.91 | 1733.81 | 41.95 | 194-209 |  | + | 1 |
| **41** | FSDIPNPIGSENSEKTT | 17 | Alpha S1 Casein | 2 | 918.44 | 1834.86 | 41.93 | 194-210 |  | + | 1 |
| **42** | FSDIPNPIGSENSEKTTMPL | 20 | Alpha S1 Casein | 2 | 1089.03 | 2176.04 | 45.00 | 194-213 |  | + | 1 |
| **43** | FSDIPNPIGSENSEKTTMPLW | 21 | Alpha S1 Casein | 2 | 1190.06 | 2378.11 | 45.96 | 194-214 |  | + | 1 |
| **44** | FVAPFPEVFG | 10 | Alpha S1 Casein | 2 | 555.29 | 1108.56 | 52.59 | 39-48 |  | + | 1 |
| **45** | FVAPFPEVFGK | 11 | Alpha S1 Casein | 2 | 619.33 | 1236.65 | 47.32 | 39-49 |  | + | 1 |
| **46** | FVAPFPEVFGKE | 12 | Alpha S1 Casein | 2 | 683.86 | 1365.70 | 47.36 | 39-50 |  | + | 1 |
| **47** | FVAPFPEVFGKEK | 13 | Alpha S1 Casein | 2 | 747.90 | 1493.79 | 44.55 | 39-51 |  | + | 1 |
| **48** | FVAPFPEVFGKEKV | 14 | Alpha S1 Casein | 3 | 531.96 | 1592.86 | 45.04 | 39-52 |  | + | 1 |
| **49** | GKEKVNELS | 9 | Alpha S1 Casein | 2 | 502.27 | 1002.53 | 17.42 | 48-56 |  | + | 1 |
| **50** | GLPQEVLNENL | 11 | Alpha S1 Casein | 2 | 613.32 | 1224.64 | 46.18 | 25-35 |  | + | 1 |
| **51** | GLPQEVLNENLL | 12 | Alpha S1 Casein | 2 | 669.87 | 1337.72 | 49.18 | 25-36 |  | + | 1 |
| **52** | GLPQEVLNENLLR | 13 | Alpha S1 Casein | 2 | 747.92 | 1493.82 | 45.22 | 25-37 |  | + | 1 |
| **53** | GYL | 3 | Alpha S1 Casein | 1 | 352.19 | 351.18 | 33.80 | 108-110 |  | + | 1 |
| **54** | HIQKEDVPS | 9 | Alpha S1 Casein | 2 | 526.77 | 1051.53 | 18.97 | 95-103 |  | + | 1 |
| **55** | HIQKEDVPSE | 10 | Alpha S1 Casein | 2 | 591.29 | 1180.57 | 20.41 | 95-104 |  | + | 1 |
| **56** | HQGLPQEVLNEN | 12 | Alpha S1 Casein | 2 | 689.34 | 1376.67 | 38.13 | 23-34 |  | + | 1 |
| **57** | HQGLPQEVLNENL | 13 | Alpha S1 Casein | 2 | 745.88 | 1489.75 | 43.33 | 23-35 |  | + | 1 |
| **58** | HQGLPQEVLNENLL | 14 | Alpha S1 Casein | 2 | 802.43 | 1602.84 | 45.34 | 23-36 |  | + | 1 |
| **59** | IPNPIGSENSE | 11 | Alpha S1 Casein | 2 | 578.78 | 1155.54 | 40.06 | 197-207 |  | + | 1 |
| **60** | IPNPIGSENSEK | 12 | Alpha S1 Casein | 2 | 642.83 | 1283.64 | 32.82 | 197-208 |  | + | 1 |
| **61** | IPNPIGSENSEKTT | 14 | Alpha S1 Casein | 2 | 743.87 | 1485.73 | 33.68 | 197-210 |  | + | 1 |
| **62** | IPNPIGSENSEKTTMP | 16 | Alpha S1 Casein | 2 | 857.92 | 1713.82 | 40.97 | 197-212 |  | + | 1 |
| **63** | IPNPIGSENSEKTTMPL | 17 | Alpha S1 Casein | 2 | 914.46 | 1826.91 | 43.31 | 197-213 |  | + | 1 |
| **64** | IPNPIGSENSEKTTMPLW | 18 | Alpha S1 Casein | 2 | 1015.50 | 2028.98 | 44.39 | 197-214 |  | + | 1 |
| **65** | IQKEDVPSE | 9 | Alpha S1 Casein | 2 | 522.76 | 1043.51 | 23.81 | 96-104 |  | + | 1 |
| **66** | KEDVPSE | 7 | Alpha S1 Casein | 2 | 402.19 | 802.37 | 19.23 | 98-104 |  | + | 1 |
| **67** | KEDVPSER | 8 | Alpha S1 Casein | 2 | 480.24 | 958.47 | 16.17 | 98-105 |  | + | 1 |
| **68** | KEDVPSERY | 9 | Alpha S1 Casein | 2 | 561.77 | 1121.54 | 22.46 | 98-106 |  | + | 1 |
| **69** | KEDVPSERYL | 10 | Alpha S1 Casein | 2 | 618.32 | 1234.62 | 31.75 | 98-107 |  | + | 1 |
| **70** | KEDVPSERYLG | 11 | Alpha S1 Casein | 2 | 646.83 | 1291.64 | 30.13 | 98-108 |  | + | 1 |
| **71** | KEDVPSERYLGY | 12 | Alpha S1 Casein | 2 | 728.36 | 1454.70 | 36.02 | 98-109 |  | + | 1 |
| **72** | KEDVPSERYLGYLEQ | 15 | Alpha S1 Casein | 3 | 609.30 | 1824.89 | 42.24 | 98-112 |  | + | 1 |
| **73** | KEGIHAQQ | 8 | Alpha S1 Casein | 2 | 455.74 | 909.47 | 13.93 | 139-146 |  | + | 1 |
| **74** | KEKVNEL | 7 | Alpha S1 Casein | 2 | 430.25 | 858.48 | 17.93 | 49-55 |  | + | 1 |
| **75** | KEKVNELS | 8 | Alpha S1 Casein | 2 | 473.76 | 945.51 | 16.86 | 49-56 |  | + | 1 |
| **76** | KEPMIGVNQ | 9 | Alpha S1 Casein | 2 | 508.27 | 1014.52 | 31.33 | 147-155 |  | + | 1 |
| **77** | KEPMIGVNQE | 10 | Alpha S1 Casein | 2 | 572.79 | 1143.56 | 32.02 | 147-156 |  | + | 1 |
| **78** | KEPMIGVNQEL | 11 | Alpha S1 Casein | 2 | 629.33 | 1256.64 | 41.61 | 147-157 |  | + | 1 |
| **79** | KEPMIGVNQELA | 12 | Alpha S1 Casein | 2 | 664.85 | 1327.68 | 41.00 | 147-158 |  | + | 1 |
| **80** | KEPMIGVNQELAY | 13 | Alpha S1 Casein | 2 | 746.38 | 1490.74 | 42.74 | 147-159 |  | + | 1 |
| **81** | KHIQKEDVPSE | 11 | Alpha S1 Casein | 2 | 655.34 | 1308.67 | 17.64 | 94-104 |  | + | 1 |
| **82** | KTTMPLW | 7 | Alpha S1 Casein | 2 | 438.74 | 875.46 | 43.39 | 208-214 |  | + | 1 |
| **83** | KVPQLEIVP | 9 | Alpha S1 Casein | 2 | 511.82 | 1021.62 | 43.73 | 120-128 |  | + | 1 |
| **84** | KVPQLEIVPN | 10 | Alpha S1 Casein | 2 | 568.84 | 1135.66 | 42.62 | 120-129 |  | + | 1 |
| **85** | KVPQLEIVPNSAEE | 14 | Alpha S1 Casein | 2 | 776.91 | 1551.81 | 42.68 | 120-133 |  | + | 1 |
| **86** | KYKVPQL | 7 | Alpha S1 Casein | 2 | 438.27 | 874.53 | 32.26 | 118-124 |  | + | 1 |
| **87** | LEIVPNSAEERLHSM | 15 | Alpha S1 Casein | 3 | 575.63 | 1723.86 | 41.86 | 124-138 |  | + | 1 |
| **88** | LGYLEQLLR | 9 | Alpha S1 Casein | 2 | 552.82 | 1103.63 | 46.45 | 107-115 |  | + | 1 |
| **89** | LNENLLR | 7 | Alpha S1 Casein | 2 | 436.25 | 870.49 | 35.17 | 31-37 |  | + | 1 |
| **90** | MKEGIHA | 7 | Alpha S1 Casein | 2 | 393.20 | 784.39 | 18.28 | 138-144 |  | + | 1 |
| **91** | MKEGIHAQQ | 9 | Alpha S1 Casein | 2 | 521.26 | 1040.51 | 17.00 | 138-146 |  | + | 1 |
| **92** | NPIGSENSE | 9 | Alpha S1 Casein | 2 | 473.71 | 945.40 | 24.27 | 199-207 |  | + | 1 |
| **93** | NPIGSENSEK | 10 | Alpha S1 Casein | 2 | 537.76 | 1073.50 | 18.48 | 199-208 |  | + | 1 |
| **94** | NPIGSENSEKTT | 12 | Alpha S1 Casein | 2 | 638.80 | 1275.59 | 20.47 | 199-210 |  | + | 1 |
| **95** | PIGSENSEKTT | 11 | Alpha S1 Casein | 2 | 581.78 | 1161.55 | 17.90 | 200-210 |  | + | 1 |
| **96** | PIGSENSEKTTMP | 13 | Alpha S1 Casein | 2 | 695.83 | 1389.64 | 29.81 | 200-212 |  | + | 1 |
| **97** | PIGSENSEKTTMPLW | 15 | Alpha S1 Casein | 2 | 845.41 | 1688.81 | 44.55 | 200-214 |  | + | 1 |
| **98** | QEVLNENL | 8 | Alpha S1 Casein | 2 | 479.75 | 957.48 | 42.29 | 28-35 |  | + | 1 |
| **99** | QEVLNENLL | 9 | Alpha S1 Casein | 2 | 536.29 | 1070.56 | 45.85 | 28-36 |  | + | 1 |
| **100** | QEVLNENLLR | 10 | Alpha S1 Casein | 2 | 614.34 | 1226.66 | 42.05 | 28-37 |  | + | 1 |
| **101** | QKEPMIGVN | 9 | Alpha S1 Casein | 2 | 508.27 | 1014.52 | 31.98 | 146-154 |  | + | 1 |
| **102** | QKEPMIGVNQE | 11 | Alpha S1 Casein | 2 | 636.82 | 1271.62 | 32.23 | 146-156 |  | + | 1 |
| **103** | QKEPMIGVNQEL | 12 | Alpha S1 Casein | 2 | 693.36 | 1384.70 | 41.61 | 146-157 |  | + | 1 |
| **104** | SDIPNPIGSENSE | 13 | Alpha S1 Casein | 2 | 679.81 | 1357.60 | 42.34 | 195-207 |  | + | 1 |
| **105** | SDIPNPIGSENSEK | 14 | Alpha S1 Casein | 2 | 743.85 | 1485.69 | 36.66 | 195-208 |  | + | 1 |
| **106** | SDIPNPIGSENSEKTTMPLW | 20 | Alpha S1 Casein | 2 | 1108.53 | 2215.05 | 46.60 | 195-214 |  | + | 1 |
| **107** | SEKTTMPLW | 9 | Alpha S1 Casein | 2 | 546.77 | 1091.53 | 43.73 | 206-214 |  | + | 1 |
| **108** | SMKEGIHA | 8 | Alpha S1 Casein | 2 | 436.72 | 871.42 | 19.46 | 137-144 |  | + | 1 |
| **109** | SMKEGIHAQ | 9 | Alpha S1 Casein | 2 | 500.75 | 999.48 | 17.71 | 137-145 |  | + | 1 |
| **110** | SMKEGIHAQQ | 10 | Alpha S1 Casein | 2 | 564.78 | 1127.54 | 17.99 | 137-146 |  | + | 1 |
| **111** | VAPFPEVFG | 9 | Alpha S1 Casein | 2 | 481.75 | 961.49 | 49.76 | 40-48 |  | + | 1 |
| **112** | VAPFPEVFGK | 10 | Alpha S1 Casein | 2 | 545.80 | 1089.59 | 44.84 | 40-49 |  | + | 1 |
| **113** | VAPFPEVFGKE | 11 | Alpha S1 Casein | 2 | 610.32 | 1218.63 | 44.89 | 40-50 |  | + | 1 |
| **114** | VAPFPEVFGKEK | 12 | Alpha S1 Casein | 2 | 674.37 | 1346.72 | 42.29 | 40-51 |  | + | 1 |
| **115** | VAPFPEVFGKEKVN | 14 | Alpha S1 Casein | 2 | 780.92 | 1559.83 | 42.62 | 40-53 |  | + | 1 |
| **116** | VAPFPEVFGKEKVNE | 15 | Alpha S1 Casein | 3 | 563.97 | 1688.88 | 42.68 | 40-54 |  | + | 1 |
| **117** | VAPFPEVFGKEKVNEL | 16 | Alpha S1 Casein | 3 | 601.66 | 1801.96 | 44.22 | 40-55 |  | + | 1 |
| **118** | VAPFPEVFGKEKVNELS | 17 | Alpha S1 Casein | 2 | 945.50 | 1888.99 | 43.69 | 40-56 |  | + | 1 |
| **119** | VFGKEKVN | 8 | Alpha S1 Casein | 2 | 460.76 | 919.51 | 19.27 | 46-53 |  | + | 1 |
| **120** | VFGKEKVNE | 9 | Alpha S1 Casein | 2 | 525.28 | 1048.56 | 20.85 | 46-54 |  | + | 1 |
| **121** | VFGKEKVNEL | 10 | Alpha S1 Casein | 2 | 581.83 | 1161.64 | 30.95 | 46-55 |  | + | 1 |
| **122** | VFGKEKVNELS | 11 | Alpha S1 Casein | 2 | 625.34 | 1248.67 | 29.12 | 46-56 |  | + | 1 |
| **123** | VLNENLLR | 8 | Alpha S1 Casein | 2 | 485.79 | 969.56 | 38.88 | 30-37 |  | + | 1 |
| **124** | VPSERYL | 7 | Alpha S1 Casein | 2 | 432.23 | 862.45 | 32.28 | 101-107 |  | + | 1 |
| **125** | VPSERYLG | 8 | Alpha S1 Casein | 2 | 460.75 | 919.48 | 30.23 | 101-108 |  | + | 1 |
| **126** | VPSERYLGY | 9 | Alpha S1 Casein | 2 | 542.28 | 1082.54 | 37.34 | 101-109 |  | + | 1 |
| **127** | VPSERYLGYLEQ | 12 | Alpha S1 Casein | 2 | 727.37 | 1452.72 | 42.79 | 101-112 |  | + | 1 |
| **128** | YKVPQLE | 7 | Alpha S1 Casein | 2 | 438.74 | 875.48 | 37.31 | 119-125 |  | + | 1 |
| **129** | YKVPQLEIVPN | 11 | Alpha S1 Casein | 2 | 650.37 | 1298.72 | 43.97 | 119-129 |  | + | 1 |
| **130** | YKVPQLEIVPNSAEE | 15 | Alpha S1 Casein | 2 | 858.45 | 1714.88 | 43.90 | 119-133 |  | + | 1 |
| **131** | YVP | 3 | Alpha S1 Casein | 1 | 378.20 | 377.20 | 18.10 | 181-183 |  | + | 1 |
| **132** | YVPLGTQYTDAPS | 13 | Alpha S1 Casein | 2 | 706.34 | 1410.67 | 42.87 | 181-193 |  | + | 1 |
| **133** | RFF | 3 | Alpha S1 Casein | 1 | 469.26 | 468.25 | 40.94 | 37-39 |  | + | 1 |
| **134** | AINPSKEN | 8 | Alpha S2 Casein | 2 | 436.73 | 871.44 | 16.78 | 42-49 |  | + | 1 |
| **135** | ALPQYLK | 7 | Alpha S2 Casein | 2 | 416.75 | 831.49 | 36.30 | 190-196 |  | + | 1 |
| **136** | ALPQYLKTVY | 10 | Alpha S2 Casein | 2 | 598.34 | 1194.66 | 43.45 | 190-199 |  | + | 1 |
| **137** | ALPQYLKTVYQ | 11 | Alpha S2 Casein | 2 | 662.37 | 1322.72 | 43.08 | 190-200 |  | + | 1 |
| **138** | ALPQYLKTVYQH | 12 | Alpha S2 Casein | 2 | 730.90 | 1459.78 | 41.61 | 190-201 |  | + | 1 |
| **139** | ALPQYLKTVYQHQ | 13 | Alpha S2 Casein | 2 | 794.93 | 1587.84 | 41.56 | 190-202 |  | + | 1 |
| **140** | AMKPWIQP | 8 | Alpha S2 Casein | 2 | 485.76 | 969.51 | 41.89 | 204-211 |  | + | 1 |
| **141** | AMKPWIQPK | 9 | Alpha S2 Casein | 2 | 549.81 | 1097.61 | 32.08 | 204-212 |  | + | 1 |
| **142** | AMKPWIQPKT | 10 | Alpha S2 Casein | 3 | 400.56 | 1198.65 | 33.42 | 204-213 |  | + | 1 |
| **143** | AVPITPTLNR | 10 | Alpha S2 Casein | 2 | 541.32 | 1080.63 | 37.77 | 131-140 |  | + | 1 |
| **144** | DMESTEVFTKK | 11 | Alpha S2 Casein | 2 | 657.82 | 1313.62 | 32.14 | 155-165 |  | + | 1 |
| **145** | EEEKNRLNFL | 10 | Alpha S2 Casein | 2 | 646.34 | 1290.66 | 40.40 | 170-179 |  | + | 1 |
| **146** | EEKNRLNF | 8 | Alpha S2 Casein | 2 | 525.27 | 1048.53 | 28.42 | 171-178 |  | + | 1 |
| **147** | EEY | 3 | Alpha S2 Casein | 1 | 440.17 | 439.16 | 42.00 | 65-67 |  | + | 1 |
| **148** | EKNRLNF | 7 | Alpha S2 Casein | 2 | 460.75 | 919.49 | 26.93 | 172-178 |  | + | 1 |
| **149** | EKNRLNFL | 8 | Alpha S2 Casein | 2 | 517.29 | 1032.57 | 38.34 | 172-179 |  | + | 1 |
| **150** | ESTEVFT | 7 | Alpha S2 Casein | 1 | 812.37 | 811.36 | 36.91 | 157-163 |  | + | 1 |
| **151** | FALPQYLK | 8 | Alpha S2 Casein | 2 | 490.28 | 978.55 | 44.03 | 189-196 |  | + | 1 |
| **152** | FALPQYLKT | 9 | Alpha S2 Casein | 2 | 540.81 | 1079.60 | 44.33 | 189-197 |  | + | 1 |
| **153** | FALPQYLKTVYQ | 12 | Alpha S2 Casein | 2 | 735.90 | 1469.79 | 46.04 | 189-200 |  | + | 1 |
| **154** | FALPQYLKTVYQH | 13 | Alpha S2 Casein | 3 | 536.62 | 1606.85 | 44.53 | 189-201 |  | + | 1 |
| **155** | FLKKISQ | 7 | Alpha S2 Casein | 2 | 432.27 | 862.53 | 22.61 | 178-184 |  | + | 1 |
| **156** | GPIVLNPWDQVK | 12 | Alpha S2 Casein | 2 | 683.38 | 1364.75 | 45.04 | 117-128 |  | + | 1 |
| **157** | IISQETYKQE | 10 | Alpha S2 Casein | 2 | 619.82 | 1237.62 | 26.29 | 29-38 |  | + | 1 |
| **158** | IQPKTKVIP | 9 | Alpha S2 Casein | 2 | 512.33 | 1022.65 | 26.82 | 209-217 |  | + | 1 |
| **159** | IQPKTKVIPY | 10 | Alpha S2 Casein | 2 | 593.86 | 1185.71 | 31.88 | 209-218 |  | + | 1 |
| **160** | ISQRYQ | 6 | Alpha S2 Casein | 2 | 397.71 | 793.41 | 17.67 | 182-187 |  | + | 1 |
| **161** | ISQRYQK | 7 | Alpha S2 Casein | 2 | 461.76 | 921.50 | 14.51 | 182-188 |  | + | 1 |
| **162** | ITPTLNR | 7 | Alpha S2 Casein | 2 | 407.74 | 813.47 | 27.41 | 134-140 |  | + | 1 |
| **163** | IVLNPWDQVK | 10 | Alpha S2 Casein | 2 | 606.34 | 1210.67 | 44.17 | 119-128 |  | + | 1 |
| **164** | KFALPQY | 7 | Alpha S2 Casein | 2 | 433.74 | 865.47 | 42.19 | 188-194 |  | + | 1 |
| **165** | KFALPQYL | 8 | Alpha S2 Casein | 2 | 490.28 | 978.55 | 45.10 | 188-195 |  | + | 1 |
| **166** | KFALPQYLK | 9 | Alpha S2 Casein | 2 | 554.33 | 1106.65 | 41.77 | 188-196 |  | + | 1 |
| **167** | KFALPQYLKT | 10 | Alpha S2 Casein | 2 | 604.86 | 1207.70 | 42.19 | 188-197 |  | + | 1 |
| **168** | KISQRYQ | 7 | Alpha S2 Casein | 2 | 461.76 | 921.50 | 15.21 | 181-187 |  | + | 1 |
| **169** | KKTKLTEEE | 9 | Alpha S2 Casein | 2 | 553.31 | 1104.60 | 14.33 | 164-172 |  | + | 1 |
| **170** | KNRLNF | 6 | Alpha S2 Casein | 2 | 396.23 | 790.44 | 24.55 | 173-178 |  | + | 1 |
| **171** | KNRLNFL | 7 | Alpha S2 Casein | 2 | 452.77 | 903.53 | 35.85 | 173-179 |  | + | 1 |
| **172** | KPWIQPKT | 8 | Alpha S2 Casein | 2 | 499.30 | 996.58 | 26.81 | 206-213 |  | + | 1 |
| **173** | KTKLTEEE | 8 | Alpha S2 Casein | 2 | 489.26 | 976.51 | 15.95 | 165-172 |  | + | 1 |
| **174** | KTKLTEEEK | 9 | Alpha S2 Casein | 2 | 553.31 | 1104.60 | 14.33 | 165-173 |  | + | 1 |
| **175** | KTVDMESTEVFT | 12 | Alpha S2 Casein | 2 | 693.83 | 1385.64 | 41.18 | 152-163 |  | + | 1 |
| **176** | KTVYQHQ | 7 | Alpha S2 Casein | 2 | 452.24 | 902.46 | 14.04 | 196-202 |  | + | 1 |
| **177** | LPQYLKTVYQ | 10 | Alpha S2 Casein | 2 | 626.85 | 1251.69 | 42.33 | 191-200 |  | + | 1 |
| **178** | LTEEEKNRLN | 10 | Alpha S2 Casein | 2 | 623.33 | 1244.64 | 19.93 | 168-177 |  | + | 1 |
| **179** | LTEEEKNRLNF | 11 | Alpha S2 Casein | 2 | 696.86 | 1391.70 | 33.31 | 168-178 |  | + | 1 |
| **180** | LTEEEKNRLNFL | 12 | Alpha S2 Casein | 2 | 753.40 | 1504.79 | 41.61 | 168-179 |  | + | 1 |
| **181** | LYQGPIVLNPWDQVK | 15 | Alpha S2 Casein | 2 | 885.48 | 1768.95 | 46.74 | 114-128 |  | + | 1 |
| **182** | MKPWIQP | 7 | Alpha S2 Casein | 2 | 450.24 | 898.47 | 41.78 | 205-211 |  | + | 1 |
| **183** | MKPWIQPKT | 9 | Alpha S2 Casein | 2 | 564.82 | 1127.62 | 32.76 | 205-213 |  | + | 1 |
| **184** | NAVPITPTLN | 10 | Alpha S2 Casein | 2 | 520.29 | 1038.57 | 43.49 | 130-139 |  | + | 1 |
| **185** | NAVPITPTLNR | 11 | Alpha S2 Casein | 2 | 598.34 | 1194.67 | 38.65 | 130-140 |  | + | 1 |
| **186** | NAVPITPTLNRE | 12 | Alpha S2 Casein | 2 | 662.86 | 1323.71 | 39.01 | 130-141 |  | + | 1 |
| **187** | NAVPITPTLNREQL | 14 | Alpha S2 Casein | 2 | 783.44 | 1564.86 | 42.69 | 130-143 |  | + | 1 |
| **188** | PWDQVK | 6 | Alpha S2 Casein | 2 | 386.70 | 771.39 | 27.34 | 123-128 |  | + | 1 |
| **189** | PWIQPKT | 7 | Alpha S2 Casein | 2 | 435.25 | 868.48 | 33.93 | 207-213 |  | + | 1 |
| **190** | QGPIVLNPWDQV | 12 | Alpha S2 Casein | 2 | 683.36 | 1364.71 | 49.71 | 116-127 |  | + | 1 |
| **191** | QGPIVLNPWDQVK | 13 | Alpha S2 Casein | 2 | 747.41 | 1492.80 | 45.05 | 116-128 |  | + | 1 |
| **192** | QKFALPQY | 8 | Alpha S2 Casein | 2 | 497.77 | 993.53 | 41.93 | 187-194 |  | + | 1 |
| **193** | QKFALPQYL | 9 | Alpha S2 Casein | 2 | 554.31 | 1106.61 | 44.83 | 187-195 |  | + | 1 |
| **194** | REQLSTSEENS | 11 | Alpha S2 Casein | 2 | 640.29 | 1278.57 | 19.88 | 140-150 |  | + | 1 |
| **195** | RNAVPITP | 8 | Alpha S2 Casein | 2 | 434.26 | 866.50 | 32.28 | 129-136 |  | + | 1 |
| **196** | RNAVPITPT | 9 | Alpha S2 Casein | 2 | 484.78 | 967.55 | 30.49 | 129-137 |  | + | 1 |
| **197** | RNAVPITPTL | 10 | Alpha S2 Casein | 2 | 541.32 | 1080.63 | 42.10 | 129-138 |  | + | 1 |
| **198** | RNAVPITPTLN | 11 | Alpha S2 Casein | 2 | 598.34 | 1194.67 | 39.48 | 129-139 |  | + | 1 |
| **199** | TEEEKNRLN | 9 | Alpha S2 Casein | 2 | 566.78 | 1131.55 | 14.98 | 169-177 |  | + | 1 |
| **200** | TEEEKNRLNF | 10 | Alpha S2 Casein | 2 | 640.32 | 1278.62 | 29.50 | 169-178 |  | + | 1 |
| **201** | TEEEKNRLNFL | 11 | Alpha S2 Casein | 2 | 696.86 | 1391.70 | 39.90 | 169-179 |  | + | 1 |
| **202** | TKLTEEE | 7 | Alpha S2 Casein | 2 | 425.21 | 848.41 | 20.14 | 166-172 |  | + | 1 |
| **203** | TKLTEEEKN | 9 | Alpha S2 Casein | 2 | 546.28 | 1090.55 | 15.63 | 166-174 |  | + | 1 |
| **204** | TKLTEEEKNRLNFL | 14 | Alpha S2 Casein | 3 | 578.98 | 1733.93 | 39.42 | 166-179 |  | + | 1 |
| **205** | TKVIPYVR | 8 | Alpha S2 Casein | 2 | 488.30 | 974.59 | 30.59 | 213-220 |  | + | 1 |
| **206** | TVYQHQ | 6 | Alpha S2 Casein | 2 | 388.19 | 774.37 | 16.28 | 197-202 |  | + | 1 |
| **207** | TVYQHQK | 7 | Alpha S2 Casein | 2 | 452.24 | 902.46 | 13.59 | 197-203 |  | + | 1 |
| **208** | VDMESTEVFTK | 11 | Alpha S2 Casein | 2 | 643.30 | 1284.59 | 40.61 | 154-164 |  | + | 1 |
| **209** | VIPYVR | 6 | Alpha S2 Casein | 2 | 373.73 | 745.45 | 34.50 | 215-220 |  | + | 1 |
| **210** | VIPYVRY | 7 | Alpha S2 Casein | 2 | 455.26 | 908.51 | 41.31 | 215-221 |  | + | 1 |
| **211** | VIPYVRYL | 8 | Alpha S2 Casein | 2 | 511.81 | 1021.60 | 44.83 | 215-222 |  | + | 1 |
| **212** | VKR | 3 | Alpha S2 Casein | 1 | 402.28 | 401.28 | 53.82 | 127-129 |  | + | 1 |
| **213** | VPITPTLNR | 9 | Alpha S2 Casein | 2 | 505.80 | 1009.59 | 35.81 | 132-140 |  | + | 1 |
| **214** | VPITPTLNREQ | 11 | Alpha S2 Casein | 2 | 634.35 | 1266.69 | 35.63 | 132-142 |  | + | 1 |
| **215** | VPITPTLNREQL | 12 | Alpha S2 Casein | 2 | 690.90 | 1379.78 | 42.10 | 132-143 |  | + | 1 |
| **216** | VRN | 3 | Alpha S2 Casein | 1 | 388.23 | 387.22 | 50.67 | 59-61 |  | + | 1 |
| **217** | WIQPKTKVIP | 10 | Alpha S2 Casein | 2 | 605.37 | 1208.73 | 37.25 | 208-217 |  | + | 1 |
| **218** | WIQPKTKVIPY | 11 | Alpha S2 Casein | 2 | 686.90 | 1371.79 | 40.90 | 208-218 |  | + | 1 |
| **219** | YLKTVYQH | 8 | Alpha S2 Casein | 2 | 526.28 | 1050.55 | 24.69 | 194-201 |  | + | 1 |
| **220** | YLY | 3 | Alpha S2 Casein | 1 | 458.23 | 457.22 | 31.90 | 113-115 |  | + | 1 |
| **221** | YQGPIVLNPWDQV | 13 | Alpha S2 Casein | 2 | 764.89 | 1527.77 | 50.50 | 115-127 |  | + | 1 |
| **222** | YQGPIVLNPWDQVK | 14 | Alpha S2 Casein | 2 | 828.94 | 1655.87 | 45.79 | 115-128 |  | + | 1 |
| **223** | YQKFALPQ | 8 | Alpha S2 Casein | 2 | 497.77 | 993.53 | 39.30 | 186-193 |  | + | 1 |
| **224** | YQKFALPQY | 9 | Alpha S2 Casein | 2 | 579.30 | 1156.59 | 42.74 | 186-194 |  | + | 1 |
| **225** | YQKFALPQYL | 10 | Alpha S2 Casein | 2 | 635.85 | 1269.68 | 45.28 | 186-195 |  | + | 1 |
| **226** | YQKFALPQYLK | 11 | Alpha S2 Casein | 2 | 699.89 | 1397.77 | 42.29 | 186-196 |  | + | 1 |
| **227** | YQKFALPQYLKT | 12 | Alpha S2 Casein | 2 | 750.42 | 1498.82 | 42.62 | 186-197 |  | + | 1 |
| **228** | AVPYPQRDMPIQ | 12 | Beta Casein | 2 | 707.86 | 1413.71 | 39.84 | 192-203 |  | + | 1 |
| **229** | AVPYPQRDMPIQA | 13 | Beta Casein | 2 | 743.38 | 1484.74 | 41.38 | 192-204 |  | + | 1 |
| **230** | DELQDKIHP | 9 | Beta Casein | 2 | 547.78 | 1093.54 | 32.32 | 58-66 |  | + | 1 |
| **231** | DELQDKIHPF | 10 | Beta Casein | 2 | 621.31 | 1240.61 | 40.20 | 58-67 |  | + | 1 |
| **232** | DELQDKIHPFAQ | 12 | Beta Casein | 2 | 720.86 | 1439.70 | 41.18 | 58-69 |  | + | 1 |
| **233** | DELQDKIHPFAQTQ | 14 | Beta Casein | 2 | 835.41 | 1668.81 | 40.96 | 58-71 |  | + | 1 |
| **234** | DKIHPF | 6 | Beta Casein | 2 | 378.71 | 755.40 | 28.85 | 62-67 |  | + | 1 |
| **235** | DKIHPFA | 7 | Beta Casein | 2 | 414.22 | 826.43 | 28.70 | 62-68 |  | + | 1 |
| **236** | DKIHPFAQ | 8 | Beta Casein | 2 | 478.25 | 954.49 | 27.95 | 62-69 |  | + | 1 |
| **237** | DKIHPFAQT | 9 | Beta Casein | 2 | 528.78 | 1055.54 | 28.98 | 62-70 |  | + | 1 |
| **238** | DKIHPFAQTQ | 10 | Beta Casein | 2 | 592.81 | 1183.60 | 28.20 | 62-71 |  | + | 1 |
| **239** | DKIHPFAQTQS | 11 | Beta Casein | 2 | 636.32 | 1270.63 | 27.99 | 62-72 |  | + | 1 |
| **240** | DKIHPFAQTQSL | 12 | Beta Casein | 2 | 692.86 | 1383.71 | 37.62 | 62-73 |  | + | 1 |
| **241** | DVENLHL | 7 | Beta Casein | 2 | 420.22 | 838.42 | 42.46 | 144-150 |  | + | 1 |
| **242** | DVENLHLPLP | 10 | Beta Casein | 2 | 573.81 | 1145.61 | 47.49 | 144-153 |  | + | 1 |
| **243** | DVENLHLPLPL | 11 | Beta Casein | 2 | 630.35 | 1258.69 | 50.69 | 144-154 |  | + | 1 |
| **244** | DVENLHLPLPLL | 12 | Beta Casein | 2 | 686.90 | 1371.78 | 53.13 | 144-155 |  | + | 1 |
| **245** | DVENLHLPLPLLQ | 13 | Beta Casein | 2 | 750.92 | 1499.83 | 51.92 | 144-156 |  | + | 1 |
| **246** | DVENLHLPLPLLQS | 14 | Beta Casein | 2 | 794.44 | 1586.87 | 51.52 | 144-157 |  | + | 1 |
| **247** | EDELQDKIHPFAQTQ | 15 | Beta Casein | 2 | 899.93 | 1797.85 | 40.82 | 57-71 |  | + | 1 |
| **248** | ELQDKIHPF | 9 | Beta Casein | 2 | 563.80 | 1125.58 | 37.72 | 59-67 |  | + | 1 |
| **249** | ELQDKIHPFAQTQ | 13 | Beta Casein | 2 | 777.90 | 1553.78 | 36.41 | 59-71 |  | + | 1 |
| **250** | EMPFPKYPVEP | 11 | Beta Casein | 2 | 667.33 | 1332.64 | 44.07 | 123-133 |  | + | 1 |
| **251** | EMPFPKYPVEPFTE | 14 | Beta Casein | 2 | 855.91 | 1709.80 | 46.26 | 123-136 |  | + | 1 |
| **252** | EMPFPKYPVEPFTESQS | 17 | Beta Casein | 2 | 1006.97 | 2011.92 | 45.63 | 123-139 |  | + | 1 |
| **253** | ENLHLPLPLLQ | 11 | Beta Casein | 2 | 643.88 | 1285.74 | 49.59 | 146-156 |  | + | 1 |
| **254** | EPVLGPVR | 8 | Beta Casein | 2 | 433.76 | 865.50 | 34.03 | 210-217 |  | + | 1 |
| **255** | EPVLGPVRGP | 10 | Beta Casein | 2 | 510.80 | 1019.58 | 38.93 | 210-219 |  | + | 1 |
| **256** | EPVLGPVRGPFP | 12 | Beta Casein | 2 | 632.86 | 1263.70 | 44.74 | 210-221 |  | + | 1 |
| **257** | EPVLGPVRGPFPI | 13 | Beta Casein | 2 | 689.40 | 1376.78 | 47.13 | 210-222 |  | + | 1 |
| **258** | EPVLGPVRGPFPII | 14 | Beta Casein | 2 | 745.94 | 1489.87 | 49.10 | 210-223 |  | + | 1 |
| **259** | EPVLGPVRGPFPIIV | 15 | Beta Casein | 2 | 795.47 | 1588.93 | 49.97 | 210-224 |  | + | 1 |
| **260** | ESQSLTLTDV | 10 | Beta Casein | 2 | 546.77 | 1091.53 | 35.02 | 136-145 |  | + | 1 |
| **261** | FPKYPVEP | 8 | Beta Casein | 2 | 488.76 | 975.51 | 40.26 | 126-133 |  | + | 1 |
| **262** | FQSEEQQQTEDELQDK | 16 | Beta Casein | 2 | 991.43 | 1980.85 | 31.57 | 48-63 |  | + | 1 |
| **263** | GPVRGPFP | 8 | Beta Casein | 2 | 413.73 | 825.45 | 36.91 | 214-221 |  | + | 1 |
| **264** | GPVRGPFPIIV | 11 | Beta Casein | 2 | 576.35 | 1150.69 | 46.90 | 214-224 |  | + | 1 |
| **265** | HLPLPLLQ | 8 | Beta Casein | 2 | 465.79 | 929.57 | 46.46 | 149-156 |  | + | 1 |
| **266** | HQPHQPLPPTVMFPPQS | 17 | Beta Casein | 3 | 646.66 | 1936.96 | 42.68 | 160-176 |  | + | 1 |
| **267** | IHPFAQTQ | 8 | Beta Casein | 2 | 471.25 | 940.48 | 29.37 | 64-71 |  | + | 1 |
| **268** | IHPFAQTQS | 9 | Beta Casein | 2 | 514.76 | 1027.51 | 29.05 | 64-72 |  | + | 1 |
| **269** | IHPFAQTQSL | 10 | Beta Casein | 2 | 571.30 | 1140.59 | 39.47 | 64-73 |  | + | 1 |
| **270** | INKKIE | 6 | Beta Casein | 2 | 372.73 | 743.45 | 14.61 | 41-46 |  | + | 1 |
| **271** | INKKIEK | 7 | Beta Casein | 2 | 436.78 | 871.55 | 13.99 | 41-47 |  | + | 1 |
| **272** | KEMPFPKYPVEP | 12 | Beta Casein | 2 | 731.38 | 1460.74 | 41.34 | 122-133 |  | + | 1 |
| **273** | KVKEAMAP | 8 | Beta Casein | 2 | 437.25 | 872.48 | 18.41 | 112-119 |  | + | 1 |
| **274** | KVKEAMAPK | 9 | Beta Casein | 2 | 501.29 | 1000.57 | 15.04 | 112-120 |  | + | 1 |
| **275** | KVLPVPQ | 7 | Beta Casein | 2 | 390.75 | 779.49 | 33.03 | 184-190 |  | + | 1 |
| **276** | KVLPVPQK | 8 | Beta Casein | 2 | 454.80 | 907.59 | 24.48 | 184-191 |  | + | 1 |
| **277** | KVLPVPQKAVP | 11 | Beta Casein | 2 | 588.38 | 1174.74 | 33.21 | 184-194 |  | + | 1 |
| **278** | KVLPVPQKAVPYPQ | 14 | Beta Casein | 2 | 782.47 | 1562.92 | 37.40 | 184-197 |  | + | 1 |
| **279** | KYPVEPFTE | 9 | Beta Casein | 2 | 555.28 | 1108.54 | 40.72 | 128-136 |  | + | 1 |
| **280** | KYPVEPFTES | 10 | Beta Casein | 2 | 598.80 | 1195.58 | 40.14 | 128-137 |  | + | 1 |
| **281** | KYPVEPFTESQ | 11 | Beta Casein | 2 | 662.82 | 1323.63 | 39.61 | 128-138 |  | + | 1 |
| **282** | KYPVEPFTESQS | 12 | Beta Casein | 2 | 706.34 | 1410.67 | 39.15 | 128-139 |  | + | 1 |
| **283** | LGPVRGPFPIIV | 12 | Beta Casein | 2 | 632.89 | 1263.77 | 48.36 | 213-224 |  | + | 1 |
| **284** | LHLPLPLLQ | 9 | Beta Casein | 2 | 522.33 | 1042.65 | 49.25 | 148-156 |  | + | 1 |
| **285** | LHLPLPLLQS | 10 | Beta Casein | 2 | 565.85 | 1129.69 | 48.98 | 148-157 |  | + | 1 |
| **286** | LLYQEPVLGPVRGP | 14 | Beta Casein | 2 | 769.44 | 1536.87 | 44.36 | 206-219 |  | + | 1 |
| **287** | LLYQEPVLGPVRGPFP | 16 | Beta Casein | 2 | 891.50 | 1780.99 | 47.23 | 206-221 |  | + | 1 |
| **288** | LLYQEPVLGPVRGPFPI | 17 | Beta Casein | 2 | 948.04 | 1894.07 | 49.41 | 206-222 |  | + | 1 |
| **289** | LPLPLLQ | 7 | Beta Casein | 2 | 397.26 | 792.51 | 50.70 | 150-156 |  | + | 1 |
| **290** | LPLPLLQS | 8 | Beta Casein | 2 | 440.78 | 879.54 | 50.19 | 150-157 |  | + | 1 |
| **291** | LPPTVMFPP | 9 | Beta Casein | 2 | 499.77 | 997.53 | 48.51 | 166-174 |  | + | 1 |
| **292** | LPPTVMFPPQS | 11 | Beta Casein | 2 | 607.32 | 1212.62 | 46.40 | 166-176 |  | + | 1 |
| **293** | LQDKIHPF | 8 | Beta Casein | 2 | 499.28 | 996.54 | 33.19 | 60-67 |  | + | 1 |
| **294** | LQDKIHPFAQTQ | 12 | Beta Casein | 2 | 713.38 | 1424.74 | 32.09 | 60-71 |  | + | 1 |
| **295** | LQDKIHPFAQTQS | 13 | Beta Casein | 2 | 756.89 | 1511.77 | 31.85 | 60-72 |  | + | 1 |
| **296** | LQPEVMGVSK | 10 | Beta Casein | 2 | 544.29 | 1086.57 | 34.90 | 103-112 |  | + | 1 |
| **297** | LSQSKVLPVPQ | 11 | Beta Casein | 2 | 598.36 | 1194.70 | 38.38 | 180-190 |  | + | 1 |
| **298** | LSSSEESITRIN | 12 | Beta Casein | 2 | 668.34 | 1334.67 | 33.83 | 31-42 |  | + | 1 |
| **299** | LTDVENLH | 8 | Beta Casein | 2 | 470.74 | 939.47 | 31.50 | 142-149 |  | + | 1 |
| **300** | LTDVENLHL | 9 | Beta Casein | 2 | 527.28 | 1052.55 | 43.39 | 142-150 |  | + | 1 |
| **301** | LTDVENLHLP | 10 | Beta Casein | 2 | 575.81 | 1149.60 | 44.09 | 142-151 |  | + | 1 |
| **302** | LTDVENLHLPLP | 12 | Beta Casein | 2 | 680.88 | 1359.74 | 47.32 | 142-153 |  | + | 1 |
| **303** | LTDVENLHLPLPL | 13 | Beta Casein | 2 | 737.42 | 1472.82 | 50.01 | 142-154 |  | + | 1 |
| **304** | LTDVENLHLPLPLLQ | 15 | Beta Casein | 2 | 857.99 | 1713.97 | 51.16 | 142-156 |  | + | 1 |
| **305** | LTDVENLHLPLPLLQS | 16 | Beta Casein | 2 | 901.51 | 1801.00 | 50.83 | 142-157 |  | + | 1 |
| **306** | LTLTDVENLH | 10 | Beta Casein | 2 | 577.81 | 1153.60 | 43.74 | 140-149 |  | + | 1 |
| **307** | LYQEPVLGPVR | 11 | Beta Casein | 2 | 635.86 | 1269.71 | 41.72 | 207-217 |  | + | 1 |
| **308** | LYQEPVLGPVRGP | 13 | Beta Casein | 2 | 712.90 | 1423.78 | 42.74 | 207-219 |  | + | 1 |
| **309** | LYQEPVLGPVRGPFP | 15 | Beta Casein | 2 | 834.96 | 1667.90 | 45.79 | 207-221 |  | + | 1 |
| **310** | LYQEPVLGPVRGPFPI | 16 | Beta Casein | 2 | 891.50 | 1780.99 | 48.16 | 207-222 |  | + | 1 |
| **311** | LYQEPVLGPVRGPFPII | 17 | Beta Casein | 2 | 948.04 | 1894.07 | 49.87 | 207-223 |  | + | 1 |
| **312** | LYQEPVLGPVRGPFPIIV | 18 | Beta Casein | 2 | 997.58 | 1993.14 | 50.66 | 207-224 |  | + | 1 |
| **313** | MPFPKYPVE | 9 | Beta Casein | 2 | 554.28 | 1106.55 | 42.68 | 124-132 |  | + | 1 |
| **314** | MPFPKYPVEP | 10 | Beta Casein | 2 | 602.81 | 1203.60 | 43.39 | 124-133 |  | + | 1 |
| **315** | MPFPKYPVEPF | 11 | Beta Casein | 2 | 676.34 | 1350.67 | 46.18 | 124-134 |  | + | 1 |
| **316** | MPFPKYPVEPFTE | 13 | Beta Casein | 2 | 791.39 | 1580.76 | 45.61 | 124-136 |  | + | 1 |
| **317** | MPFPKYPVEPFTESQSL | 17 | Beta Casein | 2 | 998.99 | 1995.97 | 46.40 | 124-140 |  | + | 1 |
| **318** | NLHLPLPLLQ | 10 | Beta Casein | 2 | 579.36 | 1156.70 | 49.53 | 147-156 |  | + | 1 |
| **319** | NLHLPLPLLQS | 11 | Beta Casein | 2 | 622.87 | 1243.73 | 49.24 | 147-157 |  | + | 1 |
| **320** | NVPGEIVESLSSSEESITR | 19 | Beta Casein | 2 | 1017.01 | 2032.00 | 45.70 | 22-40 |  | + | 1 |
| **321** | NVPGEIVESLSSSEESITRIN | 21 | Beta Casein | 2 | 1130.57 | 2259.12 | 47.22 | 22-42 |  | + | 1 |
| **322** | QEPVLGPVR | 9 | Beta Casein | 2 | 497.79 | 993.56 | 34.40 | 209-217 |  | + | 1 |
| **323** | QEPVLGPVRGP | 11 | Beta Casein | 2 | 574.82 | 1147.63 | 39.06 | 209-219 |  | + | 1 |
| **324** | QEPVLGPVRGPFP | 13 | Beta Casein | 2 | 696.89 | 1391.76 | 44.55 | 209-221 |  | + | 1 |
| **325** | QEPVLGPVRGPFPI | 14 | Beta Casein | 2 | 753.43 | 1504.84 | 46.87 | 209-222 |  | + | 1 |
| **326** | QEPVLGPVRGPFPII | 15 | Beta Casein | 2 | 809.97 | 1617.92 | 48.76 | 209-223 |  | + | 1 |
| **327** | QEPVLGPVRGPFPIIV | 16 | Beta Casein | 2 | 859.50 | 1716.99 | 49.65 | 209-224 |  | + | 1 |
| **328** | QPH | 3 | Beta Casein | 1 | 381.19 | 380.18 | 56.41 | 161-163 |  | + | 1 |
| **329** | QQQTEDELQDK | 11 | Beta Casein | 2 | 681.31 | 1360.61 | 22.26 | 53-63 |  | + | 1 |
| **330** | QQTEDELQDK | 10 | Beta Casein | 2 | 617.28 | 1232.55 | 21.96 | 54-63 |  | + | 1 |
| **331** | QTEDELQDK | 9 | Beta Casein | 2 | 553.25 | 1104.49 | 20.97 | 55-63 |  | + | 1 |
| **332** | RDMPIQA | 7 | Beta Casein | 2 | 415.71 | 829.41 | 30.16 | 198-204 |  | + | 1 |
| **333** | RDMPIQAF | 8 | Beta Casein | 2 | 489.25 | 976.48 | 42.40 | 198-205 |  | + | 1 |
| **334** | RDMPIQAFL | 9 | Beta Casein | 2 | 545.79 | 1089.56 | 46.46 | 198-206 |  | + | 1 |
| **335** | RDMPIQAFLL | 10 | Beta Casein | 2 | 602.33 | 1202.65 | 49.04 | 198-207 |  | + | 1 |
| **336** | RELEELNVPG | 10 | Beta Casein | 2 | 578.30 | 1154.59 | 39.64 | 16-25 |  | + | 1 |
| **337** | RELEELNVPGE | 11 | Beta Casein | 2 | 642.83 | 1283.64 | 40.68 | 16-26 |  | + | 1 |
| **338** | SQSKVLPVPQ | 10 | Beta Casein | 2 | 541.81 | 1081.61 | 34.82 | 181-190 |  | + | 1 |
| **339** | TDVENLHL | 8 | Beta Casein | 2 | 470.74 | 939.47 | 41.83 | 143-150 |  | + | 1 |
| **340** | TDVENLHLP | 9 | Beta Casein | 2 | 519.27 | 1036.52 | 43.03 | 143-151 |  | + | 1 |
| **341** | TDVENLHLPLP | 11 | Beta Casein | 2 | 624.34 | 1246.66 | 46.45 | 143-153 |  | + | 1 |
| **342** | TDVENLHLPLPL | 12 | Beta Casein | 2 | 680.88 | 1359.74 | 49.41 | 143-154 |  | + | 1 |
| **343** | TDVENLHLPLPLL | 13 | Beta Casein | 2 | 737.42 | 1472.82 | 51.69 | 143-155 |  | + | 1 |
| **344** | TDVENLHLPLPLLQ | 14 | Beta Casein | 2 | 801.45 | 1600.88 | 50.66 | 143-156 |  | + | 1 |
| **345** | TDVENLHLPLPLLQS | 15 | Beta Casein | 2 | 844.96 | 1687.91 | 50.33 | 143-157 |  | + | 1 |
| **346** | TDVENLHLPLPLLQSW | 16 | Beta Casein | 2 | 938.00 | 1873.99 | 53.32 | 143-158 |  | + | 1 |
| **347** | TEDELQDKIHP | 11 | Beta Casein | 2 | 662.82 | 1323.63 | 32.57 | 56-66 |  | + | 1 |
| **348** | TEDELQDKIHPF | 12 | Beta Casein | 2 | 736.36 | 1470.70 | 41.43 | 56-67 |  | + | 1 |
| **349** | TLTDVENLH | 9 | Beta Casein | 2 | 521.26 | 1040.51 | 36.49 | 141-149 |  | + | 1 |
| **350** | VENLHLPLPL | 10 | Beta Casein | 2 | 572.84 | 1143.67 | 48.21 | 145-154 |  | + | 1 |
| **351** | VENLHLPLPLLQ | 12 | Beta Casein | 2 | 693.41 | 1384.81 | 49.66 | 145-156 |  | + | 1 |
| **352** | VENLHLPLPLLQS | 13 | Beta Casein | 2 | 736.93 | 1471.84 | 49.39 | 145-157 |  | + | 1 |
| **353** | VKEAMAP | 7 | Beta Casein | 2 | 373.20 | 744.38 | 22.93 | 113-119 |  | + | 1 |
| **354** | VKEAMAPK | 8 | Beta Casein | 2 | 437.25 | 872.48 | 16.15 | 113-120 |  | + | 1 |
| **355** | VLGPVRGP | 8 | Beta Casein | 2 | 397.75 | 793.48 | 32.20 | 212-219 |  | + | 1 |
| **356** | VLGPVRGPFP | 10 | Beta Casein | 2 | 519.81 | 1037.60 | 43.31 | 212-221 |  | + | 1 |
| **357** | VLGPVRGPFPII | 12 | Beta Casein | 2 | 632.89 | 1263.77 | 47.90 | 212-223 |  | + | 1 |
| **358** | VLGPVRGPFPIIV | 13 | Beta Casein | 2 | 682.43 | 1362.84 | 48.85 | 212-224 |  | + | 1 |
| **359** | VLPVPQK | 7 | Beta Casein | 2 | 390.75 | 779.49 | 29.42 | 185-191 |  | + | 1 |
| **360** | VLPVPQKAVPYP | 12 | Beta Casein | 2 | 654.39 | 1306.76 | 42.57 | 185-196 |  | + | 1 |
| **361** | VLPVPQKAVPYPQ | 13 | Beta Casein | 2 | 718.42 | 1434.82 | 41.83 | 185-197 |  | + | 1 |
| **362** | VLPVPQKAVPYPQR | 14 | Beta Casein | 2 | 796.47 | 1590.92 | 37.63 | 185-198 |  | + | 1 |
| **363** | VMGVSKVK | 8 | Beta Casein | 2 | 424.26 | 846.50 | 20.38 | 107-114 |  | + | 1 |
| **364** | VPQKAVPYPQ | 10 | Beta Casein | 2 | 563.82 | 1125.62 | 30.19 | 188-197 |  | + | 1 |
| **365** | VPYPQRDMPIQ | 11 | Beta Casein | 2 | 672.34 | 1342.67 | 38.33 | 193-203 |  | + | 1 |
| **366** | VPYPQRDMPIQA | 12 | Beta Casein | 2 | 707.86 | 1413.71 | 40.53 | 193-204 |  | + | 1 |
| **367** | VPYPQRDMPIQAF | 13 | Beta Casein | 2 | 781.40 | 1560.78 | 44.21 | 193-205 |  | + | 1 |
| **368** | WMH | 3 | Beta Casein | 1 | 473.20 | 472.19 | 22.48 | 158-160 |  | + | 1 |
| **369** | YPF | 3 | Beta Casein | 1 | 426.20 | 425.20 | 42.72 | 75-77 |  | + | 1 |
| **370** | YPQRDMPIQ | 9 | Beta Casein | 2 | 574.28 | 1146.55 | 32.23 | 195-203 |  | + | 1 |
| **371** | YPQRDMPIQA | 10 | Beta Casein | 2 | 609.80 | 1217.59 | 35.26 | 195-204 |  | + | 1 |
| **372** | YPQRDMPIQAF | 11 | Beta Casein | 2 | 683.33 | 1364.65 | 43.31 | 195-205 |  | + | 1 |
| **373** | YPVEPFTESQS | 11 | Beta Casein | 2 | 642.29 | 1282.57 | 43.36 | 129-139 |  | + | 1 |
| **374** | YQEPVLGPVR | 10 | Beta Casein | 2 | 579.32 | 1156.62 | 38.84 | 208-217 |  | + | 1 |
| **375** | YQEPVLGPVRG | 11 | Beta Casein | 2 | 607.83 | 1213.65 | 39.01 | 208-218 |  | + | 1 |
| **376** | YQEPVLGPVRGP | 12 | Beta Casein | 2 | 656.36 | 1310.70 | 41.72 | 208-219 |  | + | 1 |
| **377** | YQEPVLGPVRGPFP | 14 | Beta Casein | 2 | 778.42 | 1554.82 | 45.10 | 208-221 |  | + | 1 |
| **378** | YQEPVLGPVRGPFPI | 15 | Beta Casein | 2 | 834.96 | 1667.90 | 47.46 | 208-222 |  | + | 1 |
| **379** | YQEPVLGPVRGPFPII | 16 | Beta Casein | 2 | 891.50 | 1780.99 | 49.25 | 208-223 |  | + | 1 |
| **380** | YQEPVLGPVRGPFPIIV | 17 | Beta Casein | 2 | 941.04 | 1880.06 | 50.06 | 208-224 |  | + | 1 |
| **381** | ALPMHIR | 7 | Beta Lactoglobulin | 2 | 419.24 | 836.47 | 30.19 | 158-164 |  | + | 1 |
| **382** | DEALEKFDKAL | 11 | Beta Lactoglobulin | 2 | 639.83 | 1277.65 | 42.46 | 146-156 |  | + | 1 |
| **383** | FCM | 3 | Beta Lactoglobulin | 1 | 457.16 | 456.15 | 13.88 | 121-123 |  | + | 1 |
| **384** | IVTQTMKGLDIQ | 12 | Beta Lactoglobulin | 2 | 673.87 | 1345.73 | 41.78 | 18-29 |  | + | 1 |
| **385** | LIVTQTMKGLDIQ | 13 | Beta Lactoglobulin | 2 | 730.41 | 1458.81 | 44.27 | 17-29 |  | + | 1 |
| **386** | QKK | 3 | Beta Lactoglobulin | 1 | 403.27 | 402.26 | 60.38 | 84-86 |  | + | 1 |
| **387** | TQTMKGLDIQ | 10 | Beta Lactoglobulin | 2 | 567.79 | 1133.58 | 36.38 | 20-29 |  | + | 1 |
| **388** | VEELKPTPEGDL | 12 | Beta Lactoglobulin | 2 | 663.84 | 1325.67 | 38.38 | 59-70 |  | + | 1 |
| **389** | VEELKPTPEGDLEILL | 16 | Beta Lactoglobulin | 2 | 897.99 | 1793.97 | 48.35 | 59-74 |  | + | 1 |
| **390** | DKTEIPTINT | 10 | Kappa Casein | 2 | 566.30 | 1130.58 | 39.89 | 136-145 |  | + | 1 |
| **391** | ESPPEINTVQVT | 12 | Kappa Casein | 2 | 657.33 | 1312.65 | 42.29 | 175-186 |  | + | 1 |
| **392** | ESPPEINTVQVTSTAV | 16 | Kappa Casein | 2 | 836.43 | 1670.84 | 44.03 | 175-190 |  | + | 1 |
| **393** | FFS | 3 | Kappa Casein | 1 | 400.19 | 399.18 | 44.66 | 38-40 |  | + | 1 |
| **394** | KYIPIQY | 7 | Kappa Casein | 2 | 462.76 | 923.51 | 39.85 | 45-51 |  | + | 1 |
| **395** | KYIPIQYVL | 9 | Kappa Casein | 2 | 568.84 | 1135.66 | 45.43 | 45-53 |  | + | 1 |
| **396** | KYIPIQYVLS | 10 | Kappa Casein | 2 | 612.36 | 1222.70 | 44.73 | 45-54 |  | + | 1 |
| **397** | PEVIESPPEINTVQVTSTAV | 20 | Kappa Casein | 2 | 1055.55 | 2109.08 | 46.04 | 171-190 |  | + | 1 |
| **398** | QDKTEIPTINT | 11 | Kappa Casein | 2 | 630.33 | 1258.64 | 38.19 | 135-145 |  | + | 1 |
| **399** | RFFSDKIA | 8 | Kappa Casein | 2 | 492.27 | 982.52 | 34.26 | 37-44 |  | + | 1 |
| **400** | SPPEINTVQ | 9 | Kappa Casein | 2 | 492.75 | 983.49 | 34.82 | 176-184 |  | + | 1 |
| **401** | SPPEINTVQVT | 11 | Kappa Casein | 2 | 592.81 | 1183.61 | 41.78 | 176-186 |  | + | 1 |
| **402** | SPPEINTVQVTSTAV | 15 | Kappa Casein | 2 | 771.90 | 1541.79 | 43.73 | 176-190 |  | + | 1 |
| **403** | YYQ | 3 | Kappa Casein | 1 | 473.20 | 472.20 | 20.04 | 63-65 |  | + | 1 |
| **404** | YYQQKPVAL | 9 | Kappa Casein | 2 | 555.30 | 1108.59 | 34.70 | 63-71 |  | + | 1 |

**Supplementary Table 12.** Milk peptides identified throughout the 28 days of storage period using the *E. faecalis* NCCB 100525.

|  | **Sequence** | **Length** | **Leading proteins** | **Charge** | **m/z** | **Mass** | **Retention time** | **Sequence** | **Day 1** | **Day 28** | **Incidence** |
| --- | --- | --- | --- | --- | --- | --- | --- | --- | --- | --- | --- |
| **1** | DIPNPIGSENSEKTTMPLW | 19 | Alpha S1 Casein | 2 | 1065.01 | 2128.01 | 48.05 | 196-214 |  |  | 1 |
| **2** | EVLNENLLR | 9 | Alpha S1 Casein | 2 | 550.31 | 1098.60 | 42.01 | 29-37 |  | + | 1 |
| **3** | FFVAPFPEVFGK | 12 | Alpha S1 Casein | 2 | 692.87 | 1383.72 | 49.96 | 38-49 |  | + | 1 |
| **4** | FSDIPNPIGSENSEK | 15 | Alpha S1 Casein | 2 | 817.39 | 1632.76 | 41.92 | 194-208 |  | + | 1 |
| **5** | FSDIPNPIGSENSEKT | 16 | Alpha S1 Casein | 2 | 867.91 | 1733.81 | 42.01 | 194-209 |  | + | 1 |
| **6** | FSDIPNPIGSENSEKTT | 17 | Alpha S1 Casein | 2 | 918.44 | 1834.86 | 42.04 | 194-210 |  | + | 1 |
| **7** | FSDIPNPIGSENSEKTTMP | 19 | Alpha S1 Casein | 2 | 1032.48 | 2062.95 | 43.45 | 194-212 |  | + | 1 |
| **8** | FSDIPNPIGSENSEKTTMPL | 20 | Alpha S1 Casein | 2 | 1089.03 | 2176.04 | 45.11 | 194-213 |  | + | 1 |
| **9** | FSDIPNPIGSENSEKTTMPLW | 21 | Alpha S1 Casein | 2 | 1190.06 | 2378.11 | 46.07 | 194-214 |  | + | 1 |
| **10** | FVAPFPEVFGK | 11 | Alpha S1 Casein | 2 | 619.33 | 1236.65 | 47.44 | 39-49 |  | + | 1 |
| **11** | FVAPFPEVFGKE | 12 | Alpha S1 Casein | 2 | 683.86 | 1365.70 | 47.48 | 39-50 |  | + | 1 |
| **12** | FVAPFPEVFGKEK | 13 | Alpha S1 Casein | 2 | 747.90 | 1493.79 | 44.64 | 39-51 |  | + | 1 |
| **13** | FVAPFPEVFGKEKV | 14 | Alpha S1 Casein | 3 | 531.96 | 1592.86 | 45.14 | 39-52 |  | + | 1 |
| **14** | HQGLPQEVLNENL | 13 | Alpha S1 Casein | 2 | 745.88 | 1489.75 | 43.37 | 23-35 |  | + | 1 |
| **15** | IPNPIGSENSEKTTMP | 16 | Alpha S1 Casein | 2 | 857.92 | 1713.82 | 41.07 | 197-212 |  | + | 1 |
| **16** | IPNPIGSENSEKTTMPL | 17 | Alpha S1 Casein | 2 | 914.46 | 1826.91 | 43.42 | 197-213 |  | + | 1 |
| **17** | IPNPIGSENSEKTTMPLW | 18 | Alpha S1 Casein | 2 | 1015.50 | 2028.98 | 44.47 | 197-214 |  | + | 1 |
| **18** | KEPMIGVNQEL | 11 | Alpha S1 Casein | 2 | 629.33 | 1256.64 | 41.71 | 147-157 |  | + | 1 |
| **19** | KTTMPLW | 7 | Alpha S1 Casein | 2 | 438.74 | 875.46 | 43.53 | 208-214 |  | + | 1 |
| **20** | QEVLNENLLR | 10 | Alpha S1 Casein | 2 | 614.34 | 1226.66 | 42.15 | 28-37 |  | + | 1 |
| **21** | QGLPQEVLNENLLR | 14 | Alpha S1 Casein | 2 | 811.95 | 1621.88 | 45.20 | 24-37 |  | + | 1 |
| **22** | QKEPMIGVNQEL | 12 | Alpha S1 Casein | 2 | 693.36 | 1384.70 | 41.72 | 146-157 |  | + | 1 |
| **23** | SDIPNPIGSENSEKTTMP | 18 | Alpha S1 Casein | 2 | 958.95 | 1915.88 | 42.07 | 195-212 |  | + | 1 |
| **24** | SDIPNPIGSENSEKTTMPLW | 20 | Alpha S1 Casein | 2 | 1108.53 | 2215.05 | 46.72 | 195-214 |  | + | 1 |
| **25** | VAPFPEVFGK | 10 | Alpha S1 Casein | 2 | 545.80 | 1089.59 | 44.96 | 40-49 |  | + | 1 |
| **26** | VAPFPEVFGKE | 11 | Alpha S1 Casein | 2 | 610.32 | 1218.63 | 45.00 | 40-50 |  | + | 1 |
| **27** | VAPFPEVFGKEK | 12 | Alpha S1 Casein | 2 | 674.37 | 1346.72 | 42.41 | 40-51 |  | + | 1 |
| **28** | ALPQYLK | 7 | Alpha S2 Casein | 2 | 416.75 | 831.49 | 36.57 | 190-196 |  | + | 1 |
| **29** | ALPQYLKT | 8 | Alpha S2 Casein | 2 | 467.27 | 932.53 | 38.85 | 190-197 |  | + | 1 |
| **30** | FALPQYLK | 8 | Alpha S2 Casein | 2 | 490.28 | 978.55 | 44.10 | 189-196 |  | + | 1 |
| **31** | FALPQYLKT | 9 | Alpha S2 Casein | 2 | 540.81 | 1079.60 | 44.43 | 189-197 |  | + | 1 |
| **32** | GPIVLNPWDQVK | 12 | Alpha S2 Casein | 2 | 683.38 | 1364.75 | 45.16 | 117-128 |  | + | 1 |
| **33** | NAVPITPTLNR | 11 | Alpha S2 Casein | 2 | 598.34 | 1194.67 | 38.88 | 130-140 |  | + | 1 |
| **34** | PWD | 3 | Alpha S2 Casein | 1 | 417.18 | 416.17 | 28.77 | 123-125 |  | + | 1 |
| **35** | VIPYVRYL | 8 | Alpha S2 Casein | 2 | 511.81 | 1021.60 | 44.93 | 215-222 |  | + | 1 |
| **36** | YQGPIVLNPWDQVK | 14 | Alpha S2 Casein | 2 | 828.94 | 1655.87 | 45.92 | 115-128 |  | + | 1 |
| **37** | YQKFALPQYLK | 11 | Alpha S2 Casein | 2 | 699.89 | 1397.77 | 42.40 | 186-196 |  | + | 1 |
| **38** | YQKFALPQYLKT | 12 | Alpha S2 Casein | 2 | 750.42 | 1498.82 | 42.69 | 186-197 |  | + | 1 |
| **39** | AVPYPQRDMPIQ | 12 | Beta Casein | 2 | 707.86 | 1413.71 | 40.08 | 192-203 |  | + | 1 |
| **40** | AVPYPQRDMPIQA | 13 | Beta Casein | 2 | 743.38 | 1484.74 | 41.46 | 192-204 |  | + | 1 |
| **41** | AVPYPQRDMPIQAF | 14 | Beta Casein | 2 | 816.91 | 1631.81 | 44.52 | 192-205 |  | + | 1 |
| **42** | AVPYPQRDMPIQAFL | 15 | Beta Casein | 2 | 873.46 | 1744.90 | 47.84 | 192-206 |  | + | 1 |
| **43** | DKIHPFAQTQ | 10 | Beta Casein | 2 | 592.81 | 1183.60 | 28.45 | 62-71 |  | + | 1 |
| **44** | DKIHPFAQTQS | 11 | Beta Casein | 2 | 636.32 | 1270.63 | 28.25 | 62-72 |  | + | 1 |
| **45** | DKIHPFAQTQSL | 12 | Beta Casein | 2 | 692.86 | 1383.71 | 37.93 | 62-73 |  | + | 1 |
| **46** | ENLHLPLPLLQS | 12 | Beta Casein | 2 | 687.39 | 1372.77 | 49.39 | 146-157 |  | + | 1 |
| **47** | EPVLGPVRGP | 10 | Beta Casein | 2 | 510.80 | 1019.58 | 39.18 | 210-219 |  | + | 1 |
| **48** | EPVLGPVRGPFP | 12 | Beta Casein | 2 | 632.86 | 1263.70 | 44.88 | 210-221 |  | + | 1 |
| **49** | EPVLGPVRGPFPI | 13 | Beta Casein | 2 | 689.40 | 1376.78 | 47.26 | 210-222 |  | + | 1 |
| **50** | EPVLGPVRGPFPII | 14 | Beta Casein | 2 | 745.94 | 1489.87 | 49.19 | 210-223 |  | + | 1 |
| **51** | EPVLGPVRGPFPIIV | 15 | Beta Casein | 2 | 795.47 | 1588.93 | 50.11 | 210-224 |  | + | 1 |
| **52** | IHPFAQTQ | 8 | Beta Casein | 2 | 471.25 | 940.48 | 29.60 | 64-71 |  | + | 1 |
| **53** | IHPFAQTQSL | 10 | Beta Casein | 2 | 571.30 | 1140.59 | 39.74 | 64-73 |  | + | 1 |
| **54** | KVLPVPQ | 7 | Beta Casein | 2 | 390.75 | 779.49 | 33.30 | 184-190 |  | + | 1 |
| **55** | KYPVEPFTE | 9 | Beta Casein | 2 | 555.28 | 1108.54 | 40.97 | 128-136 |  | + | 1 |
| **56** | KYPVEPFTES | 10 | Beta Casein | 2 | 598.80 | 1195.58 | 40.39 | 128-137 |  | + | 1 |
| **57** | LGPVRGPFPIIV | 12 | Beta Casein | 2 | 632.89 | 1263.77 | 48.47 | 213-224 |  | + | 1 |
| **58** | LYQEPVLGPVRGPFPII | 17 | Beta Casein | 2 | 948.04 | 1894.07 | 50.01 | 207-223 |  | + | 1 |
| **59** | LYQEPVLGPVRGPFPIIV | 18 | Beta Casein | 2 | 997.58 | 1993.14 | 50.81 | 207-224 |  | + | 1 |
| **60** | NVPGEIVESLSSSEESITR | 19 | Beta Casein | 2 | 1017.01 | 2032.00 | 45.80 | 22-40 |  | + | 1 |
| **61** | NVPGEIVESLSSSEESITRIN | 21 | Beta Casein | 2 | 1130.57 | 2259.12 | 47.33 | 22-42 |  | + | 1 |
| **62** | QEPVLGPVRGP | 11 | Beta Casein | 2 | 574.82 | 1147.63 | 39.32 | 209-219 |  | + | 1 |
| **63** | QEPVLGPVRGPFP | 13 | Beta Casein | 2 | 696.89 | 1391.76 | 44.66 | 209-221 |  | + | 1 |
| **64** | QEPVLGPVRGPFPI | 14 | Beta Casein | 2 | 753.43 | 1504.84 | 47.00 | 209-222 |  | + | 1 |
| **65** | QEPVLGPVRGPFPII | 15 | Beta Casein | 2 | 809.97 | 1617.92 | 48.89 | 209-223 |  | + | 1 |
| **66** | QEPVLGPVRGPFPIIV | 16 | Beta Casein | 2 | 859.50 | 1716.99 | 49.77 | 209-224 |  | + | 1 |
| **67** | TDVENLHLPLPLLQS | 15 | Beta Casein | 2 | 844.96 | 1687.91 | 50.47 | 143-157 |  | + | 1 |
| **68** | VLGPVRGPFPIIV | 13 | Beta Casein | 2 | 682.43 | 1362.84 | 48.97 | 212-224 |  | + | 1 |
| **69** | VLPVPQKAVPYPQ | 13 | Beta Casein | 2 | 718.42 | 1434.82 | 41.90 | 185-197 |  | + | 1 |
| **70** | WMH | 3 | Beta Casein | 1 | 473.20 | 472.19 | 55.04 | 158-160 |  | + | 1 |
| **71** | YQEPVLGPVRGP | 12 | Beta Casein | 2 | 656.36 | 1310.70 | 41.80 | 208-219 |  | + | 1 |
| **72** | YQEPVLGPVRGPFP | 14 | Beta Casein | 2 | 778.42 | 1554.82 | 45.20 | 208-221 |  | + | 1 |
| **73** | YQEPVLGPVRGPFPI | 15 | Beta Casein | 2 | 834.96 | 1667.90 | 47.59 | 208-222 |  | + | 1 |
| **74** | YQEPVLGPVRGPFPII | 16 | Beta Casein | 2 | 891.50 | 1780.99 | 49.38 | 208-223 |  | + | 1 |
| **75** | YQEPVLGPVRGPFPIIV | 17 | Beta Casein | 2 | 941.04 | 1880.06 | 50.21 | 208-224 |  | + | 1 |
| **76** | NQFLPYPYYAK | 11 | Kappa Casein | 2 | 702.35 | 1402.69 | 43.84 | 74-84 |  | + | 1 |
| **77** | PEVIESPPEINTVQVTSTAV | 20 | Kappa Casein | 2 | 1055.55 | 2109.08 | 46.14 | 171-190 |  | + | 1 |
| **78** | SPPEINTVQVTSTAV | 15 | Kappa Casein | 2 | 771.90 | 1541.79 | 43.77 | 176-190 |  | + | 1 |
| **79** | VQVTSTAV | 8 | Kappa Casein | 1 | 804.45 | 803.44 | 33.63 | 183-190 |  | + | 1 |
| **80** | DAFLGSFLYEYSR | 13 | Serum Albumin | 2 | 784.38 | 1566.74 | 50.49 | 347-359 | + | + | 2 |
| **81** | HLVDEPQNLIK | 11 | Serum Albumin | 2 | 653.36 | 1304.71 | 37.80 | 402-412 | + |  | 1 |
| **82** | KVPQVSTPTLVEVSR | 15 | Serum Albumin | 3 | 547.32 | 1638.93 | 41.05 | 437-451 | + |  | 1 |
| **83** | YNGVFQECCQAEDK | 14 | Serum Albumin | 2 | 874.36 | 1746.70 | 33.89 | 184-197 | + |  | 1 |
| **84** | GLVLIAFSQYLQQCPFDEHVK | 21 | Serum Albumin | 3 | 831.43 | 2491.26 | 49.91 | 45-65 |  | + | 1 |
| **85** | LGEYGFQNALIVR | 13 | Serum Albumin | 2 | 740.40 | 1478.79 | 45.06 | 421-433 |  | + | 1 |
| **86** | TVMENFVAFVDK | 12 | Serum Albumin | 2 | 700.35 | 1398.69 | 47.48 | 569-580 |  | + | 1 |
| **87** | YSR | 3 | Serum Albumin | 1 | 425.21 | 424.21 | 65.86 | 17-19 |  | + | 1 |

**Supplementary Table 13.** Milk peptides identified throughout the 28 days of storage period using the *E. faecium* NCCB 100527.

|  | **Sequence** | **Length** | **Leading proteins** | **Charge** | **m/z** | **Mass** | **Retention time** | **Sequence** | **Day 1** | **Day 28** | **Incidence** |
| --- | --- | --- | --- | --- | --- | --- | --- | --- | --- | --- | --- |
| **1** | DIPNPIGSENSEK | 13 | Alpha S1 Casein | 2 | 700.34 | 1398.66 | 38.41 | 196-208 |  |  | 1 |
| **2** | DIPNPIGSENSEKTTMP | 17 | Alpha S1 Casein | 2 | 915.43 | 1828.85 | 42.84 | 196-212 |  | + | 1 |
| **3** | DIPNPIGSENSEKTTMPLW | 19 | Alpha S1 Casein | 2 | 1065.01 | 2128.01 | 47.82 | 196-214 |  | + | 1 |
| **4** | ENLLRF | 6 | Alpha S1 Casein | 2 | 396.22 | 790.43 | 43.68 | 33-38 |  | + | 1 |
| **5** | EVLNENLLR | 9 | Alpha S1 Casein | 2 | 550.31 | 1098.60 | 41.85 | 29-37 |  | + | 1 |
| **6** | EVLNENLLRF | 10 | Alpha S1 Casein | 2 | 623.84 | 1245.67 | 46.21 | 29-38 |  | + | 1 |
| **7** | FFVAPFPE | 8 | Alpha S1 Casein | 1 | 953.48 | 952.47 | 49.99 | 38-45 |  | + | 1 |
| **8** | FFVAPFPEVFGK | 12 | Alpha S1 Casein | 2 | 692.87 | 1383.72 | 49.69 | 38-49 |  | + | 1 |
| **9** | FFVAPFPEVFGKE | 13 | Alpha S1 Casein | 2 | 757.39 | 1512.77 | 49.73 | 38-50 |  | + | 1 |
| **10** | FFVAPFPEVFGKEK | 14 | Alpha S1 Casein | 2 | 821.44 | 1640.86 | 46.51 | 38-51 |  | + | 1 |
| **11** | FPEV | 4 | Alpha S1 Casein | 1 | 491.25 | 490.24 | 42.57 | 43-46 |  | + | 1 |
| **12** | FPEVFGK | 7 | Alpha S1 Casein | 2 | 412.22 | 822.43 | 42.26 | 43-49 |  | + | 1 |
| **13** | FPEVFGKE | 8 | Alpha S1 Casein | 2 | 476.74 | 951.47 | 42.55 | 43-50 |  | + | 1 |
| **14** | FSDIPNPIGSEN | 12 | Alpha S1 Casein | 2 | 645.30 | 1288.59 | 44.59 | 194-205 |  | + | 1 |
| **15** | FSDIPNPIGSENSEK | 15 | Alpha S1 Casein | 2 | 817.39 | 1632.76 | 41.77 | 194-208 |  | + | 1 |
| **16** | FSDIPNPIGSENSEKT | 16 | Alpha S1 Casein | 2 | 867.91 | 1733.81 | 41.88 | 194-209 |  | + | 1 |
| **17** | FSDIPNPIGSENSEKTT | 17 | Alpha S1 Casein | 2 | 918.44 | 1834.86 | 41.92 | 194-210 |  | + | 1 |
| **18** | FSDIPNPIGSENSEKTTMP | 19 | Alpha S1 Casein | 2 | 1040.48 | 2078.95 | 42.14 | 194-212 |  | + | 1 |
| **19** | FSDIPNPIGSENSEKTTMPL | 20 | Alpha S1 Casein | 2 | 1089.03 | 2176.04 | 44.98 | 194-213 |  | + | 1 |
| **20** | FSDIPNPIGSENSEKTTMPLW | 21 | Alpha S1 Casein | 2 | 1182.06 | 2362.12 | 47.56 | 194-214 |  | + | 1 |
| **21** | FVAPFPEVFGK | 11 | Alpha S1 Casein | 2 | 619.33 | 1236.65 | 47.20 | 39-49 |  | + | 1 |
| **22** | FVAPFPEVFGKE | 12 | Alpha S1 Casein | 2 | 683.86 | 1365.70 | 47.24 | 39-50 |  | + | 1 |
| **23** | FVAPFPEVFGKEK | 13 | Alpha S1 Casein | 2 | 747.90 | 1493.79 | 44.51 | 39-51 |  | + | 1 |
| **24** | FVAPFPEVFGKEKV | 14 | Alpha S1 Casein | 3 | 531.96 | 1592.86 | 45.04 | 39-52 |  | + | 1 |
| **25** | GLPQEVLNENLLR | 13 | Alpha S1 Casein | 2 | 747.92 | 1493.82 | 45.19 | 25-37 |  | + | 1 |
| **26** | GLPQEVLNENLLRF | 14 | Alpha S1 Casein | 2 | 821.45 | 1640.89 | 48.94 | 25-38 |  | + | 1 |
| **27** | IPNPIGSENSEK | 12 | Alpha S1 Casein | 2 | 642.83 | 1283.64 | 32.65 | 197-208 |  | + | 1 |
| **28** | IPNPIGSENSEKTT | 14 | Alpha S1 Casein | 2 | 743.87 | 1485.73 | 33.46 | 197-210 |  | + | 1 |
| **29** | IPNPIGSENSEKTTMP | 16 | Alpha S1 Casein | 2 | 857.92 | 1713.82 | 40.71 | 197-212 |  | + | 1 |
| **30** | IPNPIGSENSEKTTMPL | 17 | Alpha S1 Casein | 2 | 914.46 | 1826.91 | 43.31 | 197-213 |  | + | 1 |
| **31** | IPNPIGSENSEKTTMPLW | 18 | Alpha S1 Casein | 2 | 1007.50 | 2012.99 | 45.93 | 197-214 |  | + | 1 |
| **32** | LFR | 3 | Alpha S1 Casein | 1 | 435.27 | 434.26 | 30.99 | 164-166 |  | + | 1 |
| **33** | NENLLRF | 7 | Alpha S1 Casein | 2 | 453.25 | 904.48 | 43.46 | 32-38 |  | + | 1 |
| **34** | NPIGSENSEK | 10 | Alpha S1 Casein | 2 | 537.76 | 1073.50 | 18.35 | 199-208 |  | + | 1 |
| **35** | NPIGSENSEKTT | 12 | Alpha S1 Casein | 2 | 638.80 | 1275.59 | 20.37 | 199-210 |  | + | 1 |
| **36** | NPIGSENSEKTTMP | 14 | Alpha S1 Casein | 2 | 752.85 | 1503.69 | 31.29 | 199-212 |  | + | 1 |
| **37** | NPIGSENSEKTTMPL | 15 | Alpha S1 Casein | 2 | 809.39 | 1616.77 | 39.58 | 199-213 |  | + | 1 |
| **38** | NPIGSENSEKTTMPLW | 16 | Alpha S1 Casein | 2 | 902.43 | 1802.85 | 44.55 | 199-214 |  | + | 1 |
| **39** | PNPIGSENSEKTTMPL | 16 | Alpha S1 Casein | 2 | 857.92 | 1713.82 | 40.71 | 198-213 |  | + | 1 |
| **40** | QEVLNENLL | 9 | Alpha S1 Casein | 2 | 536.29 | 1070.56 | 45.82 | 28-36 |  | + | 1 |
| **41** | QEVLNENLLR | 10 | Alpha S1 Casein | 2 | 614.34 | 1226.66 | 42.02 | 28-37 |  | + | 1 |
| **42** | QEVLNENLLRF | 11 | Alpha S1 Casein | 2 | 687.87 | 1373.73 | 46.08 | 28-38 |  | + | 1 |
| **43** | QGLPQEVLNENLL | 13 | Alpha S1 Casein | 2 | 733.90 | 1465.78 | 48.75 | 24-36 |  | + | 1 |
| **44** | QGLPQEVLNENLLR | 14 | Alpha S1 Casein | 2 | 811.95 | 1621.88 | 45.07 | 24-37 |  | + | 1 |
| **45** | QGLPQEVLNENLLRF | 15 | Alpha S1 Casein | 2 | 885.48 | 1768.95 | 48.74 | 24-38 |  | + | 1 |
| **46** | QKEPMIGVNQEL | 12 | Alpha S1 Casein | 2 | 693.36 | 1384.70 | 41.55 | 146-157 |  | + | 1 |
| **47** | SDIPNPIGSENSEK | 14 | Alpha S1 Casein | 2 | 743.85 | 1485.69 | 36.67 | 195-208 |  | + | 1 |
| **48** | SDIPNPIGSENSEKTTMP | 18 | Alpha S1 Casein | 2 | 958.95 | 1915.88 | 41.95 | 195-212 |  | + | 1 |
| **49** | SDIPNPIGSENSEKTTMPL | 19 | Alpha S1 Casein | 2 | 1015.49 | 2028.97 | 43.94 | 195-213 |  | + | 1 |
| **50** | SDIPNPIGSENSEKTTMPLW | 20 | Alpha S1 Casein | 2 | 1108.53 | 2215.05 | 46.49 | 195-214 |  | + | 1 |
| **51** | TDAPSFSDIPNPIGSENSEK | 20 | Alpha S1 Casein | 2 | 1052.99 | 2103.96 | 44.19 | 189-208 |  | + | 1 |
| **52** | TDAPSFSDIPNPIGSENSEKTTMPLW | 26 | Alpha S1 Casein | 2 | 1417.66 | 2833.31 | 48.99 | 189-214 |  | + | 1 |
| **53** | VAPFPEVFGK | 10 | Alpha S1 Casein | 2 | 545.80 | 1089.59 | 44.83 | 40-49 |  | + | 1 |
| **54** | VAPFPEVFGKE | 11 | Alpha S1 Casein | 2 | 610.32 | 1218.63 | 44.89 | 40-50 |  | + | 1 |
| **55** | VAPFPEVFGKEK | 12 | Alpha S1 Casein | 2 | 674.37 | 1346.72 | 42.43 | 40-51 |  | + | 1 |
| **56** | VAPFPEVFGKEKV | 13 | Alpha S1 Casein | 2 | 723.90 | 1445.79 | 43.16 | 40-52 |  | + | 1 |
| **57** | VAPFPEVFGKEKVN | 14 | Alpha S1 Casein | 2 | 780.92 | 1559.83 | 42.63 | 40-53 |  | + | 1 |
| **58** | VLNENLLR | 8 | Alpha S1 Casein | 2 | 485.79 | 969.56 | 38.88 | 30-37 |  | + | 1 |
| **59** | VLNENLLRF | 9 | Alpha S1 Casein | 2 | 559.32 | 1116.63 | 45.33 | 30-38 |  | + | 1 |
| **60** | YKVPQLEIVPN | 11 | Alpha S1 Casein | 2 | 650.37 | 1298.72 | 43.96 | 119-129 |  | + | 1 |
| **61** | YTD | 3 | Alpha S1 Casein | 1 | 398.16 | 397.15 | 52.29 | 188-190 |  | + | 1 |
| **62** | ALPQYLK | 7 | Alpha S2 Casein | 2 | 416.75 | 831.49 | 36.11 | 190-196 |  | + | 1 |
| **63** | ALPQYLKT | 8 | Alpha S2 Casein | 2 | 467.27 | 932.53 | 38.59 | 190-197 |  | + | 1 |
| **64** | ALPQYLKTVYQ | 11 | Alpha S2 Casein | 2 | 662.37 | 1322.72 | 43.09 | 190-200 |  | + | 1 |
| **65** | AMKPWIQP | 8 | Alpha S2 Casein | 2 | 485.76 | 969.51 | 42.01 | 204-211 |  | + | 1 |
| **66** | FALPQYLK | 8 | Alpha S2 Casein | 2 | 490.28 | 978.55 | 44.04 | 189-196 |  | + | 1 |
| **67** | FALPQYLKT | 9 | Alpha S2 Casein | 2 | 540.81 | 1079.60 | 44.31 | 189-197 |  | + | 1 |
| **68** | FTCLLAVALAK | 11 | Alpha S2 Casein | 3 | 402.90 | 1205.68 | 6.60 | 6-16 |  | + | 1 |
| **69** | IVLNPWDQVK | 10 | Alpha S2 Casein | 2 | 606.34 | 1210.67 | 44.18 | 119-128 |  | + | 1 |
| **70** | NAVPITPTLN | 10 | Alpha S2 Casein | 2 | 520.29 | 1038.57 | 43.59 | 130-139 |  | + | 1 |
| **71** | NAVPITPTLNR | 11 | Alpha S2 Casein | 2 | 598.34 | 1194.67 | 38.40 | 130-140 |  | + | 1 |
| **72** | NAVPITPTLNRE | 12 | Alpha S2 Casein | 2 | 662.86 | 1323.71 | 39.02 | 130-141 |  | + | 1 |
| **73** | QGPIVLNPWDQVK | 13 | Alpha S2 Casein | 2 | 747.41 | 1492.80 | 45.05 | 116-128 |  | + | 1 |
| **74** | QKFALPQY | 8 | Alpha S2 Casein | 2 | 497.77 | 993.53 | 42.05 | 187-194 |  | + | 1 |
| **75** | QKFALPQYL | 9 | Alpha S2 Casein | 2 | 554.31 | 1106.61 | 44.88 | 187-195 |  | + | 1 |
| **76** | QKFALPQYLK | 10 | Alpha S2 Casein | 2 | 618.36 | 1234.71 | 41.74 | 187-196 |  | + | 1 |
| **77** | QKFALPQYLKT | 11 | Alpha S2 Casein | 2 | 668.88 | 1335.76 | 42.15 | 187-197 |  | + | 1 |
| **78** | TEEEKNRLN | 9 | Alpha S2 Casein | 2 | 566.78 | 1131.55 | 15.26 | 169-177 |  | + | 1 |
| **79** | TEEEKNRLNFL | 11 | Alpha S2 Casein | 2 | 696.86 | 1391.70 | 39.91 | 169-179 |  | + | 1 |
| **80** | VDD | 3 | Alpha S2 Casein | 1 | 348.14 | 347.13 | 44.41 | 88-90 |  | + | 1 |
| **81** | VIPYVRY | 7 | Alpha S2 Casein | 2 | 455.26 | 908.51 | 41.19 | 215-221 |  | + | 1 |
| **82** | VIPYVRYL | 8 | Alpha S2 Casein | 2 | 511.81 | 1021.60 | 44.82 | 215-222 |  | + | 1 |
| **83** | WIQPKT | 6 | Alpha S2 Casein | 2 | 386.72 | 771.43 | 30.06 | 208-213 |  | + | 1 |
| **84** | YQGPIVLNPWDQVK | 14 | Alpha S2 Casein | 2 | 828.94 | 1655.87 | 45.73 | 115-128 |  | + | 1 |
| **85** | YQGPIVLNPWDQVKR | 15 | Alpha S2 Casein | 3 | 605.00 | 1811.97 | 43.87 | 115-129 |  | + | 1 |
| **86** | YQKFALPQ | 8 | Alpha S2 Casein | 2 | 497.77 | 993.53 | 39.07 | 186-193 |  | + | 1 |
| **87** | YQKFALPQY | 9 | Alpha S2 Casein | 2 | 579.30 | 1156.59 | 42.70 | 186-194 |  | + | 1 |
| **88** | YQKFALPQYL | 10 | Alpha S2 Casein | 2 | 635.85 | 1269.68 | 45.29 | 186-195 |  | + | 1 |
| **89** | YQKFALPQYLK | 11 | Alpha S2 Casein | 2 | 699.89 | 1397.77 | 42.26 | 186-196 |  | + | 1 |
| **90** | AVPYPQRD | 8 | Beta Casein | 2 | 473.24 | 944.47 | 25.93 | 192-199 |  | + | 1 |
| **91** | AVPYPQRDMPI | 11 | Beta Casein | 2 | 643.83 | 1285.65 | 41.92 | 192-202 |  | + | 1 |
| **92** | AVPYPQRDMPIQ | 12 | Beta Casein | 2 | 707.86 | 1413.71 | 39.53 | 192-203 |  | + | 1 |
| **93** | AVPYPQRDMPIQA | 13 | Beta Casein | 2 | 743.38 | 1484.74 | 41.29 | 192-204 |  | + | 1 |
| **94** | AVPYPQRDMPIQAFL | 15 | Beta Casein | 2 | 873.46 | 1744.90 | 47.70 | 192-206 |  | + | 1 |
| **95** | DELQDKIHPF | 10 | Beta Casein | 2 | 621.31 | 1240.61 | 41.71 | 58-67 |  | + | 1 |
| **96** | DKIHPFAQTQ | 10 | Beta Casein | 2 | 592.81 | 1183.60 | 28.10 | 62-71 |  | + | 1 |
| **97** | DKIHPFAQTQS | 11 | Beta Casein | 2 | 636.32 | 1270.63 | 27.90 | 62-72 |  | + | 1 |
| **98** | DKIHPFAQTQSL | 12 | Beta Casein | 2 | 692.86 | 1383.71 | 37.46 | 62-73 |  | + | 1 |
| **99** | DVENLHLPLPLL | 12 | Beta Casein | 2 | 686.90 | 1371.78 | 52.99 | 144-155 |  | + | 1 |
| **100** | DVENLHLPLPLLQS | 14 | Beta Casein | 2 | 794.44 | 1586.87 | 51.43 | 144-157 |  | + | 1 |
| **101** | EAMAPKH | 7 | Beta Casein | 2 | 392.19 | 782.37 | 14.82 | 115-121 |  | + | 1 |
| **102** | EMPFPKYPVEP | 11 | Beta Casein | 2 | 667.33 | 1332.64 | 44.05 | 123-133 |  | + | 1 |
| **103** | EPVLGPVRGP | 10 | Beta Casein | 2 | 510.80 | 1019.58 | 38.70 | 210-219 |  | + | 1 |
| **104** | EPVLGPVRGPFP | 12 | Beta Casein | 2 | 632.86 | 1263.70 | 44.73 | 210-221 |  | + | 1 |
| **105** | EPVLGPVRGPFPI | 13 | Beta Casein | 2 | 689.40 | 1376.78 | 47.04 | 210-222 |  | + | 1 |
| **106** | EPVLGPVRGPFPII | 14 | Beta Casein | 2 | 745.94 | 1489.87 | 48.96 | 210-223 |  | + | 1 |
| **107** | EPVLGPVRGPFPIIV | 15 | Beta Casein | 2 | 795.47 | 1588.93 | 49.83 | 210-224 |  | + | 1 |
| **108** | EQQQTEDELQDK | 12 | Beta Casein | 2 | 745.83 | 1489.65 | 23.59 | 52-63 |  | + | 1 |
| **109** | GPVRGPFPIIV | 11 | Beta Casein | 2 | 576.35 | 1150.69 | 46.80 | 214-224 |  | + | 1 |
| **110** | IHPFAQTQ | 8 | Beta Casein | 2 | 471.25 | 940.48 | 29.56 | 64-71 |  | + | 1 |
| **111** | IHPFAQTQS | 9 | Beta Casein | 2 | 514.76 | 1027.51 | 28.91 | 64-72 |  | + | 1 |
| **112** | IHPFAQTQSL | 10 | Beta Casein | 2 | 571.30 | 1140.59 | 39.23 | 64-73 |  | + | 1 |
| **113** | KVLPVPQ | 7 | Beta Casein | 2 | 390.75 | 779.49 | 32.87 | 184-190 |  | + | 1 |
| **114** | LGPVRGPF | 8 | Beta Casein | 2 | 421.75 | 841.48 | 42.01 | 213-220 |  | + | 1 |
| **115** | LGPVRGPFP | 9 | Beta Casein | 2 | 470.27 | 938.53 | 42.54 | 213-221 |  | + | 1 |
| **116** | LGPVRGPFPI | 10 | Beta Casein | 2 | 526.82 | 1051.62 | 45.22 | 213-222 |  | + | 1 |
| **117** | LGPVRGPFPIIV | 12 | Beta Casein | 2 | 632.89 | 1263.77 | 48.23 | 213-224 |  | + | 1 |
| **118** | LLYQEPVLGPVRGP | 14 | Beta Casein | 2 | 769.44 | 1536.87 | 44.42 | 206-219 |  | + | 1 |
| **119** | LPLP | 4 | Beta Casein | 1 | 439.29 | 438.28 | 44.10 | 150-153 |  | + | 1 |
| **120** | LYQEPVLGPVRGP | 13 | Beta Casein | 2 | 712.90 | 1423.78 | 42.72 | 207-219 |  | + | 1 |
| **121** | LYQEPVLGPVRGPFP | 15 | Beta Casein | 2 | 834.96 | 1667.90 | 45.71 | 207-221 |  | + | 1 |
| **122** | LYQEPVLGPVRGPFPI | 16 | Beta Casein | 2 | 891.50 | 1780.99 | 48.13 | 207-222 |  | + | 1 |
| **123** | LYQEPVLGPVRGPFPII | 17 | Beta Casein | 2 | 948.04 | 1894.07 | 49.81 | 207-223 |  | + | 1 |
| **124** | LYQEPVLGPVRGPFPIIV | 18 | Beta Casein | 2 | 997.58 | 1993.14 | 50.60 | 207-224 |  | + | 1 |
| **125** | MPFPKY | 6 | Beta Casein | 2 | 391.70 | 781.38 | 40.19 | 124-129 |  | + | 1 |
| **126** | MPFPKYPVEP | 10 | Beta Casein | 2 | 602.81 | 1203.60 | 43.40 | 124-133 |  | + | 1 |
| **127** | NLHLPLPLL | 9 | Beta Casein | 2 | 515.33 | 1028.64 | 50.29 | 147-155 |  | + | 1 |
| **128** | NLHLPLPLLQ | 10 | Beta Casein | 2 | 579.36 | 1156.70 | 49.46 | 147-156 |  | + | 1 |
| **129** | NLHLPLPLLQS | 11 | Beta Casein | 2 | 622.87 | 1243.73 | 49.18 | 147-157 |  | + | 1 |
| **130** | NVPGEIVESLSSSEESITR | 19 | Beta Casein | 2 | 1017.01 | 2032.00 | 45.71 | 22-40 |  | + | 1 |
| **131** | NVPGEIVESLSSSEESITRIN | 21 | Beta Casein | 2 | 1130.57 | 2259.12 | 47.18 | 22-42 |  | + | 1 |
| **132** | PVEPFTESQSL | 11 | Beta Casein | 2 | 617.30 | 1232.59 | 44.64 | 130-140 |  | + | 1 |
| **133** | PVLGPVRGPFPIIV | 14 | Beta Casein | 2 | 730.95 | 1459.89 | 49.55 | 211-224 |  | + | 1 |
| **134** | QEPVLGPVRGP | 11 | Beta Casein | 2 | 574.82 | 1147.63 | 38.84 | 209-219 |  | + | 1 |
| **135** | QEPVLGPVRGPF | 12 | Beta Casein | 2 | 648.36 | 1294.70 | 44.30 | 209-220 |  | + | 1 |
| **136** | QEPVLGPVRGPFP | 13 | Beta Casein | 2 | 696.89 | 1391.76 | 44.54 | 209-221 |  | + | 1 |
| **137** | QEPVLGPVRGPFPI | 14 | Beta Casein | 2 | 753.43 | 1504.84 | 46.77 | 209-222 |  | + | 1 |
| **138** | QEPVLGPVRGPFPII | 15 | Beta Casein | 2 | 809.97 | 1617.92 | 48.63 | 209-223 |  | + | 1 |
| **139** | QEPVLGPVRGPFPIIV | 16 | Beta Casein | 2 | 859.50 | 1716.99 | 49.50 | 209-224 |  | + | 1 |
| **140** | SKVLPVPQ | 8 | Beta Casein | 2 | 434.27 | 866.52 | 34.45 | 183-190 |  | + | 1 |
| **141** | SLPQNIPPLTQTP | 13 | Beta Casein | 2 | 703.39 | 1404.76 | 45.20 | 84-96 |  | + | 1 |
| **142** | SLVYPFPGPIPN | 12 | Beta Casein | 2 | 650.85 | 1299.69 | 49.56 | 72-83 |  | + | 1 |
| **143** | SQSKVLPVPQ | 10 | Beta Casein | 2 | 541.81 | 1081.61 | 34.58 | 181-190 |  | + | 1 |
| **144** | TDVENLHLP | 9 | Beta Casein | 2 | 519.27 | 1036.52 | 43.11 | 143-151 |  | + | 1 |
| **145** | TDVENLHLPLPLL | 13 | Beta Casein | 2 | 737.42 | 1472.82 | 51.60 | 143-155 |  | + | 1 |
| **146** | TDVENLHLPLPLLQS | 15 | Beta Casein | 2 | 844.96 | 1687.91 | 50.24 | 143-157 |  | + | 1 |
| **147** | TEDELQDKIHPF | 12 | Beta Casein | 2 | 736.36 | 1470.70 | 41.52 | 56-67 |  | + | 1 |
| **148** | VKEAMAPK | 8 | Beta Casein | 2 | 437.25 | 872.48 | 16.10 | 113-120 |  | + | 1 |
| **149** | VLGPVRGP | 8 | Beta Casein | 2 | 397.75 | 793.48 | 32.33 | 212-219 |  | + | 1 |
| **150** | VLGPVRGPF | 9 | Beta Casein | 2 | 471.28 | 940.55 | 42.96 | 212-220 |  | + | 1 |
| **151** | VLGPVRGPFP | 10 | Beta Casein | 2 | 519.81 | 1037.60 | 43.31 | 212-221 |  | + | 1 |
| **152** | VLGPVRGPFPI | 11 | Beta Casein | 2 | 576.35 | 1150.69 | 45.74 | 212-222 |  | + | 1 |
| **153** | VLGPVRGPFPII | 12 | Beta Casein | 2 | 632.89 | 1263.77 | 47.86 | 212-223 |  | + | 1 |
| **154** | VLGPVRGPFPIIV | 13 | Beta Casein | 2 | 682.43 | 1362.84 | 48.74 | 212-224 |  | + | 1 |
| **155** | VLPVPQKAVPYPQ | 13 | Beta Casein | 2 | 718.42 | 1434.82 | 41.91 | 185-197 |  | + | 1 |
| **156** | VPPF | 4 | Beta Casein | 1 | 459.26 | 458.25 | 42.53 | 99-102 |  | + | 1 |
| **157** | VPYPQRDMPIQ | 11 | Beta Casein | 2 | 672.34 | 1342.67 | 38.08 | 193-203 |  | + | 1 |
| **158** | YPQRDMPIQA | 10 | Beta Casein | 2 | 609.80 | 1217.59 | 35.06 | 195-204 |  | + | 1 |
| **159** | YPQRDMPIQAF | 11 | Beta Casein | 2 | 683.33 | 1364.65 | 43.29 | 195-205 |  | + | 1 |
| **160** | YPQRDMPIQAFL | 12 | Beta Casein | 2 | 739.88 | 1477.74 | 46.89 | 195-206 |  | + | 1 |
| **161** | YQEPVLGPVRGP | 12 | Beta Casein | 2 | 656.36 | 1310.70 | 41.65 | 208-219 |  | + | 1 |
| **162** | YQEPVLGPVRGPF | 13 | Beta Casein | 2 | 729.89 | 1457.77 | 44.84 | 208-220 |  | + | 1 |
| **163** | YQEPVLGPVRGPFP | 14 | Beta Casein | 2 | 778.42 | 1554.82 | 45.07 | 208-221 |  | + | 1 |
| **164** | YQEPVLGPVRGPFPI | 15 | Beta Casein | 2 | 834.96 | 1667.90 | 47.34 | 208-222 |  | + | 1 |
| **165** | YQEPVLGPVRGPFPII | 16 | Beta Casein | 2 | 891.50 | 1780.99 | 49.12 | 208-223 |  | + | 1 |
| **166** | YQEPVLGPVRGPFPIIV | 17 | Beta Casein | 2 | 941.04 | 1880.06 | 49.93 | 208-224 |  | + | 1 |
| **167** | FLPYPYYAKP | 10 | Kappa Casein | 2 | 629.83 | 1257.64 | 43.53 | 76-85 |  | + | 1 |
| **168** | FLPYPYYAKPA | 11 | Kappa Casein | 2 | 665.35 | 1328.68 | 43.44 | 76-86 |  | + | 1 |
| **169** | IAKYIPI | 7 | Kappa Casein | 2 | 409.26 | 816.51 | 42.19 | 43-49 |  | + | 1 |
| **170** | MMK | 3 | Kappa Casein | 1 | 409.19 | 408.19 | 27.06 | 1-3 |  | + | 1 |
| **171** | SPPEINTVQVT | 11 | Kappa Casein | 2 | 592.81 | 1183.61 | 41.68 | 176-186 |  | + | 1 |
| **172** | SPPEINTVQVTSTAV | 15 | Kappa Casein | 2 | 771.90 | 1541.79 | 43.72 | 176-190 |  | + | 1 |
| **173** | VIESPPEINTVQVTSTAV | 18 | Kappa Casein | 2 | 942.50 | 1882.99 | 45.25 | 173-190 |  | + | 1 |
